# Supplementary material for: Visible light mediated photocatalytic [2 + 2] cycloaddition/ring-opening rearomatization cascade of electron-deficient azaarenes and vinylarenes
Source: Commun Chem. 2020 Oct 2;3:132. doi: 10.1038/s42004-020-00378-x (PMC9814732; doi:10.1038/s42004-020-00378-x)
Supplement: Supplementary file 1 — Supporting Information [file 42004_2020_378_MOESM1_ESM.pdf]

Supplementary Information

Visible Light Mediated Photocatalytic [2+2] Cycloaddition/Ring-Opening Rearomatization Cascade of Electron-Deficient Azaarenes and Vinylarenes

Noelia Salaverri,[1] Rubén Mas-Ballesté,[2,3] Leyre Marzo,*[1] and José Alemán*[1,3]

[1]*Organic Chemistry Department, Módulo 2, Universidad Autónoma de Madrid, 28049 Madrid (Spain);*

[2]*Inorganic Chemistry Department, Módulo 7, Universidad Autónoma de Madrid, 28049 Madrid (Spain);*

[3]*Institute for Advanced Research in Chemical Sciences (IAdChem), Universidad Autónoma de Madrid, Madrid.*

E-mail: jose.aleman@uam.es, leyre.marzo@uam.es

Table of contents

Supplementary Methods. General methods and materials S3

Supplementary Notes 1. Optimization for the photocatalytic reaction with azaarenes (**1**) S4

Supplementary Notes 2. General procedures for the photocatalytic reaction S6

Supplementary Notes 3. Synthesis and characterization of azaarenes (**1**) S22

Supplementary Notes 4. Synthesis and characterization of styryl derivatives from biologically relevant structures……………………………………………………………………………………..S29

Supplementary Notes 5. Mechanistic studies on the photocatalytic reaction... .... S33

Luminescence quenching experiments S33

Regioselectivity of the reaction.. S35

DFT calculations S36

Supplementary Tables 1. Optimized geometries S37

Supplementary Figures 1. NMR Spectra S51

Supplementary References S107

Supplementary Methods

General methods and materials

NMR spectra were acquired on a BRUKER AVANCE 300 or 500 MHz spectrometer running at 300 or 500 MHz for ^{1}H, 75 or 125 MHz for ^{13}C, 282 or 471 MHz for ^{19}F, and are internally referenced to residual solvent signals ($CDCl_3$ referenced at δ 7.26 ppm for ^{1}H NMR and δ 77.2 ppm for ^{13}C NMR). Data for ^{1}H NMR are reported as follows: chemical shift (δ ppm), multiplicity (s = singlet, d = doublet, t = triplet, m = multiplet), coupling constant (Hz) and integration. Data for ^{13}C and ^{19}F are reported in terms of chemical shift.

High-Resolution Mass Spectra (HRMS) was obtained with APCI or ESI. In ESI determination, MassWorks software version 4.0.0.0 (Cerno Bioscience) was used for the formula identification. MassWorks is an MS calibration software which calibrates isotope profiles to achieve high mass accuracy and enables elemental composition determination on conventional mass spectrometers of unit mass resolution allowing highly accurate comparisons between calibrated and theoretical spectra.

Commercial grade reagents and solvents were purchased from Acros Organics, Alfa Aesar, Fluorochem, Sigma-Aldrich and TCI Chemicals, and used as received without further purification unless otherwise stated. DCM, MeCN and THF were purified by passing through a Pure Solv™ column drying system from Innovative Technology, Inc.

Analytical TLC was performed using pre-coated aluminum-backed plates (Merck TLC Silicagel 60 F_{254}) and visualized by ultraviolet irradiation. Chromatographic purification of products was accomplished by flash chromatography using silica gel (Merck Geduran® Si 60) or porous silica gel (LSI Medience Corporation Iatrobeads 6RS-8060).

A custom-made photoreactor setup was used for the photocatalytic reactions (see Figure S1). The vial is placed inside the fitted well in which irradiation takes place at the desired wavelengths (365, 385, 420, 450 or 540 nm) using 380 mW single LEDs. Reaction temperature is kept at 20-25 ºC using a recirculating chiller.

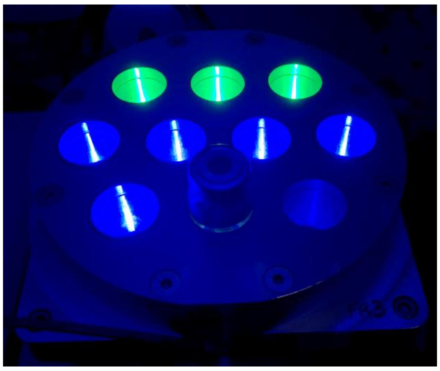

Supplementary Fig. 1. Experimental setup employed during photocatalytic reactions.

UV-Vis measurements were carried out on an Agilent 8453 UV-Visible Spectroscopy System controlled by UV-Visible ChemStation Software. Emission intensities were recorded using a JASCO Spectrofluorometer FP-8600 equipped with a TC-815 Peltier thermostated single cell holder (water-cooled) controlled by Spectra Manager Version 2.10.01. Time resolved emission spectra were recorded using an Edinburg Instruments FS5 Spectrofluorometer, and a 450 nm EPL laser.

Supplementary Notes 1.

Optimization for the photocatalytic reaction with azaarenes (1)

Initially, a solution of the quinoline derivative **1a** in the presence of styrene **2a**, the iridium photocatalyst **3a**, DBU in CH_3CN was stirred under blue LED irradiation for 17 hours obtaining **4a** in 83 % yield (entry 1, Table 1). Then, a variety of photocatalyst with different triplet energies were studied. While the iridium derivatives **3a** and **3c** (entries 1 and 3, Table 1), and the organic photocatalyst **3d** (entry 4, Table 1) afforded the final product in moderate to good yields, $Ru(bpy)_3Cl_2$ **3b** or the Fukuzumi´s catalyst **3e** did not show any reactivity (entries 2 and 5, Table 1). Next, different bases were examined in the reaction (entries 6-10, Table 1). In the absence of base, the reaction proceeded in 38% yield and with other bases it was observed that the stronger the base the higher the yield of the reaction was, being DBU the most adequate one (entry 1, Table 1). Afterwards a variety of non-polar, polar, protic or non-protic solvents were tested in the presence of 2 mol% of **3a** and DBU as base (entries 11-16, Table 1), obtaining the best result with THF (entry 15, Table 1). Decreasing or increasing the equivalents of base, or decreasing the amount of photocatalyst to 1 mol % afforded worst results in every case (entries 17-19, Table 1). Finally, control experiments revealed that either the light source or the photocatalyst are necessary for the reaction, thus confirming the photocatalytic nature of the process (entries 20 and 21, Table 1). Thus, the optimal reaction conditions were determined as 0.1 mmol **1**, 0.5 mmol **2**, 2 mol% **3a**, 0.5 mmol DBU, THF, N_2, 455 nm LED, 17 hours.

Supplementary Table 1. Optimization of the reaction conditions.

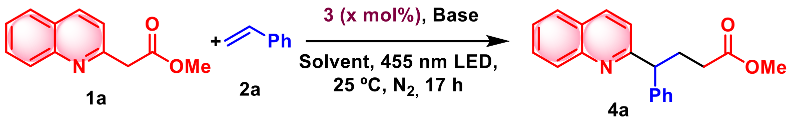

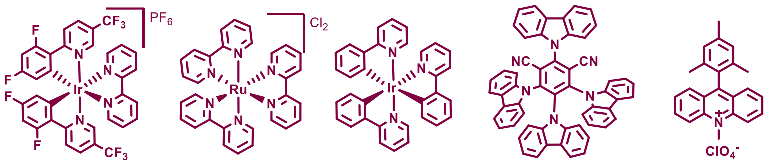

3a E_T = 62 kcal mol^{-1} 3b E_T = 46.5 kcal mol^{-1} 3c E_T = 55 kcal mol^{-1} 3d E_T = 60 kcal mol^{-1} 3e E_T = 44.5 kcal mol^{-1}

Entry	3 (mol%)	Base	Solvent	Yield[b] (%)
1	**3a** (2)	DBU	ACN	83
2	**3b** (3)	DBU	ACN	n.r.
3	**3c** (2)	DBU	ACN	80
4	**3d** (4)	DBU	ACN	43
5	**3e** (5)	DBU	ACN	n.r.
6	**3a** (2)	--	ACN	38
7	**3a** (2)	K_2CO_3	ACN	45
8	**3a** (2)	Fosfate[c]	ACN	56
9	**3a** (2)	DMAP	ACN	70
10	**3a** (2)	NEt_3	ACN	62
11	**3a** (2)	DBU	Toluene	25
12	**3a** (2)	DBU	EtOH	62
13	**3a** (2)	DBU	DMF	69
14	**3a** (2)	DBU	DMSO	82
15	**3a** (2)	DBU	THF	100 (98)
16	**3a** (2)	DBU	DCM	71
17	**3a** (2)	DBU (0.25 equiv.)	THF	97
18	**3a** (2)	DBU (1 equiv.)	THF	87
19	**3a** (1)	DBU	THF	66
20[d]	**3a** (2)	DBU	THF	n.r.
21	-	DBU	THF	n.r.

[a] All the reactions were carried our using 0.1 mmol of **1a**, 0.5 mmol of **2a**, 0.5 equivalents of base and 1 mL of solvent, under 455 nm LED irradiation for 17 h, unless indicated otherwise. [b] Determined by ^{1}H NMR using 1,3,5-trimetoxibencene as internal standard. [c] Fosfate = $(BuO)_2P(O)ONBu_3Me$. [d] Without light.

Supplementary Notes 2

General procedures for the photocatalytic reaction

General procedure A: A dry vial equipped with a magnetic stir bar was charged with **3a** (2.0 mg, 2 μmol, 0.02 equiv.), the corresponding azaarene derivative **1** (0.1 mmol, 1.0 equiv.), DBU (7.6 mg, 0.05 mmol, 0.5 equiv.), alkene **2** (0.5 mmol, 5.0 equiv.) and 1.0 mL of THF (0.1 M). Degasification of the reaction mixture was performed via freeze-pump-thaw cycling (3 x 10 min under vacuum). Then, the reaction mixture was irradiated and stirred in the photoreactor setup at 455 nm for 17 h (unless otherwise stated). The reaction mixture was concentrated under reduced pressure and purified by flash column chromatography (silica gel) to provide the product.

General procedure B: A dry vial equipped with a magnetic stir bar was charged with **3c** (1.4 mg, 2 μmol, 0.02 equiv.), the corresponding azaarene derivative **1** (0.1 mmol, 1.0 equiv.), DBU (7.6 mg, 0.05 mmol, 0.5 equiv.), alkene **2** (0.5 mmol, 5.0 equiv.) and 1.0 mL of THF (0.1 M). Degasification of the reaction mixture was performed via freeze-pump-thaw cycling (3 x 10 min under vacuum). Then, the reaction mixture was irradiated and stirred in the photoreactor setup at 455 nm for 17 h. The reaction mixture was concentrated under reduced pressure and purified by flash column chromatography (silica gel) to provide the product.

General procedure C: A dry vial equipped with a magnetic stir bar was charged with **3c** (1.4 mg, 2 μmol, 0.02 equiv.), the corresponding azaarene derivative **1** (0.1 mmol, 1.0 equiv.), K_2CO_3 (0.1 mmol, 1.0 equiv.), alkene **2** (0.5 mmol, 5.0 equiv.) and 1.0 mL of MeCN (0.1 M). Degasification of the reaction mixture was performed via freeze-pump-thaw cycling (3 x 10 min under vacuum). Then, the reaction mixture was irradiated and stirred in the photoreactor setup at 455 nm for 17 h. The reaction mixture was concentrated under reduced pressure and purified by flash column chromatography (silica gel) to provide the product.

Methyl 4-phenyl-4-(quinolin-2-yl)butanoate (4a)

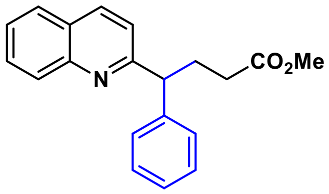

4a

Following **General Procedure A** with compound **1a** and styrene. Purification by column chromatography (silica, 10:1 cyclohexane:EtOAc) afforded 30.0 mg (98%) of the title compound **4a** as a yellowish oil. **R*f*** = 0.44 (5:1 cyclohexane:EtOAc). **^{1}H NMR (300 MHz, $CDCl_3$):** δ 8.10 (d, *J* = 8.4 Hz, 1H), 7.99 (d, *J* = 8.5 Hz, 1H), 7.77 – 7.66 (m, 2H), 7.48 (t, *J* = 7.5 Hz, 1H), 7.38 – 7.19 (m, 6H), 4.28 (t, *J* = 7.7 Hz, 1H), 3.62 (s, 3H), 2.80 – 2.68 (m, 1H), 2.58 – 2.46 (m, 1H), 2.40 – 2.34 (m, 2H). **^{13}C NMR (75 MHz, $CDCl_3$):** δ 173.9, 162.9, 147.8, 142.8, 136.2, 129.4, 129.3, 128.6 (2C), 128.2 (2C), 127.4, 126.9, 126.7, 126.0, 121.3, 53.3, 51.5, 32.4, 29.7. **HRMS (ESI):** calc'd for $C_{20}H_{20}NO_2$ $[M+H]^+$: 306.1489, found: 306.1485.

Methyl 4-(phenyl-*d5*)-4-(quinolin-2-yl)butanoate-3,3,4-*d3* (4a-D)

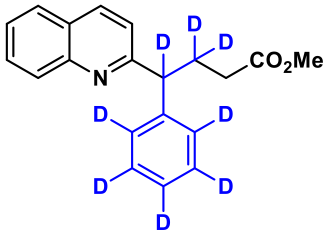

4a-D

Following **General Procedure A** with compound **1a** and styrene-d8. Purification by column chromatography (silica, 10:1 cyclohexane:EtOAc) afforded 25.6 mg (82%) of the title compound **4a-D** as a yellowish oil. **R*f*** = 0.44 (5:1 cyclohexane:EtOAc). **^{1}H NMR (300 MHz, CDCl$_3$):** δ 8.03 (d, *J* = 8.1 Hz, 1H), 7.93 (d, *J* = 9.3 Hz, 1H), 7.66 – 7.59 (m, 2H), 7.40 (t, *J* = 8.1 Hz, 1H), 7.15 (d, *J* = 8.7 Hz, 1H), 3.54 (s, 3H), 2.28 (s, 2H). **^{13}C NMR (75 MHz, CDCl$_3$):** δ 173.9, 162.9, 146.7, 142.4, 135.8, 129.3, 129.2, 128.4 (2C), 128.1 (2C), 127.7, 127.4, 126.9, 126.0, 121.2, 52.5 (t, *J* = 18.0 Hz), 51.5, 32.2, 29.0 (t, *J* = 21.5 Hz). **HRMS (ESI):** calc'd for $C_{20}H_{12}D_8NO_2$ [M+H]$^+$: 314.1991, found: 314.1986.

Methyl 4-(7-chloroquinolin-2-yl)-4-phenylbutanoate (4b)

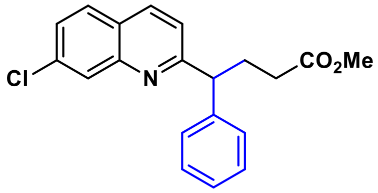

4b

Following **General Procedure A** with compound **1b** and styrene. Purification by column chromatography (silica, gradient from 3% to 30% of EtOAc in cyclohexane) afforded 28.7 mg (84%) of the title compound **4b** as a yellowish oil. **R*f*** = 0.49 (5:1 cyclohexane:EtOAc). **^{1}H NMR (300 MHz, CDCl$_3$):** δ 8.12 (d, *J* = 2.1 Hz, 1H), 7.95 (d, *J* = 8.5 Hz, 1H), 7.66 (d, *J* = 8.6 Hz, 1H), 7.43 (dd, *J* = 8.7, 2.1 Hz, 1H), 7.36 – 7.19 (m, 6H), 4.26 (t, *J* = 7.7 Hz, 1H), 3.63 (s, 3H), 2.79 – 2.67 (m, 1H), 2.56 – 2.44 (m, 1H), 2.38 – 2.33 (m, 2H). **^{13}C NMR (75 MHz, CDCl$_3$):** δ 173.9, 164.1, 148.1, 142.5, 136.0, 135.1, 128.7 (2C), 128.6, 128.5, 128.2 (2C), 127.0, 126.8, 125.2, 121.7, 53.2, 51.5, 32.3, 29.6. **HRMS (ESI):** calc'd for $C_{20}H_{19}ClNO_2$ [M+H]$^+$: 340.1099, found: 340.1085.

Methyl 4-(6-chloroquinolin-2-yl)-4-phenylbutanoate (4c)

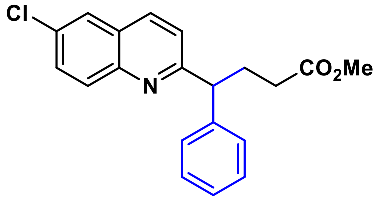

4c

Following **General Procedure A** with compound **1c** and styrene. Purification by column chromatography (silica, gradient from 3% to 30% of EtOAc in cyclohexane) afforded 33.9 mg (99%) of the title compound **4c** as a yellowish oil. **R*f*** = 0.54 (5:1 cyclohexane:EtOAc). **^{1}H NMR (300 MHz, CDCl$_3$):** δ 8.03 (d, *J* = 9.0 Hz, 1H), 7.88 (d, *J* = 8.5 Hz, 1H), 7.71 (d, *J* = 2.3 Hz, 1H), 7.61 (dd, *J* = 9.0, 2.3 Hz, 1H), 7.36 – 7.17 (m, 6H), 4.26 (t, *J* = 7.7 Hz, 1H), 3.62 (s, 3H), 2.79 – 2.67 (m, 1H), 2.56 – 2.44 (m, 1H), 2.38 – 2.33 (m, 2H). In the spectrum is possible to see the signal corresponding to the enol: 5.71 (s, 1H). **^{13}C NMR (75 MHz, CDCl$_3$):** δ 173.8, 163.3, 146.1, 142.5, 135.3, 131.6, 131.0, 130.1, 128.6 (2C), 128.2 (2C), 127.4, 126.8, 126.1, 122.3, 53.4, 51.7, 32.5, 29.8. In the spectrum is possible to see the signal corresponding to the enol: 82.3 ppm. **HRMS (APCI):** calc'd for $C_{20}H_{19}ClNO_2$ [M+H]$^+$: 340.1099, found: 340.1085.

Methyl 4-(6-fluoroquinolin-2-yl)-4-phenylbutanoate (4d)

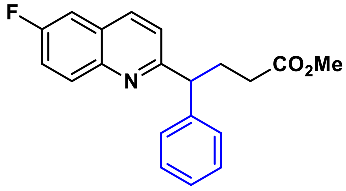

4d

Following **General Procedure A** with compound **1d** and styrene. Purification by column chromatography (silica, gradient from 3% to 30% of EtOAc in cyclohexane) afforded 31.2 mg (96%) of the title compound **4d** as a yellowish oil. **R*f*** = 0.38 (5:1 cyclohexane:EtOAc). **^{1}H NMR (300 MHz, CDCl$_3$):** δ 8.09 (dd, *J* = 9.2, 5.4 Hz, 1H), 7.92 (d, *J* = 8.5 Hz, 1H), 7.48 – 7.41 (m, 1H), 7.38 – 7.16 (m, 7H), 4.26 (t, *J* = 7.7 Hz, 1H), 3.62 (s, 3H), 2.79 – 2.67 (m, 1H), 2.57 – 2.45 (m, 1H), 2.38 – 2.33 (m, 2H). **^{13}C NMR (75 MHz, CDCl$_3$):** δ 173.9, 162.3 (d, *J* = 3.0 Hz), 160.2 (d, *J* = 245.3 Hz), 144.8, 142.7, 135.6 (d, *J* = 5.3 Hz), 131.8 (d, *J* = 9.0 Hz), 128.6 (2C), 128.2 (2C), 127.4 (d, *J* = 9.8 Hz), 126.8, 122.1, 119.3 (d, *J* = 25.5 Hz), 110.4 (d, *J* = 21.0 Hz), 53.1, 51.5, 32.4, 29.7. **^{19}F NMR (282 MHz, CDCl$_3$):** δ -114.5. **HRMS (APCI):** calc'd for $C_{20}H_{19}FNO_2$ [M+H]$^+$: 324.1394, found: 324.1389.

2-(4-Methoxy-4-oxo-1-phenylbutyl)quinoline-6-carboxylic acid (4e)

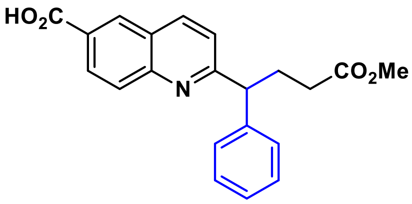

4e

Following **General Procedure A** with compound **1e** and styrene. Purification by column chromatography (silica, gradient from 10:1 to 5:1 of DCM:MeOH) afforded 19.5 mg (56%) of the title compound **4e** as a yellow oil. **R*f*** = 0.31 (10:1 DCM:MeOH). **^{1}H NMR (300 MHz, CDCl$_3$):** δ 8.57 (s, 1H), 8.33 (d, *J* = 8.9 Hz, 1H), 8.00 – 8.23 (m, 2H), 7.37 – 7.23 (m, 5H), 7.21 – 7.18 (m,

1H), 4.32 (t, *J* = 7.5 Hz, 1H), 3.63 (s, 3H), 2.80 – 2.66 (m, 1H), 2.59 – 2.47 (m, 1H), 2.40 – 2.35 (m, 2H). **^{13}C NMR (75 MHz, $CDCl_3$):** δ 173.9 (2C), 165.7, 149.8, 142.2, 137.7, 131.4, 129.5, 129.3, 128.7 (2C), 128.2 (2C), 126.9 (2C), 126.0, 122.2, 53.3, 51.6, 32.3, 29.5. **HRMS (APCI):** calc'd for $C_{21}H_{20}NO_4$ $[M+H]^+$: 350.1387, found: 350.1379.

Methyl 4-(6-methoxyquinolin-2-yl)-4-phenylbutanoate (4f)

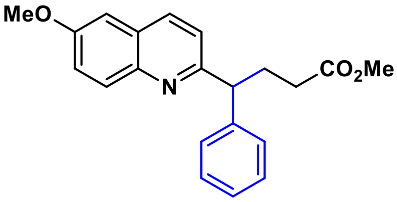

4f

Following **General Procedure A** with compound **1f**, styrene and carrying out the reaction over 30 h. Purification by column chromatography (silica, gradient from 3% to 25% of EtOAc in cyclohexane) afforded 24.6 mg (73%) of the title compound **4f** as a yellowish oil. **R_f** = 0.36 (5:1 cyclohexane:EtOAc). **^{1}H NMR (300 MHz, $CDCl_3$):** δ 7.99 (d, *J* = 9.2 Hz, 1H), 7.89 (d, *J* = 8.4 Hz, 1H), 7.37 – 7.16 (m, 7H), 7.01 (d, *J* = 2.8 Hz, 1H), 4.24 (t, *J* = 7.7 Hz, 1H), 3.90 (s, 3H), 3.62 (s, 3H), 2.77 – 2.65 (m, 1H), 2.55 – 2.43 (m, 1H), 2.38 – 2.33 (m, 2H). **^{13}C NMR (75 MHz, $CDCl_3$):** δ 174.0, 160.5, 157.4, 143.8, 143.1, 135.1, 130.8, 128.6 (2C), 128.2 (2C), 127.7, 126.6, 121.7, 121.5, 105.1, 55.5, 53.1, 51.5, 32.5, 29.8. **HRMS (ESI):** calc'd for $C_{21}H_{22}NO_3$ $[M+H]^+$: 336.1594, found: 336.1606.

***N,N*,4-Triphenyl-4-(quinolin-2-yl)butanamide (4g)**

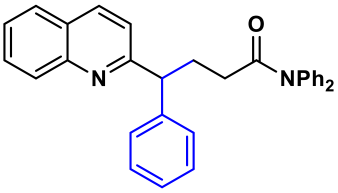

4g

Following **General Procedure A** with compound **1g** and styrene. Purification by column chromatography (silica, gradient from 3% to 50% of EtOAc in cyclohexane) afforded 28.6 mg (65%) of the title compound **4g** as a yellow oil. **R_f** = 0.29 (5:1 cyclohexane:EtOAc). **^{1}H NMR (300 MHz, $CDCl_3$):** δ 8.04 (d, *J* = 8.4 Hz, 1H), 7.95 (d, *J* = 8.5 Hz, 1H), 7.74 – 7.65 (m, 2H), 7.50 – 7.45 (m, 1H), 7.34 – 7.31 (m, 2H), 7.27 – 7.13 (m, 14H), 4.33 (t, *J* = 7.8 Hz, 1H), 2.77 – 2.51 (m, 2H), 2.41 – 2.23 (m, 2H). **^{13}C NMR (75 MHz, $CDCl_3$):** δ 172.8, 163.3, 147.7, 143.0, 142.7 (2C), 136.2, 129.3, 129.1 (3C), 128.5 (6C), 128.2 (6C), 127.4, 126.8, 126.5, 125.9, 121.2, 53.0, 33.3, 30.3. **HRMS (APCI):** calc'd for $C_{31}H_{27}N_2O$ $[M+H]^+$: 443.2118, found: 443.2105.

4-Phenyl-4-(quinolin-2-yl)butanenitrile (4h)

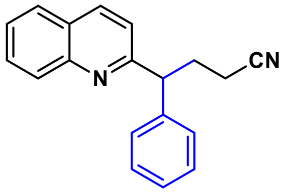

4h

Following **General Procedure B** with compound **1h** and styrene. Purification by column chromatography (silica, gradient from 3% to 30% of EtOAc in cyclohexane) afforded 18.0 mg (70%) of the title compound **4h** as a yellow oil. **R*f*** = 0.27 (5:1 cyclohexane:EtOAc). **^{1}H NMR (300 MHz, CDCl$_3$):** δ 8.10 (d, *J* = 8.4 Hz, 1H), 8.00 (d, *J* = 8.5 Hz, 1H), 7.77 – 7.69 (m, 2H), 7.51 (td, *J* = 8.1, 1.2 Hz, 1H), 7.35 – 7.19 (m, 6H), 4.37 (t, *J* = 7.5 Hz, 1H), 2.92 – 2.82 (m, 1H), 2.54 – 2.31 (m, 3H). **^{13}C NMR (75 MHz, CDCl$_3$):** δ 161.3, 147.6, 141.9, 136.4, 129.4, 129.4, 128.9 (2C), 128.2 (2C), 127.5, 127.1, 126.9, 126.2, 122.0, 119.7, 52.2, 30.1, 15.7. **HRMS (APCI):** calc'd for $C_{19}H_{17}N_2$ [M+H]$^+$: 273.1386, found: 273.1376.

2-(1-Phenyl-3-(phenylsulfonyl)propyl)quinoline (4i)

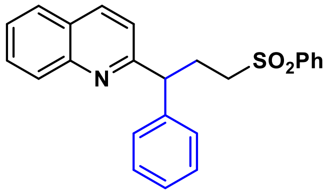

4i

Following **General Procedure B** with compound **1i** and styrene. Purification by column chromatography (silica, gradient from 3% to 30% of EtOAc in cyclohexane) afforded 27.0 mg (61%) of the title compound **4i** as a yellow oil. **R*f*** = 0.28 (5:1 cyclohexane:EtOAc). **^{1}H NMR (300 MHz, CDCl$_3$):** δ 8.02 – 7.90 (m, 4H), 7.76 – 7.48 (m, 7H), 7.32 – 7.27 (m, 2H), 7.23 – 7.13 (m, 3H), 4.34 (t, *J* = 8.3 Hz, 1H), 3.31 – 3.17 (m, 2H), 2.90 – 2.73 (m, 1H), 2.64 – 2.52 (m, 1H). **^{13}C NMR (75 MHz, CDCl$_3$):** δ 161.5, 147.6, 142.0, 139.2, 136.4, 133.5, 129.4, 129.4, 129.2 (2C), 128.8 (2C), 128.1 (2C), 128.1 (2C), 127.5, 127.1, 126.9, 126.2, 121.5, 54.6, 52.0, 27.6. **HRMS (APCI):** calc'd for $C_{24}H_{22}NO_2S$ [M+H]$^+$: 388.1366, found: 388.1353.

1,4-diphenyl-4-(quinolin-2-yl)butan-1-one (4j)

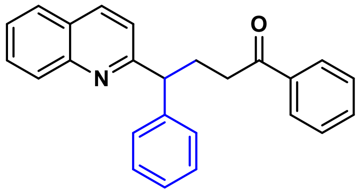

4j

Following **General Procedure C** with compound **1j** and styrene. Purification by column chromatography (silica, gradient from 3% to 30% of EtOAc in cyclohexane) afforded 22.0 mg (64%) of the title compound **4j** as a yellow solid. **R*f*** = 0.33 (5:1 cyclohexane:EtOAc). **^{1}H NMR (300 MHz, $CDCl_3$):** δ 8.11 (d, *J* = 8.5 Hz, 1H), 8.00 (d, *J* = 8.5 Hz, 1H), 7.92 – 7.90 (m, 2H), 7.76 – 7.67 (m, 2H), 7.54 – 7.38 (m, 6H), 7.31 – 7.17 (m, 4H), 4.38 (t, *J* = 7.6 Hz, 1H), 3.07 – 3.00 (m, 2H), 2.95 – 2.81 (m, 1H), 2.66 – 2.55 (m, 1H). **^{13}C NMR (75 MHz, $CDCl_3$):** δ 200.2, 163.2, 147.7, 143.1, 136.9, 136.3, 132.9, 129.3, 129.3, 128.6 (2C), 128.5 (2C), 128.2 (2C), 128.1 (2C), 127.5, 126.9, 126.7, 126.0, 121.4, 53.5, 37.1, 29.4. **HRMS (APCI):** calc'd for $C_{25}H_{22}NO$ $[M+H]^+$: 352.1696, found: 352.1683.

Methyl 4-phenyl-4-(quinolin-4-yl)butanoate (4k)

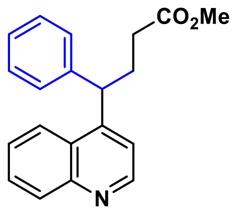

4k

Following **General Procedure B** with compound **1k** and styrene. Purification by column chromatography (silica, gradient from 8% to 40% of EtOAc in cyclohexane) afforded 14.5 mg (47%) of the title compound **4k** as a colorless oil. **R*f*** = 0.18 (4:1 cyclohexane:EtOAc). **^{1}H NMR (300 MHz, $CDCl_3$):** δ 8.89 (d, *J* = 4.5 Hz, 1H), 8.16 – 8.09 (m, 2H), 7.67 (td, *J* = 8.3, 1.3 Hz, 1H), 7.52 (td, *J* = 8.3, 1.2 Hz, 1H), 7.38 – 7.28 (m, 5H). 7.23 – 7.18 (m, 1H), 4.80 (t, *J* = 7.4 Hz, 1H), 3.65 (s, 3H), 2.57 – 2.45 (m, 2H), 2.41 – 2.36 (m, 2H). **^{13}C NMR (75 MHz, $CDCl_3$):** δ 173.5, 150.2, 149.5, 148.6, 142.1, 130.4, 129.0, 128.8 (2C), 128.1 (2C), 127.2, 127.0, 126.6, 123.5, 119.0, 51.7, 44.9, 32.1, 30.4. **HRMS (APCI):** calc'd for $C_{20}H_{20}NO_2$ $[M+H]^+$: 306.1489, found: 306.1483.

Methyl 4-(isoquinolin-2-yl)-4-phenylbutanoate (4l)

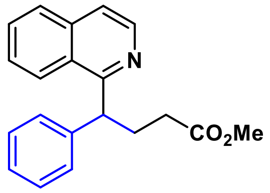

4l

Following **General Procedure A** with compound **1l** and styrene. Purification by column chromatography (silica, 10:1 cyclohexane:EtOAc) afforded 22.9 mg (75%) of the title compound **4l** as a colorless oil. **R*f*** = 0.44 (5:1 cyclohexane:EtOAc). **^{1}H NMR (300 MHz, $CDCl_3$):** δ 8.57 (d, *J* = 5.7 Hz, 1H), 8.20 (d, *J* = 8.7 Hz, 1H), 7.77 (d, *J* = 8.1 Hz, 1H), 7.62 – 7.47 (m, 3H), 7.38 – 7.35 (m, 2H), 7.26 – 7.21 (m, 2H), 7.17 – 7.11 (m, 1H), 4.96 (t, *J* = 7.4 Hz, 1H), 3.63 (s, 3H), 2.86 – 2.75 (m, 1H), 2.59 – 2.27 (m, 3H). **^{13}C NMR (75 MHz, $CDCl_3$):** δ 174.1, 161.2, 143.4, 141.7, 136.5,

129.5, 128.5 (2C), 128.1 (2C), 127.4, 127.2, 127.1, 126.5, 124.9, 119.6, 51.4, 47.6, 32.4, 30.9. **HRMS (APCI):** calc'd for $C_{20}H_{20}NO_2$ $[M+H]^+$: 306.1489, found: 306.1477.

Methyl 4-phenyl-4-(quinoxalin-2-yl)butanoate (4m)

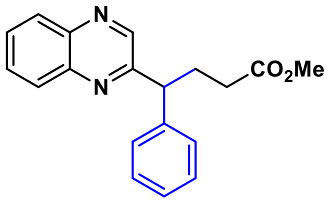

4m

Following **General Procedure A** with compound **1m** and styrene. Purification by column chromatography (silica, gradient from 3% to 30% of EtOAc in cyclohexane) afforded 11.4 mg (37%) of the title compound **4m** as a red solid. **R*f*** = 0.50 (1:1 cyclohexane:EtOAc). **^{1}H NMR (300 MHz, $CDCl_3$):** δ 8.66 (s, 1H), 8.11 (dd, *J* = 7.5, 1.8 Hz, 1H), 8.04 (dd, *J* = 7.9, 1.9 Hz, 1H), 7.79 – 7.68 (m, 2H), 7.37 – 7.19 (m, 5H), 4.36 (t, *J* = 7.6 Hz, 1H), 3.64 (s, 3H), 2.82 – 2.70 (m, 1H), 2.61 – 2.49 (m, 1H), 2.37 (t, *J* = 7.5 Hz, 2H). **^{13}C NMR (75 MHz, $CDCl_3$):** δ 173.7, 157.6, 146.2, 142.0, 141.5, 141.2, 129.9, 129.3, 129.2, 129.1, 128.9 (2C), 128.3 (2C), 127.2, 51.6, 50.8, 32.1, 29.4. **HRMS (APCI):** calc'd for $C_{19}H_{19}N_2O_2$ $[M+H]^+$: 307.1441, found: 307.1436.

1,4-Diphenyl-4-(pyrazin-2-yl)butan-1-one (4o)

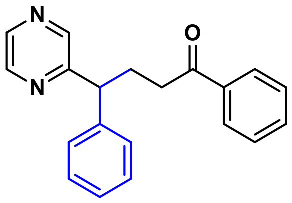

4o

Following **General Procedure C** with compound **1o** and styrene. Purification by column chromatography (silica, gradient from 3% to 30% of EtOAc in cyclohexane) afforded 18.0 mg (61%) of the title compound **4o** as a red solid. **R*f*** = 0.46 (2:1 cyclohexane:EtOAc). **^{1}H NMR (300 MHz, $CDCl_3$):** δ 8.55 – 8.54 (m, 1H), 8.49 – 8.48 (m, 1H), 8.40 – 8.39 (m, 1H), 7.89 – 7.85 (m, 2H), 7.56 – 7.51 (m, 1H), 7.45 – 7.20 (m, 7H), 4.24 (t, *J* = 7.8 Hz, 1H), 2.95 (t, *J* = 7.6 Hz, 2H), 2.77 – 2.50 (m, 2H). **^{13}C NMR (75 MHz, $CDCl_3$):** δ 199.6, 158.8, 144.9, 144.0, 142.5, 141.9, 136.8, 133.0, 128.8 (2C), 128.5 (2C), 128.1 (2C), 128.0 (2C), 127.0, 50.2, 36.4, 29.1. **HRMS (APCI):** calc'd for $C_{20}H_{19}N_2O$ $[M+H]^+$: 303.1492, found: 303.1487.

Methyl 4-(quinolin-2-yl)-4-(*p*-tolyl)butanoate (4p)

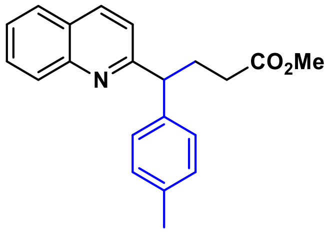

4p

Following **General Procedure A** with **1a** and 4-methylstyrene. Purification by column chromatography (silica, 10:1 cyclohexane:EtOAc) afforded 24.8 mg (78%) of the title compound **4p** as a yellowish oil. **R*f*** = 0.46 (5:1 cyclohexane:EtOAc). **^{1}H NMR (300 MHz, $CDCl_3$):** δ 8.09 (d, *J* = 8.3 Hz, 1H), 7.97 (d, *J* = 8.5 Hz, 1H), 7.73 – 7.65 (m, 2H), 7.47 (t, *J* = 8.1 Hz, 1H), 7.26 – 7.19 (m, 3H), 7.10 – 7.06 (m, 2H), 4.24 (t, *J* = 7.7 Hz, 1H), 3.61 (s, 3H), 2.78 – 2.66 (m, 1H), 2.56 – 2.44 (m, 1H), 2.39 – 2.33 (m, 2H), 2.28 (s, 3H). **^{13}C NMR (75 MHz, $CDCl_3$):** δ 174.0, 163.2, 147.7, 139.8, 136.2, 136.2, 129.4, 129.3 (2C), 129.2, 128.1 (2C), 127.4, 126.9, 125.9, 121.3, 52.9, 51.4, 32.4, 29.7, 21.0. **HRMS (ESI):** calc'd for $C_{21}H_{22}NO_2$ $[M+H]^+$: 320.1651, found: 320.1669.

Methyl 4-(4-methoxyphenyl)-4-(quinolin-2-yl)butanoate (4q)

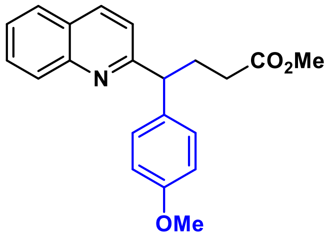

4q

Following **General Procedure A** with **1a** and 4-methoxystyrene. Purification by column chromatography (silica, gradient from 10:1 to 5:1 of cyclohexane:EtOAc) afforded 32.6 mg (97%) of the title compound **4q** as a yellow oil. **R*f*** = 0.36 (5:1 cyclohexane:EtOAc). **^{1}H NMR (300 MHz, $CDCl_3$):** δ 8.09 (d, *J* = 8.5 Hz, 1H), 7.97 (d, *J* = 7.7 Hz, 1H), 7.73 – 7.65 (m, 2H), 7.47 (td, *J* = 8.1, 1.2 Hz, 1H), 7.29 – 7.25 (m, 2H), 7.20 (d, *J* = 8.7 Hz, 1H), 6.84 – 6.81 (m, 2H), 4.23 (t, *J* = 7.7 Hz, 1H), 3.75 (s, 3H), 3.62 (s, 3H), 2.76 – 2.64 (m, 1H), 2.55 – 2.42 (m, 1H), 2.39 – 2.33 (m, 2H). **^{13}C NMR (75 MHz, $CDCl_3$):** δ 174.0, 163.3, 158.4, 147.7, 136.2, 134.9, 129.3, 129.2, 129.1 (2C), 127.4, 126.8, 125.9, 121.2, 114.0 (2C), 55.2, 52.5, 51.4, 32.4, 39.8. **HRMS (ESI):** calc'd for $C_{21}H_{22}NO_3$ $[M+H]^+$: 336.1594, found: 336.1595.

Methyl 4-(3,4-dimethoxyphenyl)-4-(quinolin-2-yl)butanoate (4r)

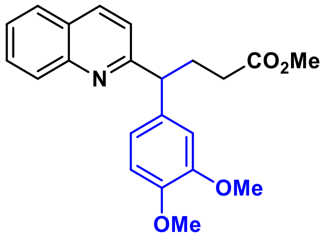

4r

Following **General Procedure A** with **1a** and 3,4-dimethoxystyrene. Purification by column chromatography (silica, gradient from 10:1 to 2:1 of cyclohexane:EtOAc) afforded 34.3 mg (94%) of the title compound **4r** as a yellowish oil. **R*f*** = 0.15 (5:1 cyclohexane:EtOAc). **^{1}H NMR (300 MHz, CDCl$_3$):** δ 8.10 (d, *J* = 8.1 Hz, 1H), 8.00 (d, *J* = 8.5 Hz, 1H), 7.76 – 7.67 (m, 2H), 7.51 – 7.46 (m, 1H), 7.26 – 7.22 (m, 1H), 6.92 – 6.91 (m, 2H), 6.81 – 6.78 (m, 1H), 4.22 (t, *J* = 7.7 Hz, 1H), 3.83 (s, 3H), 3.82 (s, 3H), 3.63 (s, 3H), 2.76 – 2.64 (m, 1H), 2.56 – 2.44 (m, 1H), 2.40 – 2.34 (m, 2H). **^{13}C NMR (75 MHz, CDCl$_3$):** δ 173.9, 163.2, 149.0, 147.8, 147.7, 136.3, 135.3, 129.3, 129.2, 127.4, 126.8, 125.9, 121.2, 120.0, 111.5, 111.2, 55.8, 55.8, 52.8, 51.4, 32.4, 39.8. **HRMS (ESI):** calc'd for $C_{22}H_{24}NO_4$ $[M+H]^+$: 366.1700, found: 366.1700.

Methyl 4-(quinolin-2-yl)-4-(4-trifluoromethyl)phenyl)butanoate (4s)

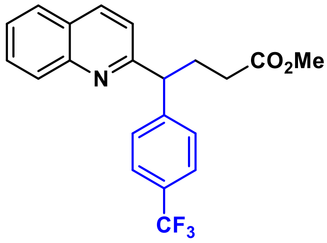

4s

Following **General Procedure A** with **1a** and 4-(trifluoromethyl)styrene. Purification by column chromatography (silica, gradient from 3% to 30% of EtOAc in cyclohexane) afforded 33.9 mg (91%) of an inseparable mixture of De Mayo product and Michael addition product (5.2:1) **4s** as a yellow oil. **R*f*** = 0.30 (5:1 cyclohexane:EtOAc). **^{1}H NMR (500 MHz, CDCl$_3$)** (De Mayo:Michael 5.2:1): δ 8.13 – 8.08 (m, 1H+2H$_{Michael}$), 8.02 (dd, *J* = 8.5, 0.8 Hz, 1H), 7.80 (dd, *J* = 8.0, 1.4 Hz, 1H$_{Michael}$), 7.75 (dd, *J* = 8.1, 1.4 Hz, 1H), 7.73 – 7.69 (m, 1H+1H$_{Michael}$), 7.57 – 7.47 (m, 5H+3H$_{Michael}$), 7.42 (d, *J* = 8.5 Hz, 1H$_{Michael}$) 7.28 (d, *J* = 8.0 Hz, 2H$_{Michael}$), 7.20 (d, *J* = 8.5 Hz, 1H), 4.35 (t, *J* = 7.7 Hz, 1H), 4.05 (t, *J* = 7.5 Hz, 1H$_{Michael}$), 3.69 (s, 3H$_{Michael}$), 3.63 (s, 3H), 2.81 – 2.64 (m, 1H+2H$_{Michael}$), 2.57 – 2.48 (m, 1H+1H$_{Michael}$), 2.43 – 2.31 (m, 2H+1H$_{Michael}$). **^{13}C NMR (125 MHz, CDCl$_3$):** δ 173.7, 173.1 (C$_{Michael}$), 161.7, 158.2 (C$_{Michael}$), 147.8, 146.9, 145.4 (C$_{Michael}$), 136.9 (C$_{Michael}$), 136.5, 129.6 (C$_{Michael}$), 129.5, 129.4, 129.3 (C$_{Michael}$), 129.1 (q, J_{C-F} = 32.5 Hz), 128.9 (C$_{Michael}$), 128.5 (2C), 127.5 (C$_{Michael}$), 127.5, 127.2 (C$_{Michael}$), 126.9, 126.5 (C$_{Michael}$), 126.2, 125.5 (q, J_{C-F} = 3.8 Hz, 2C), 125.3 (q, J_{C-F} = 3.6 Hz, C$_{Michael}$), 124.4 (q, J_{C-F} = 272.2 Hz) 121.2, 120.1 (C$_{Michael}$), 53.9 (C$_{Michael}$), 53.0, 52.2 (C$_{Michael}$), 51.5, 33.4 (C$_{Michael}$), 33.4 (C$_{Michael}$), 32.2, 29.8. It was

not possible to determine all the signals of the Michael addition due to the low amount of this product. **^{19}F NMR (282 MHz, $CDCl_3$):** δ -62.3 ($F_{Michael}$), -62.4. **HRMS (APCI):** calc'd for $C_{21}H_{19}F_3NO_2$ $[M+H]^+$: 374.1362, found: 374.1362.

Methyl 4-(2-bromophenyl)-4-(quinolin-2-yl)butanoate (4t)

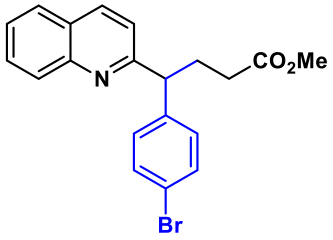

4t

Following **General Procedure A** with **1a** and 4-bromostyrene. Purification by column chromatography (silica, gradient from 3% to 30% of EtOAc in cyclohexane) afforded 25.3 mg (66%) of the title compound **4t** as a yellow oil. **R_f** = 0.23 (5:1 cyclohexane:EtOAc). **^{1}H NMR (300 MHz, $CDCl_3$):** δ 8.09 (d, *J* = 8.4 Hz, 1H), 8.00 (d, *J* = 8.5 Hz, 1H), 7.78 – 7.68 (m, 2H), 7.50 (t, *J* = 6.9 Hz, 1H), 7.42 – 7.39 (m, 2H), 7.27 – 7.24 (m, 2H), 7.18 (d, *J* = 8.7 Hz, 1H), 4.24 (t, *J* = 7.7 Hz, 1H), 3.63 (s, 3H), 2.78 – 2.66 (m, 1H), 2.54 – 2.42 (m, 1H), 2.39 – 2.30 (m, 2H). **^{13}C NMR (75 MHz, $CDCl_3$):** δ 173.8, 162.2, 147.8, 141.9, 136.4, 131.7 (2C),129.9 (2C), 129.4 (2C), 127.4, 126.9, 126.1, 121.2, 120.6, 52.6, 51.5, 32.2, 29.7. **HRMS (APCI):** calc'd for $C_{20}H_{19}BrNO_2$ $[M+H]^+$: 384.0594, found: 384.0593.

Methyl 4-(2-chlorophenyl)-4-(quinolin-2-yl)butanoate (4u)

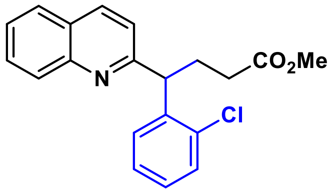

4u

Following **General Procedure A** with **1a** and 2-chlorostyrene. Purification by column chromatography (silica, 10:1 cyclohexane:EtOAc) afforded 26.4 mg (78%) of the title compound **4u** as a yellowish solid. **R_f** = 0.54 (5:1 cyclohexane:EtOAc). **^{1}H NMR (300 MHz, $CDCl_3$):** δ 8.12 (d, *J* = 8.4 Hz, 1H), 7.99 (d, *J* = 8.4 Hz, 1H), 7.77 – 7.67 (m, 2H), 7.49 (td, *J* = 8.0, 1.2 Hz, 1H), 7.39 (td, *J*= 8.4, 2.1 Hz, 2H), 7.27 – 7.10 (m, 3H), 4.90 (t, *J*= 7.5 Hz, 1H), 3.64 (s, 3H), 2.88 – 2.76 (m, 1H), 2.54 – 2.35 (m, 3H). **^{13}C NMR (75 MHz, $CDCl_3$):** δ 173.9, 161.7, 147.9, 140.5, 136.2, 134.0, 129.6, 129.5, 129.5, 129.2, 127.8, 127.4, 127.1, 126.9, 126.0, 122.1, 51.5, 48.2, 32.3, 29.4. **HRMS (ESI):** calc'd for $C_{20}H_{18}ClNO_2$ $[M+H]^+$: 340.1104, found: 340.1151.

Methyl 4-(quinolin-2-yl)-4-(*o*-tolyl)butanoate (4v)

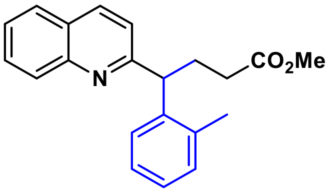

4v

Following **General Procedure A** with **1a** and 2-methylstyrene. Purification by column chromatography (silica, gradient from 10:1 to 2:1 of cyclohexane:EtOAc) afforded 31.5 mg (99%) of the title compound **4v** as a yellow solid. **R_f** = 0.38 (5:1 cyclohexane:EtOAc). **^{1}H NMR (300 MHz, $CDCl_3$):** δ 8.11 (d, *J* = 8.5 Hz, 1H), 7.97 (d, *J* = 8.5 Hz, 1H), 7.75 – 7.68 (m, 2H), 7.49 (t, *J* = 8.1 Hz, 1H), 7.37 – 7.34 (m, 1H), 7.19 – 7.11 (m, 4H), 4.56 (t, *J* = 8.1 Hz, 1H), 3.62 (s, 3H), 2.79 – 2.66 (m, 1H), 2.52 – 2.44 (m, 3H), 2.40 (s, 3H). **^{13}C NMR (75 MHz, $CDCl_3$):** δ 174.0, 163.0, 147.6, 140.9, 136.6, 136.2, 130.6, 129.3, 129.2, 127.4, 127.3, 126.8, 126.5, 126.3, 125.9, 121.2, 51.4, 48.8, 32.4, 29.6, 20.0. **HRMS (ESI):** calc'd for $C_{21}H_{22}NO_2$ $[M+H]^+$: 320.1645, found: 320.1641.

Methyl 4-phenyl-4-(quinolin-2-yl)pentanoate (4w)

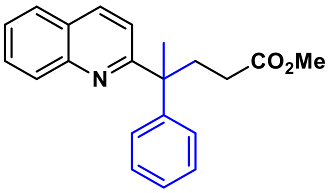

4w

Following **General Procedure A** with **1a** and α-methylstyrene. Purification by column chromatography (silica, gradient from 10:1 to 5:1 of cyclohexane:EtOAc) afforded 21.9 mg (70%) of the title compound **4w** as a yellowish oil. **R_f** = 0.56 (5:1 cyclohexane:EtOAc). **^{1}H NMR (300 MHz, $CDCl_3$):** δ 8.13 (d, *J* = 8.4 Hz, 1H), 7.92 (d, *J* = 8.7 Hz, 1H), 7.76 – 7.68 (m, 2H), 7.50 (td, *J* = 8.2, 1.2 Hz, 1H), 7.31 – 7.20 (m, 5H), 7.03 (d, *J* = 8.7 Hz, 1H), 3.61 (s, 3H), 2.80 – 2.63 (m, 2H), 2.38 – 2.12 (m, 2H), 1.78 (s, 3H). **^{13}C NMR (75 MHz, $CDCl_3$):** δ 174.5, 166.7, 147.4, 147.0, 135.7, 129.7, 129.1, 128.3 (2C), 127.3 (2C), 127.2, 126.5, 126.2, 126.1, 121.2, 51.5, 49.0, 35.7, 30.2, 25.9. **HRMS (ESI):** calc'd for $C_{21}H_{22}NO_2$ $[M+H]^+$: 320.1651, found: 320.1687.

(rac)-(3*R*,4*S*)-methyl 4-(4-methoxyphenyl)-3-methyl-4-(quinolin-2-yl)butanoate (4x)

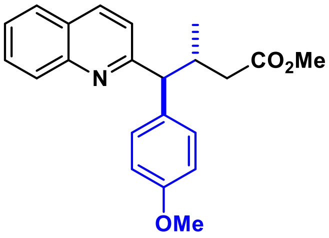

4x

Following **General Procedure A** with **1a** and *trans*-anethole. Purification by column chromatography (silica, gradient from 3% to 30% of EtOAc in cyclohexane) afforded 28.0 mg (80%) of the title compound **4x** as a yellow oil. **R*f*** = 0.38 (5:1 cyclohexane:EtOAc). **^{1}H NMR (300 MHz, $CDCl_3$):** δ 8.10 (d, *J* = 8.2 Hz, 1H), 7.99 (d, *J* = 8.5 Hz, 1H), 7.73 – 7.64 (m, 2H), 7.48 – 7.30 (m, 4H), 6.81 (d, *J* = 8.7 Hz, 2H), 3.96 (d, *J* = 10.7 Hz, 1H), 3.75 (s, 3H), 3.57 (s, 3H), 3.26 – 3.16 (m, 1H), 2.41 (dd, *J* = 15.2, 4.0 Hz, 1H), 2.21 (dd, *J* = 15.1, 9.1 Hz, 1H), 0.97 (d, *J* = 6.6 Hz, 3H). The relative stereochemistry between the aryl and methyl substituents was determined as *anti*. The coupling constant of 10.7 Hz between the hydrogen at 3.96 ppm and the hydrogen at 3.26 – 3.16 ppm can only be assumed if both hydrogens are in an *anti* disposition.[1] **^{13}C NMR (75 MHz, $CDCl_3$):** δ 173.5, 163.0, 158.3, 147.8, 136.3, 134.1, 129.6 (2C), 129.4, 129.2, 127.4, 126.8, 125.9, 121.5, 113.9 (2C), 59.7, 55.1, 51.3, 39.9, 34.0, 18.5. **HRMS (APCI):** calc'd for $C_{22}H_{24}NO_3$ $[M+H]^+$: 350.1751, found: 350.1749.

(rac)-(3*R*,4*S*)-methyl 3-methyl-4-phenyl-4-(quinolin-2-yl)butanoate (4y)

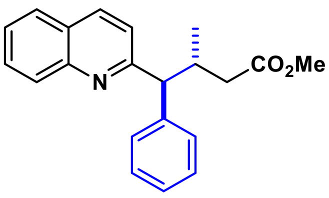

4y

Following **General Procedure B** with **1a** and *trans*-β-methylstyrene. Purification by column chromatography (silica, gradient from 3% to 30% of EtOAc in cyclohexane) afforded 10.0 mg (32%) of the title compound **4y** as a colorless oil. **R*f*** = 0.58 (5:1 cyclohexane:EtOAc). **^{1}H NMR (300 MHz, $CDCl_3$):** δ 8.09 (d, *J* = 8.4 Hz, 1H), 7.99 (d, *J* = 8.4 Hz, 1H), 7.74 – 7.65 (m, 2H), 7.49 – 7.45 (m, 3H), 7.35 – 7.24 (m, 3H), 7.20 – 7.14 (m, 1H), 4.01 (d, *J* = 10.8 Hz, 1H), 3.57 (s, 3H), 3.32 – 3.18 (m, 1H), 2.40 (dd, *J* = 15.0, 3.6 Hz, 1H), 2.20 (dd, *J* = 15.3, 9.3 Hz, 1H), 0.96 (d, *J* = 6.6 Hz, 3H). The relative stereochemistry between the aryl and methyl substituents was determined as *anti*. The coupling constant of 10.8 Hz between the hydrogen at 4.01 ppm and the hydrogen at 3.32 – 3.18 ppm can only be assumed if both hydrogens are in an *anti* disposition.[1] **^{13}C NMR (75 MHz, $CDCl_3$):** δ 173.5, 162.7, 147.9, 142.0, 136.3, 129.4, 129.2, 128.7 (2C), 128.5 (2C), 127.4, 126.8, 126.6, 125.9, 121.6, 60.6, 51.3, 39.9, 33.9, 18.5. **HRMS (APCI):** calc'd for $C_{21}H_{22}NO_2$ $[M+H]^+$: 320.1645, found: 320.1643.

Methyl 4,5-dimethyl-4-(quinolin-2-yl)hex-5-enoate (4z)

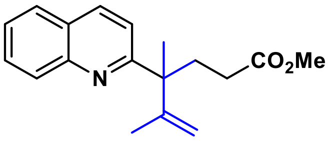

4z

Following **General Procedure A** with **1a** and 2,3-dimethyl-1,3-butadiene. Purification by column chromatography (silica, 5:1 cyclohexane:EtOAc) afforded 19.6 mg (70%) of the title compound **4z** as a yellow oil. **R*f*** = 0.59 (5:1 cyclohexane:EtOAc). **^{1}H NMR (300 MHz, $CDCl_3$):** δ 8.08 (d, *J* = 8.4 Hz, 1H), 8.02 (d, *J* = 8.7 Hz, 1H), 7.76 (dd, *J* = 8.0, 1.5 Hz, 1H), 7.68 (td, *J* = 8.5, 1.5 Hz, 1H), 7.49 (td, *J* = 8.1, 1.4 Hz, 1H), 7.37 (dd, *J* = 8.7, 1.4 Hz, 1H), 5.07 (s, 2H), 3.65 (s, 3H), 2.62 – 2.11 (m, 4H), 1.53 (s, 3H), 1.49 (s, 3H). **^{13}C NMR (75 MHz, $CDCl_3$):** δ 174.7, 165.5, 149.7, 147.3, 135.8, 129.6, 129.0, 127.2, 126.6, 125.9, 119.8, 112.3, 51.5, 50.1, 32.1, 29.9, 23.8, 20.3. **HRMS (ESI):** calc'd for $C_{18}H_{22}NO_2$ $[M+H]^+$: 284.1650, found: 284.1611.

Methyl 4-(pyridin-2-yl)-4-(quinolin-2-yl)butanoate (4aa)

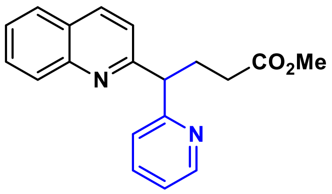

4aa

Following **General Procedure A** with **1a** and 2-vinylpyridine. Purification by column chromatography (silica, 2:1 cyclohexane:EtOAc) afforded 10.6 mg (35%) of the title compound **4aa** as a yellow oil. **R*f*** = 0.10 (5:1 cyclohexane:EtOAc). **^{1}H NMR (300 MHz, $CDCl_3$):** δ 8.52 (d, *J* = 4.2 Hz, 1H), 8.15 – 8.02 (m, 2H), 7.79 (dd, *J* = 8.1, 1.4 Hz, 1H), 7.70 (t, *J* = 8.1 Hz, 1H), 7.59 – 7.45 (m, 3H), 7.15 – 7.06 (m, 2H), 4.10 (t, *J* = 7.5 Hz, 1H), 3.69 (s, 3H), 2.92 – 2.62 (m, 3H), 2.57 – 2.46 (m, 1H). **^{13}C NMR (75 MHz, $CDCl_3$):** δ 173.2, 161.0, 158.6, 149.3, 147.8, 136.8, 136.3, 129.5, 129.4, 127.4, 127.2, 126.4, 123.0, 121.1, 120.3, 54.2, 52.1, 36.0, 31.9. **HRMS (ESI):** calc'd for $C_{19}H_{18}N_2O_2$ $[M+H]^+$: 307.1447, found: 307.1441.

Preparation of 2-vinylthiophene **SI1**:

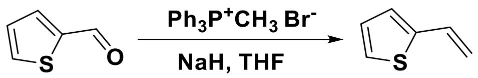

SI1

To a solution of methyltriphenylphosphonium bromide (9.6 g, 27 mmol, 1.5 equiv.) in dry THF (90 mL) under N_2 atmosphere at 0 ºC, NaH (60% in mineral oil, 1.1 g, 27 mmol, 1.5 equiv.) was added and the solution was stirred at this temperature for 1 h. Then, a solution of thiophene-2-carbaldehyde (1.7 mL, 18 mmol, 1.0 equiv.) in dry THF (4.0 mL) was added dropwise and the reaction was stirred at r.t. for 24 h. H_2O (100 mL) was added and the mixture was extracted with Et_2O (3 x 150 mL). The combined organic layers were dried over $MgSO_4$, filtered and concentrated under reduced pressure (200 mbar, 30 ºC). Purification by flash column chromatography (Iatrobeads, 8:1 pentane:Et_2O) afforded 254 mg (25%) of 2-vinylthiophene **SI1** in a 0.7 M solution of THF as a colorless liquid. **R*f*** = 0.90 (8:1 pentane:Et_2O). **^{1}H NMR (300 MHz,**

$CDCl_3$): δ 6.98 (d, *J* = 4.2 Hz, 1H), 6.80 – 6.78 (m, 2H), 6.65 (dd, *J* = 17.4, 10.8 Hz, 1H), 5.42 (d, *J* = 17.4 Hz, 1H), 4.97 (d, *J* = 10.8 Hz, 1H).

Spectral data is in accordance with previous reports.[2]

Methyl 4-(quinolin-2-yl)-4-(thiophen-2-yl)butanoate (4ab)

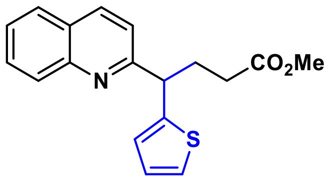

4ab

Following **General Procedure A** with **1a** and 2-vinylthiophene. Purification by column chromatography (silica, gradient from 10:1 to 5:1 of cyclohexane:EtOAc) afforded 32.9 mg (99%) of the title compound **4ab** as a yellow oil. **R*f*** = 0.44 (5:1 cyclohexane:EtOAc). **^{1}H NMR (300 MHz, $CDCl_3$):** δ 8.07 (d, *J* = 8.4 Hz, 1H), 7.77 – 7.67 (m, 2H), 7.52 – 7.43 (m, 1H), 7.33 (d, *J* = 8.4 Hz, 1H), 7.17 (d, *J* = 5.1 Hz, 1H) 7.02 – 6.90 (m, 2H), 4.58 (t, *J* = 7.6 Hz, 1H), 3.63 (s, 3H), 2.78 – 2.26 (m, 4H). In the spectrum is possible to see the signal to the corresponding enol: 6.07 (s, 1H). **^{13}C NMR (75 MHz, $CDCl_3$):** δ 173.6, 162.3, 147.7, 146.1, 136.7, 129.4, 129.3, 127.4, 127.0, 126.6, 126.2, 124.8, 124.4, 120.5, 51.5, 48.9, 32.2, 31.3. **HRMS (ESI):** calc'd for $C_{18}H_{17}NO_2S$ $[M+H]^+$: 312.1058, found: 312.1091.

Preparation of 1,2-dimethyl-3-vinyl-1*H*-indole **SI2**:

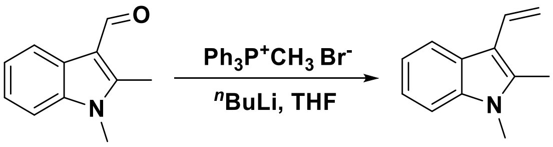

SI2

To a solution of methyltriphenylphosphonium bromide (1.0 g, 2.9 mmol, 1.1 equiv.) in dry THF (8.0 mL) under N_2 atmosphere at 0 ºC, ^{n}BuLi (2.5 M in hexanes, 1.2 mL, 3.0 mmol, 1.2 equiv.) was added dropwise and the solution was stirred at this temperature for 1 h. Then, a solution of 1,2-dimethylindole-3-carbaldehyde (0.43 g, 2.5 mmol, 1.0 equiv.) in dry THF (8.0 mL) was added dropwise and the reaction was stirred at r.t. for 1 h. Then, H_2O (20 mL) was added and the mixture was extracted with EtOAc (3 x 40 mL). The combined organic layers were dried over $MgSO_4$, filtered and concentrated under reduced pressure. Purification by flash column chromatography (Iatrobeads, gradient from 3% to 30% of EtOAc in cyclohexane) afforded 31.5 mg (10%) of 1,2-dimethyl-3-vinyl-1*H*-indole **SI2** as a colorless oil. **R*f*** = 0.74 (1:1 cyclohexane:EtOAc). **^{1}H NMR (300 MHz, $CDCl_3$):** δ 7.84 (dd, *J* = 8.2, 1.3 Hz, 1H), 7.25 – 7.11 (m, 3H), 6.89 (dd, *J* = 18.0, 11.7 Hz, 1H), 5.64 (dd, *J* = 17.7, 1.3 Hz, 1H), 5.17 (dd, *J* = 11.4, 1.5 Hz, 1H), 3.60 (s, 3H), 3.40 (s, 3H).

Spectral data are in accordance with previous reports.[3]

Methyl 4-(1,2-dimethyl-1H-indol-3-yl)-4-(quinolin-2-yl)butanoate (4ac)

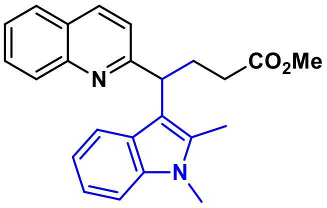

4ac

Following **General Procedure A** with **1a** and 1,2-dimethyl-3-vinyl-1*H*-indole. Purification by column chromatography (silica, gradient from 3% to 15% of EtOAc in cyclohexane) afforded 22.1 mg (60%) of the title compound **4ac** as a yellow oil. **R*f*** = 0.35 (5:1 cyclohexane:EtOAc). **^{1}H NMR (300 MHz, $CDCl_3$):** δ 8.13 (d, *J* = 8.1 Hz, 1H), 7.86 (d, *J* = 8.5, 1H), 7.71 – 7.65 (m, 2H), 7.55 (d, *J* = 7.9 Hz, 1H), 7.46 (td, *J* = 8.1, 1.2 Hz, 1H), 7.25 – 7.17 (m, 2H), 7.10 (td, *J* = 8.2, 1.2 Hz, 1H), 6.95 (td, *J* = 8.0, 1.1 Hz, 1H), 4.56 (dd, *J* = 9.3, 6.2 Hz, 1H), 3.65 (s, 3H), 3.59 (s, 3H), 3.08 – 2.95 (m, 1H), 2.77 – 2.62 (m, 1H), 2.45 – 2.40 (m, 5H). **^{13}C NMR (75 MHz, $CDCl_3$):** δ 174.4, 163.7, 147.4, 136.8, 135.7, 134.1, 129.4, 129.0, 127.3, 126.9, 126.7, 125.7, 121.9, 120.5, 119.3, 118.9, 111.6, 108.6, 51.3, 44.3, 32.7, 29.6, 27.9, 10.6. **HRMS (APCI):** calc'd for $C_{24}H_{25}N_2O_2$ $[M+H]^+$: 373.1911, found: 373.1908.

Methyl 4-((8*R*,9*S*,13*S*,14*S*)-13-methyl-17-oxo-7,8,9,11,12,13,14,15,16,17-decahydro-6*H*-cyclopenta[a]phenanthren-3-yl)-4-(quinolin-2-yl)butanoate (5a)

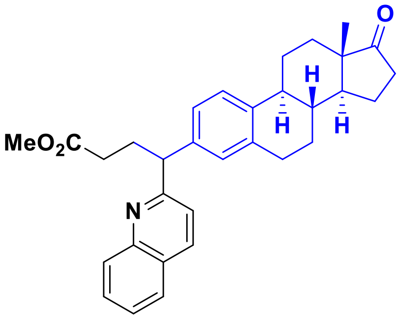

5a

Following **General Procedure A** with **1a** and vinyl-estrone. Purification by column chromatography (silica, gradient from 12:1 to 5:1 of cyclohexane:EtOAc) afforded 41.4 mg (86%) of the title compound **5a** in a 50:50 diastereomeric mixture as a colorless oil. **R*f*** = 0.19 (5:1 cyclohexane:EtOAc). **^{1}H NMR (300 MHz, $CDCl_3$):** δ 8.10 (d, *J* = 7.5 Hz, 1H), 8.00 (d, *J* = 8.5 Hz, 1H), 7.77 – 7.64 (m, 2H), 7.51 – 7.46 (m, 1H), 7.27 – 7.13 (m, 3H), 7.08 (s, 1H), 4.22 (t, *J* = 7.6 Hz, 1H), 3.63 (s, 3H), 2.86 (dd, *J* = 8.6, 3.9 Hz, 2H), 2.78 – 2.63 (m, 1H), 2.55 – 1.92 (m, 10H), 1.63 – 1.38 (m, 6H), 0.88 (d, *J* = 0.9 Hz, 3H). **^{13}C NMR (75 MHz, $CDCl_3$):** δ 220.8, 174.0, 163.1, 147.7, 140.2, 138.1, 136.6, 136.3, 129.3, 129.2, 128.7, 128.7, 127.4, 126.9, 125.9, 125.5, 125.4, 121.2, 52.9, 51.4, 50.5, 47.9, 44.3, 38.1, 35.8, 32.5, 31.6, 29.7, 29.4, 26.5, 25.6, 21.5, 13.8. **HRMS (APCI):** calc'd for $C_{32}H_{36}NO_3$ $[M+H]^+$: 482.2690, found: 482.2692.

Methyl 4-(4-((*S*)-2-((tert-butoxycarbonyl)amino)-3-methoxy-3-oxopropyl)phenyl)-4-(quinolin-2-yl)butanoate (5b)

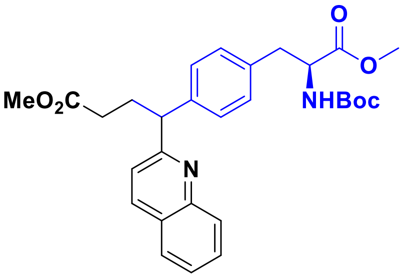

5b

Following **General Procedure A** with **1a** and vinyl-Boc-Tyr-OMe. Purification by column chromatography (silica, gradient from 3% to 30% of EtOAc in cyclohexane) afforded 46.3 mg (89%) of the title compound **5b** in a 50:50 diastereomeric mixture as a yellow oil. **R*f*** = 0.29 (2:1 cyclohexane:EtOAc). **^{1}H NMR (300 MHz, $CDCl_3$):** δ 8.09 (d, *J* = 8.5 Hz, 1H), 7.99 (d, *J* = 8.5 Hz, 1H), 7.76 – 7.63 (m, 2H), 7.52 – 7.45 (m, 1H), 7.29 (d, *J* = 7.9 Hz, 2H), 7.21 (d, *J* = 8.5 Hz, 1H), 7.05 (d, *J* = 8.1 Hz, 2H), 4.94 (d, *J* = 8.3 Hz, 1H), 4.65 – 4.51 (m, 1H), 4.25 (t, *J* = 7.7 Hz, 1H), 3.67 (s, 3H), 3.62 (s, 3H), 3.09 – 2.93 (m, 2H), 2.80 – 2.63 (m, 1H), 2.57 – 2.42 (m, 1H), 2.41 – 2.28 (m, 2H), 1.37 (s, 9H). **^{13}C NMR (75 MHz, $CDCl_3$):** δ 173.9 (2C), 172.3, 162.8, 154.9, 147.7, 141.6, 136.3, 134.4, 129.5 (2C), 129.4, 129.2, 128.3, 127.4, 126.9, 126.0, 121.53, 79.8, 54.3, 52.9, 52.1, 51.4, 38.0, 32.4, 29.7, 28.2. **HRMS (APCI):** calc'd for $C_{29}H_{35}N_2O_6$ $[M+H]^+$: 507.2490, found: 507.2485. The diastereomeric ratio was determined for compound **5b** using a Chiralpak IA-3 column: CO_2/MeOH gradient from 95:5 to 60:40, flow rate 2 mL/min, τ_1 = 5.9 min, τ_2 = 6.0 min, *dr* = 50:50. The *dr* of compounds **5a** and **5c** was assumed to be the same by analogy.

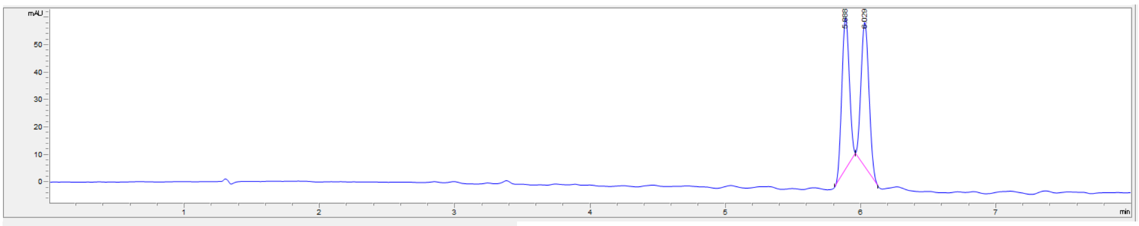

#	Time	Type	Area	Height	Width	Area%	Symmetry
1	5.888	BB	211.2	55.1	0.0607	49.682	0.994
2	6.029	BB	213.9	52.7	0.0634	50.318	0.833

Methyl 4-((*R*)-2,8-dimethyl-2-((4*R*,8*R*)-4,8,12-trimethyltridecyl)chroman-6-yl)-4-(quinolin-2-yl)butanoate (5c)

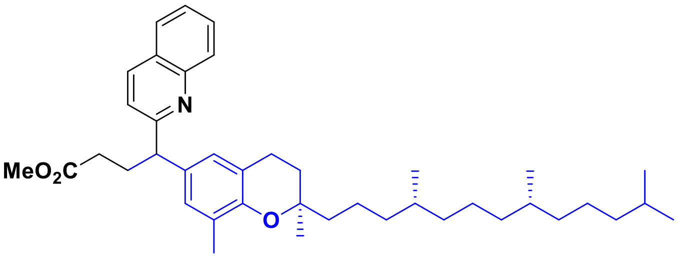

5c

Following **General Procedure A** with **1a** and vinyl-δ-tocopherol. Purification by column chromatography (silica, gradient from 3% to 15% of EtOAc in cyclohexane) afforded 58.9 mg (92%) of the title compound **5c** in a 50:50 diastereomeric mixture as a yellow oil. **R*f*** = 0.50 (5:1 cyclohexane:EtOAc). **^{1}H NMR (300 MHz, $CDCl_3$):** δ 8.10 (d, *J* = 8.4 Hz, 1H), 7.97 (d, *J* = 8.6 Hz, 1H), 7.75 – 7.63 (m, 2H), 7.46 (t, *J* = 7.5 Hz, 1H), 7.27 – 7.20 (m, 1H), 6.92 (s, 1H), 6.85 (s, 1H), 4.12 (t, *J* = 7.6 Hz, 1H), 3.62 (s, 3H), 2.73 – 2.59 (m, 3H), 2.52 – 2.30 (m, 3H), 2.10 (s, 3H), 1.84 – 1.61 (m, 2H), 1.59 – 0.96 (m, 24H), 0.87 – 0.82 (m, 12H). **^{13}C NMR (75 MHz, $CDCl_3$):** δ 174.1, 163.8, 150.9, 147.7, 136.1, 132.7, 129.4, 129.1, 128.0, 127.4, 126.9, 126.4, 126.2, 125.8, 121.3, 120.4, 75.9, 52.7, 51.4, 40.3, 40.3, 39.4, 37.4, 37.4, 37.3, 32.8, 32.7, 32.6, 31.2, 29.8, 28.0, 24.8, 24.4, 24.2, 24.2, 22.7, 22.6, 22.4, 21.0, 19.7, 19.6, 16.1. **HRMS (APCI):** calc'd for $C_{41}H_{60}NO_3$ $[M+H]^+$: 614.4568, found: 614.4566.

Supplementary Notes 3

Synthesis and characterization of azaarenes (1)

- **General procedure D1 for the synthesis of azaaryl acetates 1a, 1e, 1f, 1l.**

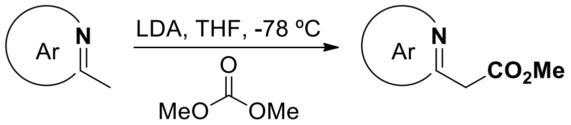

Following the reported procedure,[4] to a solution of diisopropyl amine (3.0 equiv.) in dry THF (1.0 M) under N_2 atmosphere at 0 ºC, was added *ⁿ*BuLi (2.5 M in hexanes, 3.0 equiv.) and the solution was stirred at this temperature for 15 min. A solution of the corresponding methyl azaarene (1.0 equiv.) in dry THF (0.5 M) was added dropwise to the LDA solution at -78ºC. The reaction mixture was stirred at this temperature for 2 h. After this time, dimethyl carbonate (1.2 equiv.) was added to the reaction mixture quickly. After stirring at -78 ºC for 15 min, the reaction mixture was quenched by water and warmed to r.t. The mixture was diluted by water (50 mL) and extracted with EtOAc (100 mL). The organic layer was dried over Na_2SO_4, filtered, concentrated under reduced pressure, and purified by flash column chromatography to provide the pure esters.

- **General procedure D2 for the synthesis of azaaryl acetates 1b, 1c, 1d, 1m, 1n.**

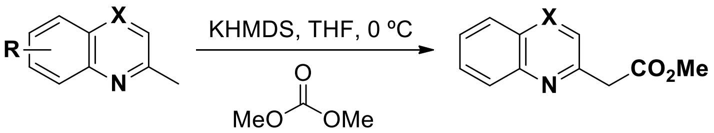

To a solution of the corresponding methyl azaarene (1.0 equiv.) in dry THF (0.3 M) under N_2 atmosphere at 0 ºC, KHMDS (1.0 M in THF, 1.2 equiv.) was added dropwise. The reaction was stirred at this temperature for 30 min. Then, dimethyl carbonate (1.3 equiv.) was added quickly. After stirring at 0 ºC for 15 min, the reaction mixture was quenched by water and warmed to r.t. The mixture was diluted by water (50 mL) and extracted with EtOAc (100 mL). The combined

organic layers were dried over Na_2SO_4, filtered, concentrated under reduced pressure, and purified by flash column chromatography to provide the pure esters.

- **General procedure D3 for the synthesis of azaaryl ketones 1j, 1o.**

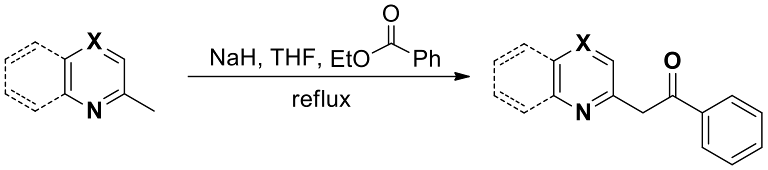

To a solution of the corresponding methyl azaarene (1.0 equiv.) in dry THF (0.2 M), ethyl benzoate (1.3 equiv.) and NaH (60% dispersion in mineral oil, 3.0 equiv.) were added under N_2 atmosphere. After stirring under reflux for 12 h, the reaction mixture was quenched with water (20 mL) and extracted with Et_2O (2 x 50 mL). The combined organic layers were dried over Na_2SO_4, filtered, concentrated under reduced pressure, and purified by flash column chromatography to provide the pure ketones.

Methyl 2-(quinolin-2-yl)acetate (1a)

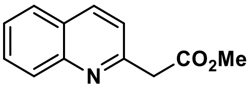

1a

Following **General Procedure D1** on a 10.0 mmol scale with 2-methylquinoline. Purification by column chromatography (silica, 5:1 cyclohexane:EtOAc) afforded 1.43 g (86%) of the title compound **1a** as a yellow solid. **R*f*** = 0.33 (2:1 cyclohexane:EtOAc). **^{1}H NMR (300 MHz, $CDCl_3$):** δ 8.13 (d, *J* = 8.5 Hz, 1H), 8.06 (d, *J* = 8.5 Hz, 1H), 7.80 (d, *J* = 8.0 Hz, 1H), 7.70 (td, *J* = 8.4, 1.5 Hz, 1H), 7.52 (td, *J* = 8.2, 1.2 Hz, 1H), 7.43 (d, *J* = 8.4 Hz, 1H), 4.06 (s, 2H), 3.73 (s, 3H). Spectral data is in accordance with previous reports.[4]

Methyl 2-(7-chloroquinolin-2-yl)acetate (1b)

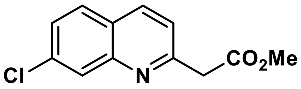

1b

Following **General Procedure D2** on a 5.0 mmol scale with 7-chloro-2-methylquinoline. Purification by column chromatography (silica, gradient from 8:1 to 5:1 of cyclohexane:EtOAc) afforded 201 mg (17%) of the title compound **1b** as a yellow solid. **R*f*** = 0.53 (2:1 cyclohexane:EtOAc). **^{1}H NMR (300 MHz, $CDCl_3$):** δ 8.11 – 8.06 (m, 2H), 7.73 (d, *J* = 8.7 Hz, 1H), 7.49 – 7.41 (m, 2H), 4.03 (s, 2H), 3.74 (s, 3H). **^{13}C NMR (75 MHz, $CDCl_3$):** δ 170.7, 155.9, 148.2, 136.4, 135.5, 128.7, 128.2, 127.5, 125.4, 121.9, 52.2, 44.5. **HRMS (ESI):** calc'd for $C_{12}H_{11}ClNO_2$ $[M+H]^+$: 236.0478, found: 236.0480.

Methyl 2-(6-chloroquinolin-2-yl)acetate (1c)

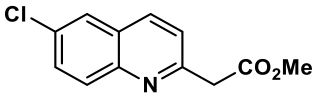

1c

Following **General Procedure D2** on a 2.8 mmol scale with 6-chloro-2-methylquinoline. Purification by column chromatography (silica, gradient from 3% to 30% of EtOAc in cyclohexane) afforded 125 mg (19%) of the title compound **1c** as a yellow solid. **R*f*** = 0.14 (5:1 cyclohexane:EtOAc). **^{1}H NMR (300 MHz, $CDCl_3$):** δ 8.02 – 7.96 (m, 2H), 7.72 (d, *J* = 2.3 Hz, 1H), 7.58 (dd, *J* = 9.0, 2.4 Hz, 1H), 7.40 (d, *J* = 8.4 Hz, 1H), 4.00 (s, 2H), 3.71 (s, 3H). **^{13}C NMR (75 MHz, $CDCl_3$):** δ 170.6, 154.9, 146.1, 135.5, 132.0, 130.6, 130.4, 127.5, 126.0, 122.5, 52.1, 44.4. **HRMS (APCI):** calc'd for $C_{12}H_{11}ClNO_2$ $[M+H]^+$: 236.0473, found: 236.0462.

Methyl 2-(6-fluoroquinolin-2-yl)acetate (1d)

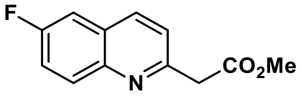

1d

Following **General Procedure D2** on a 3.1 mmol scale with 6-fluoro-2-methylquinoline. Purification by column chromatography (silica, gradient from 3% to 30% of EtOAc in cyclohexane) afforded 200 mg (30%) of the title compound **1d** as a yellow solid. **R*f*** = 0.14 (3:1 cyclohexane:EtOAc). **^{1}H NMR (300 MHz, $CDCl_3$):** δ 8.10 – 8.03 (m, 2H), 7.51 – 7.40 (m, 3H), 4.04 (s, 2H), 3.74 (s, 3H). **^{13}C NMR (75 MHz, $CDCl_3$):** δ 170.7, 160.2 (d, *J* = 246.0 Hz), 154.0 (d, *J* = 3.0 Hz), 144.9, 135.9 (d, *J* = 5.3 Hz), 131.6 (d, *J* = 9.0 Hz), 127.6 (d, *J* = 9.8 Hz), 122.4, 119.6 (d, *J* = 25.5 Hz), 110.4 (d, *J* = 21.8 Hz), 52.1, 44.3. **^{19}F NMR (282 MHz, $CDCl_3$):** δ -113.6. **HRMS (APCI):** calc'd for $C_{12}H_{11}FNO_2$ $[M+H]^+$: 220.0768, found: 220.0771.

2-(2-Methoxy-2-oxoethyl)quinoline-6-carboxylic acid (1e)

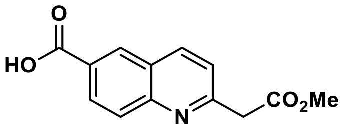

1e

Following **General Procedure D1** on a 1.0 mmol scale with 2-methylquinoline-6-carboxylic acid. Purification by column chromatography (silica, gradient from 20:1 to 5:1 of DCM:MeOH) afforded 24.5 mg (10%) of the title compound **1e** as a yellow solid. **R*f*** = 0.63 (10:1 DCM:MeOH). **^{1}H NMR (300 MHz, $CDCl_3$):** δ 8.67 (s, 1H), 8.35 (d, *J* = 8.4 Hz, 1H), 8.26 (d, *J* = 7.8 Hz, 1H), 8.14 (d, *J* = 8.7 Hz, 1H), 8.53 (d, *J* = 8.4 Hz, 1H), 4.11 (s, 2H), 3.76 (s, 3H). **^{13}C NMR (125 MHz, $(CD_3)_2SO$):** δ 170.6 (2C), 157.3, 148.7, 137.9 (2C), 130.3, 129.4, 128.6, 126.0, 123.1, 52.0, 43.8. **HRMS (ESI):** calc'd for $C_{13}H_{12}NO_4$ $[M+H]^+$: 246.0766, found: 246.0764.

Methyl 2-(6-methoxyquinolin-2-yl)acetate (1f)

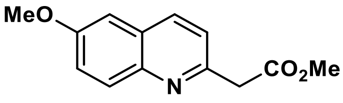

1f

Following **General Procedure D1** on a 2.7 mmol scale with 6-methoxy-2-methylquinoline. Purification by column chromatography (silica, 10:1 cyclohexane:EtOAc) afforded 283 mg (45%) of the title compound **1f** as an orange solid. **R*f*** = 0.35 (2:1 cyclohexane:EtOAc). **^{1}H NMR (300 MHz, $CDCl_3$):** δ 8.02 (d, *J* = 8.5 Hz, 1H), 7.95 (d, *J* = 9.2 Hz, 1H), 7.39 – 7.33 (m, 2H), 7.06 (d, *J* = 2.8 Hz, 1H), 4.01 (s, 2H), 3.92 (s, 3H), 3.73 (s, 3H). **^{13}C NMR (75 MHz, $CDCl_3$):** δ 171.1, 157.7, 152.1, 144.0, 135.4, 130.5, 128.0, 122.2, 121.9, 105.1, 55.5, 52.1, 44.4. **HRMS (ESI):** calc'd for $C_{13}H_{14}NO_3$ $[M+H]^+$: 232.0968, found: 232.0967.

***N,N*-Diphenyl-2-(quinolin-2-yl)acetamide (1g)**

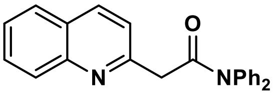

1g

To a solution of 2-methylquinoline (0.67 mL, 5.0 mmol, 1.0 equiv.) in dry THF (10 mL) under N_2 atmosphere at 0 ºC, ^{n}BuLi (1.6 M in hexanes, 3.8 mL, 6.0 mmol, 1.2 equiv.) was added dropwise. The reaction mixture was stirred at r.t. for 1 h. Then, the solution was cooled to 0 ºC and a solution of diphenylcarbamoyl chloride (1.3 g, 5.5 mmol, 1.1 equiv.) in dry THF (12 mL) was added dropwise. After stirring overnight at r.t., the reaction mixture was quenched with water (20 mL) and extracted with EtOAc (2 x 50 mL). The combined organic layers were dried over Na_2SO_4, filtered, concentrated under reduced pressure, and purified by flash column chromatography (silica gel, gradient from 9:1 to 1:1 of cyclohexane:EtOAc) to afford 580 mg (34%) of the title compound **1g** as an orange solid. **R*f*** = 0.18 (2:1 cyclohexane:EtOAc). **^{1}H NMR (300 MHz, $CDCl_3$)**: δ 8.08 (d, *J* = 8.4 Hz, 1H), 7.90 (d, *J* = 8.1 Hz, 1H), 7.77 (d, *J* = 8.1 Hz, 1H), 7.65 (t, *J* = 7.2 Hz, 1H), 7.52 (d, *J* = 8.4 Hz, 2H), 7.36 – 7.13 (m, 20H), 6.75 (d, *J* = 7.8 Hz, 4H), 5.40 (s, 1H). In the spectrum is possible to see the signal of the corresponding ketone: 4.07 (s, 1H) and the two rotamers. **^{13}C NMR (75 MHz, $CDCl_3$):** δ 167.8, 154.8, 147.5, 142.7, 142.0, 135.9, 129.6 (4C), 129.4, 129.0, 128.8 (2C), 128.1, 127.4, 127.1, 126.3 (4C), 122.1, 60.7. **HRMS (APCI):** calc'd for $C_{23}H_{19}N_2O$ $[M+H]^+$: 339.1492, found: 339.1478.

2-(Quinolin-2-yl)acetonitrile (1h)

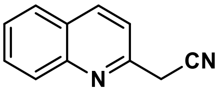

1h

2-(Bromomethyl)quinoline (400 mg, 1.8 mmol, 1.0 equiv.) was dissolved in a mixture of DCM/H_2O (3.6 mL: 0.9 mL). TBAB (145 mg, 0.4 mmol, 0.25 equiv.) and sodium cyanide (352 mg, 7.2 mol,

4.0 equiv.) was added. The mixture was heated to 40°C and stirred overnight. The residue was extracted with EtOAc (3 x 20 mL). The combined organic layers were dried over sodium sulfate and concentrated. The residue was purified by column chromatography (silica, gradient from 4:1 to 3:1 of cyclohexane:EtOAc) to afford 197 mg (65%) of the title compound **1h** as a brown solid. **R*f*** = 0.44 (2:1 cyclohexane:EtOAc). **^{1}H NMR (300 MHz, $CDCl_3$):** δ 8.22 (d, *J* = 8.4 Hz, 1H), 8.06 (d, *J* = 8.5 Hz, 1H), 7.84 (d, *J* = 8.1 Hz, 1H), 7.75 (t, *J* = 7.4 Hz, 1H), 7.60 – 7.75 (m, 2H), 4.12 (s, 2H). **^{13}C NMR (76 MHz, $CDCl_3$):** δ 150.5, 147.9, 137.7, 130.3, 129.1, 127.6, 127.2, 127.1, 119.5, 166.9, 27.4. **HRMS (APCI):** calc'd for $C_{11}H_9N_2$ $[M+H]^+$: 169.0760, found: 169.0763.

2-((Phenylsulfonyl)methyl)quinoline (1i)

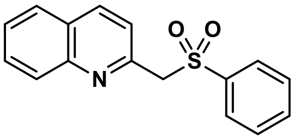

1i

Following a reported procedure,[5] to a solution of sodium benzenesulfinate (1.0 g, 5.2 mmol, 2.5 equiv.), KI (348 mg, 2.1 mmol, 1.0 equiv.), 2-methylquinoline (0.28 mL, 2.1 mmol, 1.0 equiv.), acetic acid (3.3 mL) and DMSO (3.3 mL) under air, TBHP (0.29 mL, 2.1 mmol, 1.0 equiv.) was slowly added. The reaction mixture was stirred at 80 ºC for 16 h. $NaHCO_3$ (20 mL) was added and the reaction was extracted with DCM (3 x 50 mL). The combined organic layers were dried over sodium sulfate and concentrated. The residue was purified by column chromatography (silica, 2:1 cyclohexane:EtOAc) to afford 385 mg (65%) of the title compound **1i** as a yellow solid. **R*f*** = 0.32 (2:1 cyclohexane:EtOAc). **^{1}H NMR (300 MHz, $CDCl_3$):** δ 8.18 (d, *J* = 8.5 Hz, 1H), 7.83 (dd, *J* = 8.4, 1.2 Hz, 2H), 7.70 – 7.53 (m, 6H), 7.41 (td, *J* = 7.9, 1.7 Hz, 2H), 4.74 (s, 2H). Spectral data is in accordance with previous reports.[5]

1-Phenyl-2-(quinolin-2-yl)ethenone (1j)

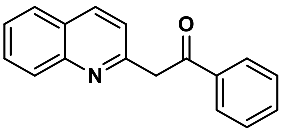

1j

Following **General Procedure D3** on a 5.0 mmol scale with 2-methylquinoline. Purification by column chromatography (silica, 5:1 cyclohexane:EtOAc) afforded 346 mg (27%) of the title compound **1j** as an orange solid. **R*f*** = 0.66 (2:1 cyclohexane:EtOAc). **^{1}H NMR (300 MHz, $CDCl_3$)**: δ 15.71 (s, 1H), 8.01 – 7.90 (m, 2H), 7.67 (d, *J* = 9.1 Hz, 1H), 7.58 – 7.42 (m, 6H), 7.29 – 7.23 (m, 1H), 6.88 (d, *J* = 9.1 Hz, 1H), 6.09 (s, 1H). In the spectrum is possible to see the signal corresponding to the ketone: 4.71 (s, 1H). Spectral data is in accordance with previous reports.[6]

Methyl 2-(quinolin-4-yl)acetate (1k)

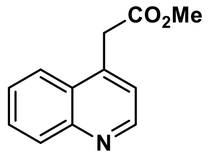

1k

Following a reported procedure,[7] to a solution of 4-methylquinoline (0.66 mL, 5.0 mmol, 1.0 equiv.) and dimethyl carbonate (0.55 mL, 6.5 mmol, 1.3 equiv.) in dry THF (6 mL) under N_2 atmosphere at 0 ºC, LiHMDS (1.0 M in THF, 20 mL, 20 mmol, 4.0 equiv.) was added dropwise. After stirring at 0 ºC for 3 h, the reaction mixture was quenched with a saturated solution of NH_4Cl (5 mL) and extracted with Et_2O (2 x 50 mL). The combined organic layers were dried over Na_2SO_4, filtered, concentrated under reduced pressure, and purified by flash column chromatography (silica gel, gradient from 5:1 to 2:1 cyclohexane:EtOAc) to afford 553 mg (55%) of the title compound **1k** as an orange solid. **R_f** = 0.24 (2:1 cyclohexane:EtOAc). **^{1}H NMR (300 MHz, $CDCl_3$):** δ 8.87 (d, *J* = 8.7 Hz, 1H), 8.14 (d, *J* = 8.5 Hz, 1H), 7.99 (dd, *J* = 8.4, 1.5 Hz, 1H), 7.76 – 7.70 (m, 1H), 7.62 – 7.56 (m, 1H), 7.33 (d, *J* = 4.4 Hz, 1H), 4.08 (s, 2H), 3.70 (s, 3H). **^{13}C NMR (76 MHz, $CDCl_3$):** δ 170.5, 150.1, 148.4, 139.9, 130.3, 129.4, 127.4, 126.9, 123.5, 122.5, 52.4, 38.1 **HRMS (ESI):** calc'd for $C_{12}H_{12}NO_2$ $[M+H]^+$: 202.0863, found: 202.0857.

Methyl 2-(isoquinolin-1-yl)acetate (1l)

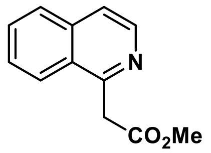

1l

Following **General Procedure D1** on a 3.0 mmol scale with 1-methylisoquinoline. Purification by column chromatography (silica, gradient from 3:1 to 1:3 of cyclohexane:EtOAc) afforded 512 mg (85%) of the title compound **1l** as a green solid. **R_f** = 0.42 (1:1 cyclohexane:EtOAc). **^{1}H NMR (300 MHz, $CDCl_3$):** δ 8.48 (d, *J* = 5.8 Hz, 1H), 8.09 (d, *J* = 8.4 Hz, 1H), 7.85 (d, *J* = 8.7 Hz, 1H), 7.73 – 7.60 (m, 3H), 4.37 (s, 2H), 3.72 (s, 3H). Spectral data is in accordance with previous reports.[4]

Methyl 2-(quinoxalin-2-yl)acetate (1m)

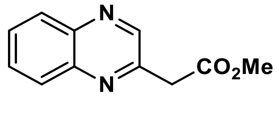

1m

Following **General Procedure D2** on a 5.0 mmol scale with 2-methylquinoxaline. Purification by column chromatography (silica, gradient from 7% to 30% of EtOAc in cyclohexane) afforded 290 mg (29%) of the title compound **1m** as a red liquid. **R_f** = 0.10 (5:1 cyclohexane:EtOAc). **^{1}H NMR (300 MHz, $CDCl_3$):** δ 8.87 (s, 1H), 8.13 – 8.05 (m, 2H), 7.80 – 7.73 (m, 2H), 4.10 (s, 2H), 3.76 (s,

3H). **^{13}C NMR (76 MHz, CDCl$_3$):** δ 170.0, 149.7, 145.8, 142.1, 141.5, 130.2, 129.8, 129.3, 129.1, 52.4, 42.0. **HRMS (APCI):** calc'd for $C_{11}H_{11}N_2O_2$ [M+H]$^+$: 203.0815, found: 203.0820.

Methyl 2-(pyrazin-2-yl)acetate (1n)

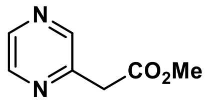

1n

Following **General Procedure D2** on a 10.0 mmol scale with 2-methylpyrazine. Purification by column chromatography (silica, 3:1 pentane:EtOAc) afforded 175 mg (13%) of the title compound **1n** as a brown liquid. **R$_f$** = 0.14 (3:1 pentane:EtOAc). **^{1}H NMR (300 MHz, CDCl$_3$)**: δ 8.60 (d, *J* = 1.2 Hz, 1H), 8.54 – 8.49 (m, 2H), 3.89 (s, 2H), 3.74 (s, 3H). Spectral data is in accordance with previous reports.[4]

1-Phenyl-2-(pyrazin-2-yl)ethenone (1o)

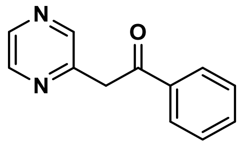

1o

Following **General Procedure D3** on a 10.6 mmol scale with 2-methylpyrazine. Purification by column chromatography (silica, 9:1 cyclohexane:EtOAc) afforded 363 mg (17%) of the title compound **1o** as a yellow solid. **R$_f$** = 0.38 (2:1 cyclohexane:EtOAc). **^{1}H NMR (300 MHz, CDCl$_3$)** (ketone:enol* 5:1): δ 8.63 (s, 1H), 8.55 – 8.49 (m, 2H), 8.49 – 8.48* (m, 1H), 8.29 – 8.28* (m, 2H), 8.08 – 8.04 (m, 2H), 7.87 – 7.83* (m, 2H), 7.64 – 7.58 (m, 1H), 7.52 – 7.47 (m, 2H), 7.44 – 7.42* (m, 3H), 6.16* (s, 1H), 4.54 (s, 2H). Spectral data is in accordance with previous reports.[6]

Supplementary Notes 4

Synthesis and characterization of styryl derivatives from biologically relevant structures.

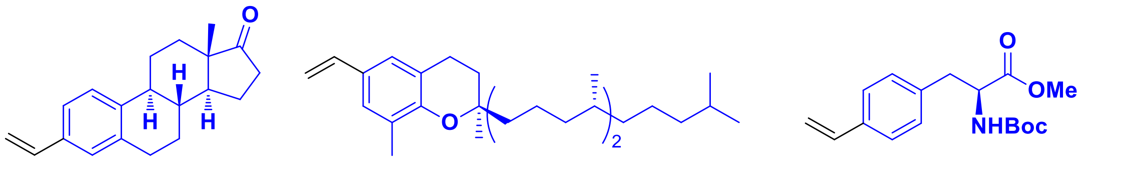

Estrone (hormone) **δ-Tocopherol** (vitamin E) **L-Tyrosine derivative** (amino acid)

Styryl derivatives from biologically relevant structures were synthesized according the reported procedure, with the coupling of potassium vinyltrifluoroborate with the corresponding triflate in the presence of $PdCl_2$, PPh_3 and Cs_2CO_3.[8]

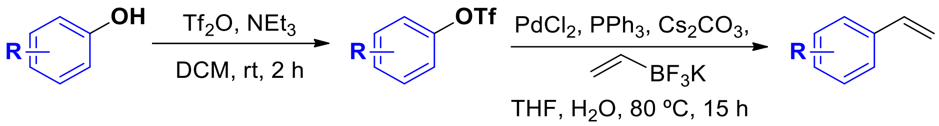

General procedure E for the synthesis of the corresponding triflates.

To a solution of the corresponding phenol (2.5 mmol, 1.0 equiv.) in dry DCM (8.0 mL) under N_2 atmosphere, was added dry triethylamine (0.69 mL, 5.0 mmol, 2.0 equiv.). The reaction mixture was cooled at 0 ºC and trifluoromethanesulfonic anhydride (0.46 mL, 3.7 mmol, 1.5 equiv.) was added dropwise. The resulting mixture was warmed to r.t. and stirred for 2 h. After this time, the reaction was quenched with water (20 mL) and extracted with DCM (3 x 40 mL). The combined organic layers were dried over $MgSO_4$, filtered and concentrated under reduced pressure. The crude product was purified through flash column chromatography.

Estrone-triflate

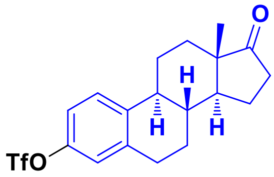

(8*R*,9*S*,13*S*,14*S*)-13-Methyl-3-oxo-7,8,9,11,12,13,15,16,17-decahydro-6*H*-cyclopenta[a] phenanthren-3-yl trifluoromethanesulfonate

Following **General Procedure E** with estrone. Purification by flash column chromatography (silica, 10:1 cyclohexane:EtOAc) afforded 387 mg (40%) of the title compound estrone-triflate as a white solid. **R*f*** = 0.41 (5:1 cyclohexane:EtOAc). **^{1}H NMR (300 MHz, $CDCl_3$):** δ 7.35 (d, *J* = 8.4 Hz, 1H), 7.06 – 6.99 (m, 2H), 2.94 (dd, *J* = 8.7, 2.9 Hz, 2H), 2.57 – 1.97 (m, 7H), 1.68 – 1.47 (m, 6H), 0.92 (s, 3H). Spectral data is in accordance with previous reports.[9]

Boc-Tyr-OMe-triflate

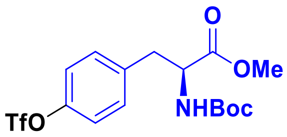

(*S*)-Methyl 2-((tert-butoxycarbonyl)amino)-3-(4-(((trifluoromethyl)sulfonyl)oxy)phenyl) propanoate

Following **General Procedure E** with Boc-Tyr-OMe**.** Purification by flash column chromatography (silica, gradient from 5% to 30% of EtOAc in cyclohexane) afforded 750 mg (70%) of the title compound Boc-Tyr-OMe-triflate as a white solid. **R*f*** = 0.21 (5:1 cyclohexane:EtOAc). **^{1}H NMR (300 MHz, $CDCl_3$):** δ 7.25 – 7.18 (m, 4H), 5.04 – 5.00 (m, 1H), 4.61 – 4.59 (m, 1H), 3.72 (s, 3H), 3.17 (dd, *J* = 14.0, 5.8 Hz, 1H), 3.03 (dd, *J* = 14.0, 6.7 Hz, 1H), 1.41 (s, 9H). Spectral data is in accordance with previous reports.[10]

δ-Tocopherol-triflate

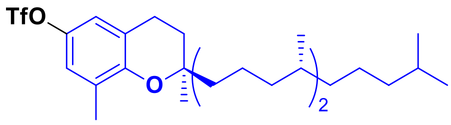

(*R*)-2,8-Dimethyl-2-((4*R*,8*R*)-4,8,12-trimethyltridecyl)chroman-6-yl trifluoromethanesulfonate

Following **General Procedure E** with δ-tocopherol**.** Purification by flash column chromatography (silica, gradient from 3% to 30% of EtOAc in cyclohexane) afforded 1.08 g (85%) of the title compound δ-tocopherol triflate as a yellow oil. **R*f*** = 0.79 (5:1 cyclohexane:EtOAc). **^{1}H NMR (300 MHz, $CDCl_3$):** δ 6.88 – 6.76 (m, 2H), 2.77 – 2.73 (m, 2H), 2.16 (s, 3H), 1.87 – 1.73 (m, 2H), 1.57 – 1.07 (m, 24H), 0.88 – 0.76 (m, 12H). Spectral data is in accordance with previous reports.[11]

General Procedure F for the synthesis of the corresponding vinyl derivatives.

A sealed tube was charged with the corresponding triflate (1.0 mmol, 1.0 equiv.), potassium vinyltrifluoroborate (268 mg, 2.0 mmol, 2.0 equiv.), palladium (II) chloride (17 mg, 0.1 mmol, 0.1 equiv.), triphenyl phosphine (31 mg, 0.12 mmol, 0.12 equiv.) and cesium carbonate (0.97 g, 3.0 mmol, 3.0 equiv.). Then, it was evacuated and refilled with N_2 three times. THF (2.0 mL) and distilled water (0.2 mL) were added, the tube was sealed with the cap and the reaction was stirred at 80 ºC for 15 h. The mixture was filtered, extracted with water (15 mL) and DCM (3 x 30 mL). The combined organic layers were dried over $MgSO_4$, filtered and concentrated under reduced pressure. The crude product was purified through column chromatography.

Vinyl-estrone (SI3)

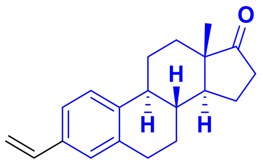

SI3

(8*R*,9*S*,13*S*,14*S*)-13-Methyl-3-vinyl-7,8,9,11,12,13,15,16-octahydro-6*H*-cyclopenta[a] phenanthrene-17(14*H*)-one (SI3)

Following **General Procedure F** with estrone-triflate**.** Purification by column chromatography (silica, gradient from 5% to 20% of EtOAc in cyclohexane) afforded 166 mg (58%) of the title compound **SI3** as a white solid. **R*f*** = 0.40 (10:1 cyclohexane:EtOAc). **^{1}H NMR (300 MHz, $CDCl_3$):** δ 7.26 – 7.19 (m, 2H), 7.15 (s, 1H), 6.67 (dd, *J* = 17.6, 10.9 Hz, 1H), 5.71 (dd, *J* = 17.7, 1.0 Hz, 1H), 5.20 (d, *J* = 11.1 Hz, 1H), 2.92 (dd, *J* = 8.9, 4.2 Hz, 2H), 2.59 – 2.23 (m, 3H), 2.22 – 1.94 (m, 4H), 1.71 – 1.40 (m, 6H), 0.91 (s, 3H). Spectral data is in accordance with previous reports.[9]

Vinyl-Boc-Tyr-OMe (SI4)

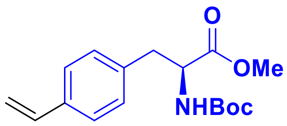

SI4

(*S*)-Methyl 2-((tert-butoxycarbonyl)amino)-3-(4-vinylphenyl)propanoate (SI4)

Following **General Procedure F** with Boc-Tyr-OMe-triflate**.** Purification by column chromatography (silica, gradient from 5% to 25% of EtOAc in cyclohexane) afforded 243 mg (71%) of the title compound **SI4** as a white solid. **R*f*** = 0.40 (4:1 cyclohexane:EtOAc). **^{1}H NMR (300 MHz, $CDCl_3$):** δ ^{1}H NMR (300 MHz, $CDCl_3$) δ 7.33 (d, *J* = 8.1 Hz, 2H), 7.08 (d, *J* = 8.1 Hz, 2H), 6.68 (dd, *J* = 17.6, 10.9 Hz, 1H), 5.71 (d, *J* = 17.6 Hz, 1H), 5.23 (d, *J* = 10.8 Hz, 1H), 5.02 – 5.00 (m, 1H), 4.64 – 4.54 (m, 1H), 3.71 (s, 3H), 3.14 – 3.00 (m, 2H), 1.42 (s, 9H). Spectral data is in accordance with previous reports.[10]

Vinyl-δ-tocopherol (SI5)

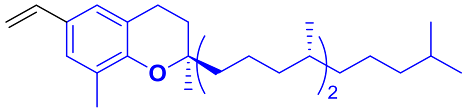

SI5

(*R*)-2,8-Dimethyl-2-((4*R*,8*R*)-4,8,12-trimethyltridecyl)-6-vinylchroman (SI5)

Following **General Procedure F** with δ-tocopherol-triflate**.** Purification by column chromatography (silica, gradient from 1% to 10% of EtOAc in cyclohexane) afforded 338 mg (78%) of the title

compound **SI5** as a colorless oil. **R*f*** = 0.83 (10:1 cyclohexane:EtOAc). **^{1}H NMR (300 MHz, $CDCl_3$):** δ 7.05 (s, 1H), 6.95 (s, 1H), 6.59 (dd, *J* = 17.6, 10.9 Hz, 1H), 5.55 (dd, *J* = 17.6, 1.1 Hz, 1H), 5.04 (dd, *J* = 10.8, 1.1 Hz, 1H), 2.74 (t, *J* = 6.8 Hz, 2H), 2.17 (s, 3H), 1.87 – 1.68 (m, 2H), 1.63 – 1.07 (m, 24H), 0.91 – 0.80 (m, 12H). Spectral data is in accordance with previous reports.[12]

Supplementary Notes 5

Mechanistic studies on the photocatalytic reaction

- **Time resolved luminescence quenching experiments**

For the steady-state and time resolved fluorescence quenching studies of **3a*** or **3c***, increasing concentrations of quencher were added to a solution 2 mM of **3** in THF (for **3a**) or CH_3CN (for **3b**) under N_2 atmosphere (λ_{exc} = 450 nm).

a)

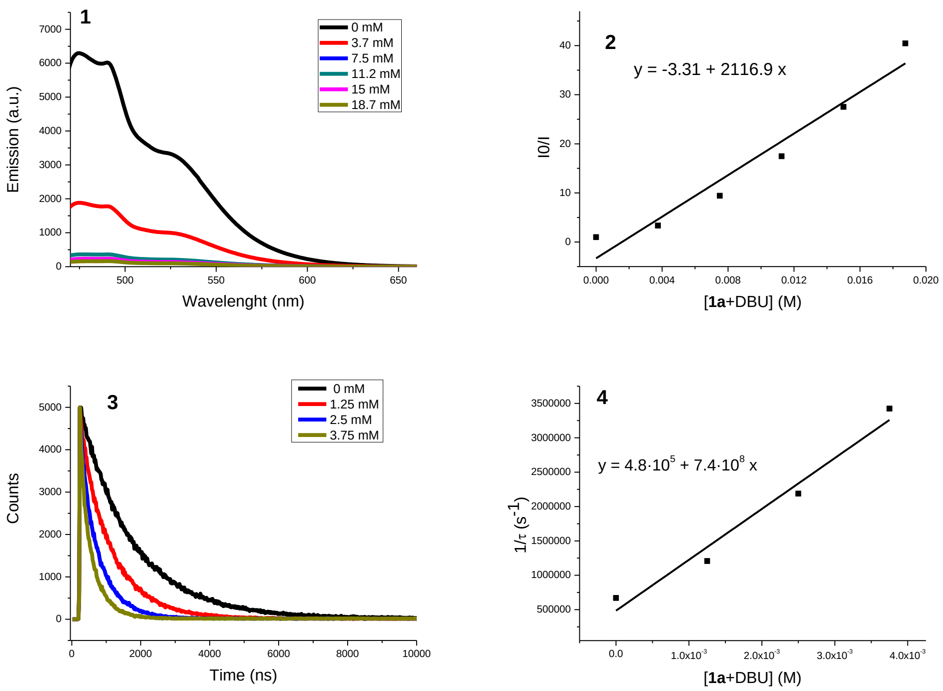

Kq **= 7.4x10⁸ M⁻¹ s⁻¹**

b)

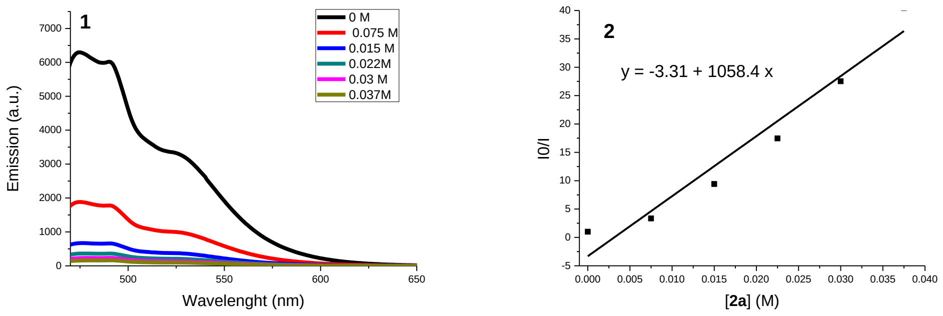

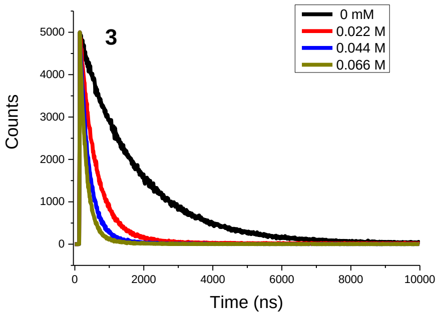

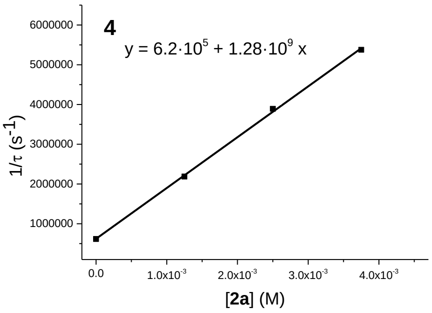

***Kq* = 1.3x10^{9} M^{-1} s^{-1}**

---

c)

3

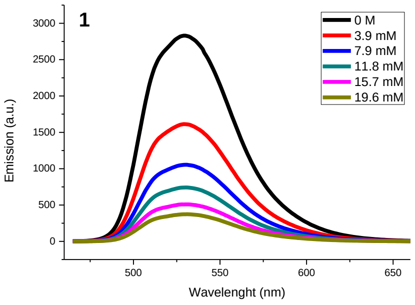

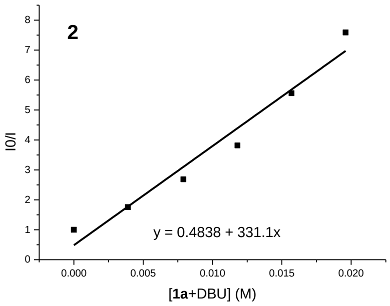

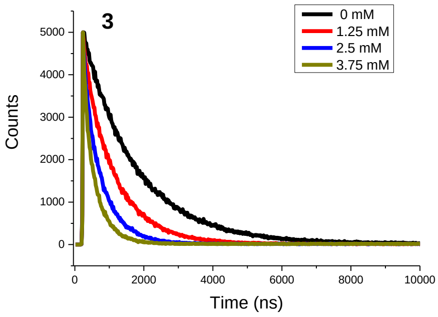

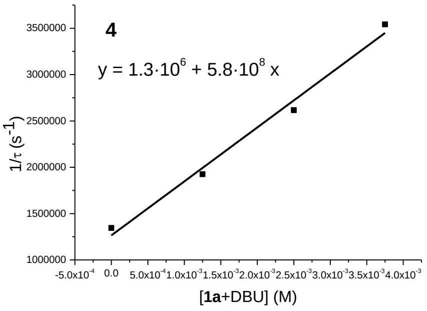

***Kq* = 5.8x10^{8} M^{-1} s^{-1}**

---

d)

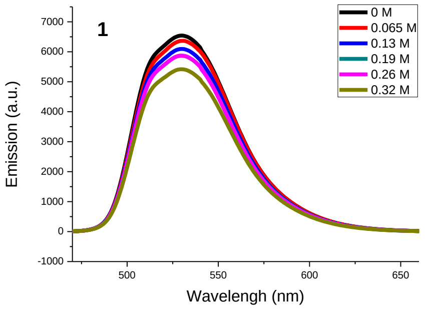

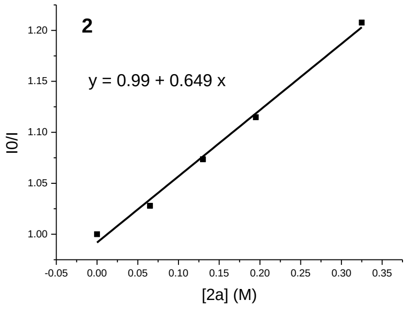

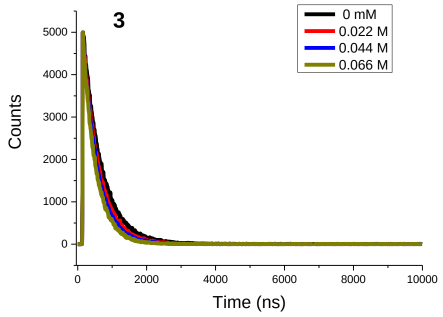

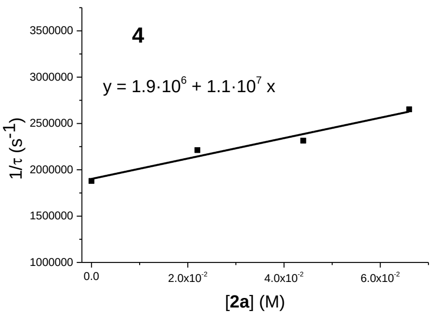

K_q = 1.1x10^{7} M^{-1} s^{-1}

Supplementary Fig. 2.: a) Quenching studies of **3a** with (**1a**+DBU): (1) Steady state luminescence quenching spectrum and (2) Stern-Volmer plot of the steady state; (3) Time resolved luminescence quenching and (4) Stern-Volmer plot to obtain *Kq*; b) Quenching studies of **3a** with **2a**: (1) Steady state luminescence quenching spectrum and (2) Stern-Volmer plot of the steady state; (3) Time resolved luminescence quenching and (4) Stern-Volmer plot to obtain *Kq*; time resolved luminescence quenching of **1a*** with **3a**; c) Quenching studies of **3c** with (**1a**+DBU): (1) Steady state luminescence quenching spectrum and (2) Stern-Volmer plot of the steady state; (3) Time resolved luminescence quenching and (4) Stern-Volmer plot to obtain *Kq*; d) Quenching studies of **3c** with **2a**: (1) Steady state luminescence quenching spectrum and (2) Stern-Volmer plot of the steady state; (3) Time resolved luminescence quenching and (4) Stern-Volmer plot to obtain *Kq*.

▪ Regioselectivity of the reaction

In addition, the formation of the observed unique regioisomer **4a** can be explained assuming that the [2+2] cycloaddition takes place through the formation of the most stable 1,4-biradical (equation a) in comparison with the other regioisomer (equation b).[13]

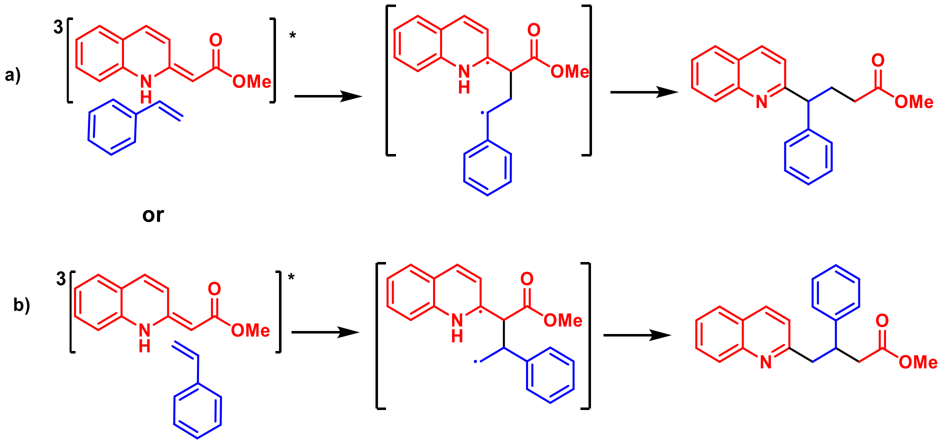

Supplementary Fig. 3.: Possible resioselectivities of the reaction.

▪ DFT calculations: optimized geometries

General methods for DFT calculations:

Quantum chemistry calculations were carried out using the density functional theory (DFT). In particular, geometry optimizations were performed using the M06-2X functional[14] in combination with the 6-311G**[15] basis set including acetonitrile (ε = 37.5) solvent effects with the solvation model density (SMD).[16] All optimizations were performed without any geometrical constraint and harmonic vibrational frequencies have been also evaluated at the same level of theory to characterize minima and transition states in the potential energy surface. Transition states have been connected to products by optimization of geometries slightly modified from the transition states. All the calculations were performed using the Gaussian09 program.[17]

Calculated triplet energies for **1a´** and **2a**:

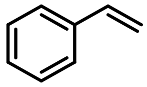

ΔG (T_1-S_0) = 54.9 kcal mol^{-1}

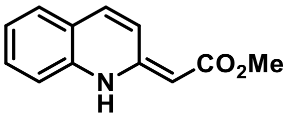

ΔG (T_1-S_0) = 48.8 kcal mol^{-1}

Supplementary Tables 1.

Optimized geometries:

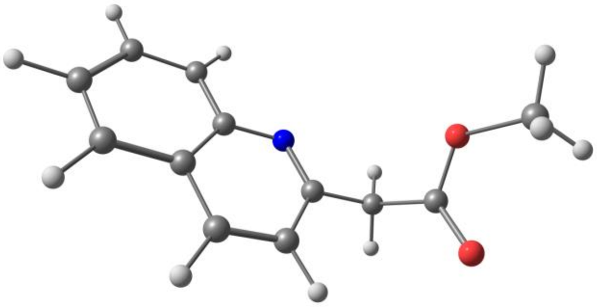

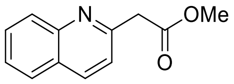

S = 0

Sum of electronic and thermal Free Energies= -668.868272

6	-4.139001000	-0.143600000	-0.666888000
6	-3.756811000	-1.346430000	-0.025730000
6	-2.495284000	-1.491683000	0.491595000
6	-1.555803000	-0.435662000	0.390040000
6	-1.939517000	0.769858000	-0.250792000
6	-3.249342000	0.893598000	-0.777039000
7	-0.307332000	-0.620031000	0.915285000
6	0.557609000	0.359005000	0.821106000
6	0.265670000	1.605600000	0.199041000
6	-0.974475000	1.806275000	-0.332222000
6	1.941606000	0.133133000	1.390259000
6	2.940723000	0.098341000	0.255655000
8	3.585240000	1.042731000	-0.121990000
8	2.990017000	-1.105859000	-0.314804000
6	3.856898000	-1.220642000	-1.452431000
1	-5.140231000	-0.047536000	-1.070266000
1	-4.471478000	-2.157389000	0.054894000
1	-2.183707000	-2.404840000	0.985779000
1	-3.529667000	1.820294000	-1.266679000
1	1.032870000	2.370118000	0.157803000
1	-1.236633000	2.742384000	-0.814358000
1	2.216889000	0.954956000	2.051983000
1	1.953529000	-0.812662000	1.928689000
1	3.782112000	-2.255023000	-1.777465000
1	3.528293000	-0.548546000	-2.245984000
1	4.883646000	-0.983276000	-1.172604000

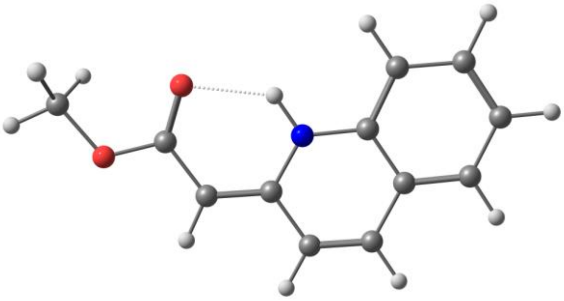 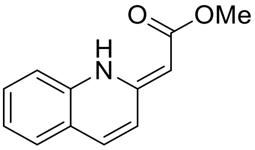

S = 0

Sum of electronic and thermal Free Energies= -668.865574

6	4.246089000	-0.701842000	0.000846000
6	3.413713000	-1.830326000	0.000472000
6	2.037580000	-1.697893000	-0.000131000
6	1.468775000	-0.416795000	-0.000331000
6	2.291909000	0.724145000	0.000072000
6	3.685191000	0.561245000	0.000632000
7	0.099616000	-0.257410000	-0.000707000
6	-0.543497000	0.946795000	-0.000615000
6	0.304944000	2.121579000	-0.000273000
6	1.648353000	2.014659000	-0.000005000
6	-1.917805000	1.056257000	-0.000630000
6	-2.780910000	-0.090586000	-0.000684000
8	-2.427061000	-1.265216000	-0.002296000
8	-4.085970000	0.247103000	0.001549000
6	-5.010273000	-0.841502000	0.001538000
1	5.322353000	-0.822675000	0.001338000
1	3.850701000	-2.822206000	0.000697000
1	1.390175000	-2.567939000	-0.000370000
1	4.311227000	1.447362000	0.000940000
1	-0.186839000	3.086148000	-0.000250000
1	2.272507000	2.902223000	0.000232000
1	-2.359049000	2.041820000	-0.000418000
1	-0.497099000	-1.083668000	-0.001331000
1	-5.999551000	-0.389168000	0.009489000
1	-4.886137000	-1.455273000	-0.892216000
1	-4.875760000	-1.464259000	0.887474000

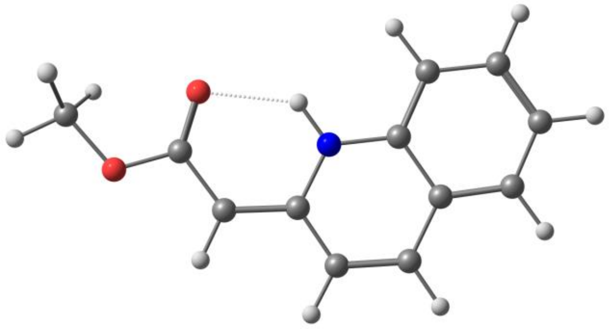

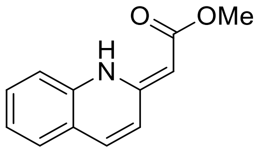

S = 1

Sum of electronic and thermal Free Energies= -668.787677

6	-4.254104000	-0.717753000	0.004051000
6	-3.426560000	-1.850094000	0.003316000
6	-2.044912000	-1.694091000	0.000747000
6	-1.483551000	-0.416783000	-0.001022000
6	-2.307751000	0.757030000	-0.000151000
6	-3.713215000	0.552938000	0.002329000
7	-0.113934000	-0.255644000	-0.003469000
6	0.496656000	0.985745000	-0.002816000
6	-0.276214000	2.117317000	-0.002048000
6	-1.699077000	2.023108000	-0.001495000
6	1.938634000	1.040123000	-0.000340000
6	2.813638000	-0.114662000	-0.003039000
8	2.458652000	-1.283033000	-0.014376000
8	4.101906000	0.249777000	0.008989000
6	5.050878000	-0.822661000	0.006718000
1	-5.331276000	-0.841666000	0.006060000
1	-3.857503000	-2.843565000	0.004853000
1	-1.385721000	-2.555916000	0.000231000
1	-4.356796000	1.426005000	0.002970000
1	0.219125000	3.079763000	-0.001154000
1	-2.311226000	2.915765000	-0.000986000
1	2.399110000	2.018790000	0.004987000
1	0.485044000	-1.076762000	-0.006139000
1	6.028731000	-0.349176000	0.040796000
1	4.907398000	-1.460551000	0.879800000
1	4.949652000	-1.418436000	-0.901539000

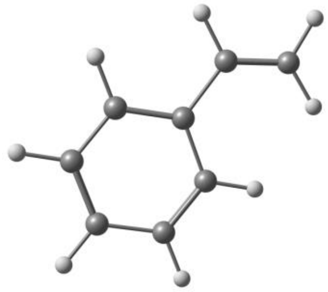

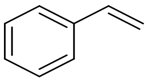

S = 0

Sum of electronic and thermal Free Energies= -309.487778

6	0.016445000	1.087122000	-0.000111000
6	-1.350865000	1.327866000	-0.000027000
6	-2.254592000	0.265687000	0.000063000
6	-1.777657000	-1.040523000	0.000066000
6	-0.406905000	-1.280872000	0.000009000
6	0.510208000	-0.224491000	-0.000076000
6	1.953128000	-0.537045000	-0.000124000
6	2.955860000	0.340354000	0.000165000
1	0.702977000	1.926204000	-0.000201000
1	-1.715730000	2.348742000	-0.000058000
1	-3.321269000	0.458063000	0.000118000
1	-2.470566000	-1.874226000	0.000138000
1	-0.036614000	-2.301079000	0.000019000
1	2.187306000	-1.599294000	-0.000381000
1	3.984878000	-0.000463000	0.000115000
1	2.795281000	1.413463000	0.000460000

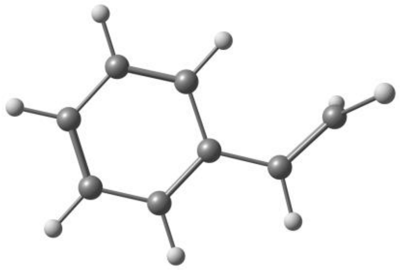

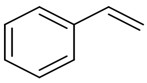

S = 1

Sum of electronic and thermal Free Energies= -309.400174

6	-0.071034000	1.061402000	-0.000043000
6	1.280220000	1.364642000	-0.000022000
6	2.236883000	0.347282000	0.000045000
6	1.822615000	-0.987802000	0.000021000
6	0.475818000	-1.303172000	-0.000032000
6	-0.511995000	-0.285784000	0.000006000
6	-1.892521000	-0.622827000	-0.000012000
6	-2.974521000	0.353876000	0.000066000
1	-0.807825000	1.858082000	-0.000076000
1	1.596848000	2.401874000	-0.000043000
1	3.292933000	0.590363000	0.000062000
1	2.560085000	-1.782827000	0.000055000
1	0.156831000	-2.340580000	-0.000033000
1	-2.144751000	-1.682635000	-0.000145000
1	-3.423543000	0.695038000	0.927950000
1	-3.423370000	0.694980000	-0.927947000

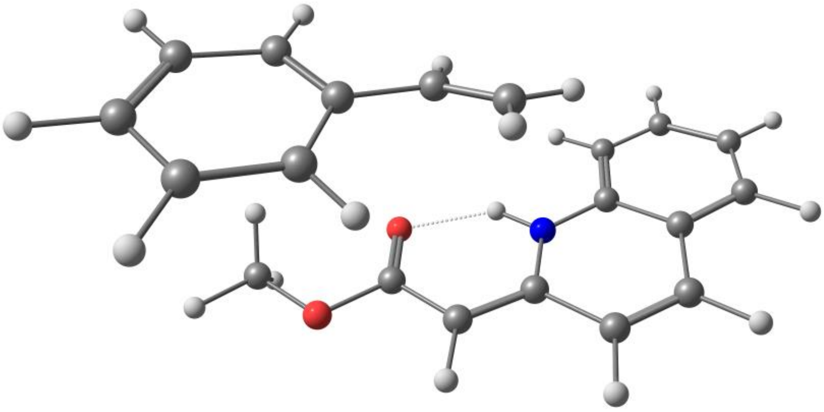

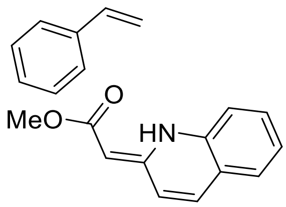

S = 0

Sum of electronic and thermal Free Energies= -978.344339

6	5.286040000	-1.316081000	0.201979000
6	4.414426000	-1.698622000	1.228156000
6	3.185665000	-1.084569000	1.381606000
6	2.804142000	-0.065909000	0.498010000
6	3.669793000	0.323443000	-0.541361000
6	4.910457000	-0.313139000	-0.670778000
7	1.585609000	0.556317000	0.626614000
6	1.096354000	1.497527000	-0.238199000
6	2.001725000	1.924581000	-1.287123000
6	3.217424000	1.365970000	-1.430751000
6	-0.180179000	1.989825000	-0.138429000
6	-1.104995000	1.506334000	0.854244000
8	-0.862030000	0.672003000	1.717954000
8	-2.323198000	2.062162000	0.736608000
6	-3.313819000	1.541724000	1.620257000
1	6.245991000	-1.804313000	0.093608000
1	4.703130000	-2.486692000	1.913209000
1	2.510829000	-1.378608000	2.177458000
1	5.571539000	-0.005508000	-1.473783000
1	1.650503000	2.696436000	-1.959692000
1	3.879282000	1.686179000	-2.228366000
1	-0.518780000	2.739623000	-0.837097000
1	0.918906000	0.245852000	1.334472000
1	-4.243378000	2.031934000	1.340983000
1	-3.400217000	0.461105000	1.496573000
1	-3.056588000	1.766339000	2.656677000
6	-0.822873000	-1.661740000	-0.464564000
6	0.064835000	-1.382266000	-1.415603000
6	-2.260027000	-1.332984000	-0.514855000
6	-3.165627000	-2.079672000	0.245421000
6	-2.754947000	-0.290873000	-1.305349000
6	-4.529346000	-1.818392000	0.192084000
6	-4.116704000	-0.023803000	-1.354483000
6	-5.010494000	-0.790487000	-0.612496000
1	-2.791916000	-2.878079000	0.877583000
1	-2.062445000	0.335062000	-1.856848000
1	-5.215634000	-2.414540000	0.782037000

1	-4.479567000	0.795591000	-1.963818000
1	-6.072720000	-0.580366000	-0.651920000
1	-0.497523000	-2.192662000	0.426992000
1	-0.218984000	-0.877170000	-2.332599000
1	1.109042000	-1.652938000	-1.304849000

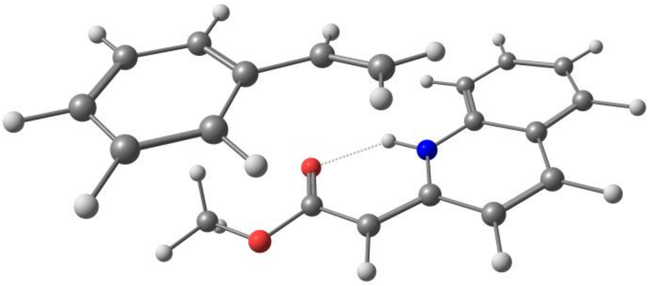

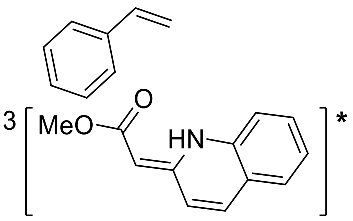

S = 1

Sum of electronic and thermal Free Energies= -978.266751

6	5.443511000	1.276399000	-0.245397000
6	4.586260000	1.693091000	-1.273383000
6	3.326440000	1.116342000	-1.397275000
6	2.917838000	0.126656000	-0.502905000
6	3.774177000	-0.313573000	0.559517000
6	5.051740000	0.299451000	0.649122000
7	1.670804000	-0.451164000	-0.620786000
6	1.200058000	-1.405639000	0.259148000
6	2.004800000	-1.841234000	1.278812000
6	3.315863000	-1.306075000	1.443715000
6	-0.156752000	-1.874773000	0.094818000
6	-1.078575000	-1.409825000	-0.923052000
8	-0.834618000	-0.582125000	-1.787543000
8	-2.272124000	-2.003155000	-0.805757000
6	-3.285716000	-1.526897000	-1.696659000
1	6.425529000	1.726354000	-0.150005000
1	4.900288000	2.460667000	-1.969828000
1	2.646602000	1.422827000	-2.185367000
1	5.717121000	-0.017988000	1.444635000
1	1.616839000	-2.587357000	1.960722000
1	3.954400000	-1.646817000	2.248494000
1	-0.514540000	-2.620911000	0.791656000
1	1.035915000	-0.129313000	-1.346542000
1	-4.197382000	-2.046815000	-1.412184000
1	-3.408610000	-0.448397000	-1.583015000
1	-3.024030000	-1.761229000	-2.729891000
6	-0.956523000	1.497204000	0.816377000
6	-0.166840000	1.010923000	1.773150000
6	-2.401187000	1.232636000	0.666115000
6	-3.158782000	2.054189000	-0.177184000
6	-3.049121000	0.187575000	1.338093000
6	-4.528537000	1.859167000	-0.324769000
6	-4.416377000	-0.008236000	1.191029000
6	-5.162922000	0.829078000	0.363186000
1	-2.664034000	2.857562000	-0.714002000
1	-2.477119000	-0.484456000	1.968953000
1	-5.099732000	2.509977000	-0.977230000
1	-4.901926000	-0.823799000	1.715495000
1	-6.229307000	0.671296000	0.248329000
1	-0.531457000	2.165499000	0.070410000
1	-0.535628000	0.356218000	2.557128000
1	0.889262000	1.258334000	1.799392000

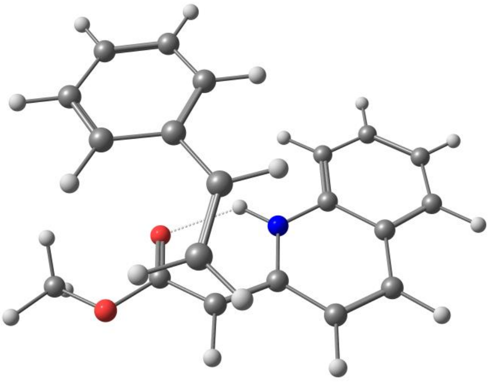

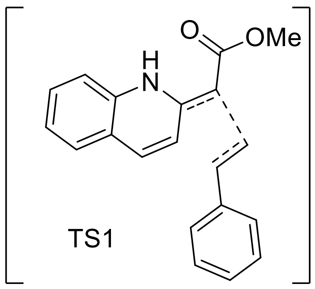

TS1

Sum of electronic and thermal Free Energies= -978.255282

6	4.847781000	1.382855000	-0.407450000
6	3.778217000	2.016841000	-1.046132000
6	2.529007000	1.399375000	-1.082137000
6	2.346866000	0.153544000	-0.484500000
6	3.423858000	-0.513284000	0.176202000
6	4.676243000	0.145610000	0.192338000
7	1.111229000	-0.468624000	-0.520860000
6	0.863451000	-1.688102000	0.074910000
6	1.885102000	-2.339153000	0.717158000
6	3.185064000	-1.774655000	0.769084000
6	-0.509554000	-2.185337000	0.076901000
6	-1.532986000	-1.685718000	-0.823341000
8	-1.423853000	-0.729392000	-1.574332000
8	-2.677501000	-2.381384000	-0.713240000
6	-3.752416000	-1.937422000	-1.547843000
1	5.819741000	1.862503000	-0.379298000
1	3.914236000	2.984841000	-1.513150000
1	1.685346000	1.874796000	-1.572323000
1	5.507576000	-0.344358000	0.688206000
1	1.672991000	-3.289539000	1.191588000
1	3.992999000	-2.291427000	1.271718000
1	-0.662423000	-3.174783000	0.487995000
1	0.348474000	-0.029010000	-1.027991000
1	-4.600082000	-2.570977000	-1.298197000
1	-3.987584000	-0.891197000	-1.347932000
1	-3.490163000	-2.055367000	-2.600426000
6	-0.974942000	0.055457000	1.892890000
6	-1.353225000	-1.247358000	2.005075000
6	-1.714434000	1.095359000	1.191668000
6	-1.058042000	2.297249000	0.881300000
6	-3.049663000	0.936086000	0.782552000
6	-1.705256000	3.300258000	0.170807000
6	-3.695067000	1.941390000	0.078129000
6	-3.025698000	3.125110000	-0.234974000
1	-0.028485000	2.431299000	1.198293000
1	-3.588554000	0.025808000	1.023557000
1	-1.180237000	4.218614000	-0.065783000
1	-4.725889000	1.805211000	-0.228993000
1	-3.534249000	3.907206000	-0.786810000
1	0.010697000	0.340223000	2.253089000
1	-2.353864000	-1.581552000	1.753882000
1	-0.754133000	-1.924346000	2.603352000

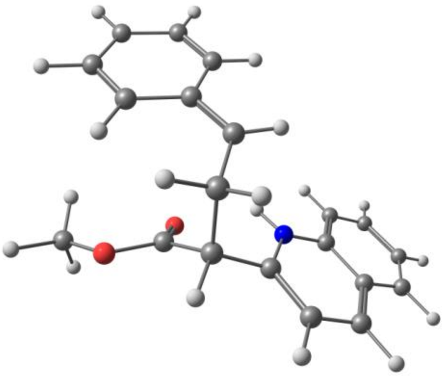

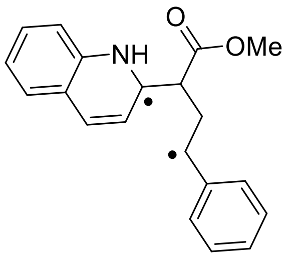

1,4-birradical S = 1

Sum of electronic and thermal Free Energies= -978.294202

6	-5.223384000	1.614608000	0.529616000
6	-4.119387000	2.272359000	1.069665000
6	-2.848198000	1.711080000	0.955373000
6	-2.680130000	0.491081000	0.303206000
6	-3.793451000	-0.201206000	-0.248874000
6	-5.061424000	0.396624000	-0.119225000
7	-1.420911000	-0.073788000	0.176086000
6	-1.196363000	-1.295301000	-0.430073000
6	-2.265495000	-1.976364000	-0.958601000
6	-3.567807000	-1.458173000	-0.887555000
6	0.220120000	-1.775333000	-0.588993000
6	1.049668000	-1.675229000	0.676182000
8	0.896308000	-0.863236000	1.555755000
8	2.033065000	-2.573747000	0.675857000
6	2.940894000	-2.506923000	1.786687000
1	-6.211590000	2.051364000	0.617831000
1	-4.242236000	3.221361000	1.577975000
1	-1.978399000	2.211246000	1.368592000
1	-5.919356000	-0.120077000	-0.536685000
1	-2.077549000	-2.927120000	-1.443981000
1	-4.406446000	-2.000764000	-1.305387000
1	0.180737000	-2.827283000	-0.874867000
1	-0.649548000	0.362674000	0.666700000
1	3.702417000	-3.257116000	1.590325000
1	3.387225000	-1.513772000	1.850674000
1	2.414709000	-2.733847000	2.714365000
6	1.195749000	0.428537000	-1.482273000
6	0.984602000	-1.027686000	-1.734499000
6	2.361199000	0.997900000	-0.908162000
6	2.422940000	2.400747000	-0.697785000
6	3.487857000	0.229386000	-0.515363000
6	3.535674000	2.992863000	-0.129823000
6	4.597342000	0.834310000	0.052854000
6	4.633444000	2.215599000	0.251657000
1	1.571565000	3.007392000	-0.989713000
1	3.489353000	-0.845335000	-0.658238000
1	3.554695000	4.066351000	0.021354000
1	5.445490000	0.224667000	0.345086000
1	5.504169000	2.680780000	0.698093000
1	0.388748000	1.106018000	-1.740152000
1	1.932132000	-1.547212000	-1.894980000
1	0.382187000	-1.160221000	-2.636568000

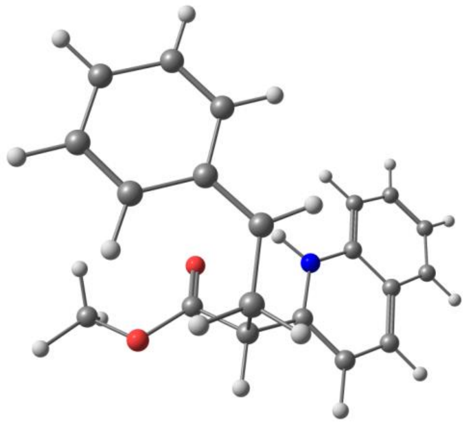 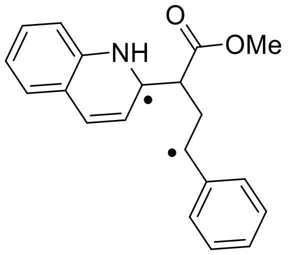

Open shell singlet 1,4-birradical
Sum of electronic and thermal Free Energies= -978.296719

6	-5.103162000	1.630245000	0.536694000
6	-3.977739000	2.292242000	1.028751000
6	-2.719346000	1.708333000	0.914840000
6	-2.583087000	0.458855000	0.309673000
6	-3.716107000	-0.235807000	-0.191038000
6	-4.972304000	0.385474000	-0.063591000
7	-1.334612000	-0.129932000	0.182686000
6	-1.144417000	-1.380595000	-0.371418000
6	-2.238645000	-2.063040000	-0.859466000
6	-3.523627000	-1.523854000	-0.783370000
6	0.256286000	-1.844535000	-0.580112000
6	1.133485000	-1.723803000	0.647845000
8	0.997202000	-0.909900000	1.529043000
8	2.127109000	-2.610348000	0.620027000
6	3.062538000	-2.534652000	1.707046000
1	-6.082347000	2.086582000	0.625325000
1	-4.077676000	3.262916000	1.500008000
1	-1.833688000	2.211883000	1.288432000
1	-5.845420000	-0.134550000	-0.443772000
1	-2.072519000	-3.031380000	-1.317613000
1	-4.380570000	-2.067297000	-1.161288000
1	0.232202000	-2.894104000	-0.874219000
1	-0.553479000	0.297356000	0.665654000
1	3.825026000	-3.278728000	1.492121000
1	3.501996000	-1.537805000	1.759464000
1	2.561970000	-2.764613000	2.648110000
6	1.093609000	0.401500000	-1.543959000
6	0.962919000	-1.058767000	-1.767666000
6	2.207915000	1.037796000	-0.933456000
6	2.183010000	2.440765000	-0.719605000
6	3.369586000	0.338167000	-0.516096000
6	3.245553000	3.096338000	-0.124148000
6	4.428180000	1.005450000	0.078684000
6	4.378380000	2.385758000	0.282128000
1	1.304468000	2.997903000	-1.029706000
1	3.442121000	-0.733179000	-0.667286000
1	3.196779000	4.168746000	0.029030000
1	5.304868000	0.445359000	0.385935000
1	5.209462000	2.900160000	0.749808000
1	0.273283000	1.037952000	-1.857320000
1	1.933206000	-1.535182000	-1.926056000
1	0.349937000	-1.255739000	-2.648414000

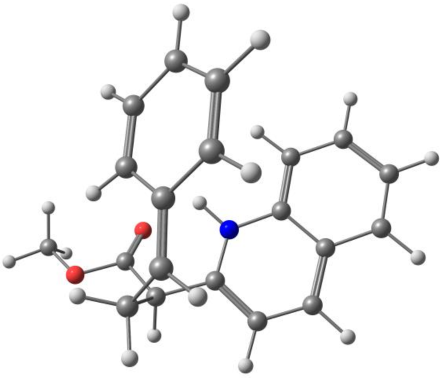

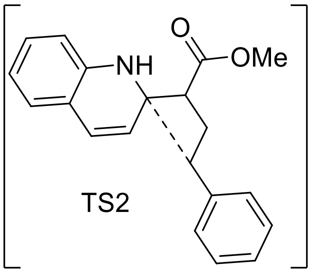

Sum of electronic and thermal Free Energies= -978.284288

6	4.500793000	-0.398330000	-0.850908000
6	3.662510000	0.467424000	-1.589630000
6	2.295709000	0.383754000	-1.488864000
6	1.744192000	-0.580429000	-0.626659000
6	2.559496000	-1.450919000	0.123373000
6	3.963497000	-1.341104000	-0.011725000
7	0.383150000	-0.685737000	-0.491938000
6	-0.226101000	-1.526463000	0.328261000
6	0.560694000	-2.420231000	1.084173000
6	1.923653000	-2.385354000	0.981401000
6	-1.715239000	-1.471735000	0.508192000
6	-2.470725000	-1.027735000	-0.725535000
8	-1.999998000	-0.831362000	-1.821923000
8	-3.769563000	-0.922003000	-0.464762000
6	-4.599809000	-0.520047000	-1.565893000
1	5.575693000	-0.310560000	-0.952125000
1	4.105338000	1.206995000	-2.245852000
1	1.636012000	1.038887000	-2.047552000
1	4.595062000	-2.008682000	0.563160000
1	0.057085000	-3.113584000	1.745258000
1	2.538211000	-3.064497000	1.562290000
1	-2.059939000	-2.494229000	0.688486000
1	-0.195280000	-0.034657000	-1.026770000
1	-5.608889000	-0.465251000	-1.166449000
1	-4.282567000	0.454423000	-1.938323000
1	-4.544784000	-1.257183000	-2.367129000
6	-0.966880000	0.353486000	2.217550000
6	-2.028265000	-0.631354000	1.831345000
6	-0.630746000	1.440553000	1.421258000
6	0.545302000	2.244011000	1.660545000
6	-1.386707000	1.830364000	0.256265000
6	0.942769000	3.244854000	0.803369000
6	-0.964306000	2.846841000	-0.591825000
6	0.210405000	3.564354000	-0.358272000
1	1.145338000	2.022017000	2.539654000
1	-2.340346000	1.349671000	0.056284000
1	1.849642000	3.799183000	1.031223000
1	-1.576257000	3.089021000	-1.457483000
1	0.534039000	4.351506000	-1.027719000
1	-0.311582000	0.087710000	3.040838000
1	-3.017556000	-0.187647000	1.673014000
1	-2.146236000	-1.367676000	2.627716000

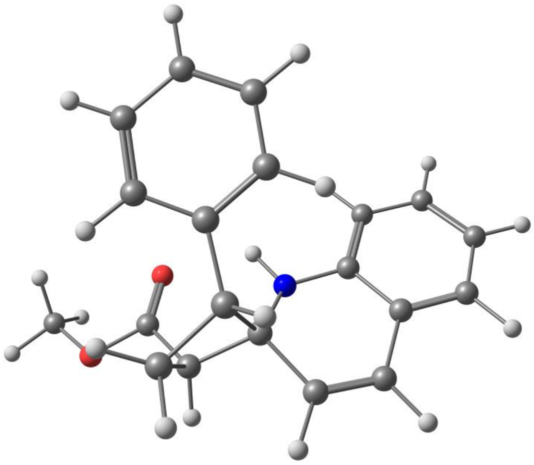

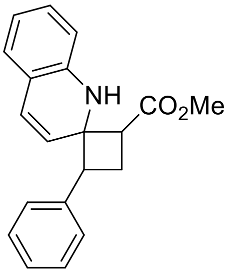

Sum of electronic and thermal Free Energies= -978.324724

6	-4.774521000	0.032608000	0.748956000
6	-3.921206000	0.723483000	1.610760000
6	-2.547453000	0.526314000	1.561907000
6	-1.999035000	-0.377530000	0.641980000
6	-2.855094000	-1.080053000	-0.230772000
6	-4.232687000	-0.868096000	-0.160973000
7	-0.644770000	-0.632801000	0.608156000
6	0.004378000	-1.134594000	-0.574662000
6	-0.910997000	-2.098085000	-1.282733000
6	-2.237883000	-2.040717000	-1.140471000
6	1.422303000	-1.750109000	-0.408824000
6	2.264933000	-1.263478000	0.741994000
8	1.942780000	-0.493352000	1.613938000
8	3.485124000	-1.800027000	0.669846000
6	4.412013000	-1.388926000	1.684818000
1	-5.844828000	0.193220000	0.790709000
1	-4.329366000	1.429640000	2.325647000
1	-1.884393000	1.067789000	2.229136000
1	-4.878106000	-1.424913000	-0.833512000
1	-0.449651000	-2.801165000	-1.969112000
1	-2.885241000	-2.708701000	-1.700122000
1	1.455904000	-2.841415000	-0.403470000
1	-0.061339000	-0.055916000	1.200952000
1	5.336971000	-1.917624000	1.469781000
1	4.570886000	-0.310885000	1.637506000
1	4.039066000	-1.661793000	2.672463000
6	0.622485000	-0.116270000	-1.643148000
6	1.831823000	-1.068130000	-1.746112000
6	0.839181000	1.270447000	-1.096263000
6	-0.286508000	2.094009000	-0.964290000
6	2.073798000	1.769723000	-0.680802000
6	-0.185917000	3.372237000	-0.430719000
6	2.179921000	3.054301000	-0.147923000
6	1.053881000	3.858114000	-0.017628000
1	-1.253635000	1.718865000	-1.287643000
1	2.971251000	1.167062000	-0.765751000
1	-1.072354000	3.990237000	-0.340509000
1	3.149521000	3.423111000	0.167494000
1	1.139763000	4.855651000	0.397892000
1	0.012792000	-0.049857000	-2.546418000
1	2.827704000	-0.629735000	-1.781052000

1	1.724735000	-1.758426000	-2.581929000

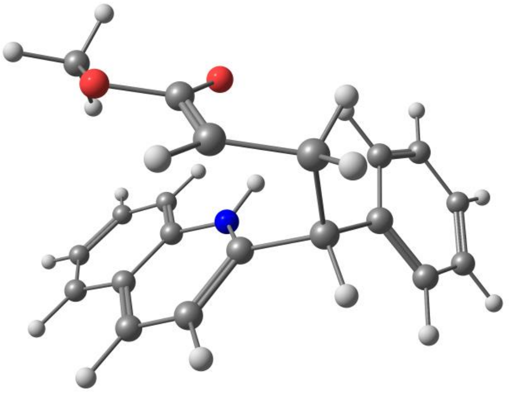

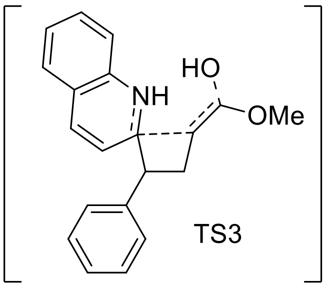

Sum of electronic and thermal Free Energies= -978.301133

6	-3.913477000	-2.274697000	-0.403221000
6	-3.054554000	-2.164221000	-1.510484000
6	-1.815495000	-1.571233000	-1.384343000
6	-1.422330000	-1.077840000	-0.132460000
6	-2.264670000	-1.182046000	0.987992000
6	-3.523230000	-1.793121000	0.827940000
7	-0.179849000	-0.492708000	0.026898000
6	0.212802000	0.135309000	1.137644000
6	-0.589677000	-0.038335000	2.308854000
6	-1.787414000	-0.669625000	2.239232000
6	-0.022077000	2.478929000	0.806110000
6	-0.655864000	2.283802000	-0.417476000
8	-0.147063000	1.781945000	-1.439452000
8	-1.997253000	2.612670000	-0.417091000
6	-2.729155000	2.197965000	-1.557867000
1	-4.884291000	-2.740589000	-0.521921000
1	-3.370112000	-2.543069000	-2.475341000
1	-1.143662000	-1.472996000	-2.230239000
1	-4.176023000	-1.872901000	1.690221000
1	-0.217180000	0.376408000	3.237094000
1	-2.406707000	-0.785816000	3.121750000
1	-0.563757000	2.944499000	1.618854000
1	0.363645000	-0.342313000	-0.821587000
1	-3.736252000	2.595012000	-1.432048000
1	-2.293187000	2.586852000	-2.480164000
1	-2.776221000	1.105522000	-1.625597000
6	1.638631000	0.697984000	1.220738000
6	1.460286000	2.205112000	0.906366000
6	2.646105000	-0.054770000	0.373821000
6	3.494287000	-0.989082000	0.971772000
6	2.747152000	0.158665000	-1.005665000
6	4.424666000	-1.695061000	0.215100000
6	3.673564000	-0.551898000	-1.764285000
6	4.516420000	-1.477894000	-1.156549000
1	3.427044000	-1.159224000	2.041579000
1	2.091863000	0.881526000	-1.484724000
1	5.079574000	-2.412167000	0.697063000
1	3.739422000	-0.376453000	-2.832159000
1	5.241958000	-2.024994000	-1.747514000
1	1.930829000	0.569294000	2.265815000
1	1.963021000	2.457113000	-0.030623000
1	1.927694000	2.798682000	1.694276000

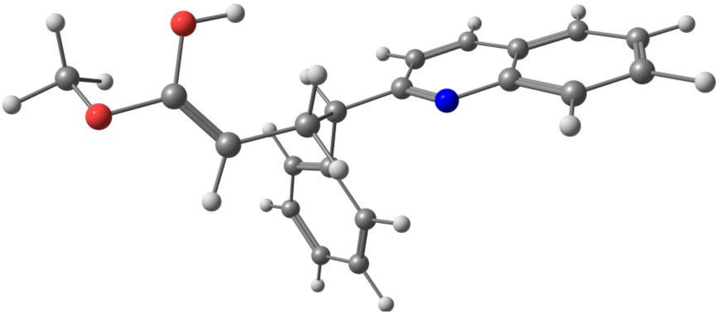 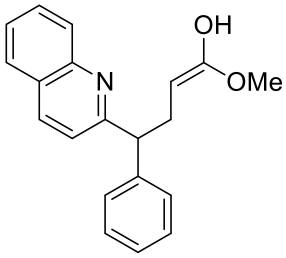

Sum of electronic and thermal Free Energies= -978.327772

6	5.767936000	-0.780525000	-0.102771000
6	5.148407000	-1.067872000	-1.342167000
6	3.786242000	-0.983229000	-1.478235000
6	2.979668000	-0.604694000	-0.376411000
6	3.601077000	-0.314786000	0.864077000
6	5.009728000	-0.411675000	0.978703000
7	1.625445000	-0.533502000	-0.551155000
6	0.877455000	-0.181611000	0.465553000
6	1.414092000	0.132427000	1.748787000
6	2.761466000	0.063292000	1.944553000
6	-2.632648000	-0.939687000	-1.016831000
6	-3.429282000	-1.793276000	-0.366487000
8	-3.034052000	-2.741627000	0.516564000
8	-4.758556000	-1.825641000	-0.577993000
6	-5.568733000	-1.969166000	0.593256000
1	6.845574000	-0.853788000	-0.013577000
1	5.759661000	-1.357075000	-2.189407000
1	3.293976000	-1.200121000	-2.419477000
1	5.472430000	-0.189351000	1.934657000
1	0.739553000	0.418341000	2.547852000
1	3.204980000	0.288708000	2.909176000
1	-3.120647000	-0.210757000	-1.651170000
1	-2.091098000	-2.645118000	0.698916000
1	-6.596375000	-1.834094000	0.262196000
1	-5.451775000	-2.958271000	1.036629000
1	-5.316565000	-1.199996000	1.329175000
6	-0.621498000	-0.068807000	0.261248000
6	-1.133163000	-0.945838000	-0.900627000
6	-1.005962000	1.388084000	0.061680000
6	-1.946773000	1.999793000	0.889338000
6	-0.436375000	2.129913000	-0.977297000
6	-2.313984000	3.328657000	0.685941000
6	-0.801830000	3.455366000	-1.182425000
6	-1.742479000	4.059675000	-0.350084000
1	-2.395620000	1.430348000	1.697357000
1	0.300897000	1.663815000	-1.624375000
1	-3.047017000	3.790133000	1.338156000
1	-0.350125000	4.019186000	-1.991011000
1	-2.025473000	5.093886000	-0.509255000
1	-1.102493000	-0.407562000	1.185112000
1	-0.748014000	-1.962549000	-0.758247000
1	-0.689186000	-0.589303000	-1.831987000

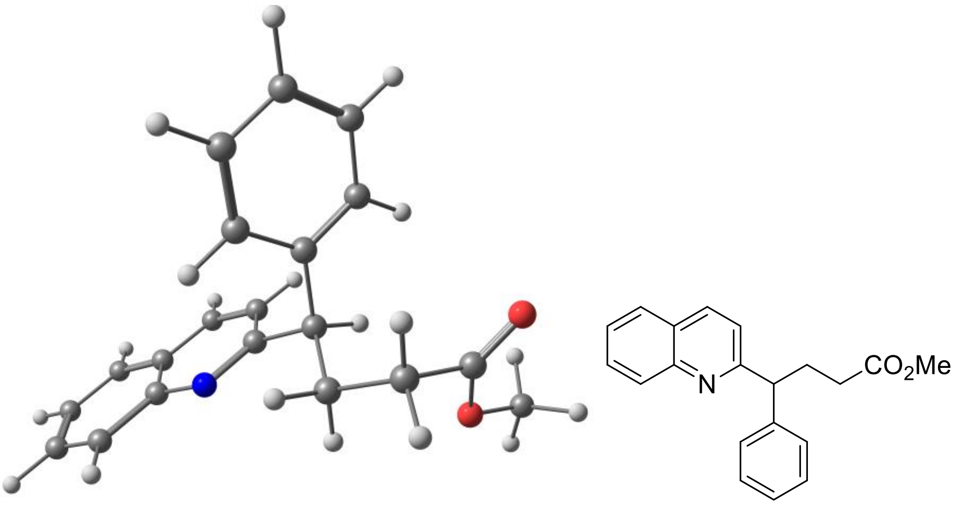

4a

Sum of electronic and thermal Free Energies= -978.369823

6	5.688698000	-0.411949000	0.037003000
6	5.171757000	-0.593205000	-1.267700000
6	3.817885000	-0.592316000	-1.488270000
6	2.917997000	-0.407163000	-0.409840000
6	3.436471000	-0.225639000	0.896977000
6	4.838475000	-0.232865000	1.098284000
7	1.575904000	-0.408763000	-0.671918000
6	0.741117000	-0.234313000	0.322159000
6	1.169872000	-0.044344000	1.668749000
6	2.503211000	-0.043004000	1.951606000
6	-2.618865000	-0.898513000	-1.549576000
6	-3.419651000	-1.538420000	-0.443582000
8	-4.424446000	-1.082671000	0.041516000
8	-2.892600000	-2.707631000	-0.057489000
6	-3.592254000	-3.391175000	0.989120000
1	6.761082000	-0.415957000	0.192769000
1	5.856059000	-0.733457000	-2.096481000
1	3.402620000	-0.729441000	-2.480376000
1	5.222843000	-0.094212000	2.103386000
1	0.425135000	0.094244000	2.444563000
1	2.866163000	0.093139000	2.965227000
1	-2.823976000	-1.465092000	-2.463194000
1	-3.011859000	0.109539000	-1.686865000
1	-3.039890000	-4.310955000	1.164441000
1	-3.609289000	-2.782099000	1.893875000
1	-4.614020000	-3.615758000	0.681150000
6	-0.747716000	-0.188149000	0.028624000
6	-1.111744000	-0.887461000	-1.287236000
6	-1.227814000	1.255946000	0.046623000
6	-2.254300000	1.655842000	0.901295000
6	-0.664688000	2.196537000	-0.820829000
6	-2.715904000	2.970651000	0.889165000
6	-1.124298000	3.508550000	-0.834992000
6	-2.152656000	3.899596000	0.020704000
1	-2.698585000	0.929791000	1.574774000
1	0.140654000	1.896495000	-1.484906000
1	-3.516864000	3.266663000	1.557222000
1	-0.679949000	4.228347000	-1.513335000
1	-2.510766000	4.922605000	0.009428000
1	-1.250115000	-0.706245000	0.853790000
1	-0.735035000	-1.912130000	-1.257172000
1	-0.612736000	-0.383368000	-2.116794000

Supplementary Figures 1

NMR Spectra

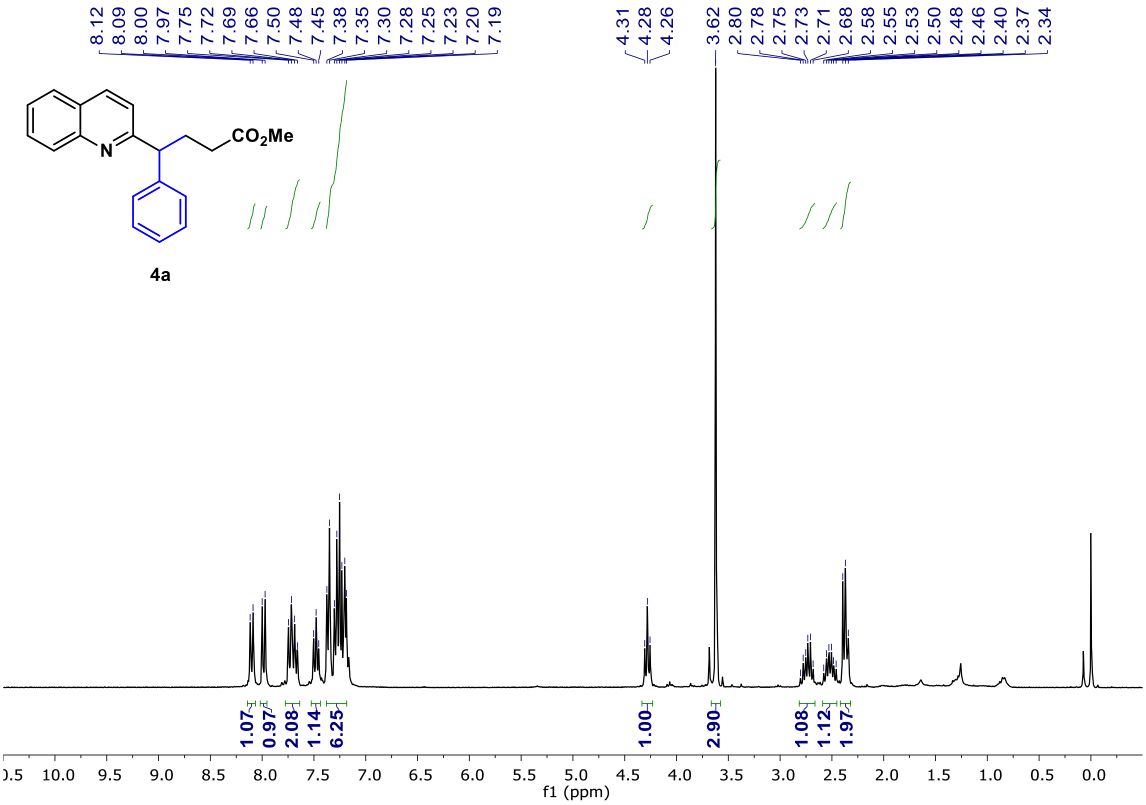

Supplementary Fig. 4. ^{1}H NMR Spectra of **4a**.

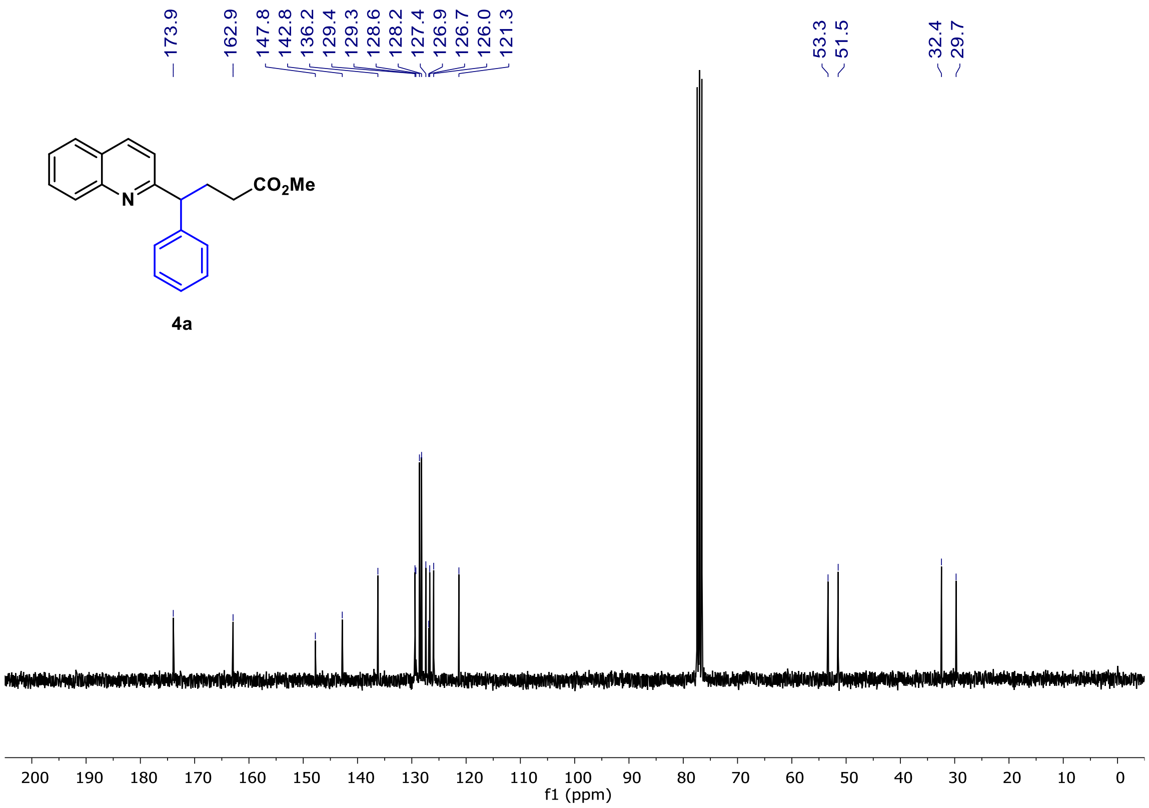

Supplementary Fig. 5. ^{13}C NMR Spectra of **4a**.

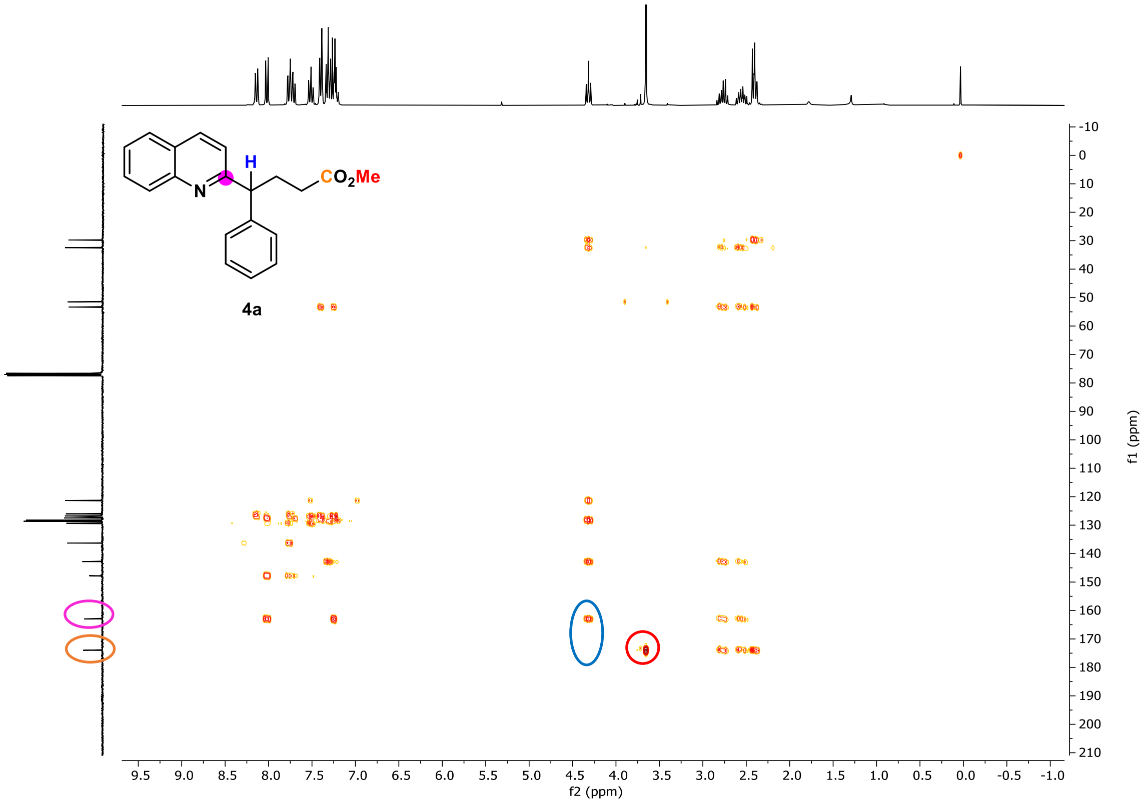

Supplementary Fig. 6. HMBC Spectra of **4a**.

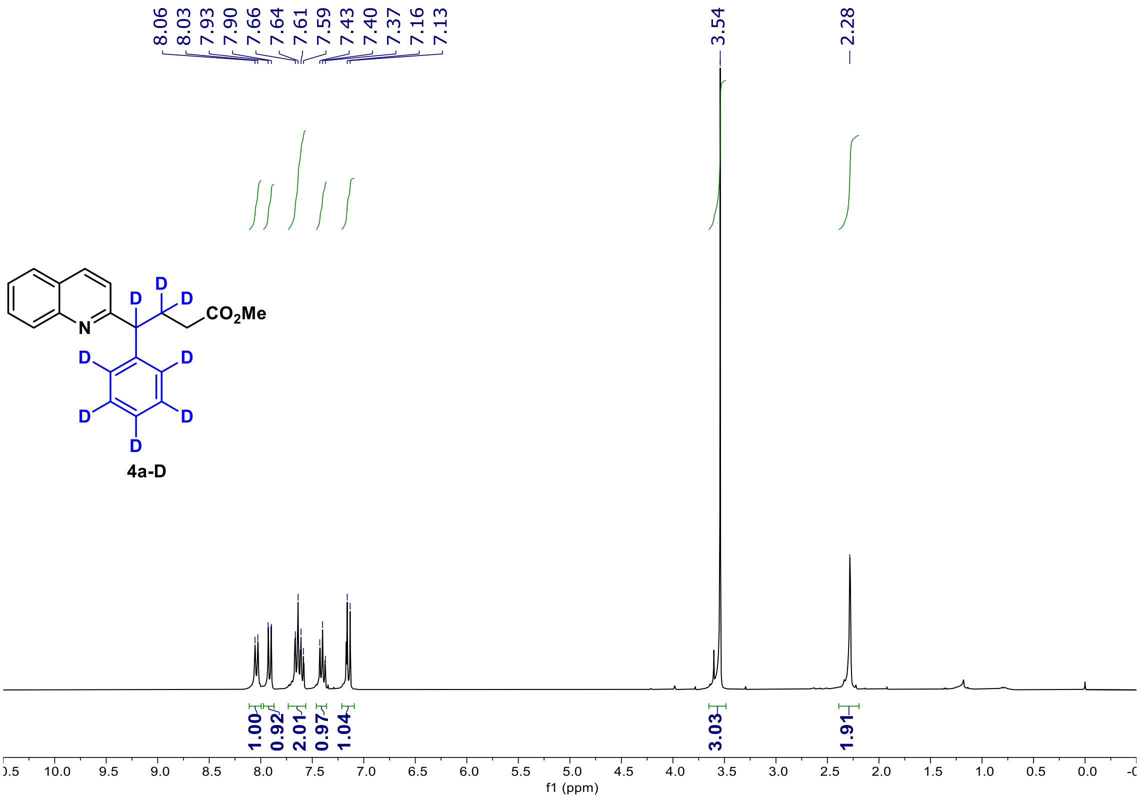

Supplementary Fig. 7. ^{1}H NMR Spectra of **4a'**.

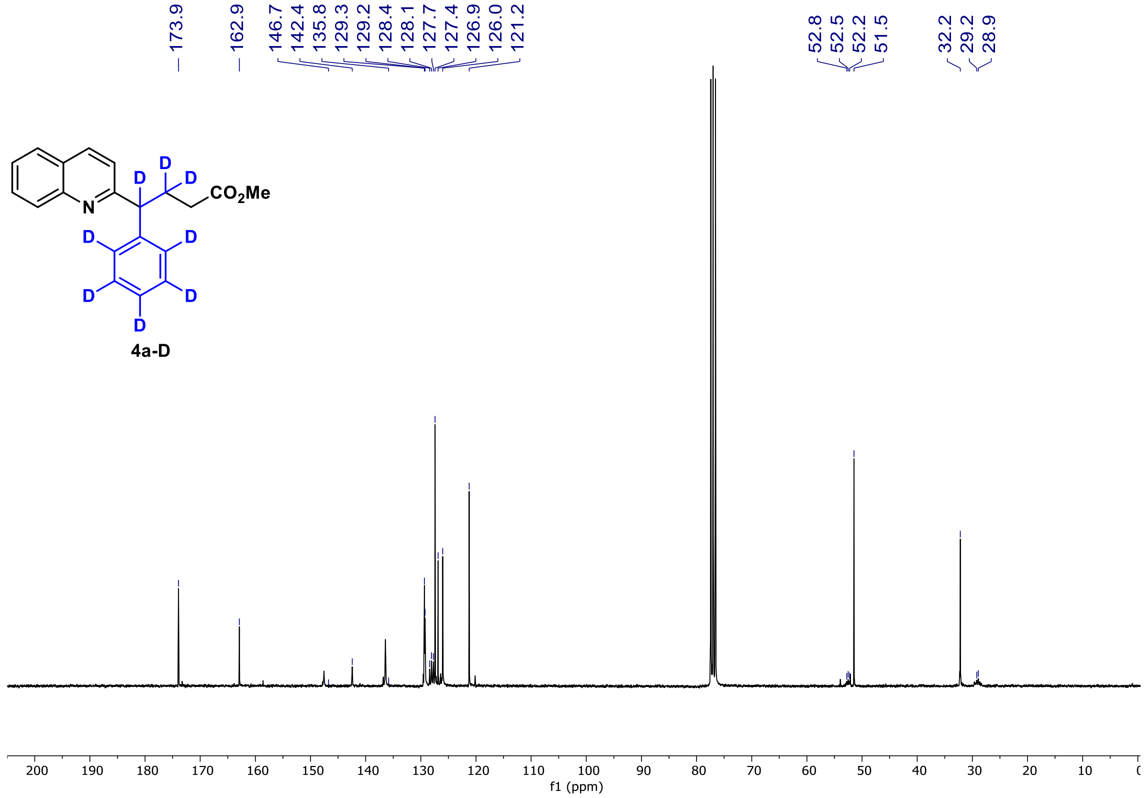

Supplementary Fig. 8. ^{13}C NMR Spectra of **4a’**.

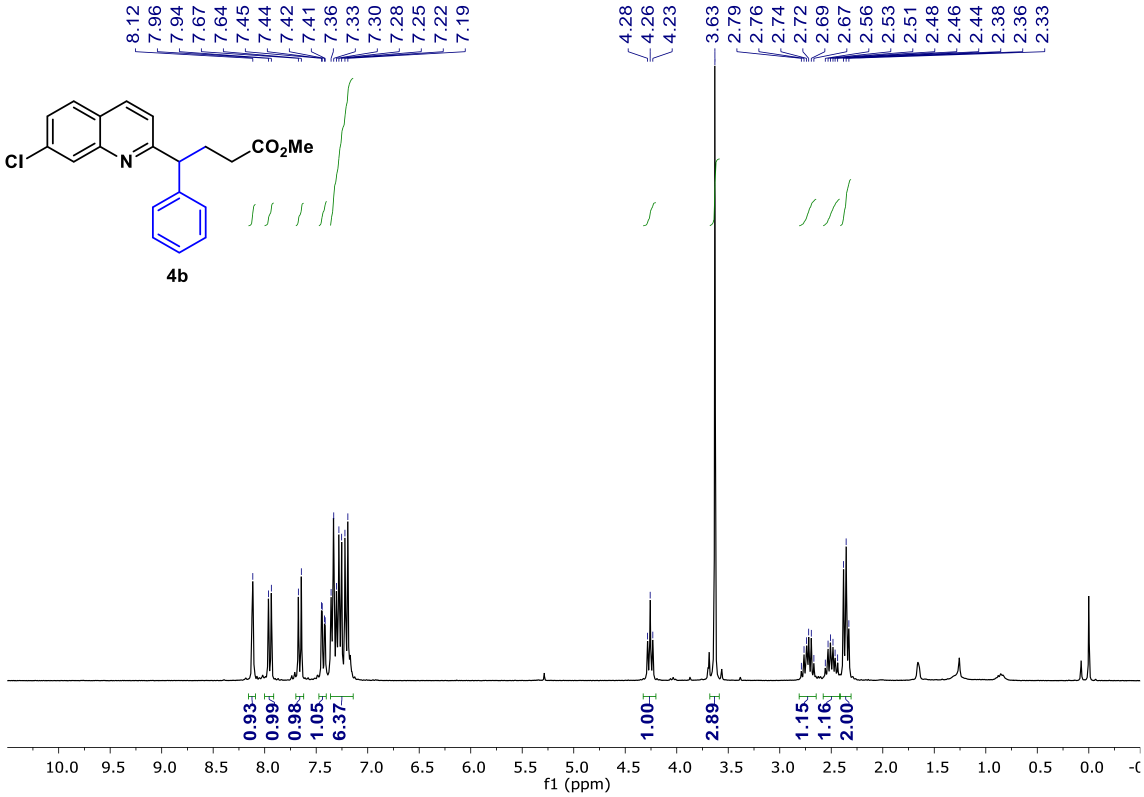

Supplementary Fig. 9. ^{1}H NMR Spectra of **4b**.

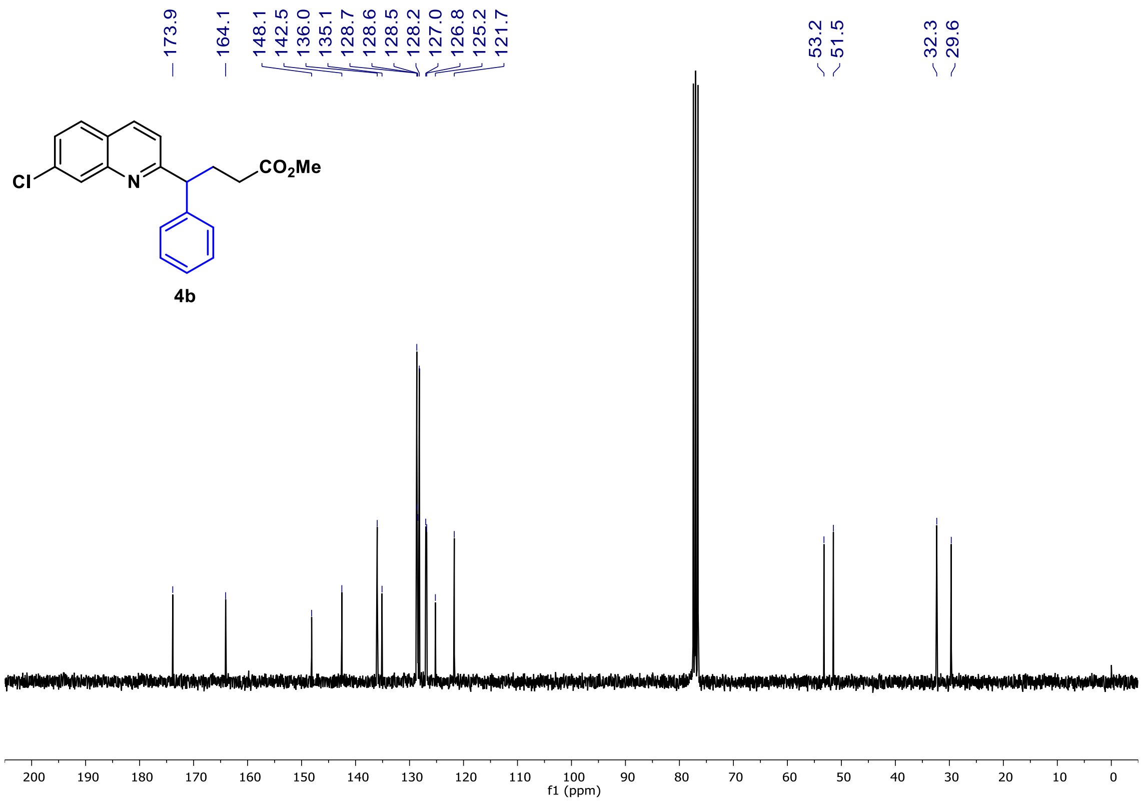

Supplementary Fig. 10. ^{13}C NMR Spectra of **4b**.

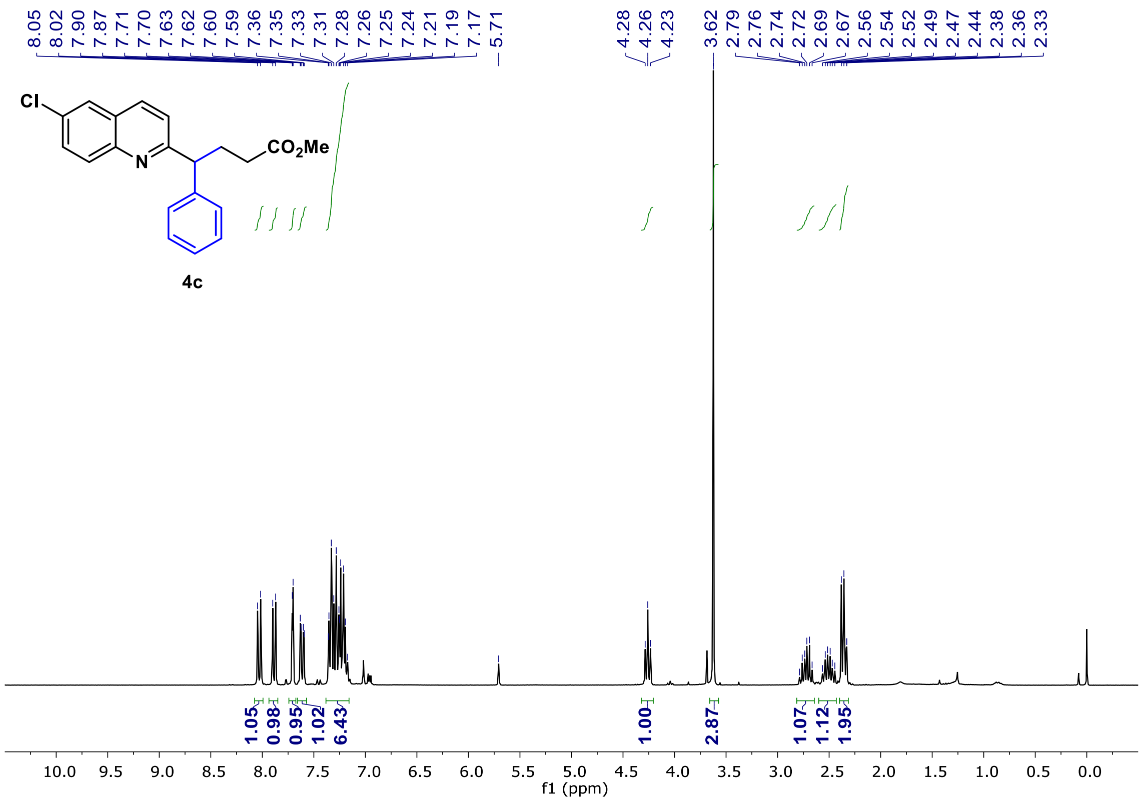

Supplementary Fig. 11. ^{1}H NMR Spectra of **4c**.

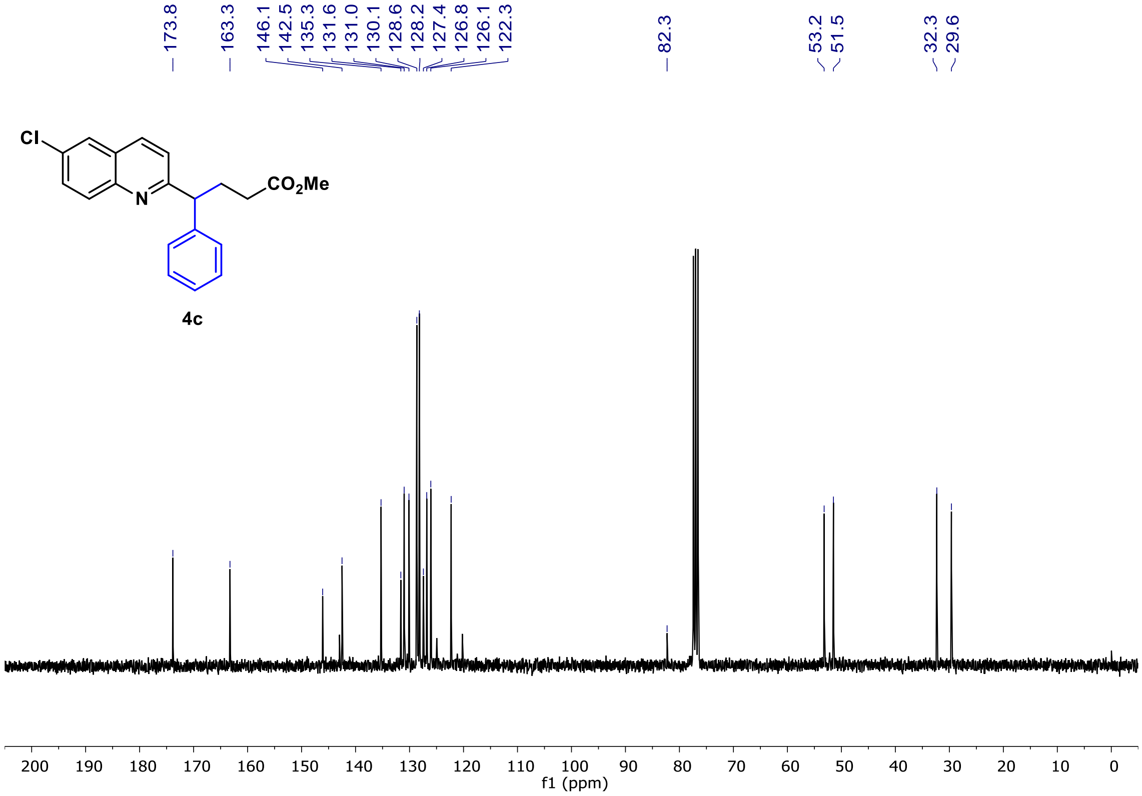

Supplementary Fig. 12. ^{13}C NMR Spectra of **4c**.

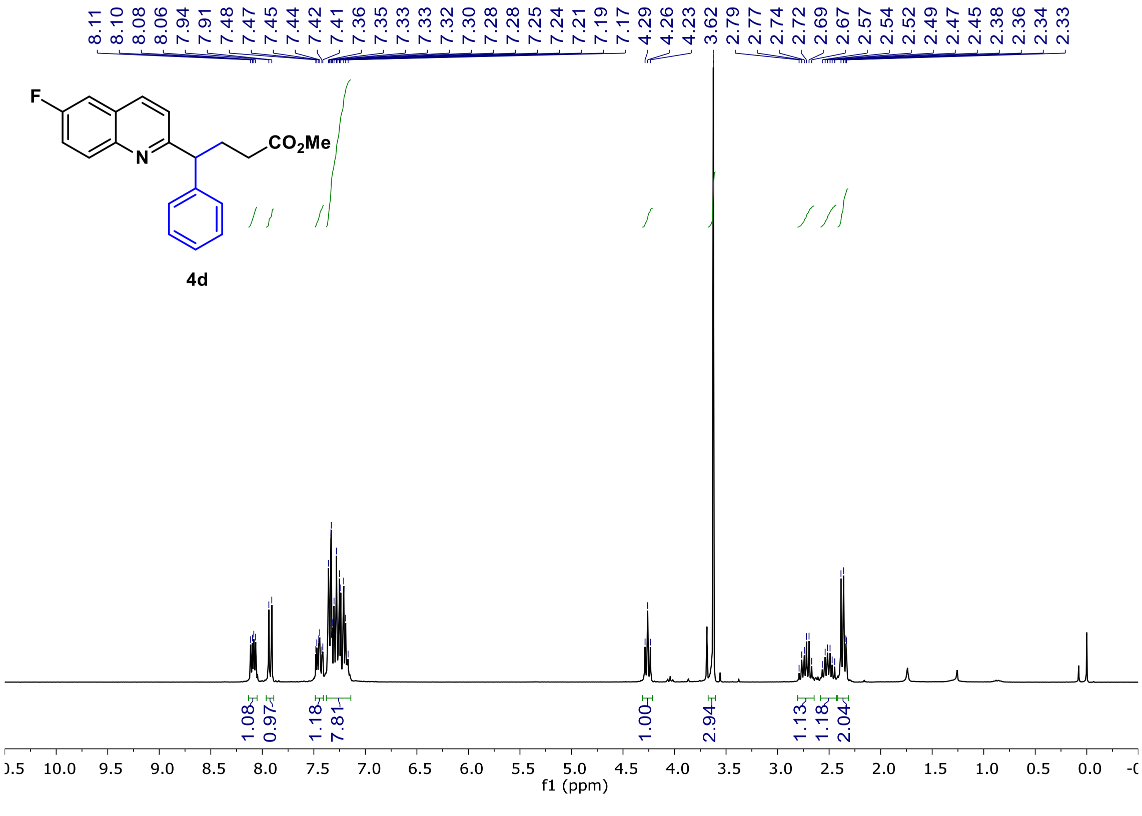

Supplementary Fig. 13. ^{1}H NMR Spectra of **4d**.

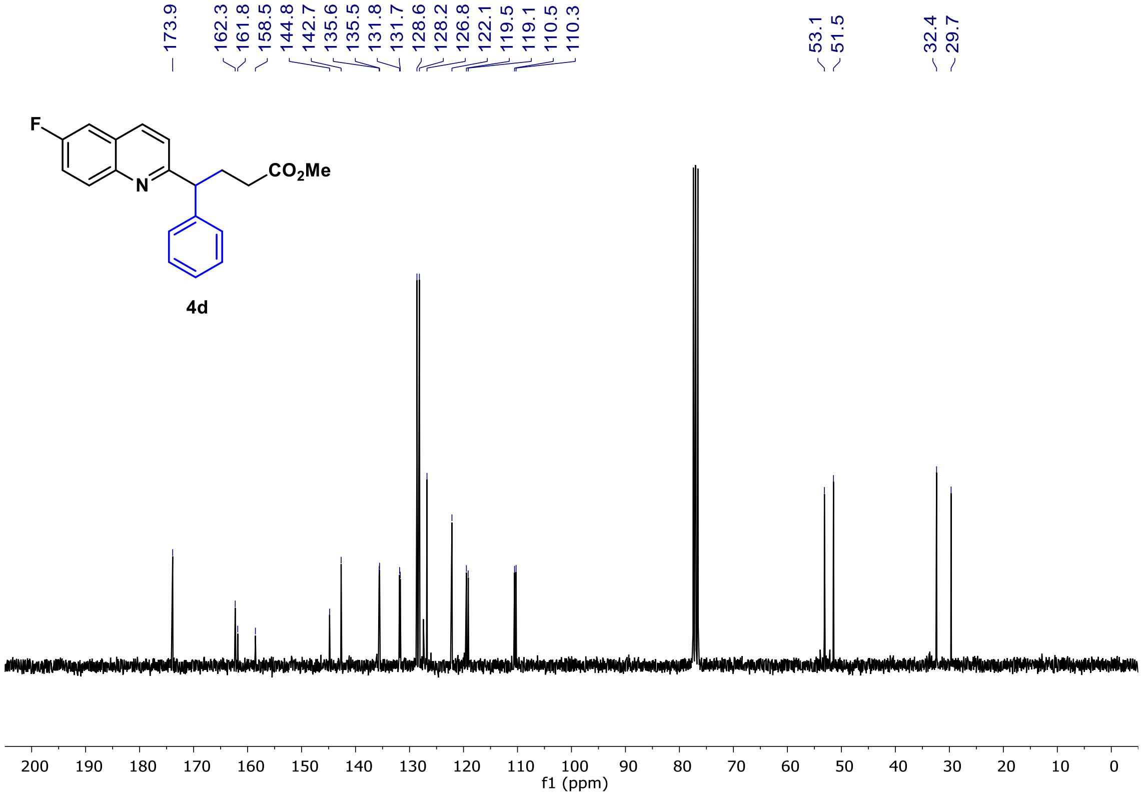

Supplementary Fig. 14. ^{13}C NMR Spectra of **4d**.

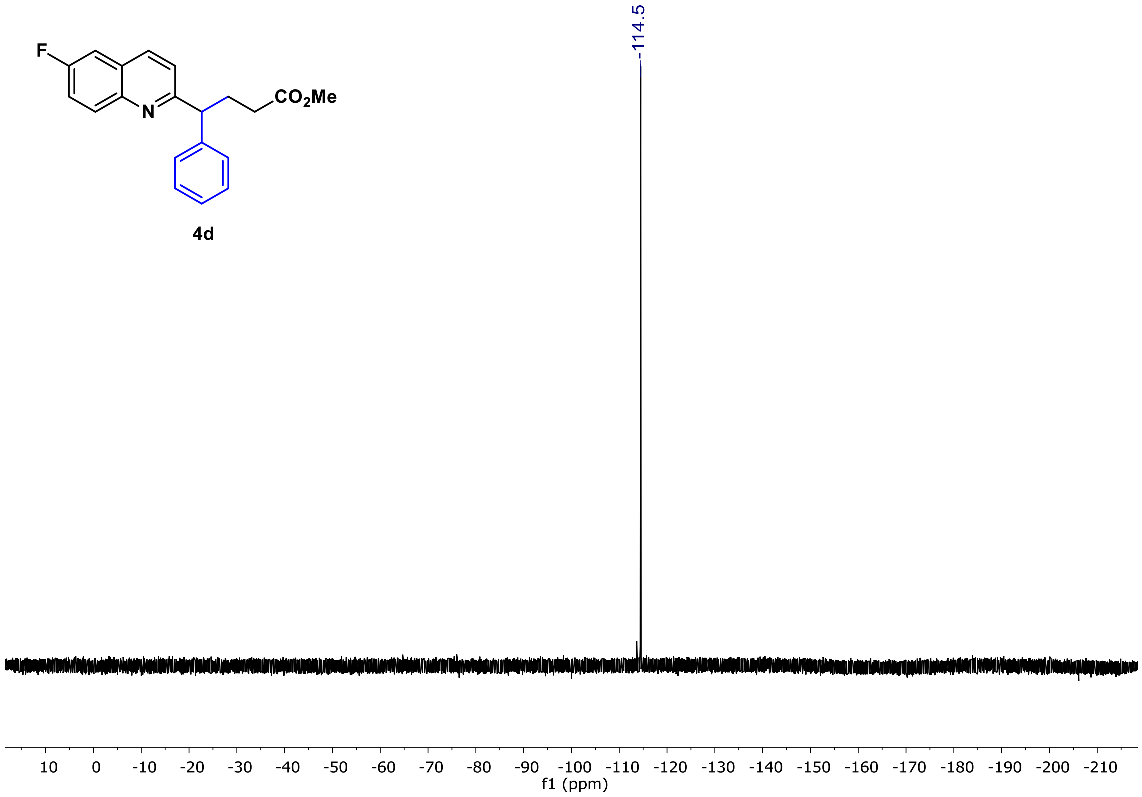

Supplementary Fig. 15. ^{19}F NMR Spectra of **4d**.

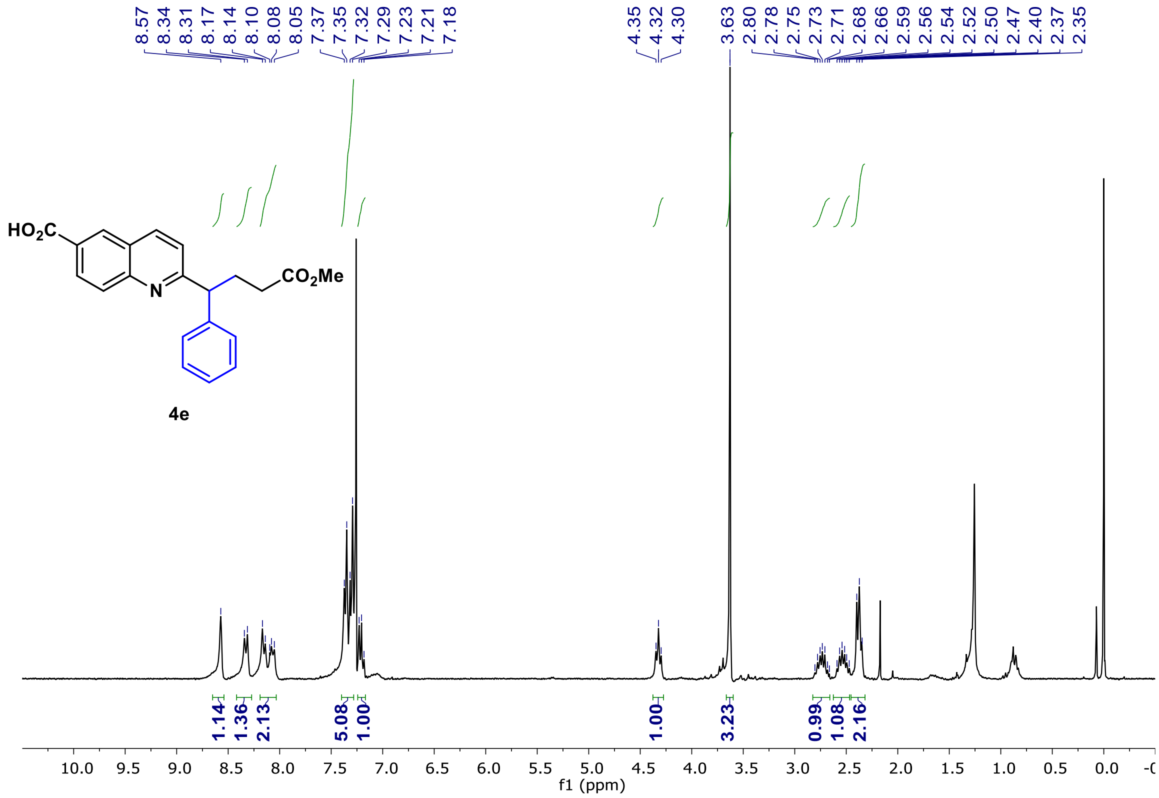

Supplementary Fig. 16. ^{1}H NMR Spectra of **4e**.

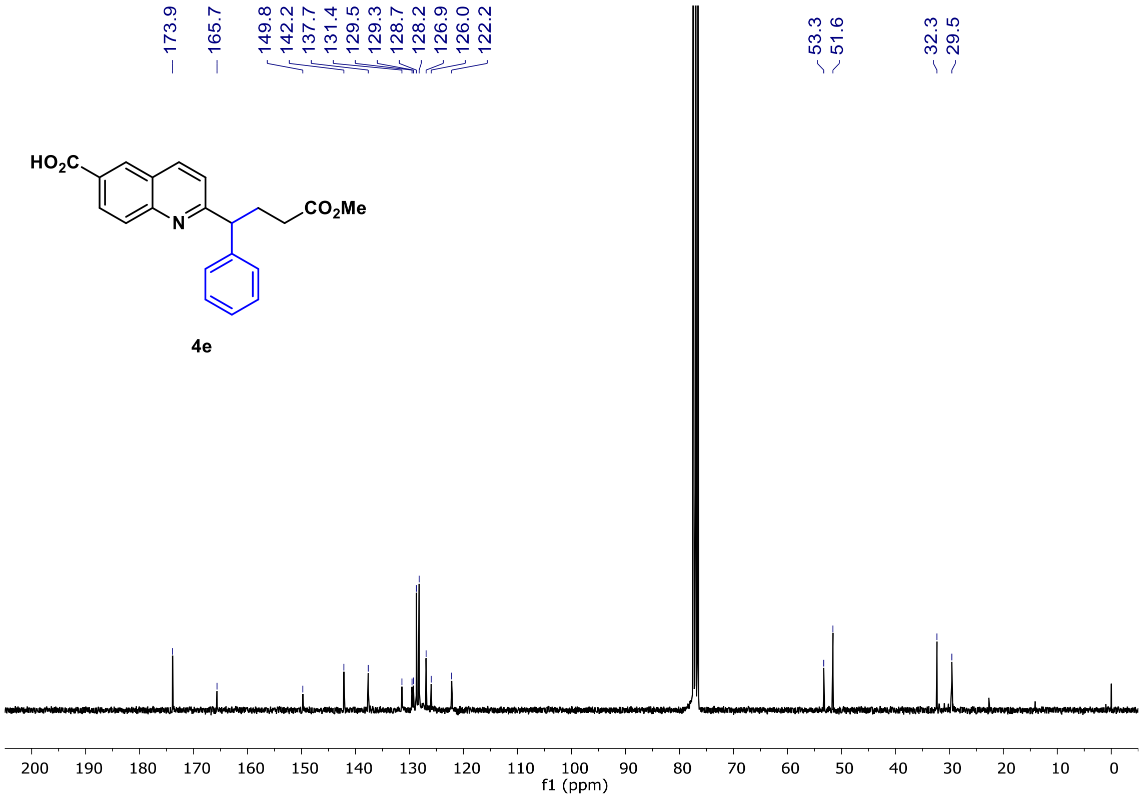

Supplementary Fig. 17. ^{13}C NMR Spectra of **4e**.

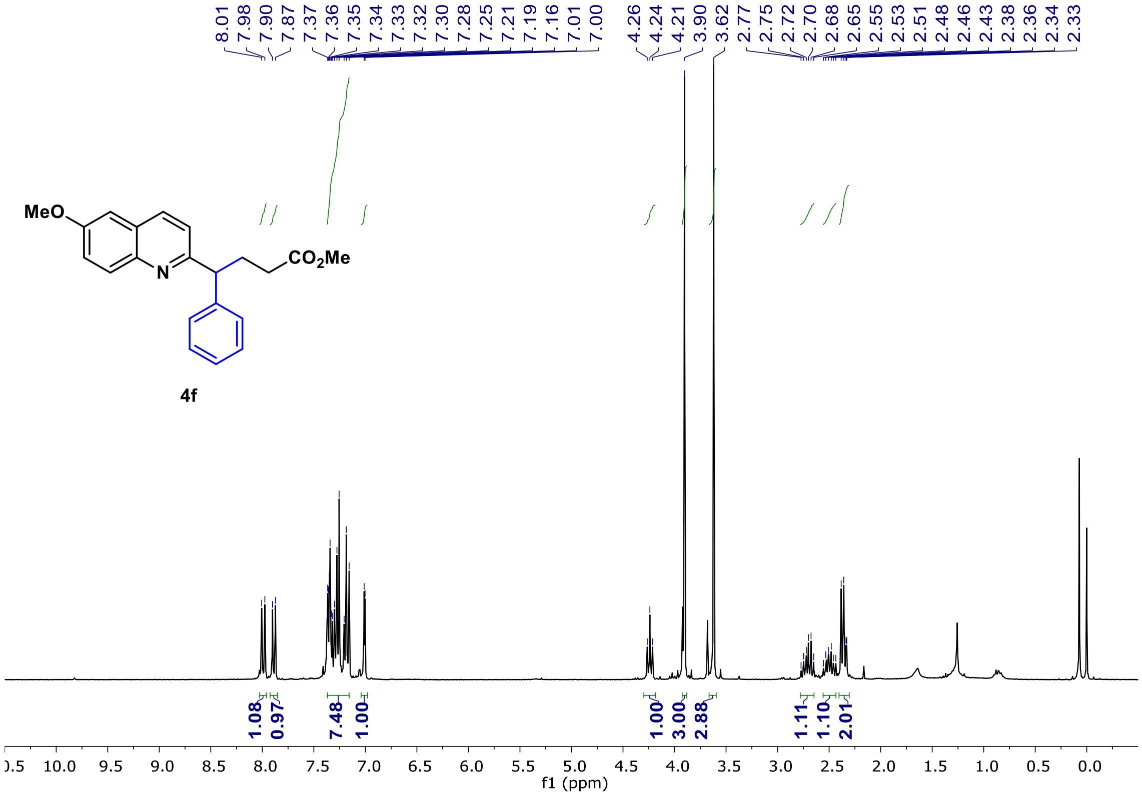

Supplementary Fig. 18. ^{1}H NMR Spectra of **4f**.

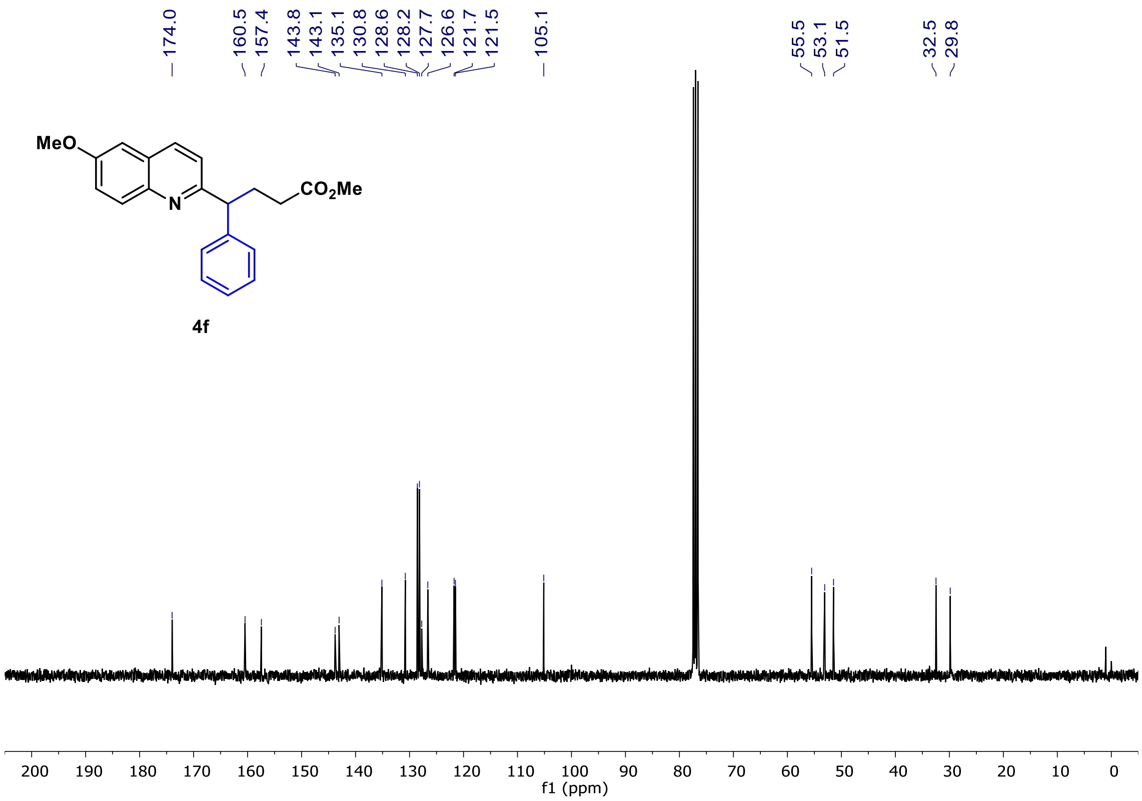

Supplementary Fig. 19. ^{13}C NMR Spectra of **4f**.

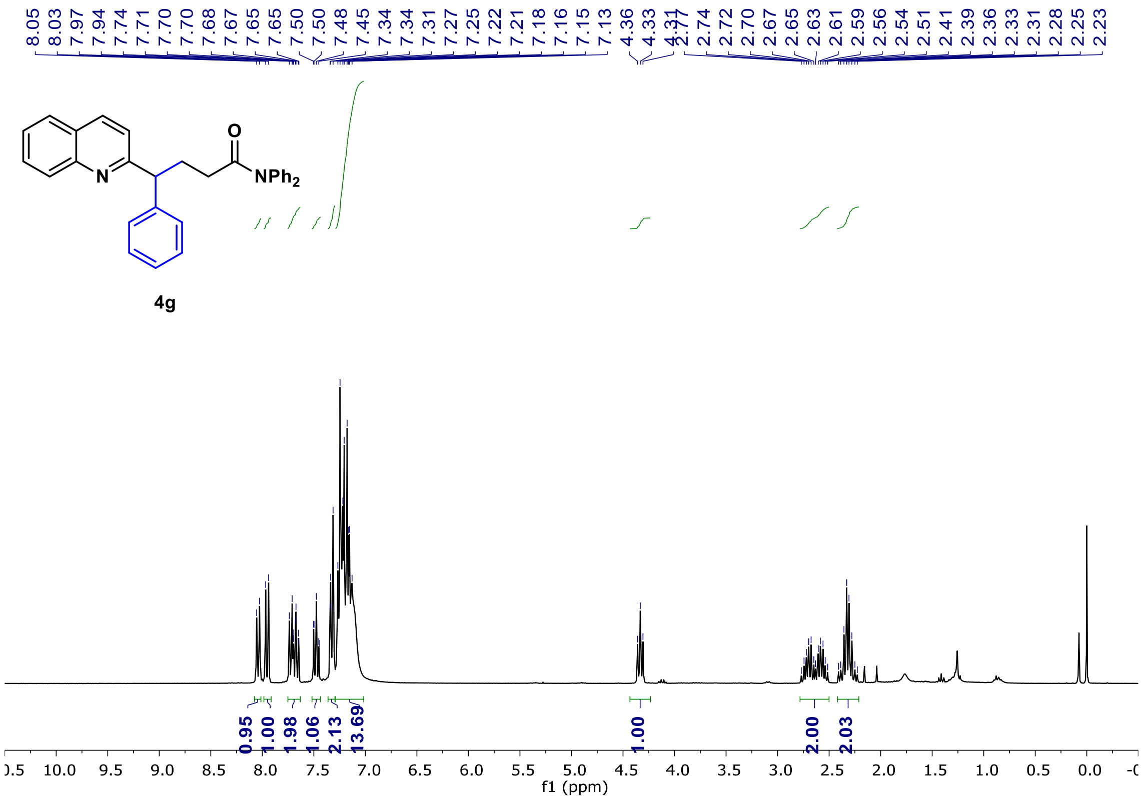

Supplementary Fig. 20. ^{1}H NMR Spectra of **4g**.

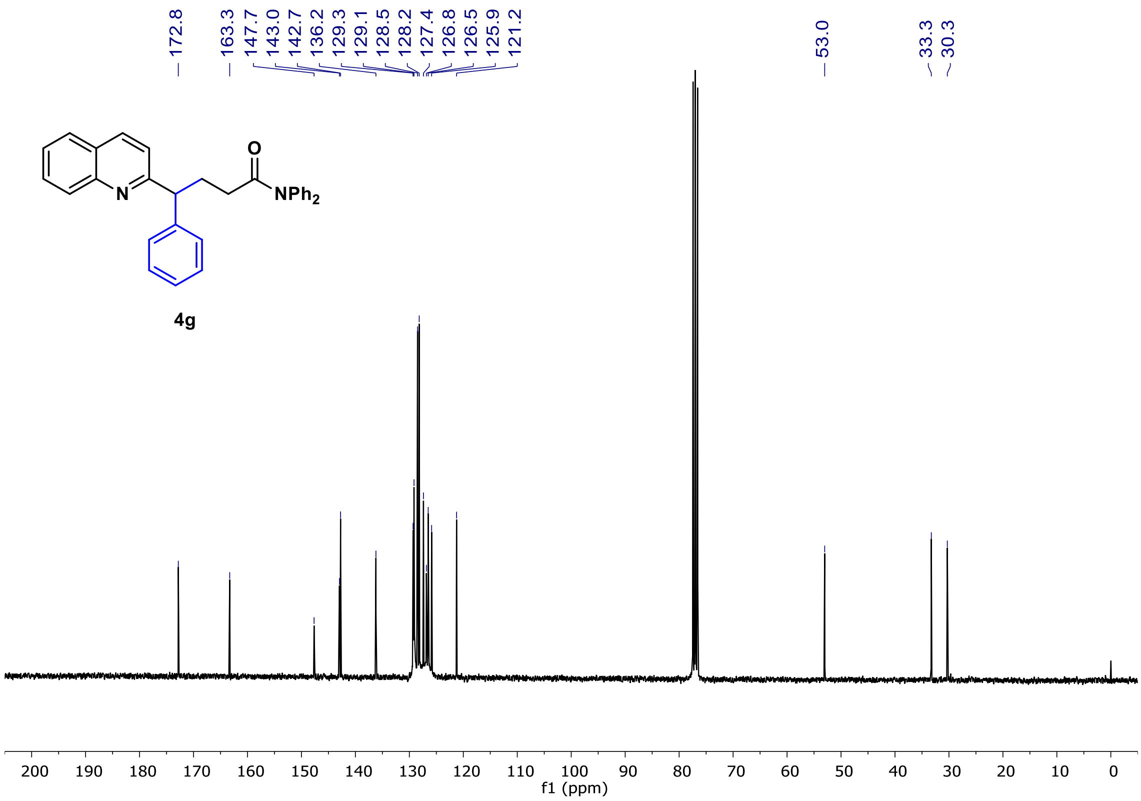

Supplementary Fig. 21. ^{13}C NMR Spectra of **4g**.

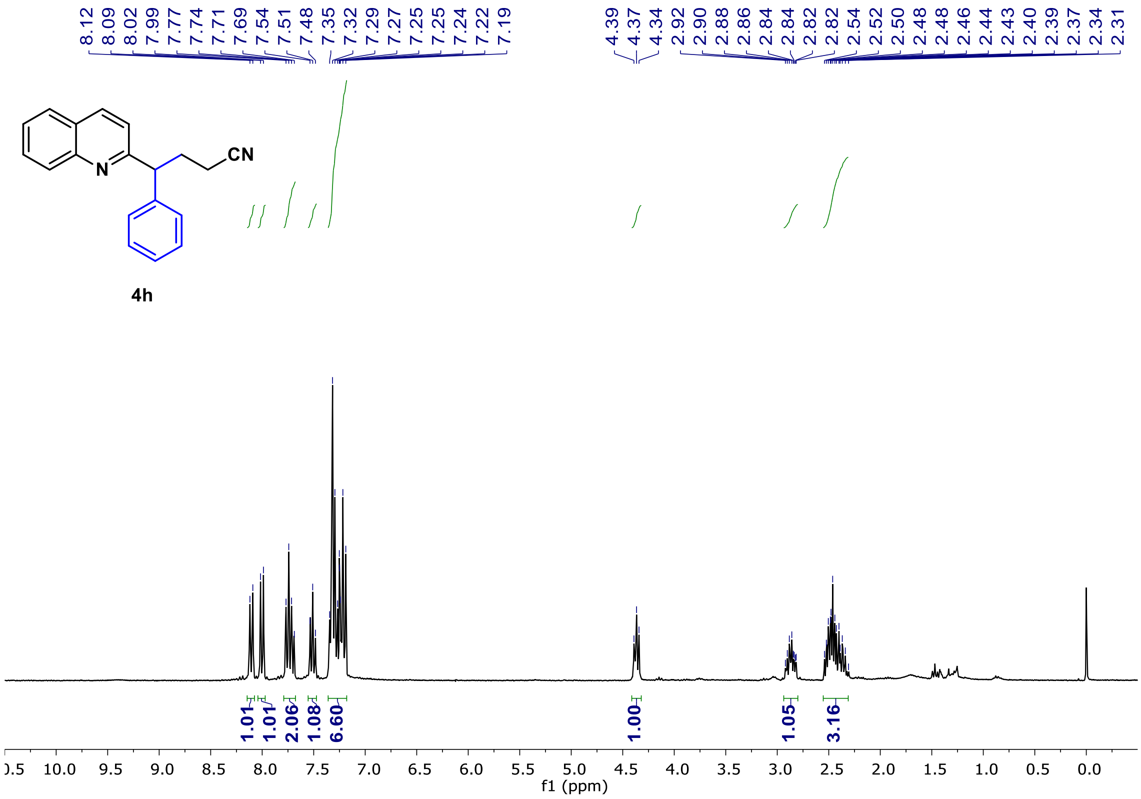

Supplementary Fig. 22. ^{1}H NMR Spectra of **4h**.

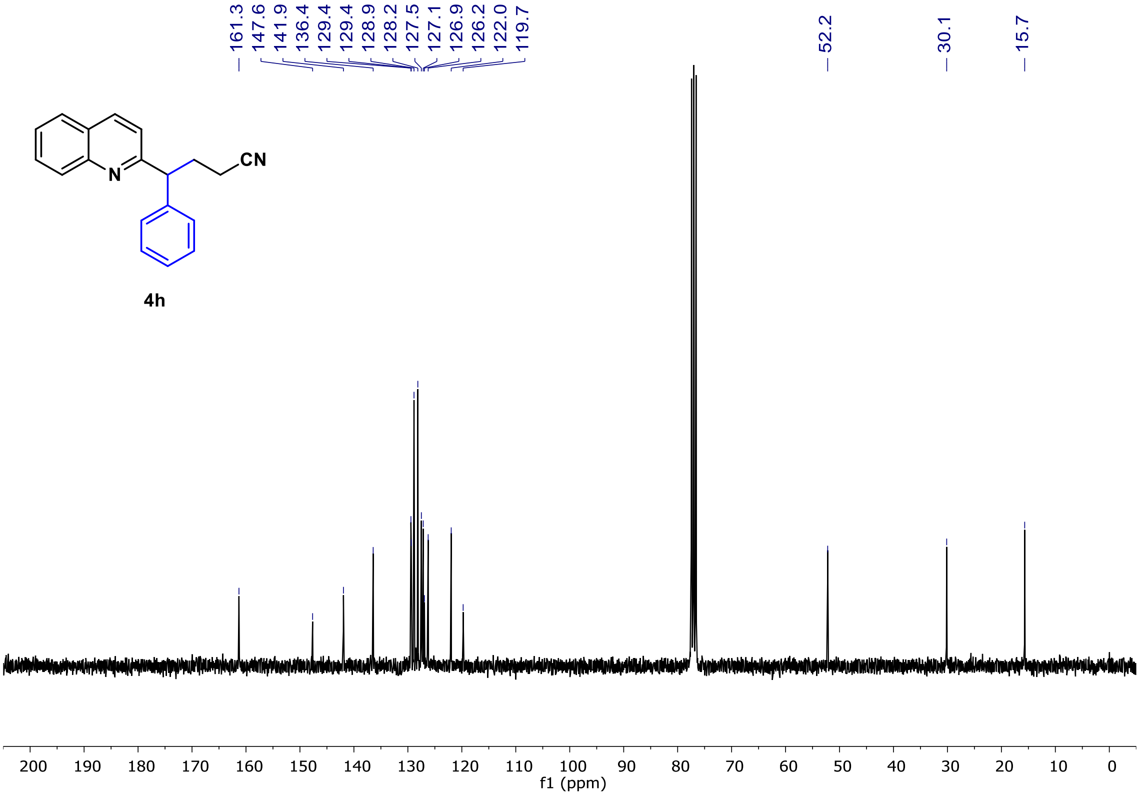

Supplementary Fig. 23. ^{13}C NMR Spectra of **4h**.

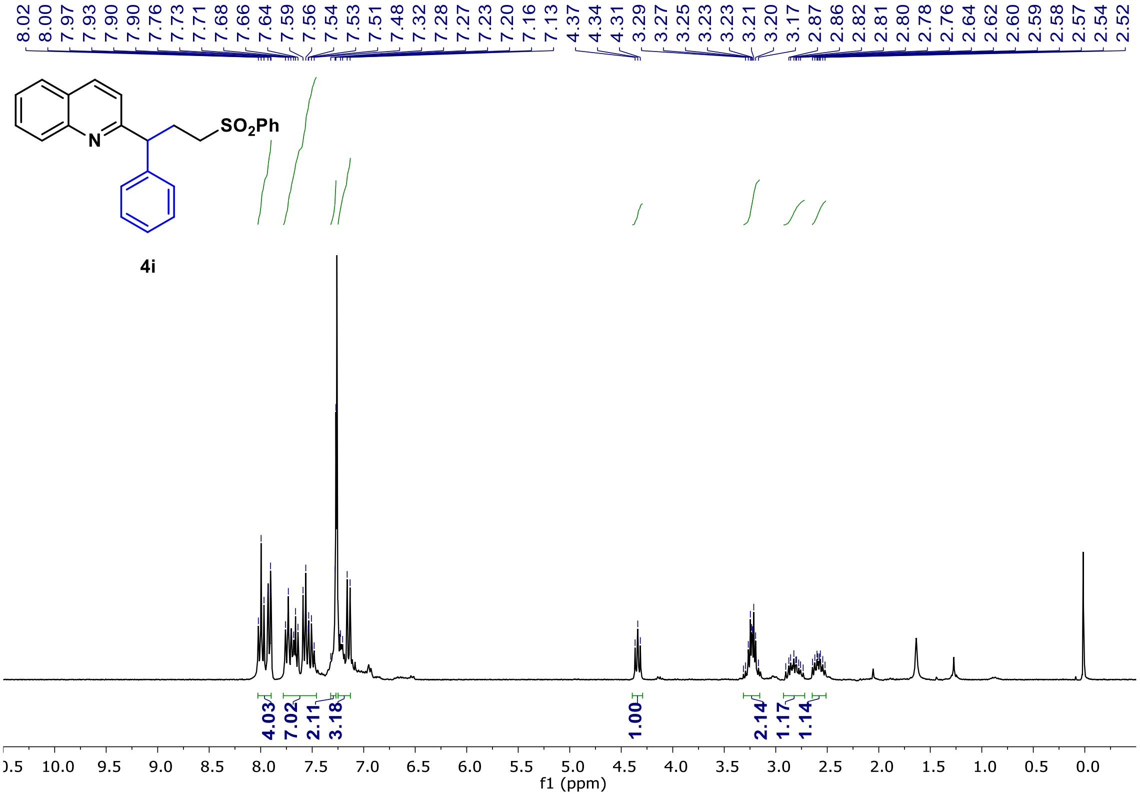

Supplementary Fig. 24. ^{1}H NMR Spectra of **4i**.

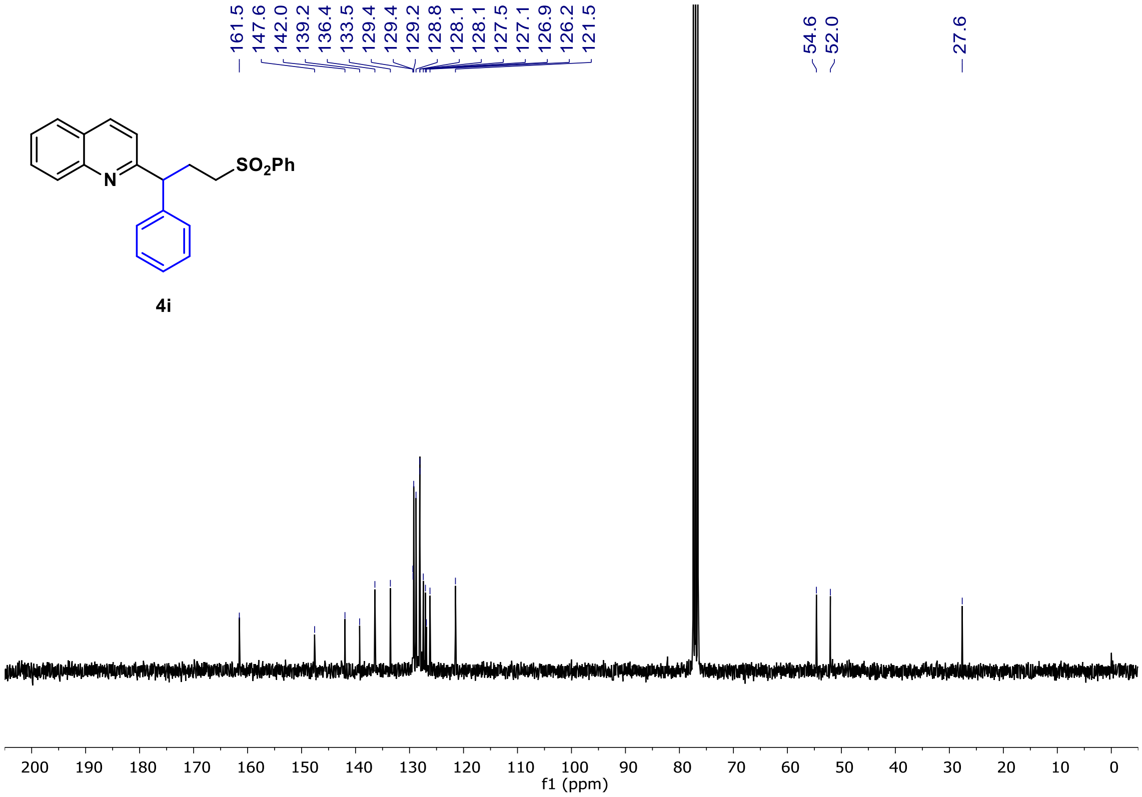

Supplementary Fig. 25. ^{13}C NMR Spectra of **4i**.

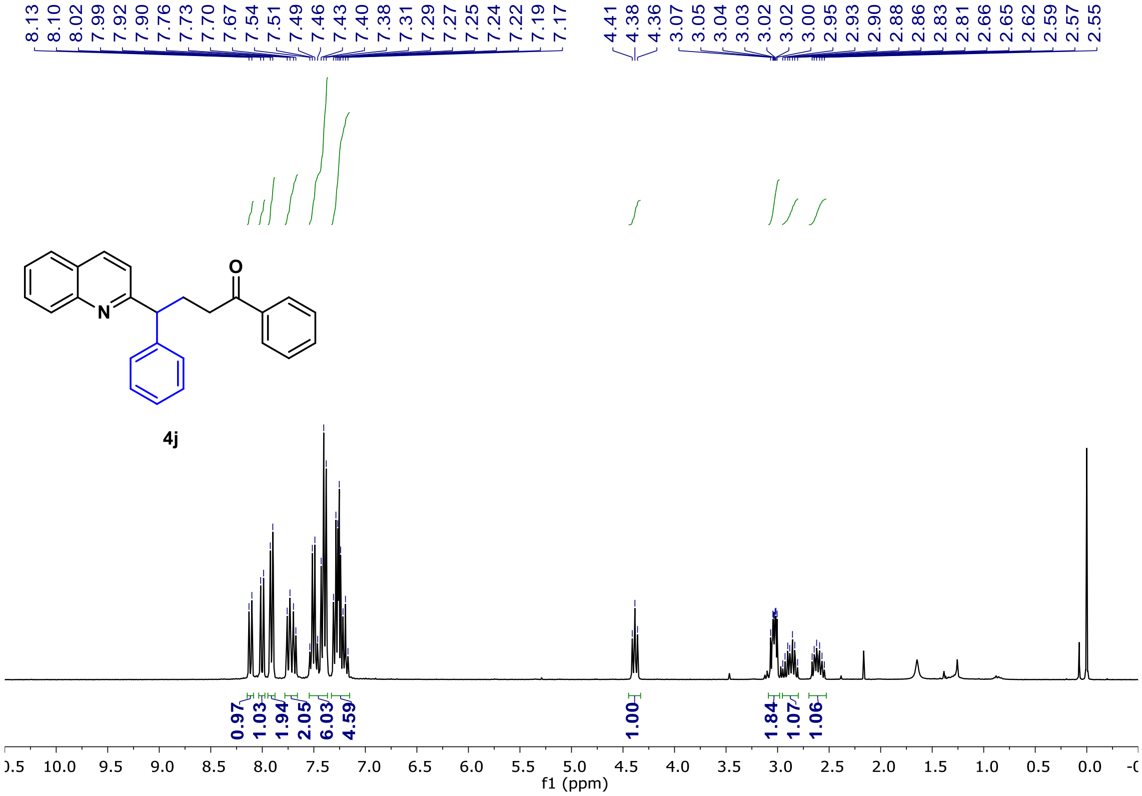

Supplementary Fig. 26. ^{1}H NMR Spectra of **4j**.

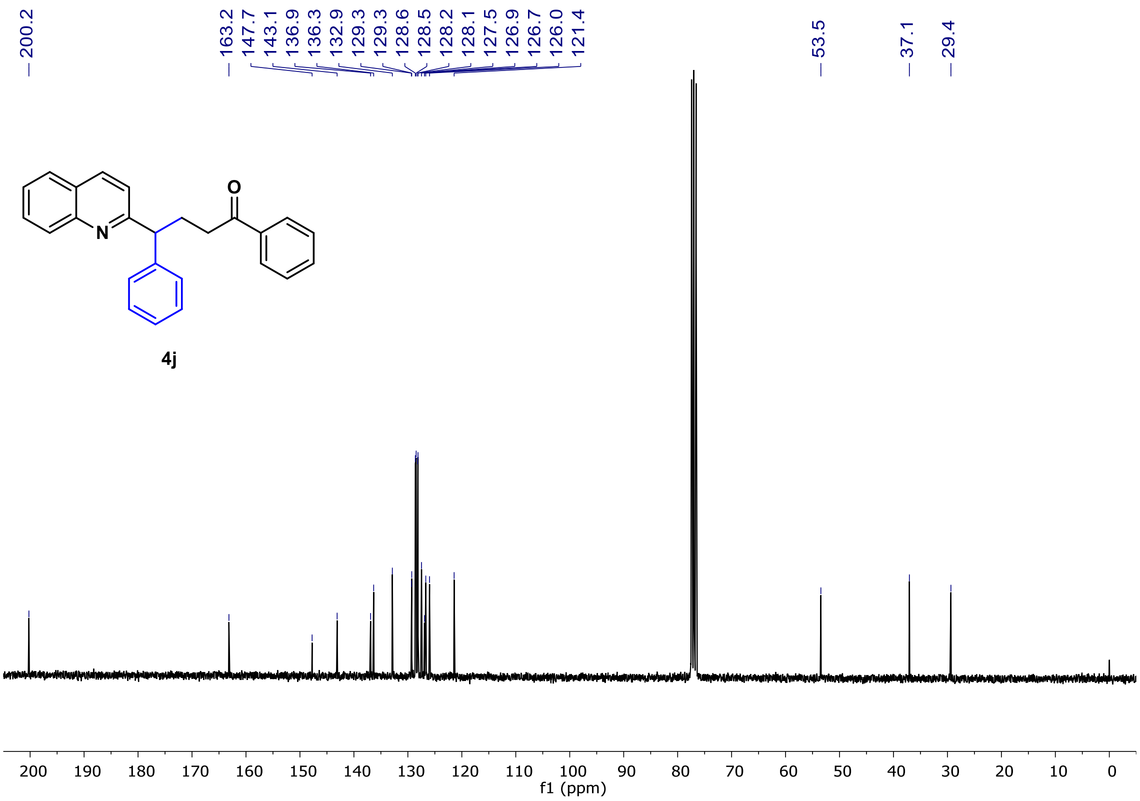

Supplementary Fig. 27. ^{13}C NMR Spectra of **4j**.

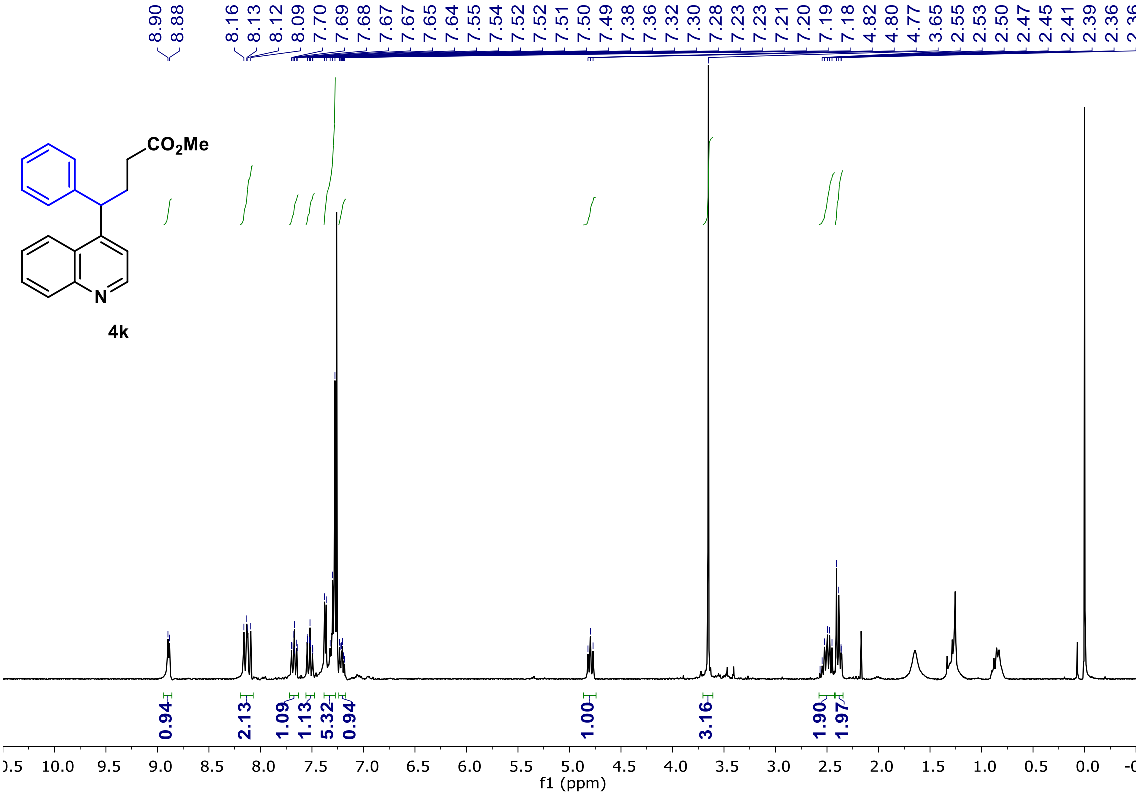

Supplementary Fig. 28. ^{1}H NMR Spectra of **4k**.

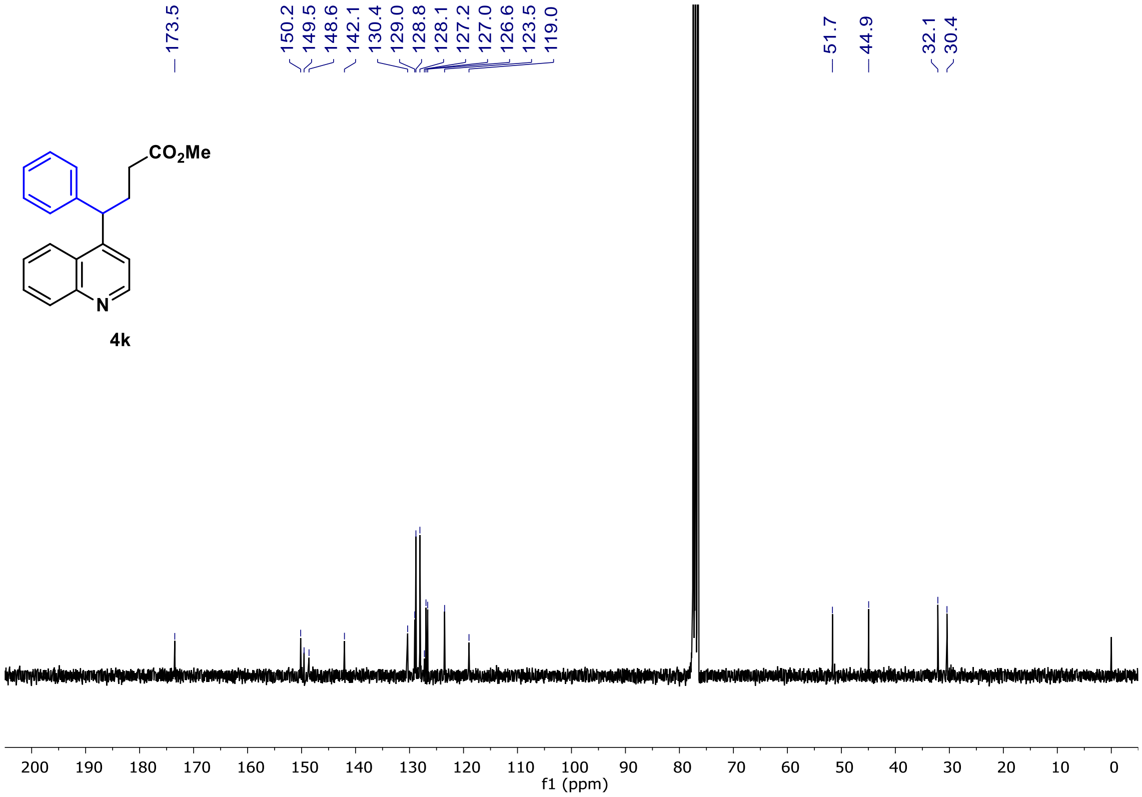

Supplementary Fig. 29. ^{13}C NMR Spectra of **4k**.

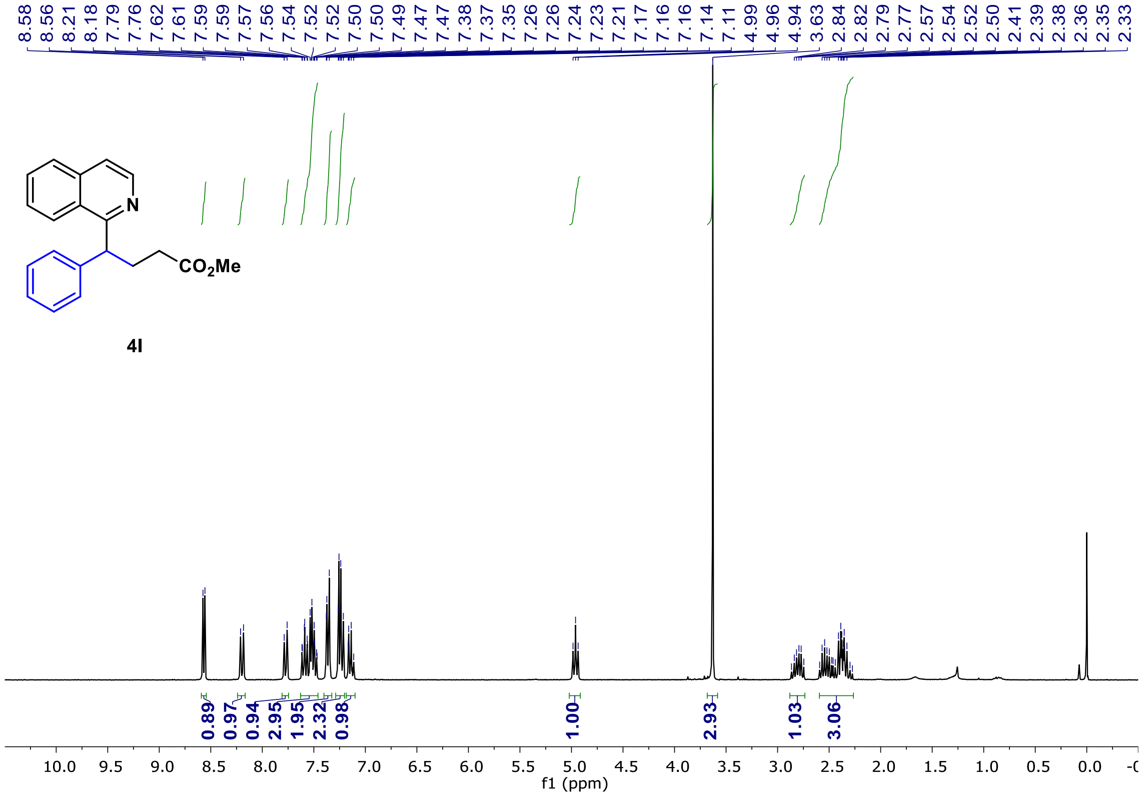

Supplementary Fig. 30. ^{1}H NMR Spectra of **4l**.

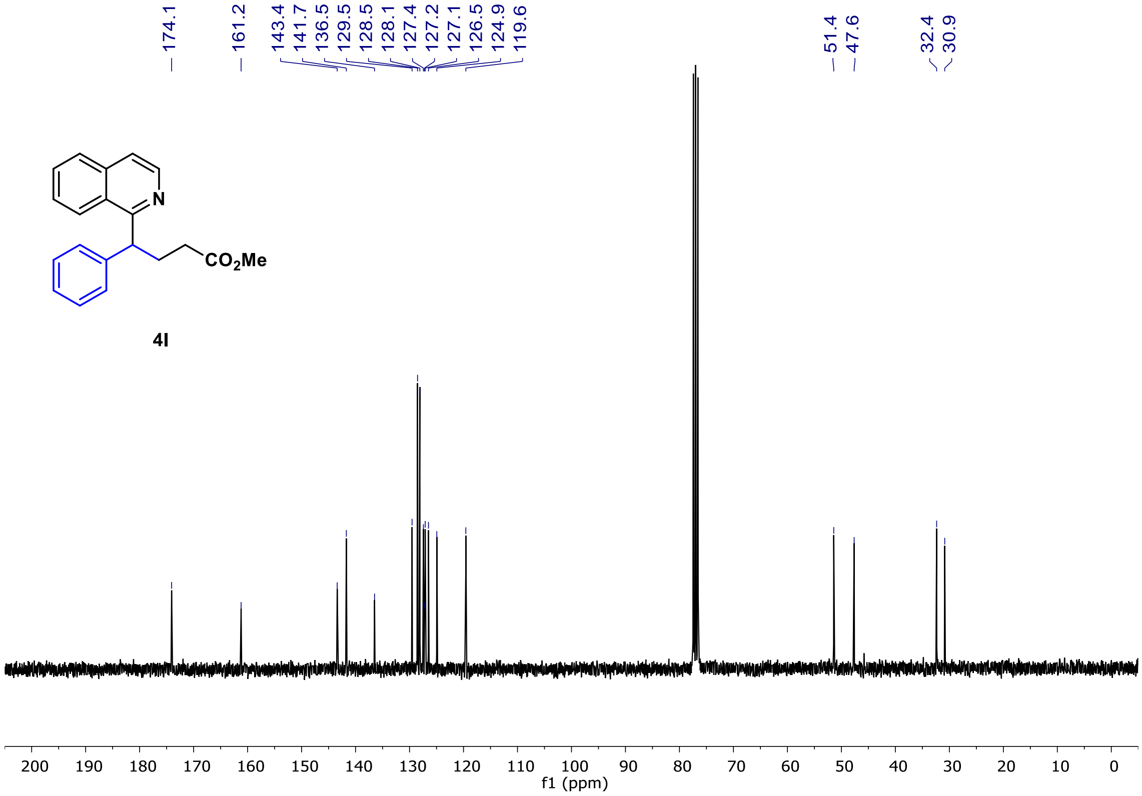

Supplementary Fig. 31. ^{13}C NMR Spectra of **4l**.

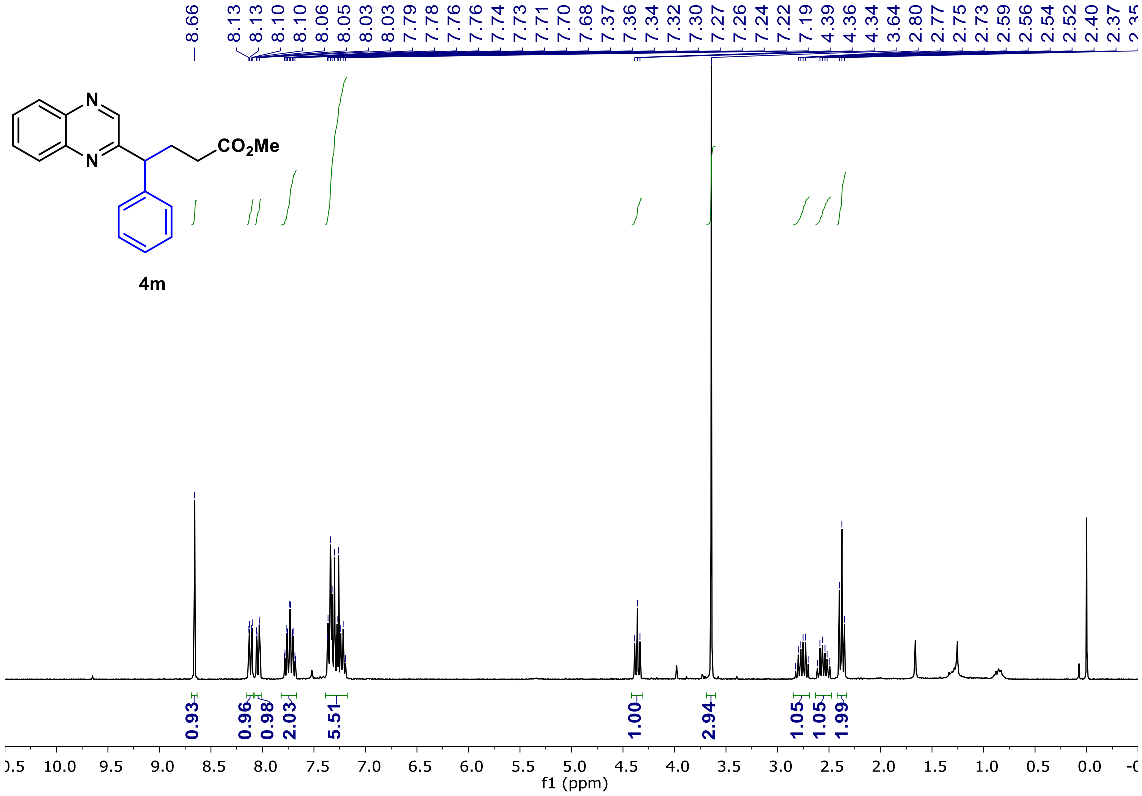

Supplementary Fig. 32. ^{1}H NMR Spectra of **4m**.

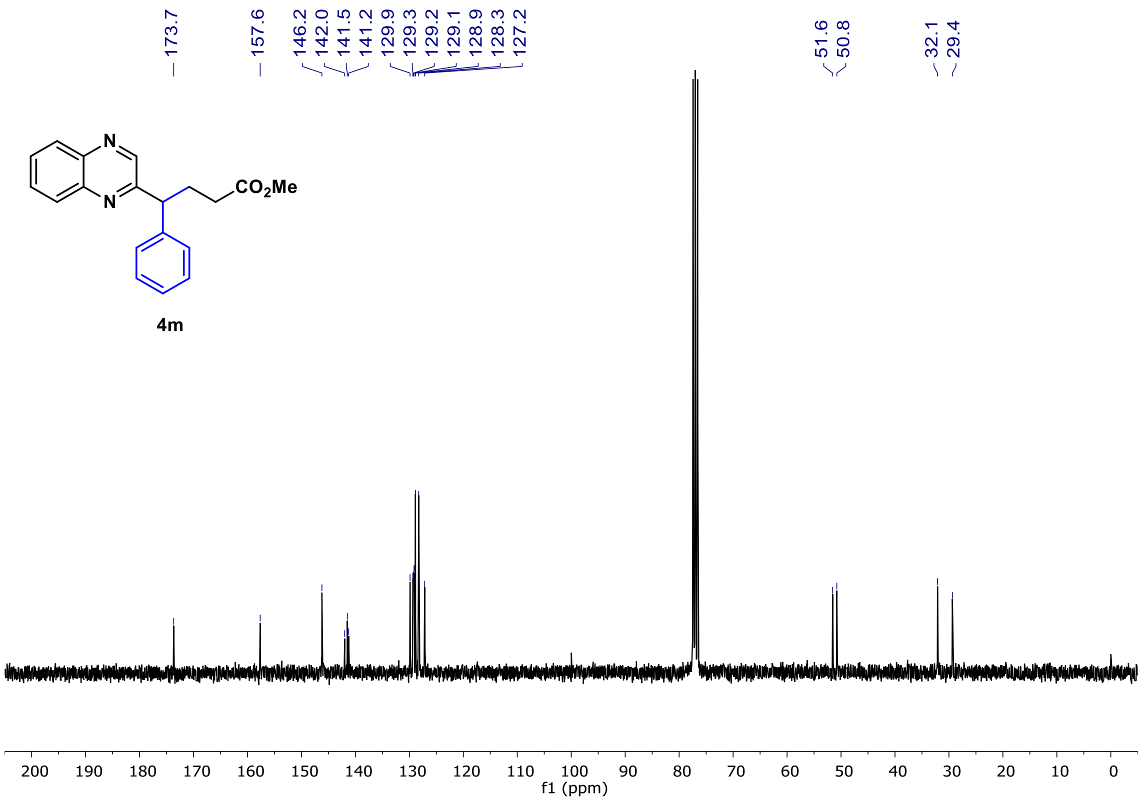

Supplementary Fig. 33. ^{13}C NMR Spectra of **4m**.

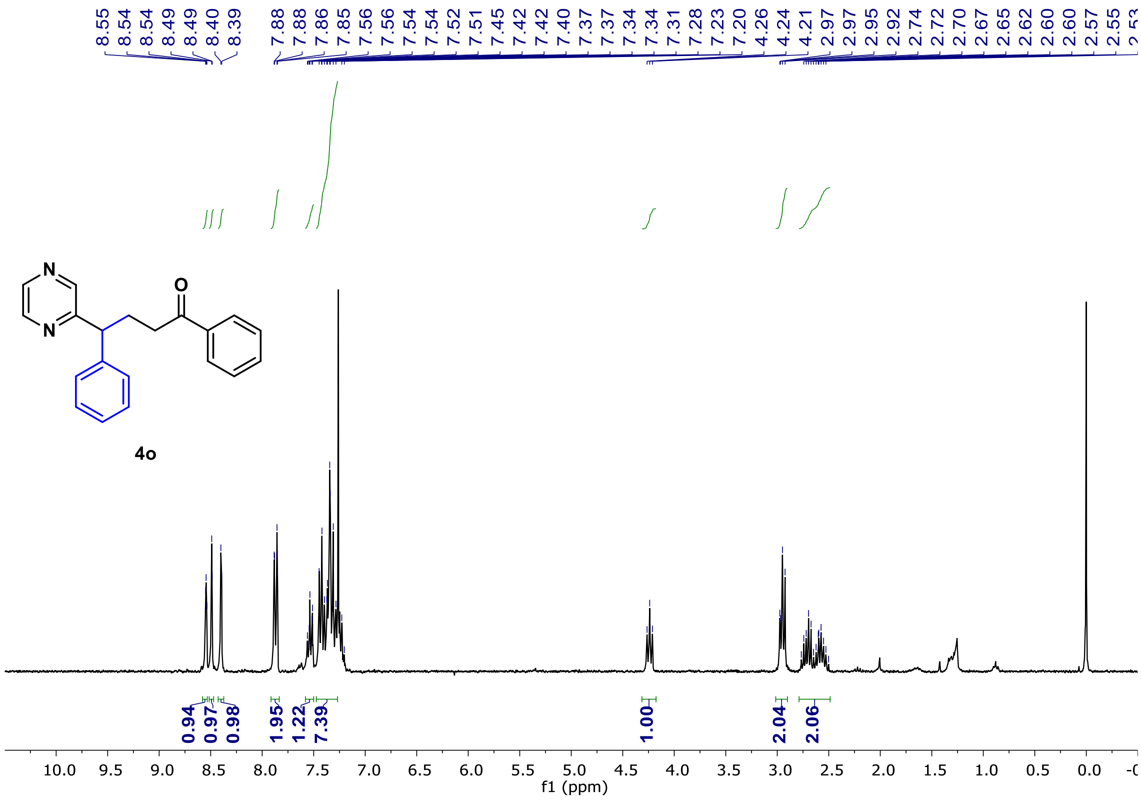

Supplementary Fig. 34. ^{1}H NMR Spectra of **4o**.

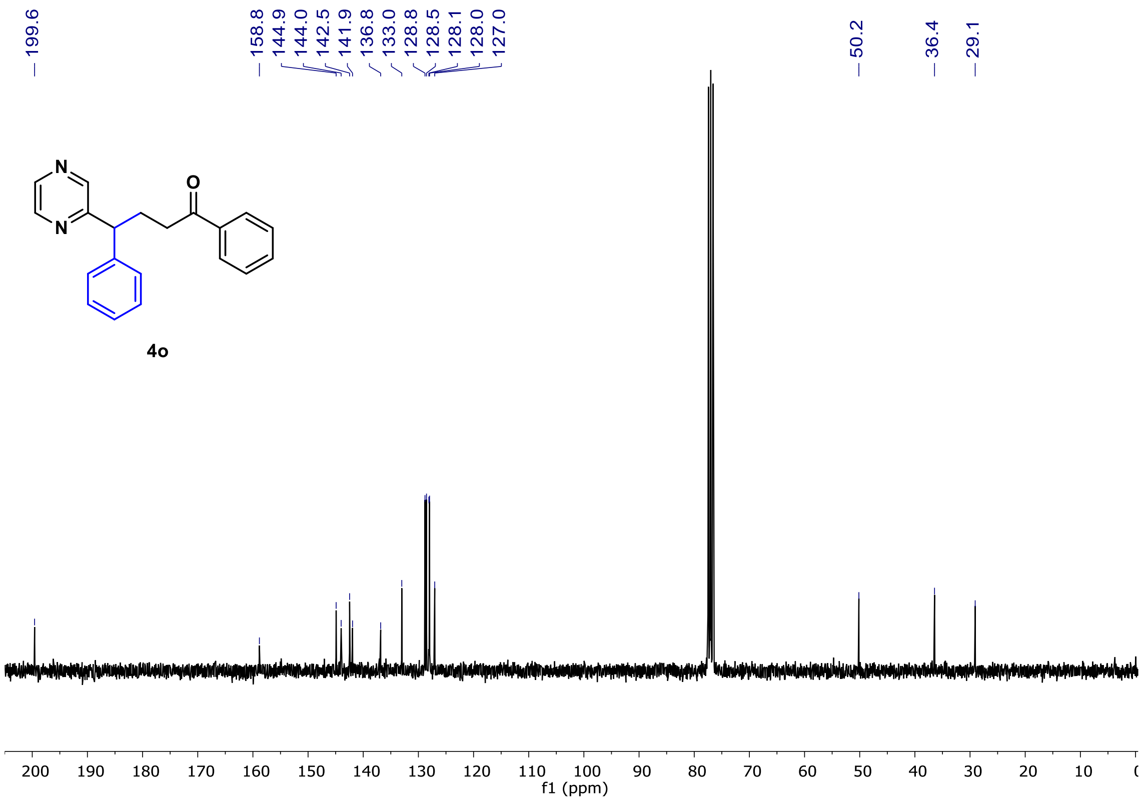

Supplementary Fig. 35. ^{13}C NMR Spectra of **4o**.

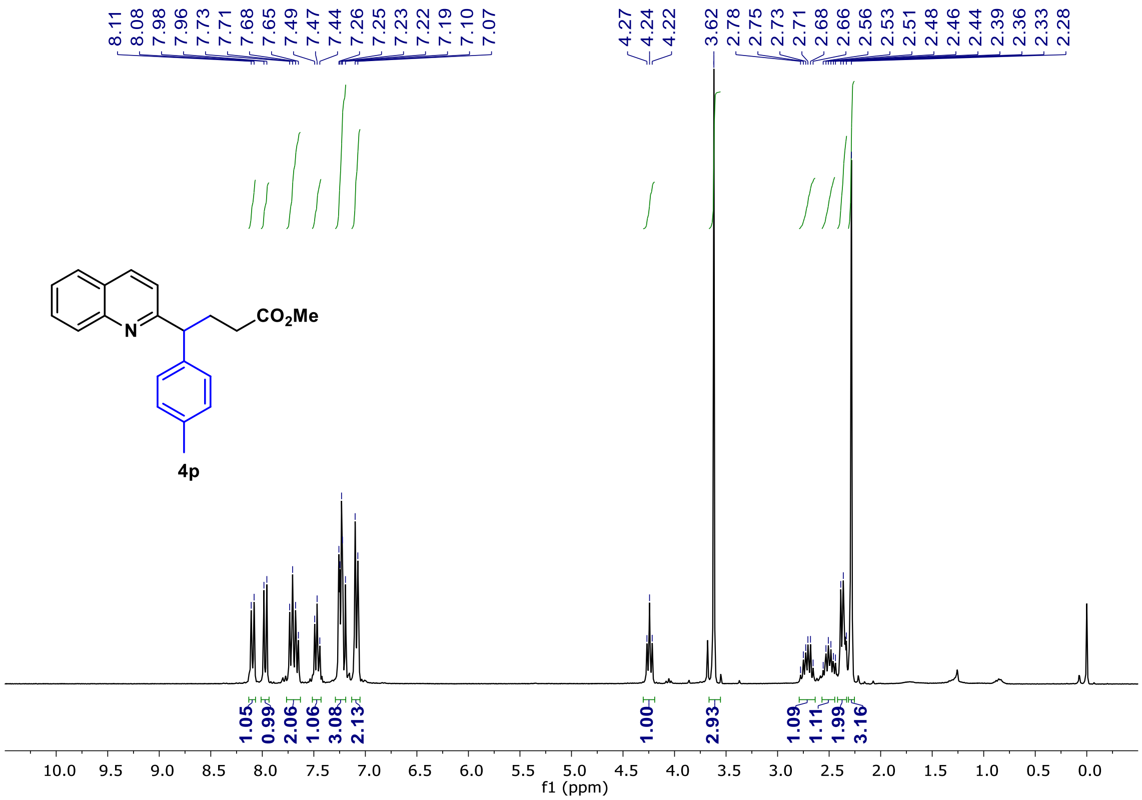

Supplementary Fig. 36. ^{1}H NMR Spectra of **4p**.

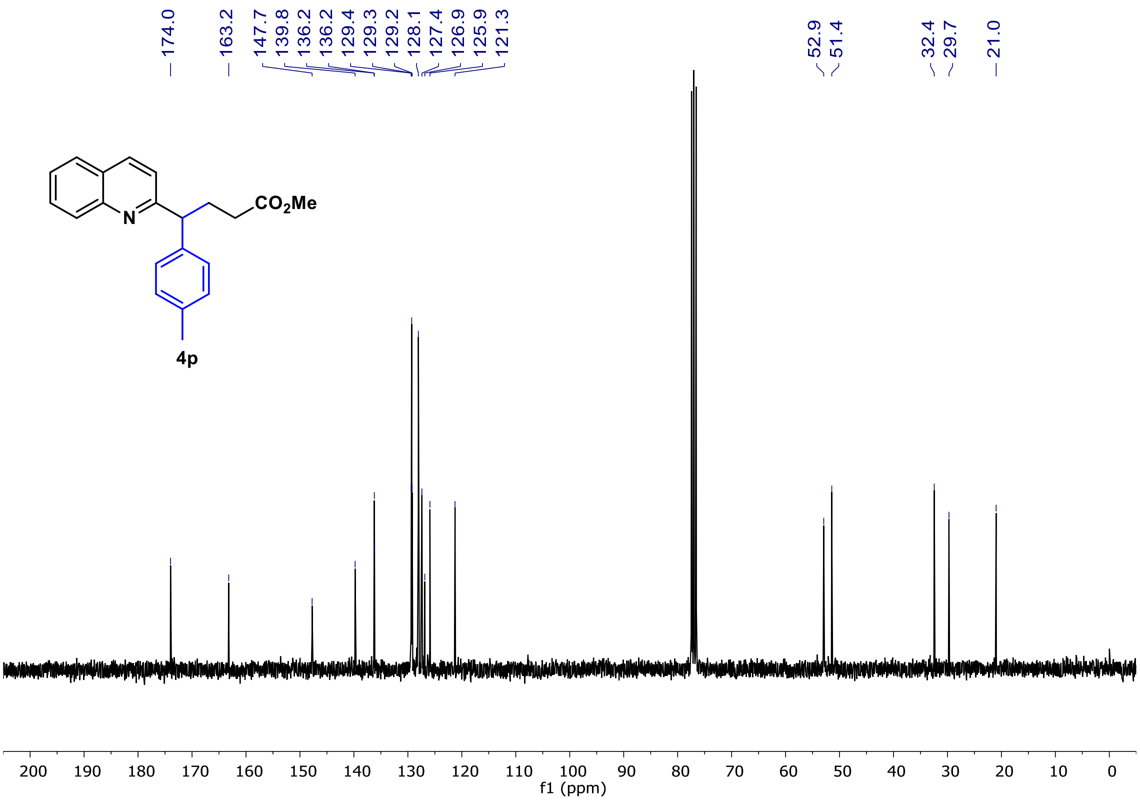

Supplementary Fig. 37. ^{13}C NMR Spectra of **4p**.

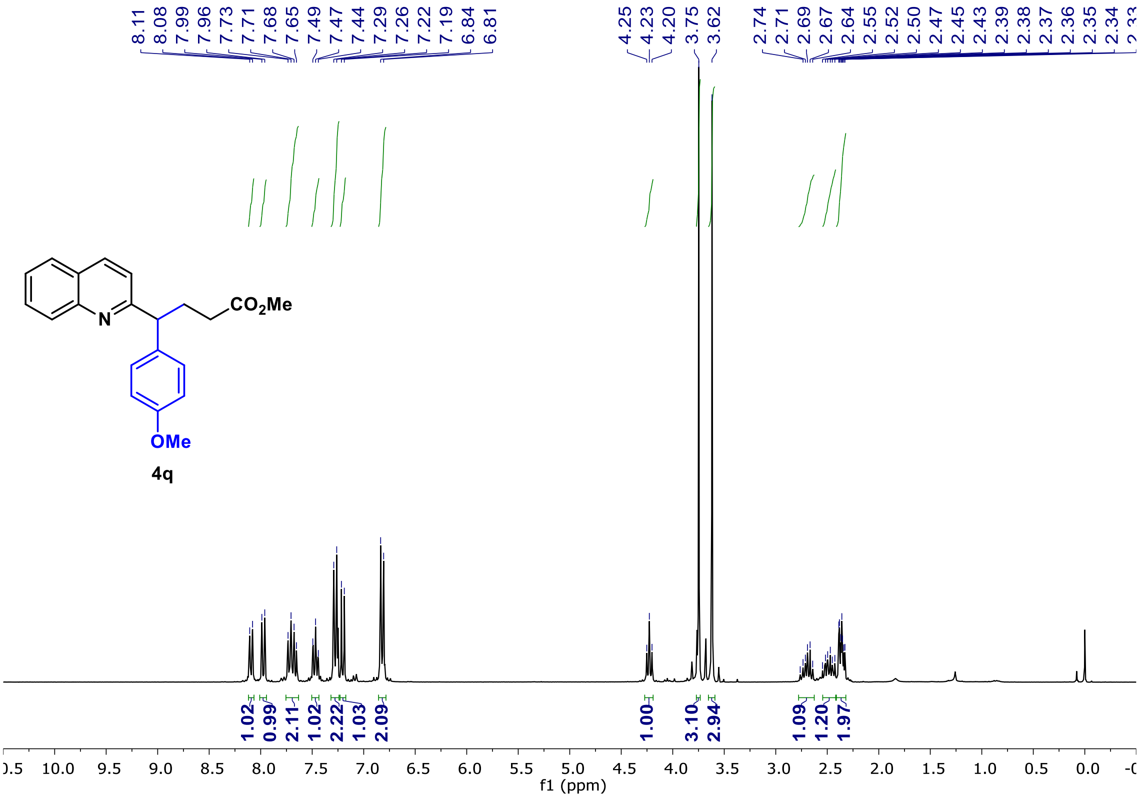

Supplementary Fig. 38. ^{1}H NMR Spectra of **4q**.

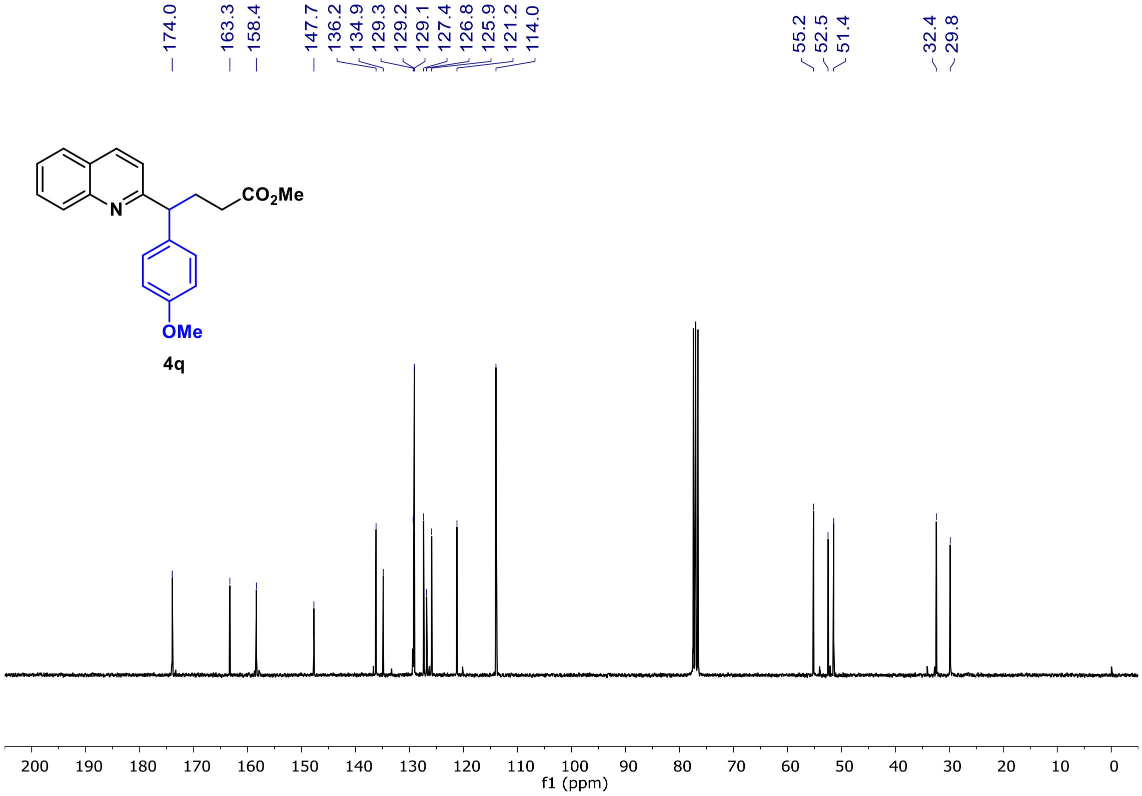

Supplementary Fig. 39. ^{13}C NMR Spectra of **4q**.

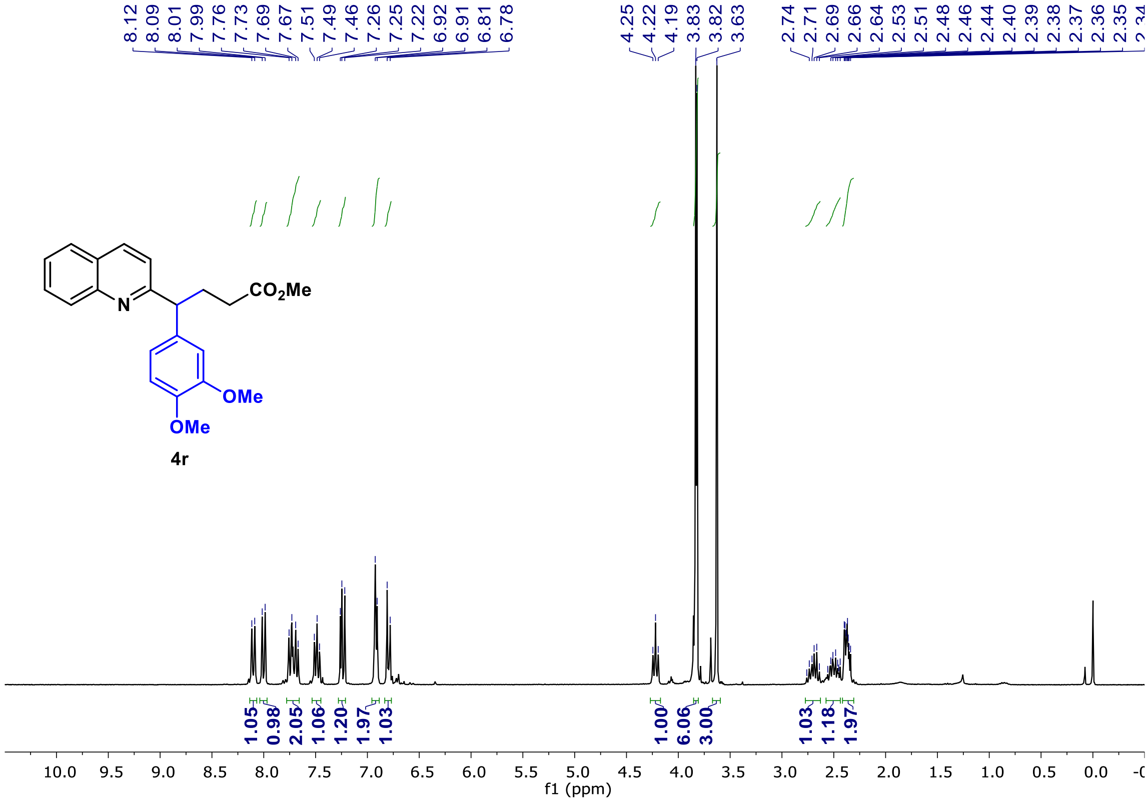

Supplementary Fig. 40. ^{1}H NMR Spectra of **4r**.

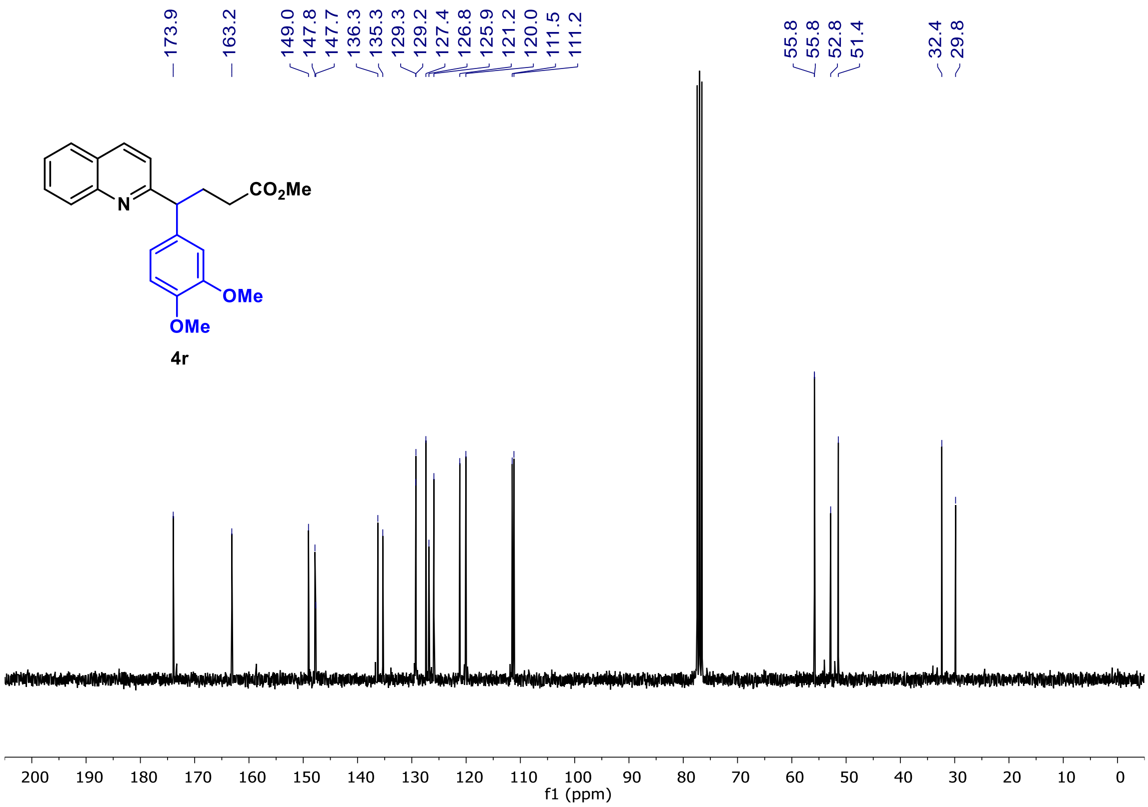

Supplementary Fig. 41. ^{13}C NMR Spectra of **4r**.

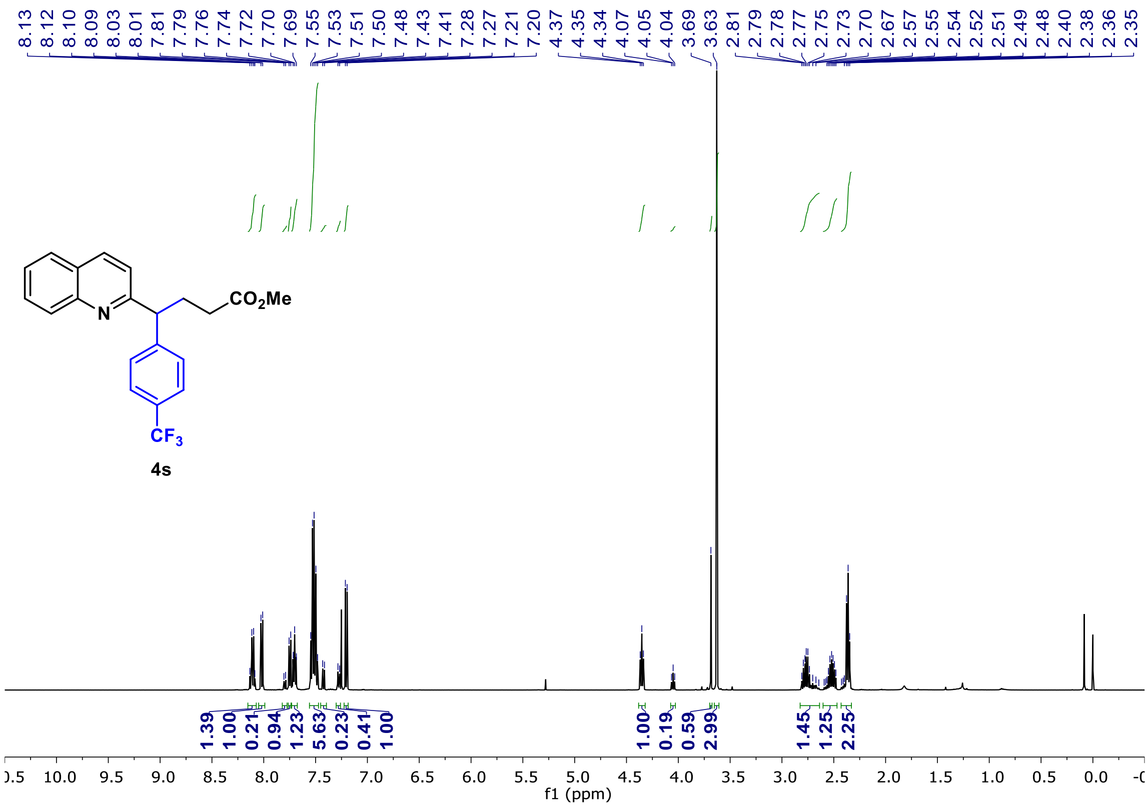

Supplementary Fig. 42. ^{1}H NMR Spectra of **4s**.

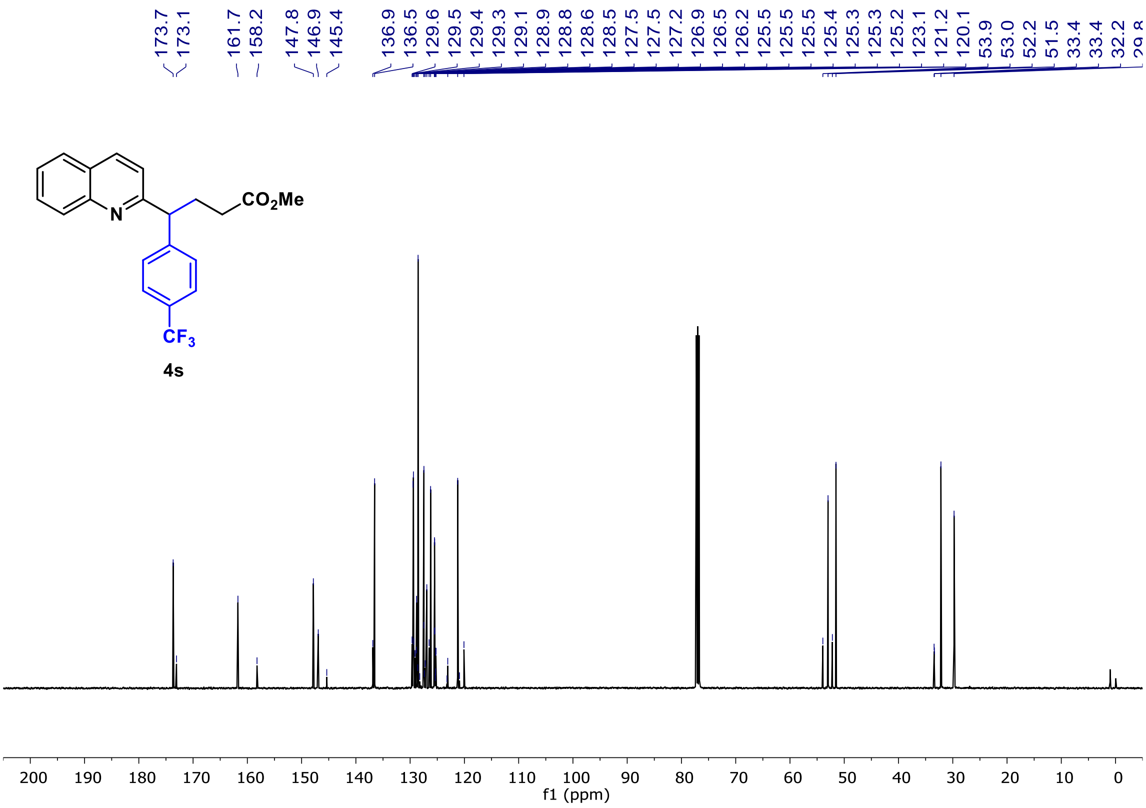

Supplementary Fig. 43. ^{13}C NMR Spectra of **4s**.

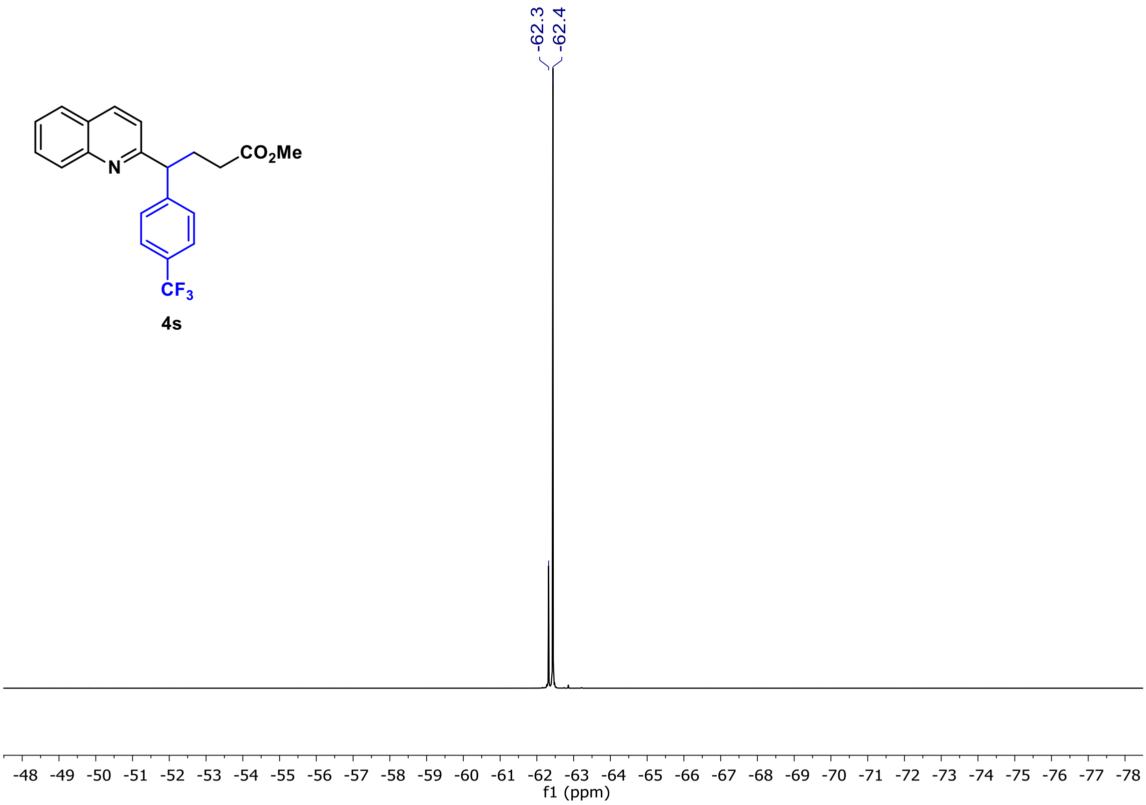

Supplementary Fig. 44. ^{19}F NMR Spectra of **4s**.

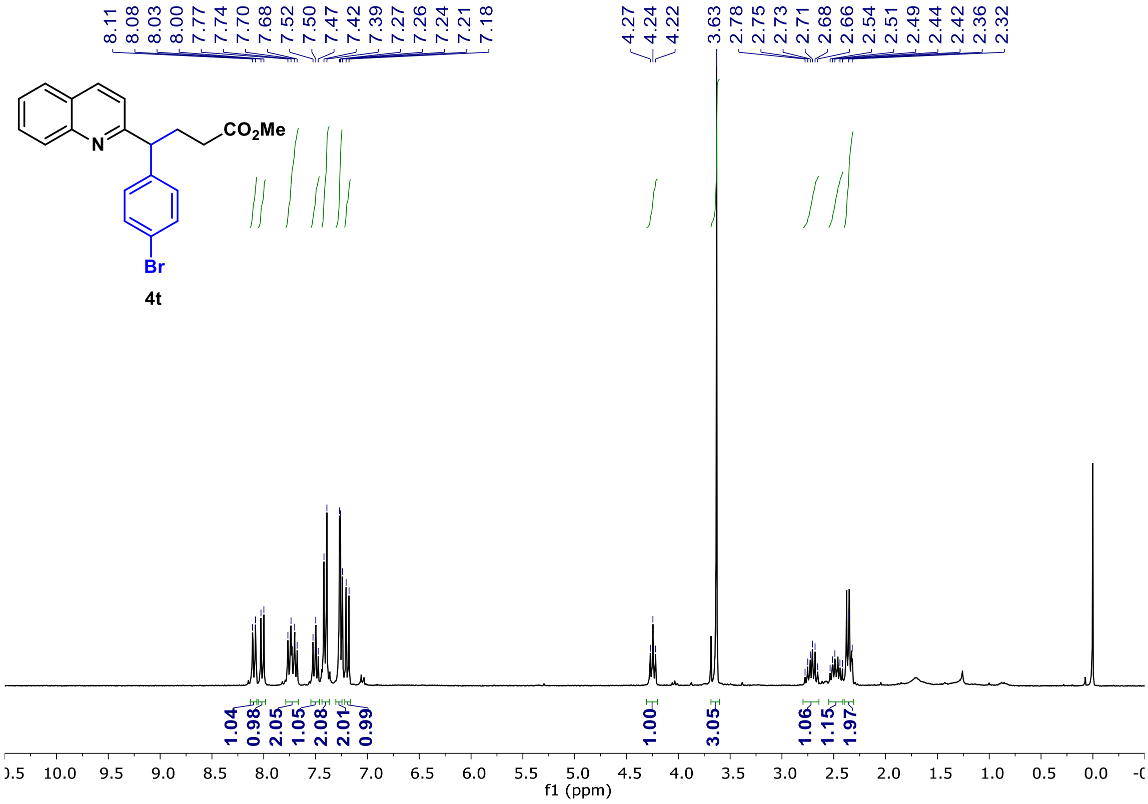

Supplementary Fig. 45. ^{1}H NMR Spectra of **4t**.

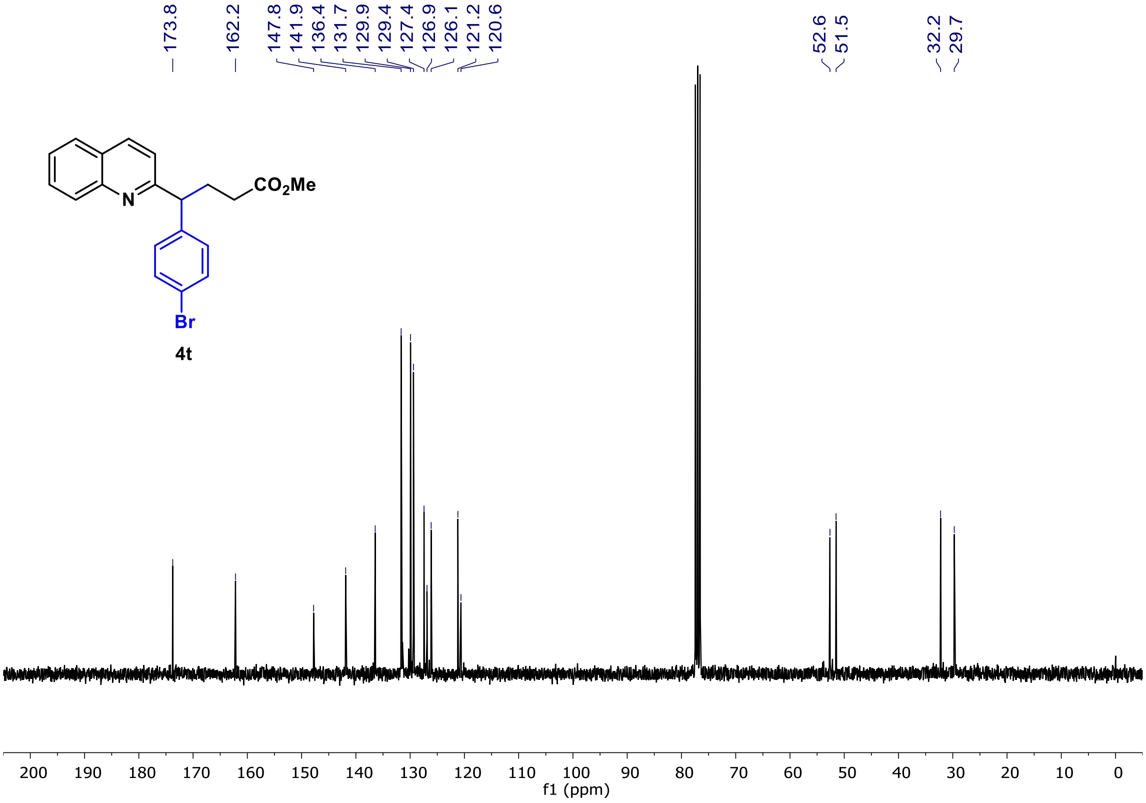

Supplementary Fig. 46. ^{13}C NMR Spectra of **4t**.

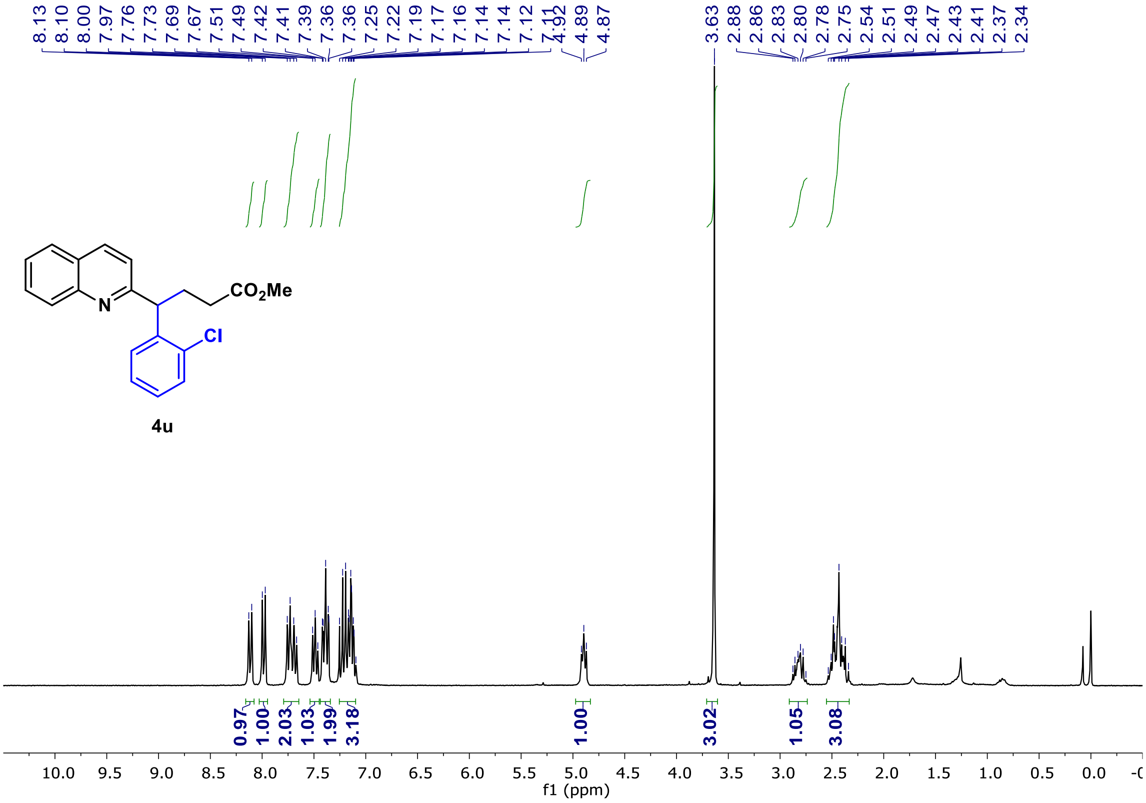

Supplementary Fig. 47. ^{1}H NMR Spectra of **4u**.

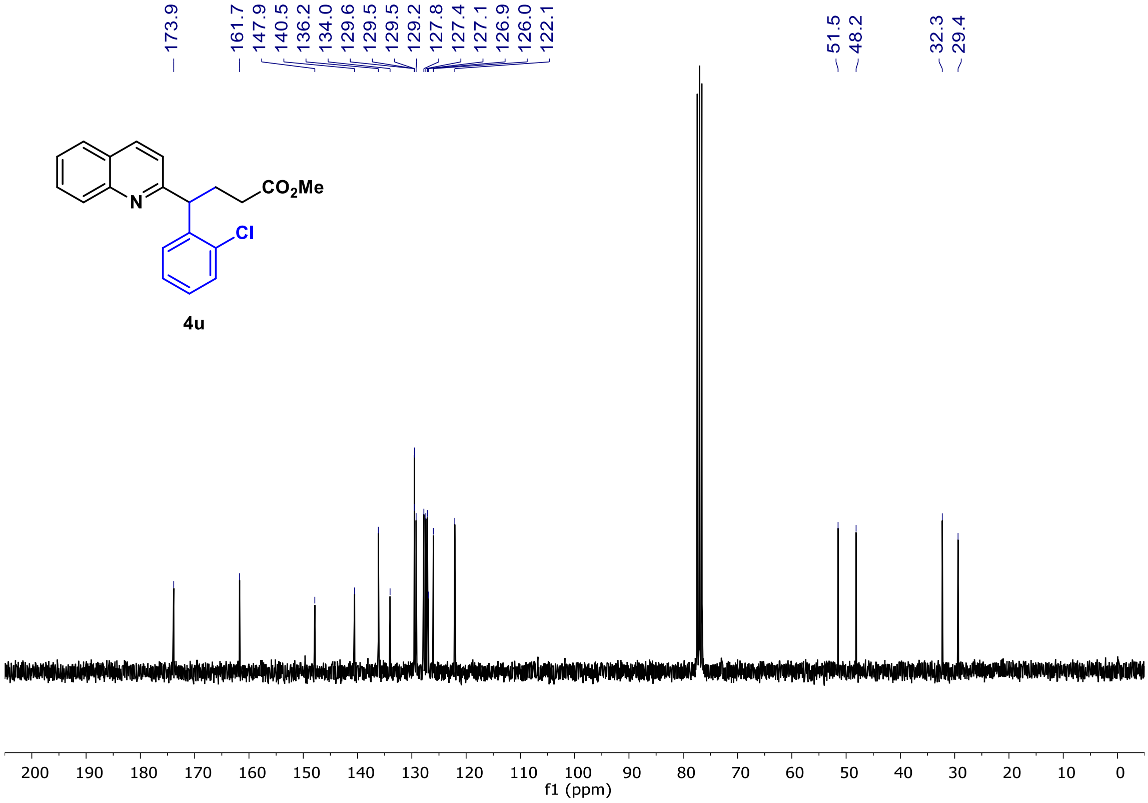

Supplementary Fig. 48. ^{13}C NMR Spectra of **4u**.

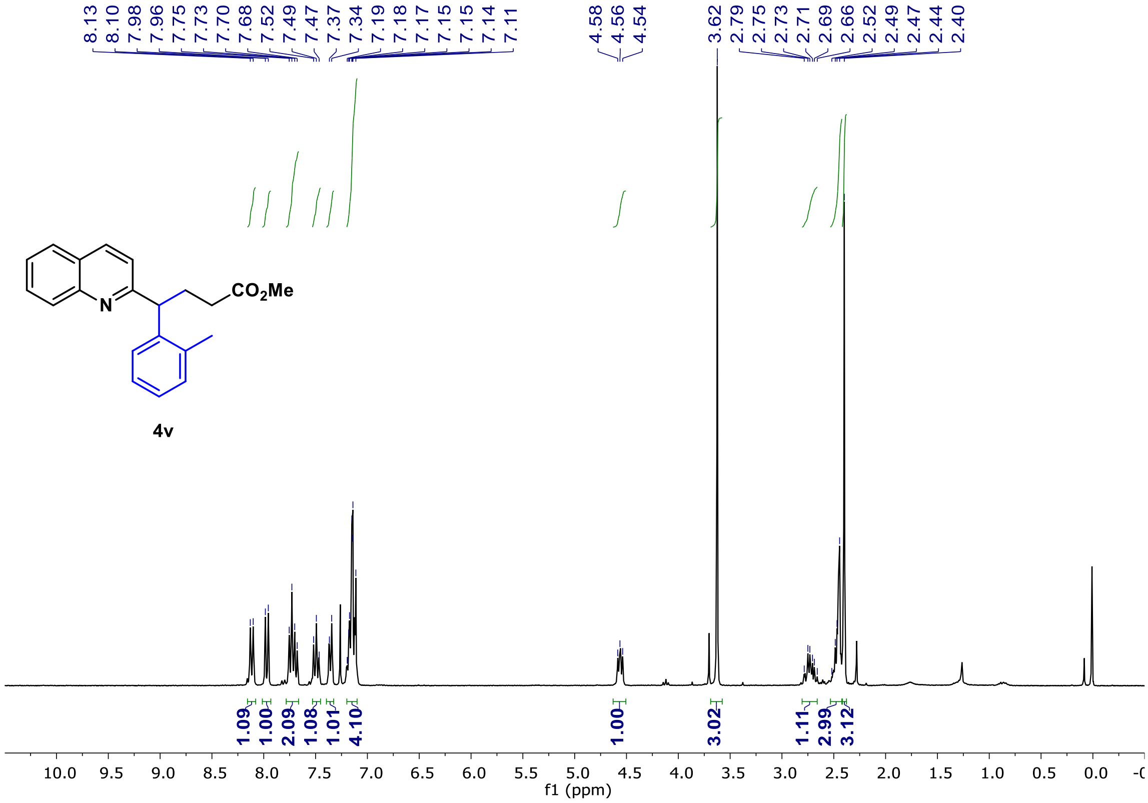

Supplementary Fig. 49. ^{1}H NMR Spectra of **4v**.

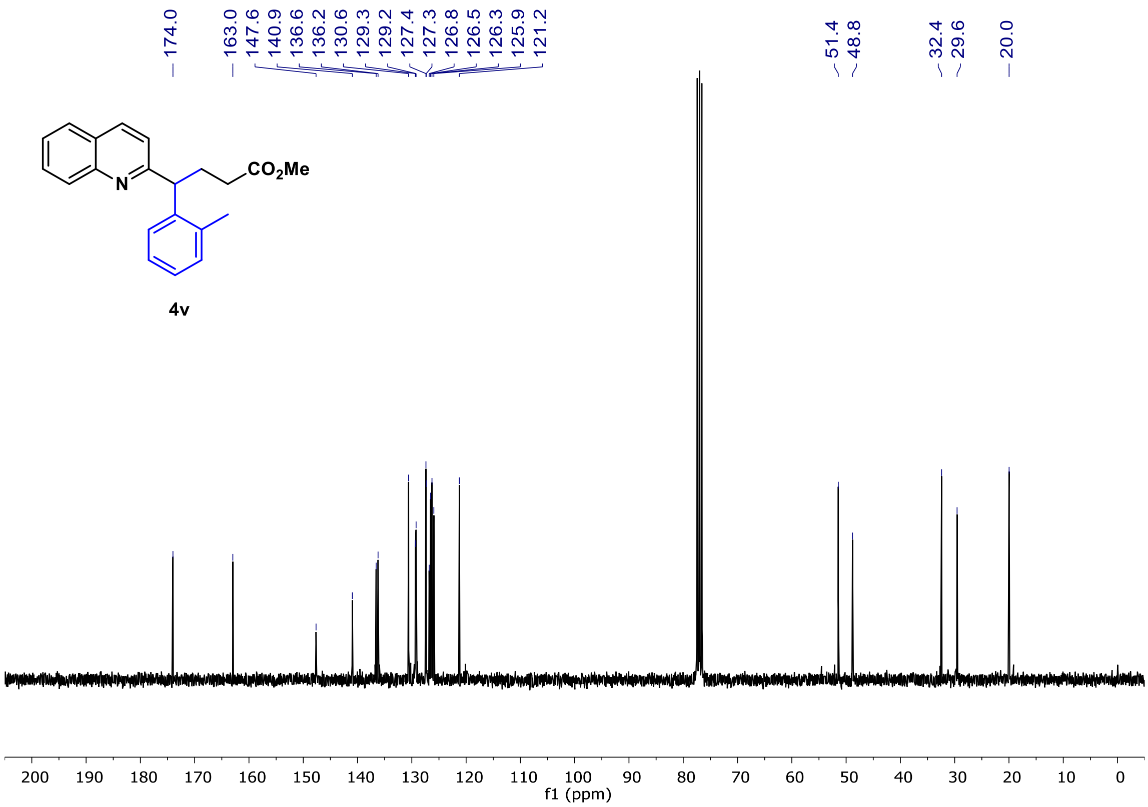

Supplementary Fig. 50. ^{13}C NMR Spectra of **4v**.

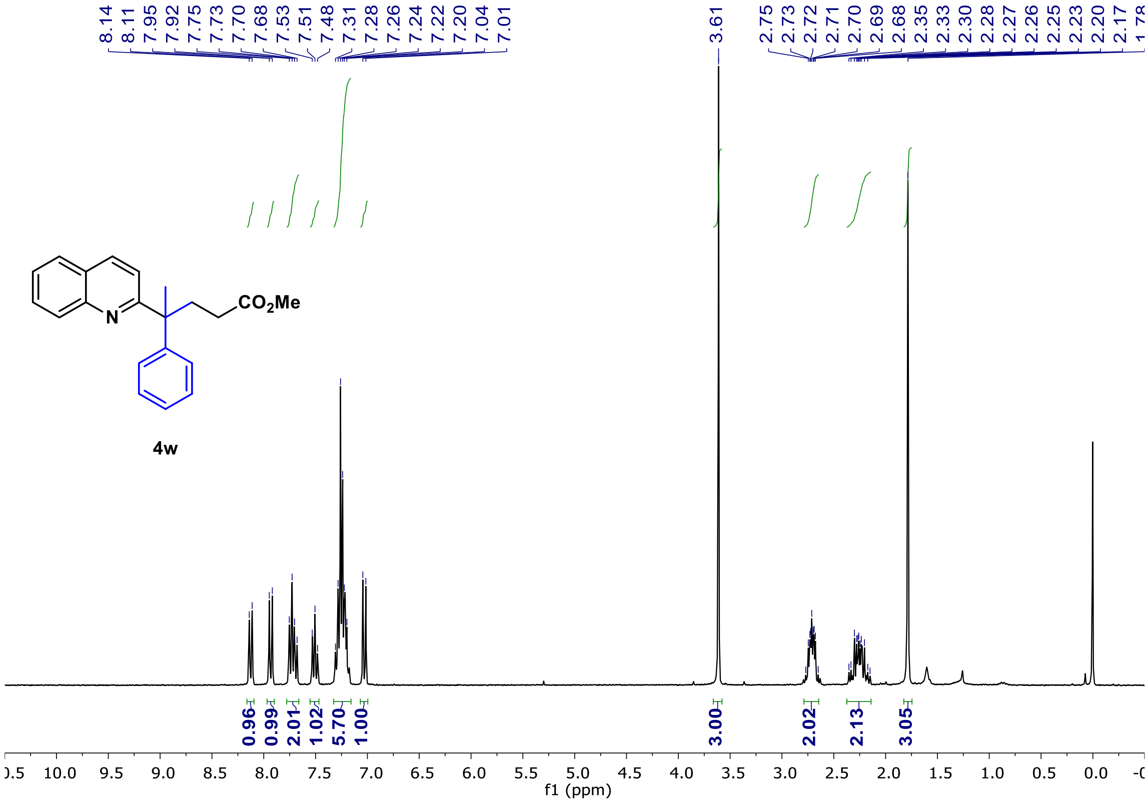

Supplementary Fig. 51. ^{1}H NMR Spectra of **4w**.

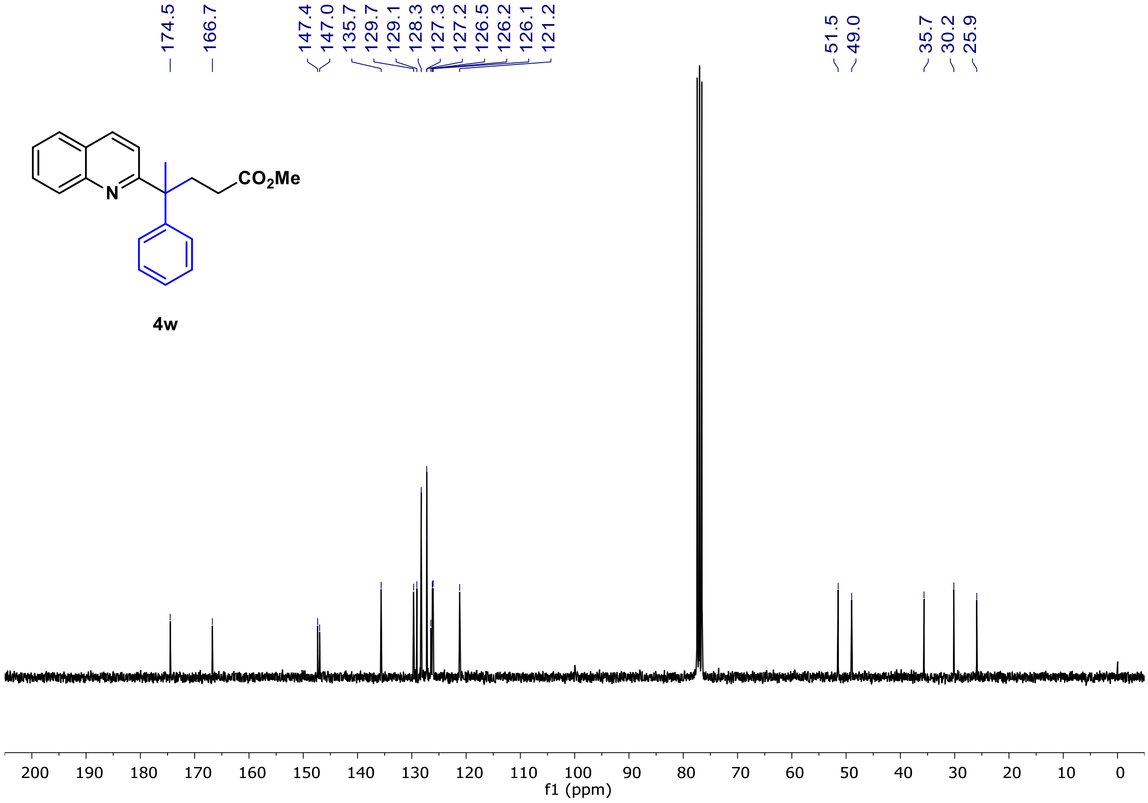

Supplementary Fig. 52. ^{13}C NMR Spectra of **4w**.

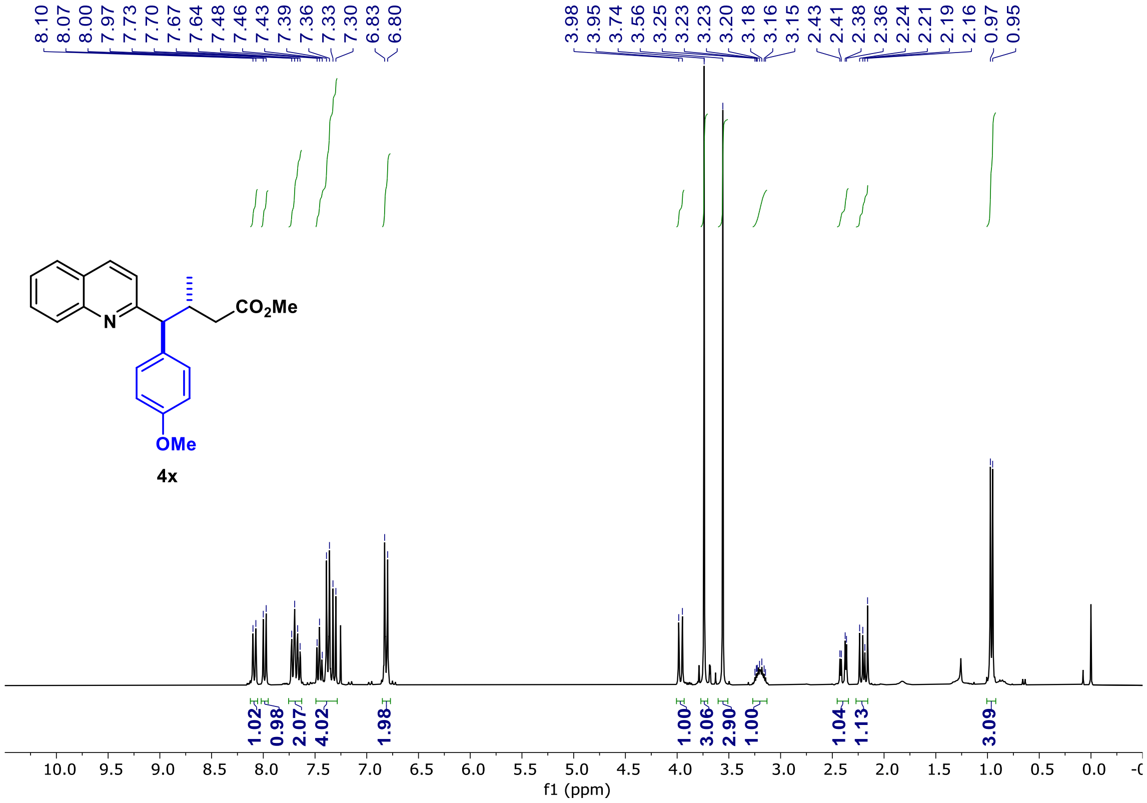

Supplementary Fig. 53. ^{1}H NMR Spectra of **4x**.

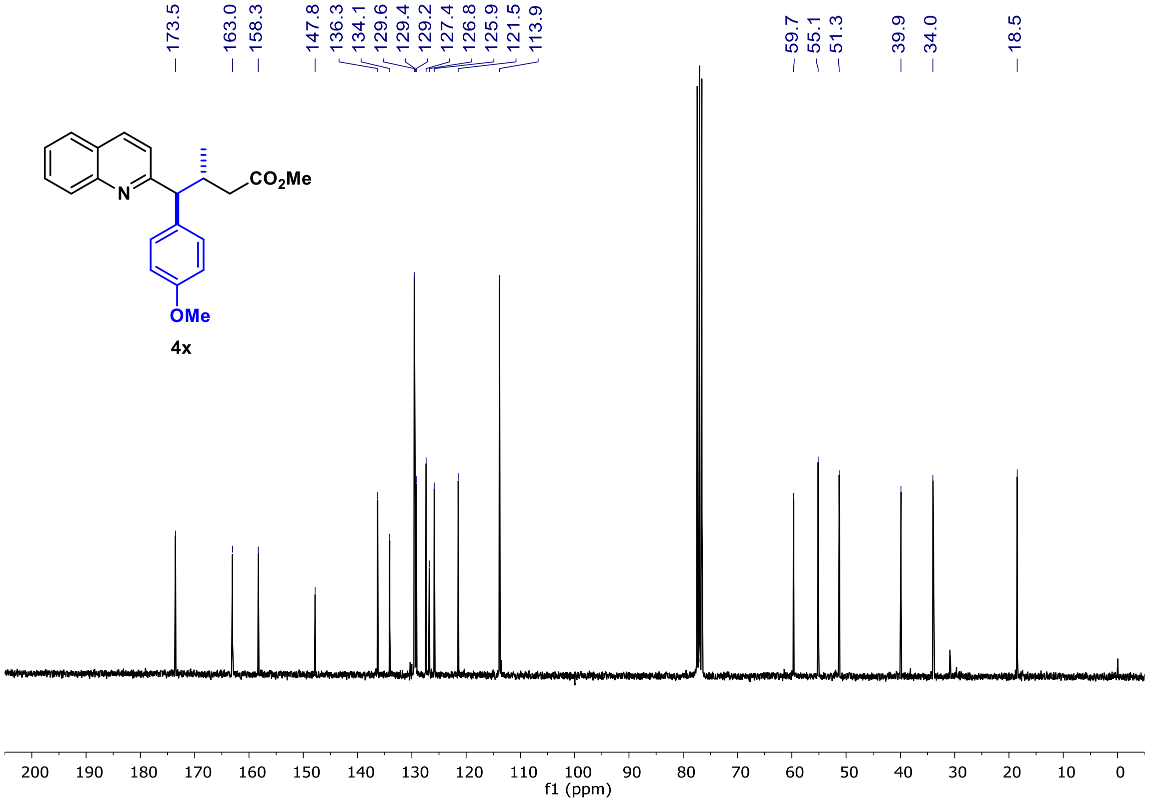

Supplementary Fig. 54. ^{13}C NMR Spectra of **4x**.

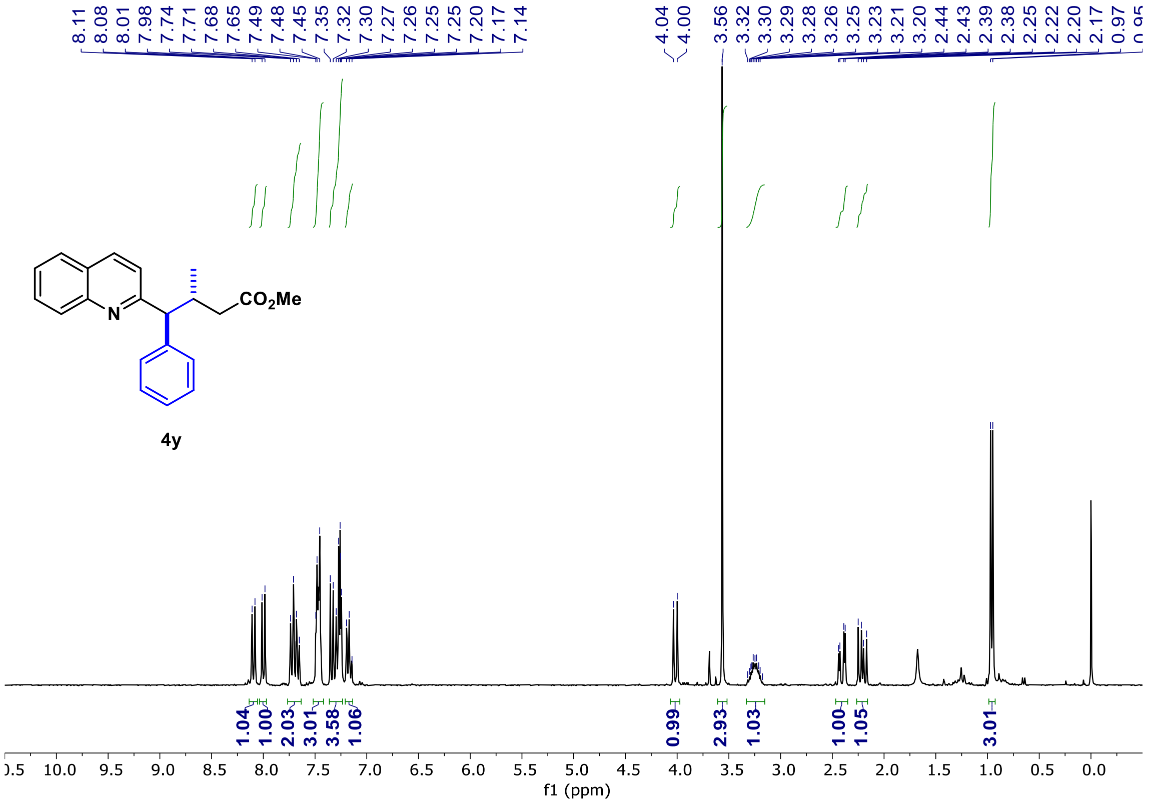

Supplementary Fig. 55. ^{1}H NMR Spectra of **4y**.

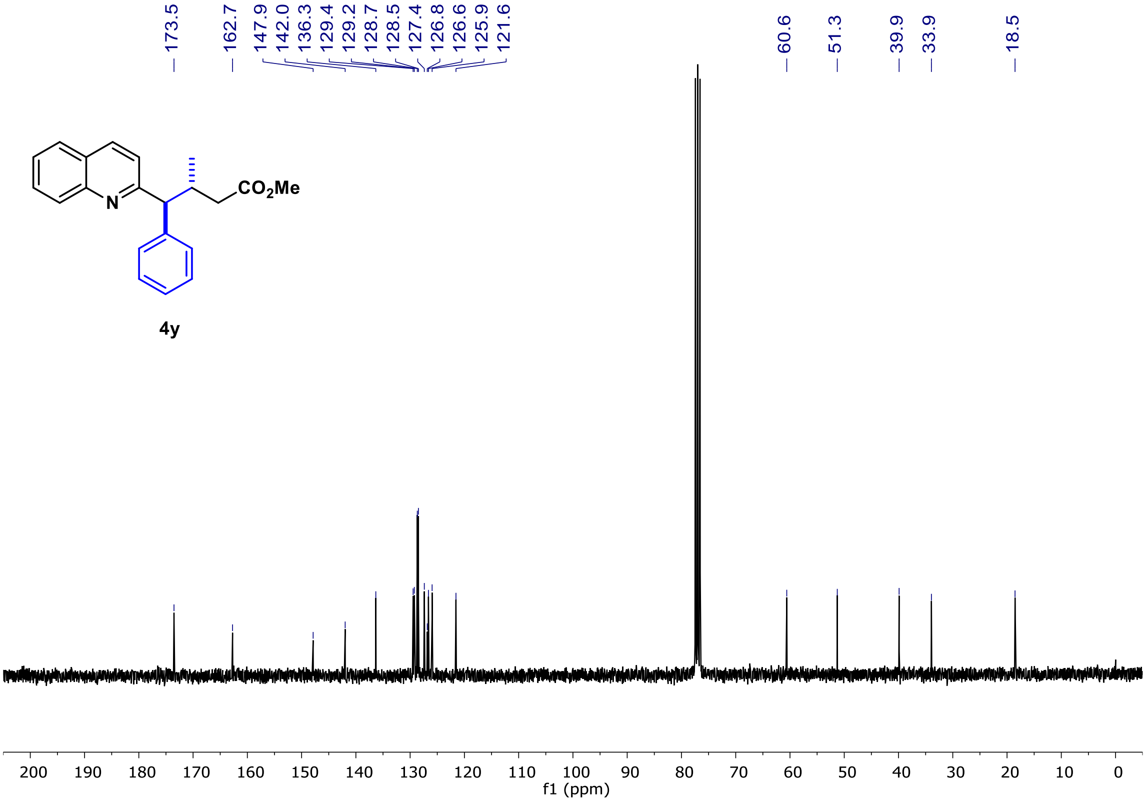

Supplementary Fig. 56. ^{13}C NMR Spectra of **4y**.

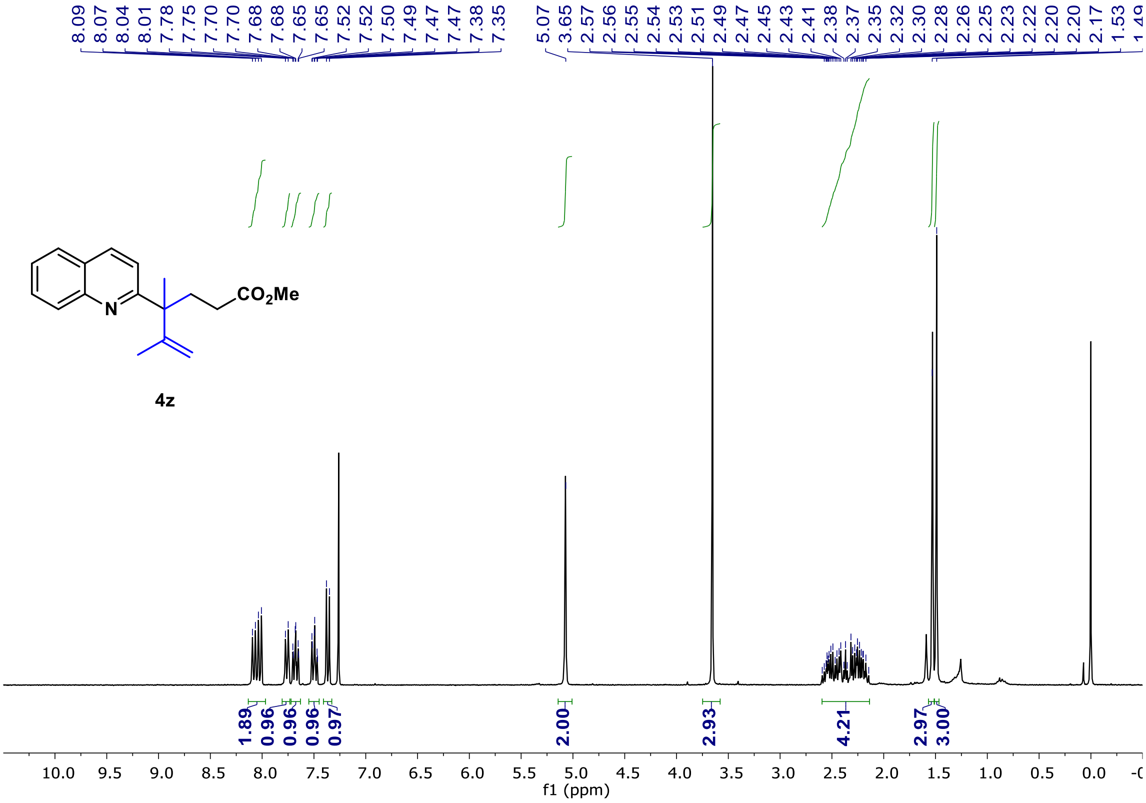

Supplementary Fig. 57. ^{1}H NMR Spectra of **4z**.

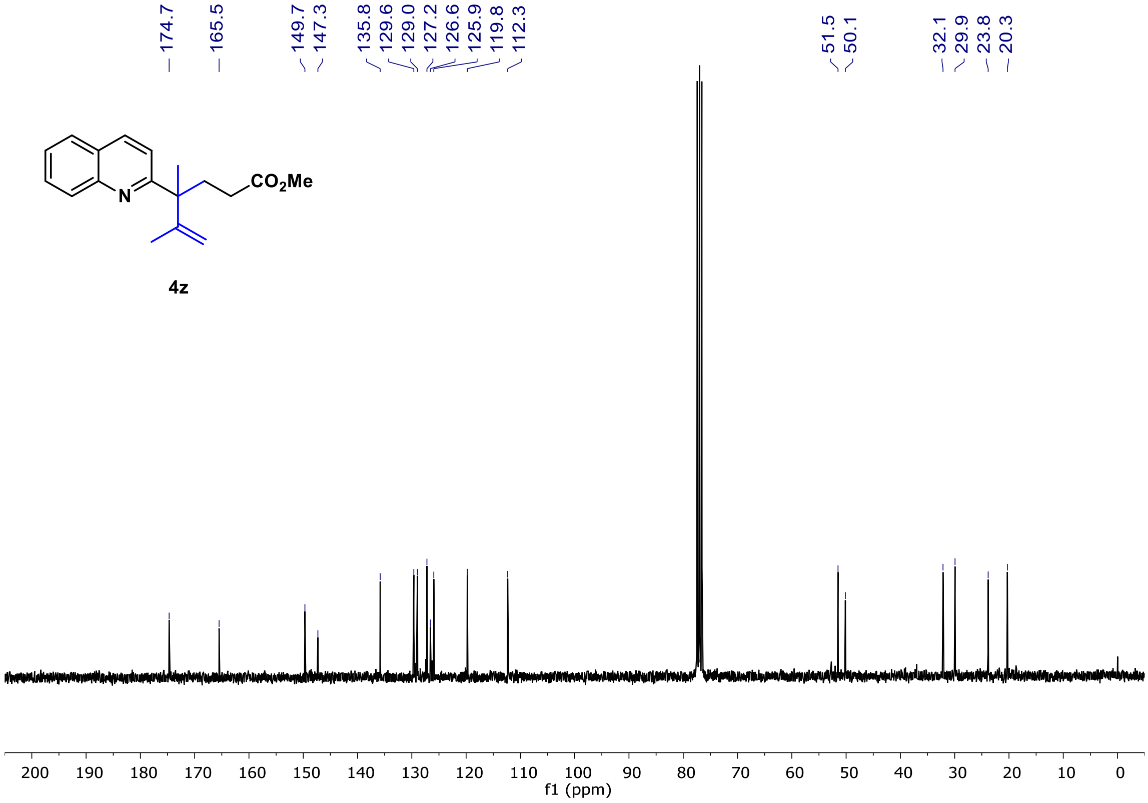

Supplementary Fig. 58. ^{13}C NMR Spectra of **4z**.

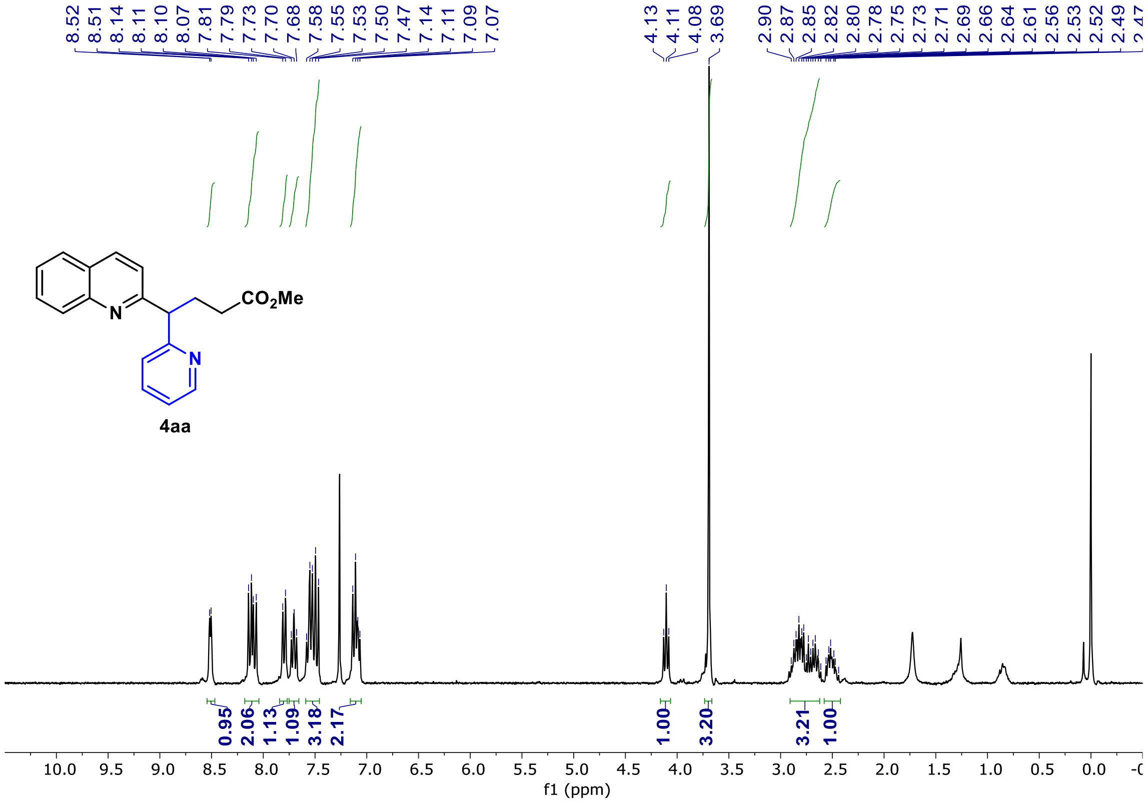

Supplementary Fig. 59. ^{1}H NMR Spectra of **4aa**.

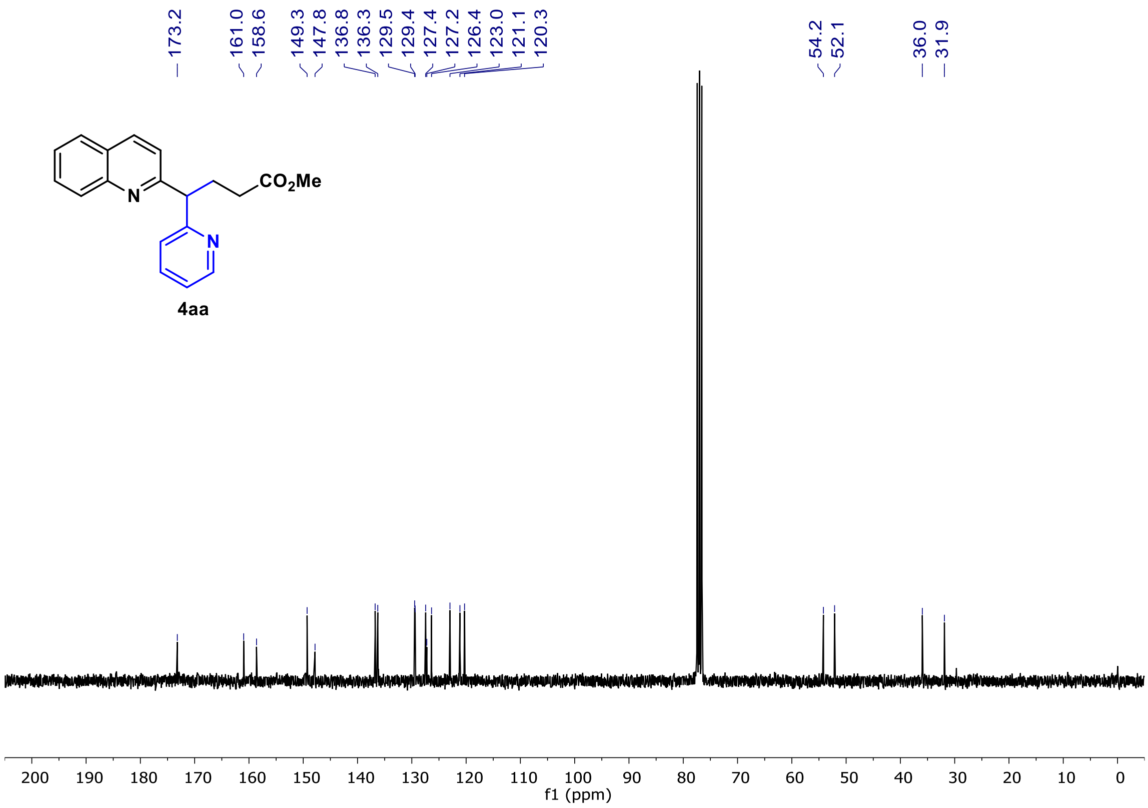

Supplementary Fig. 60. ^{13}C NMR Spectra of **4aa**.

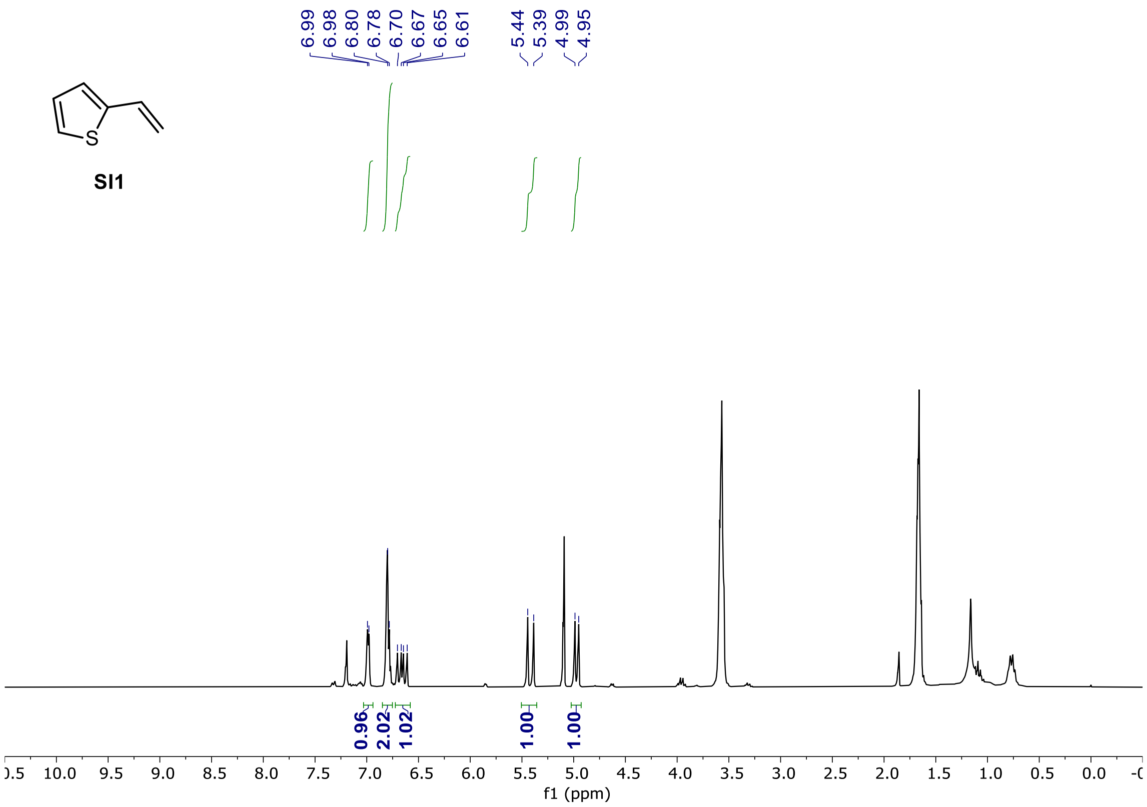

Supplementary Fig. 61. ^{1}H NMR Spectra of **SI1**.

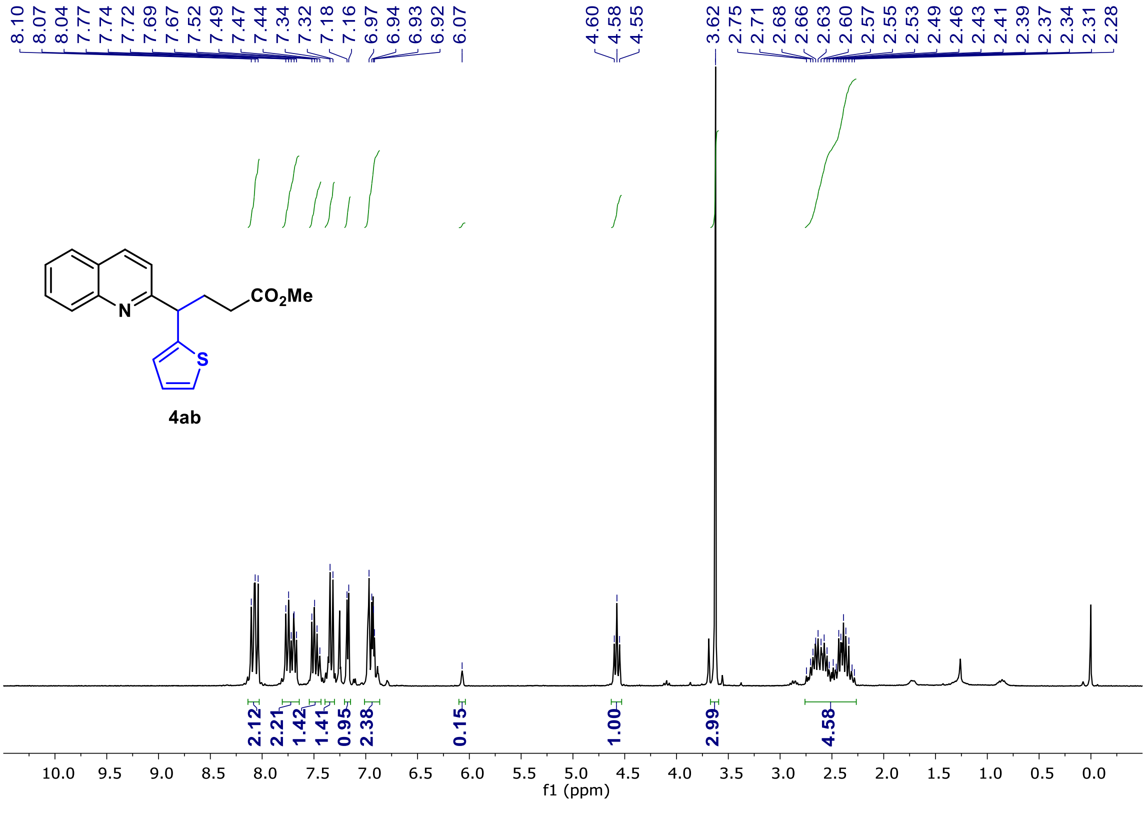

Supplementary Fig. 62. ^{1}H NMR Spectra of **4ab**.

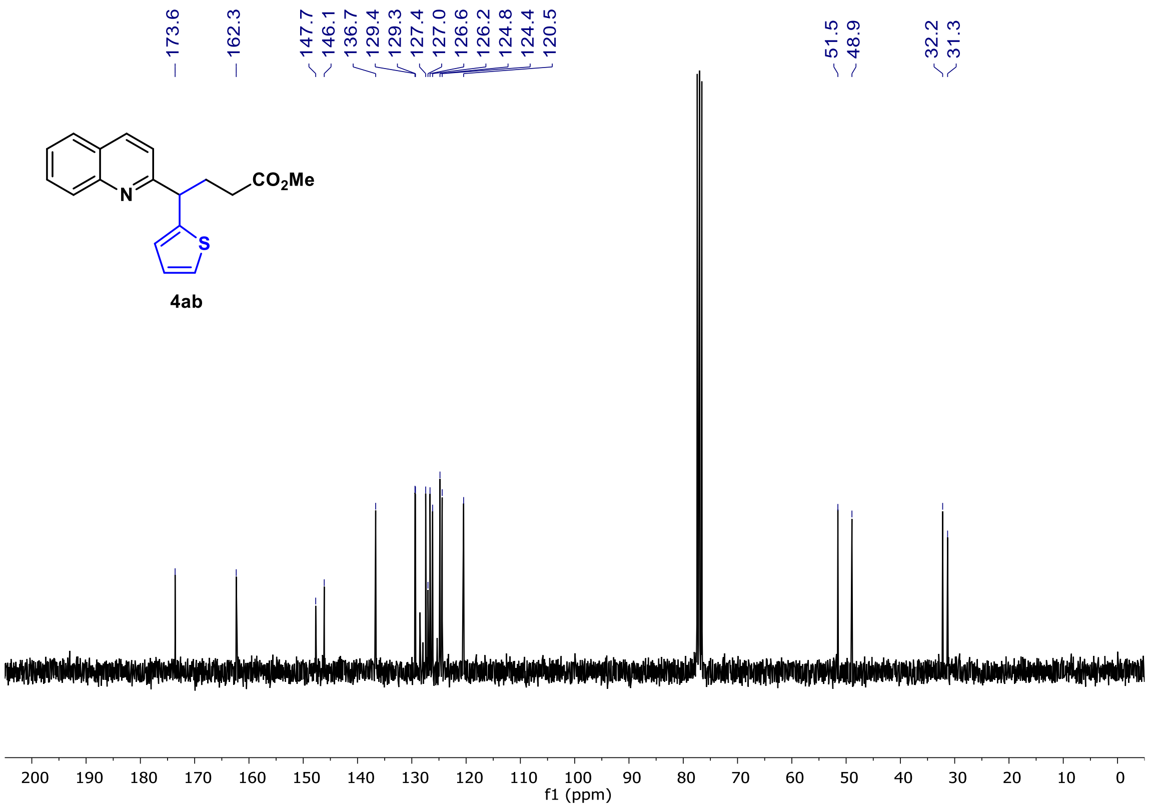

Supplementary Fig. 63. ^{13}C NMR Spectra of **4ab**.

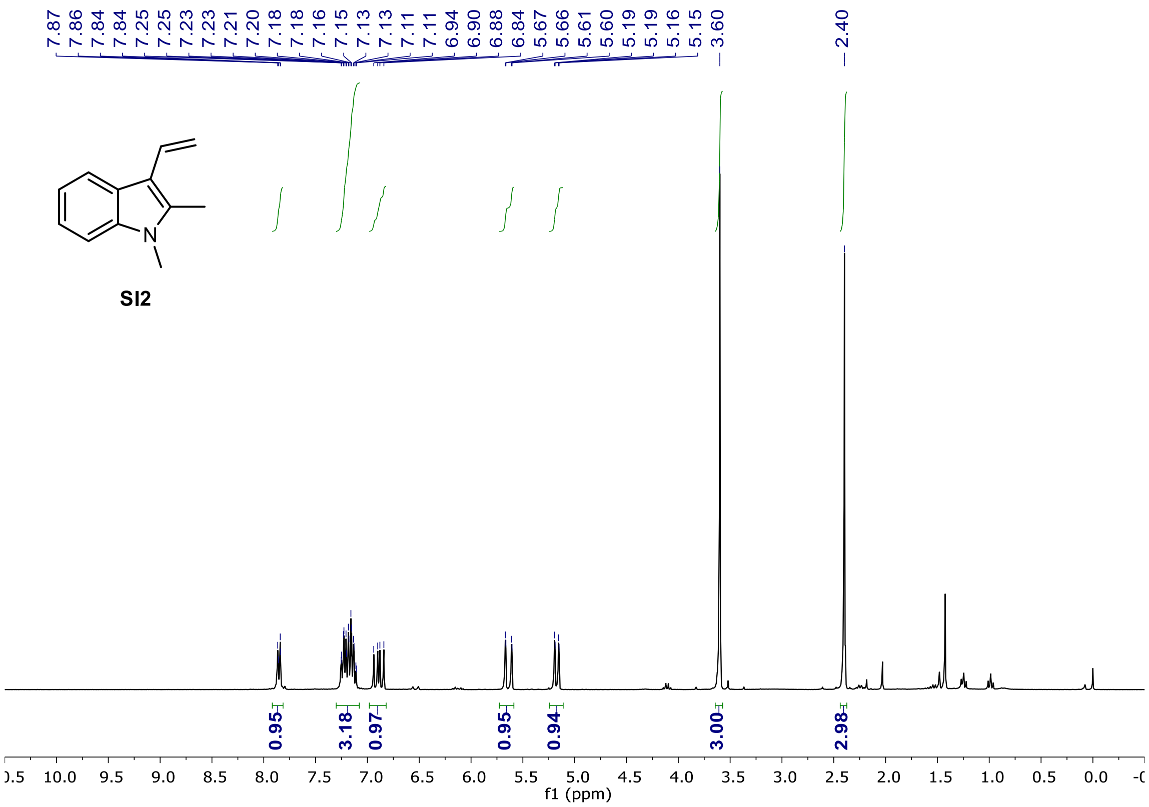

Supplementary Fig. 64. ^{1}H NMR Spectra of **SI2**.

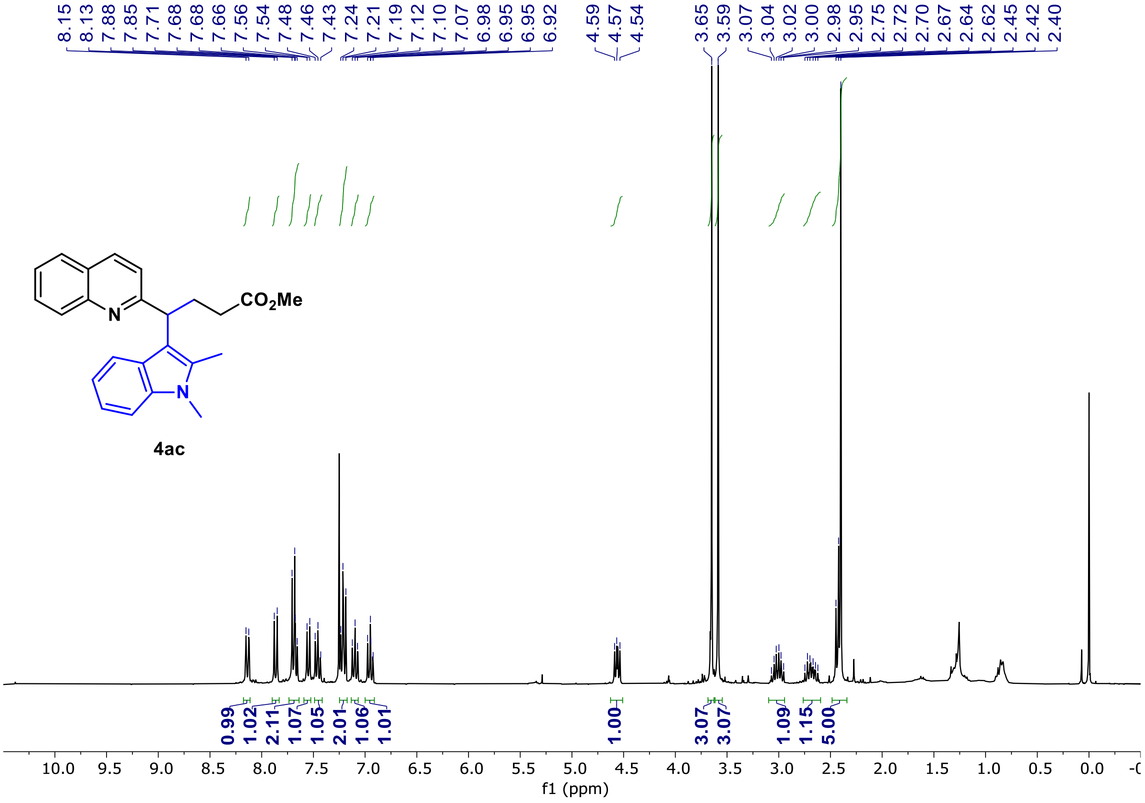

Supplementary Fig. 65. ^{1}H NMR Spectra of **4ac**.

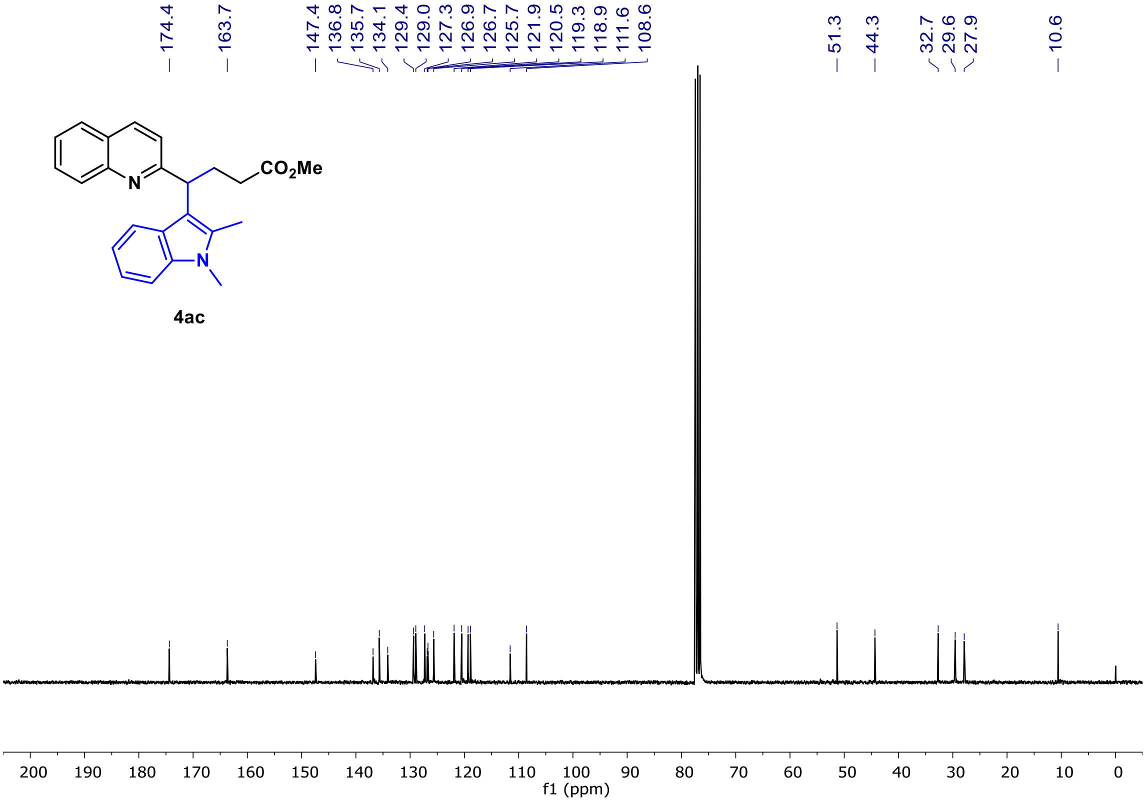

Supplementary Fig. 66. ^{13}C NMR Spectra of **4ac**.

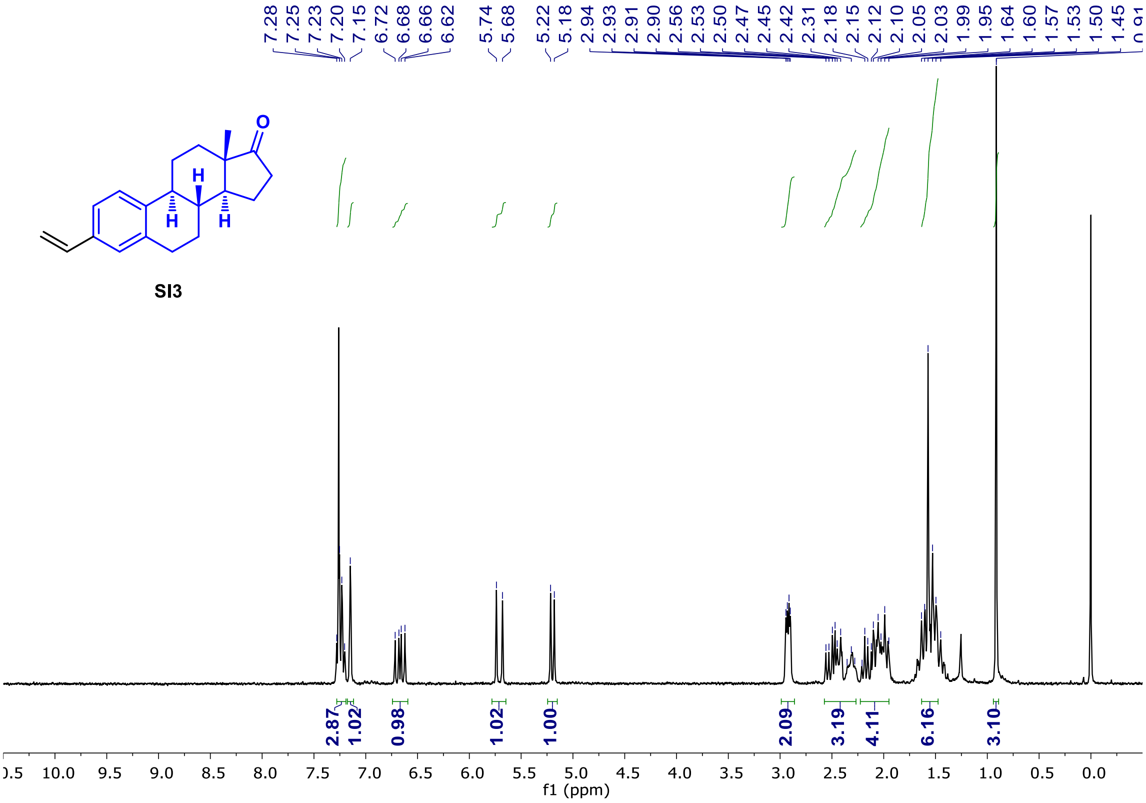

Supplementary Fig. 67. ^{1}H NMR Spectra of **SI3**.

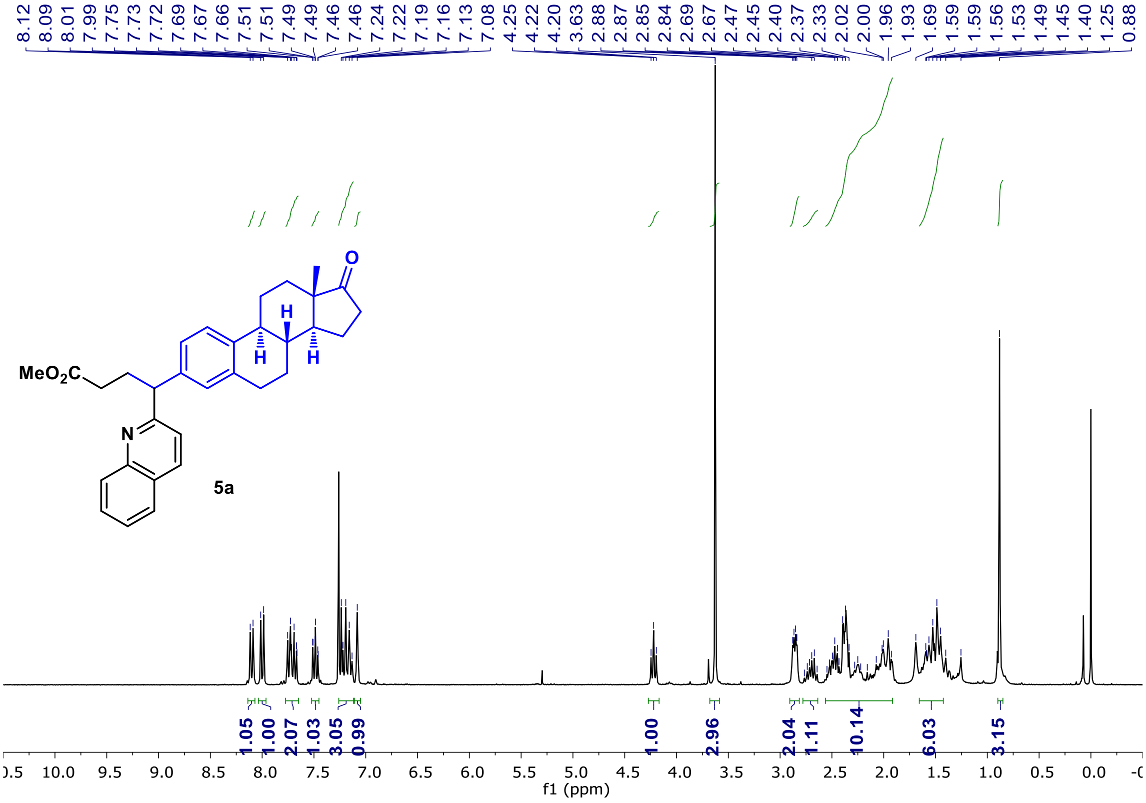

Supplementary Fig. 68. ^{1}H NMR Spectra of **5a**.

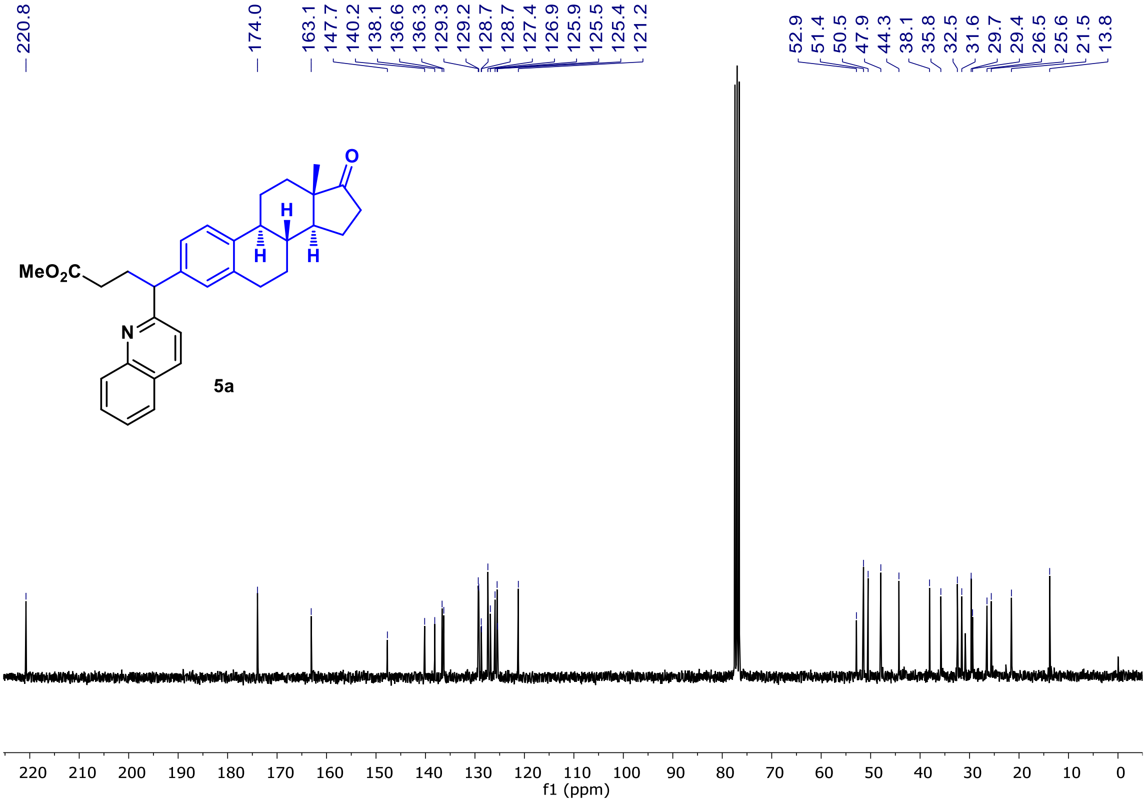

Supplementary Fig. 69. ^{13}C NMR Spectra of **5a**.

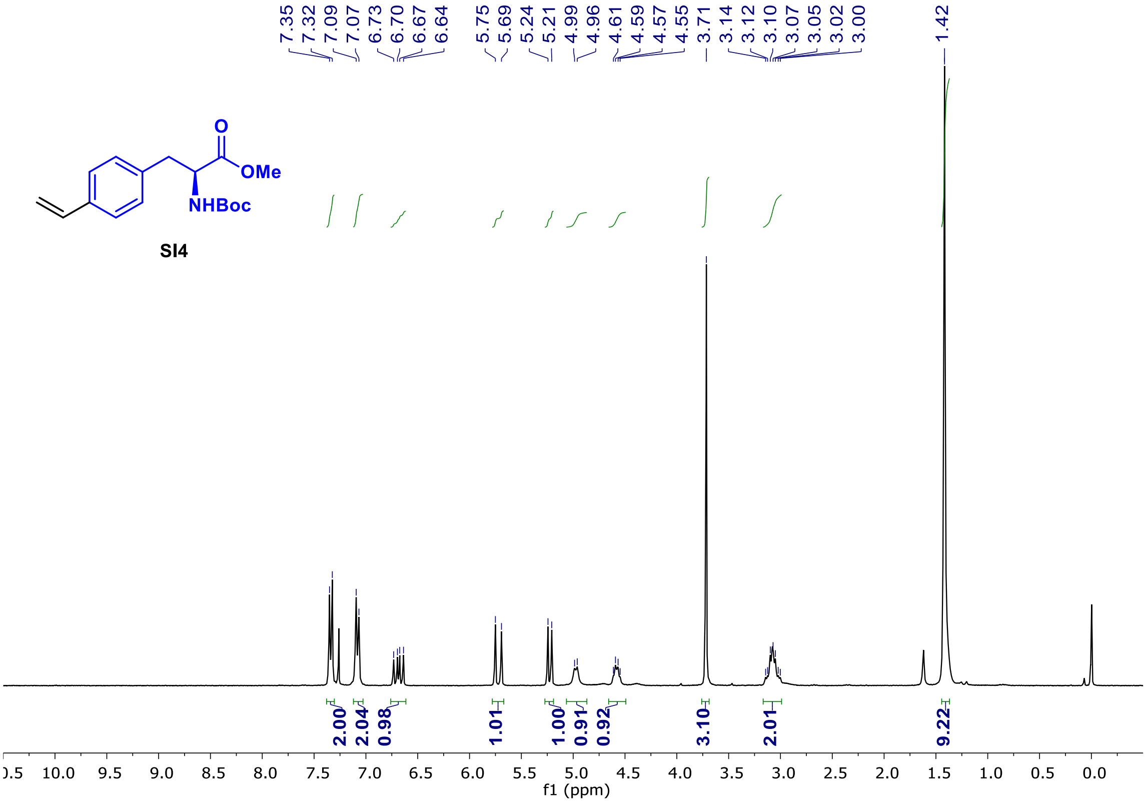

Supplementary Fig. 70. ^{1}H NMR Spectra of **SI4**.

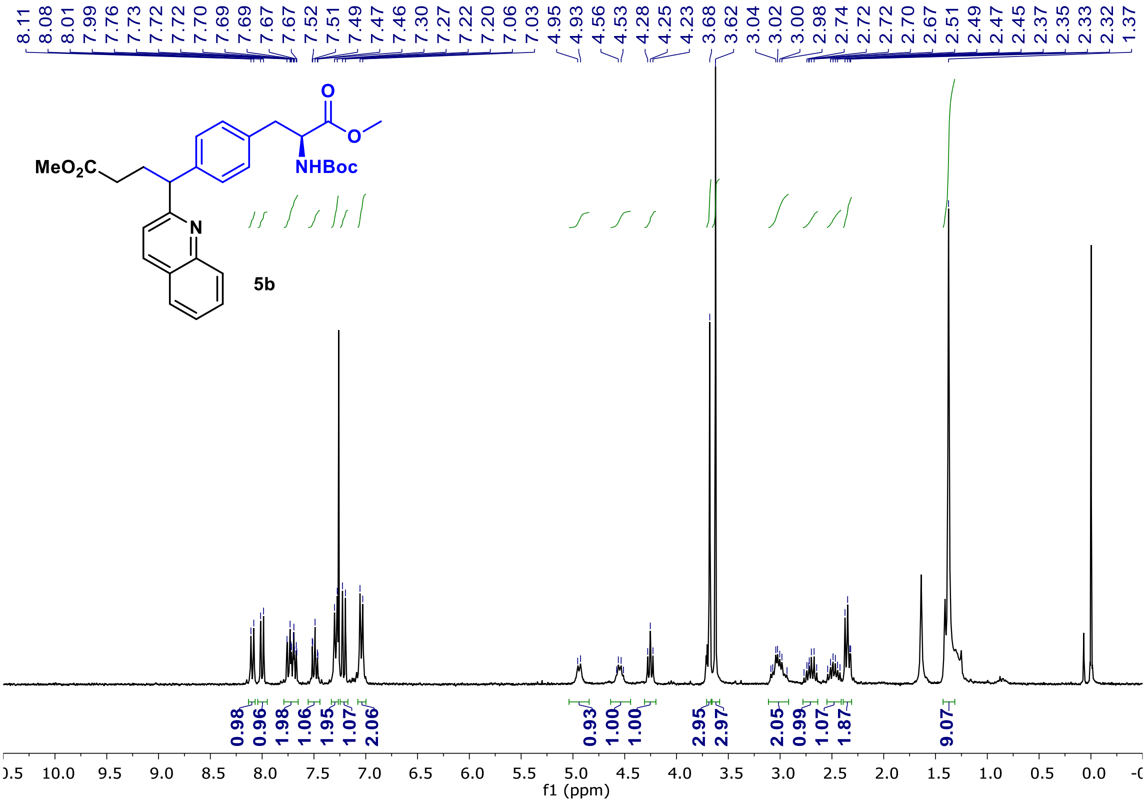

Supplementary Fig. 71. ^{1}H NMR Spectra of **5b**.

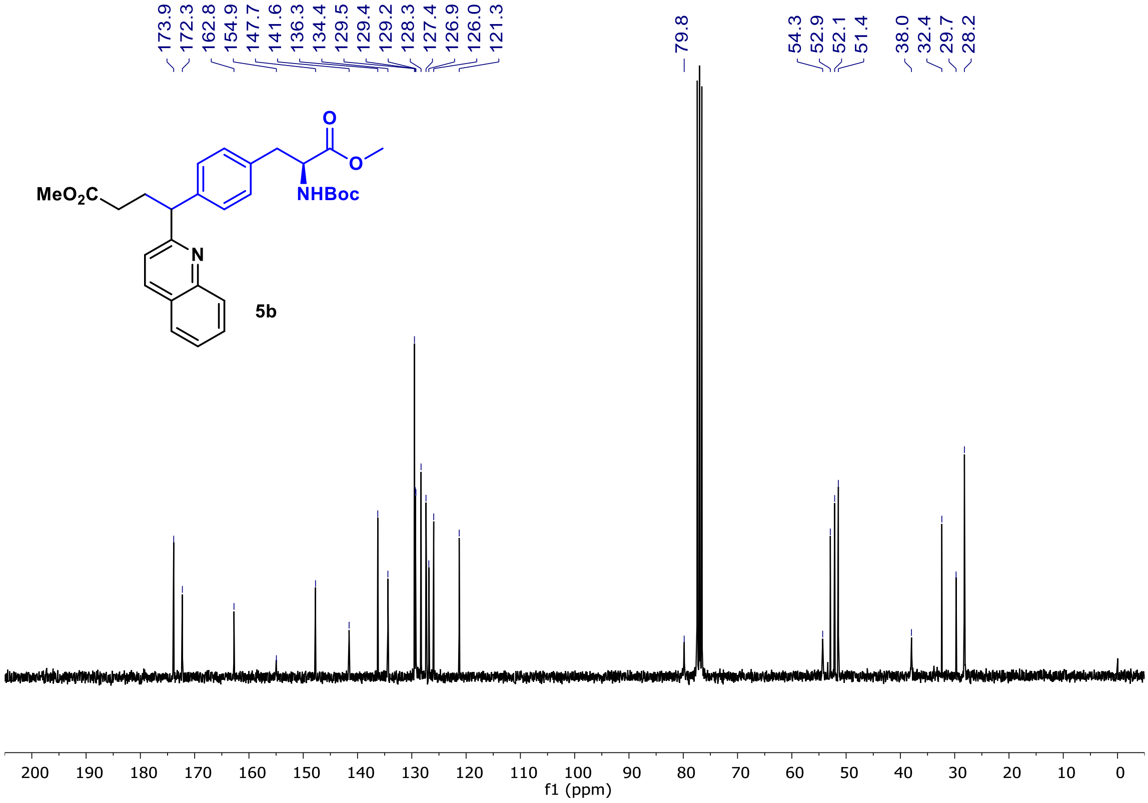

5b

Supplementary Fig. 72. ^{13}C NMR Spectra of **5b**.

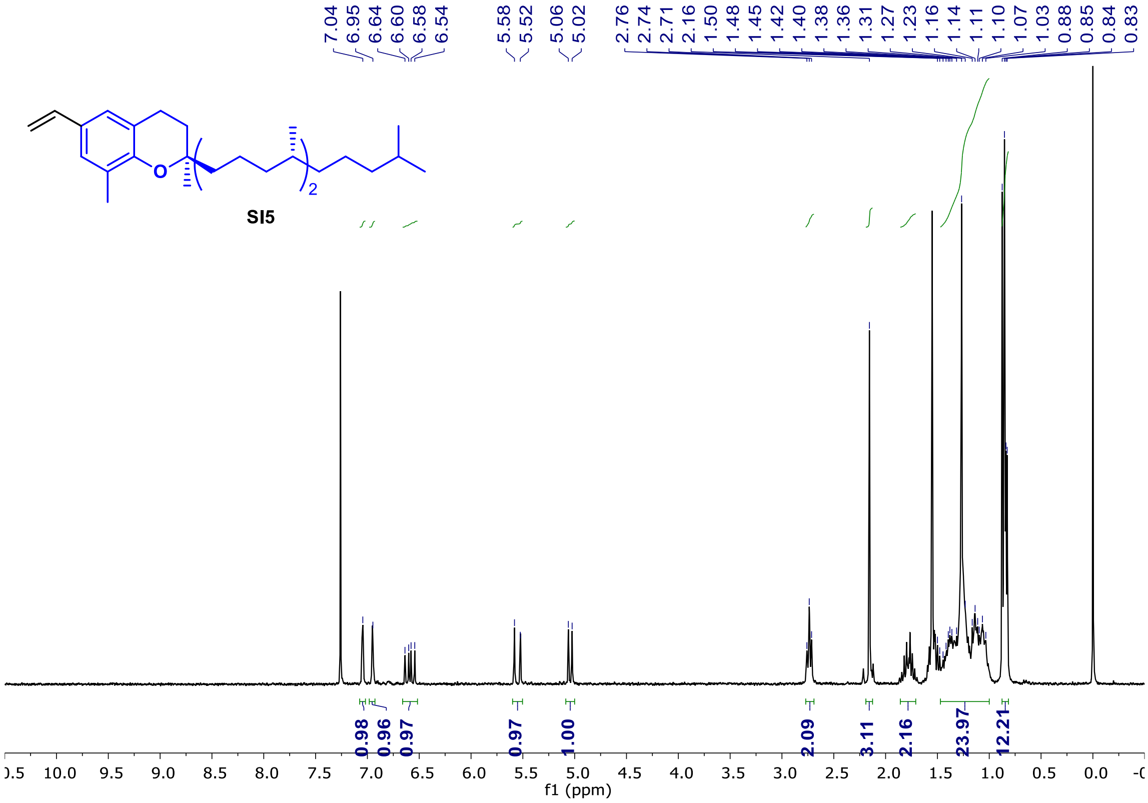

SI5

Supplementary Fig. 73. ^{1}H NMR Spectra of **SI5**.

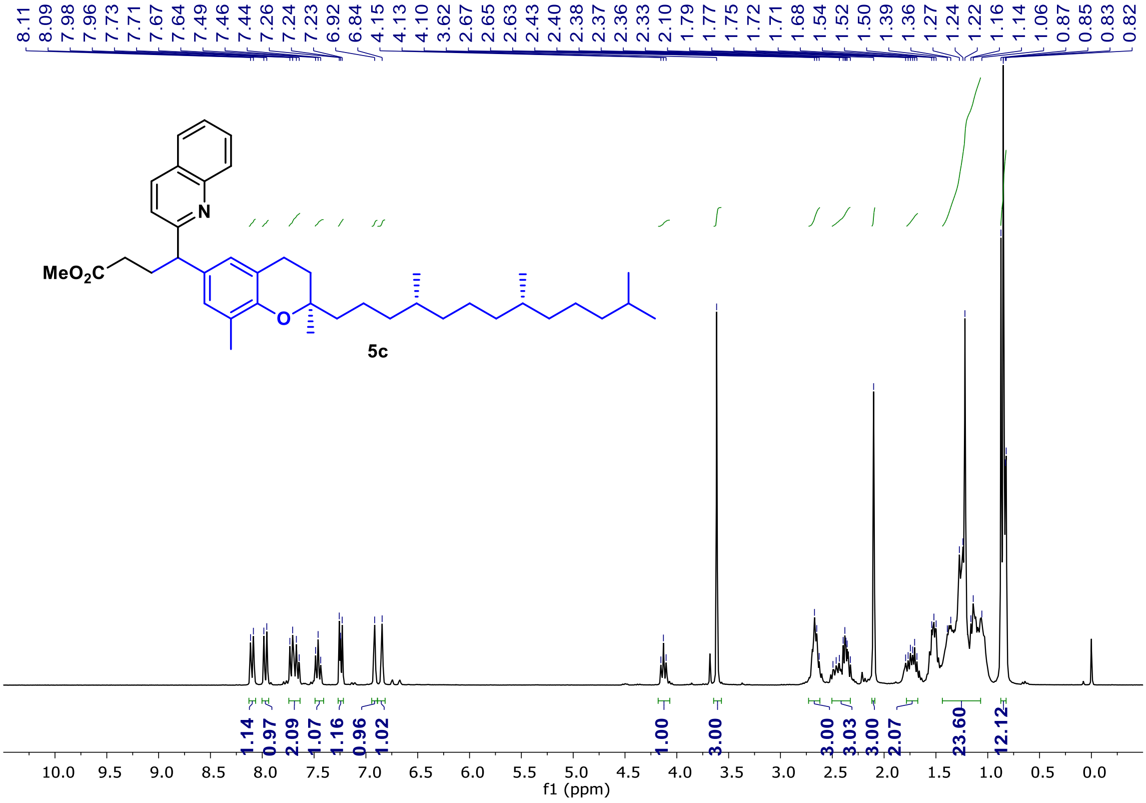

Supplementary Fig. 74. ^{1}H NMR Spectra of **5c**.

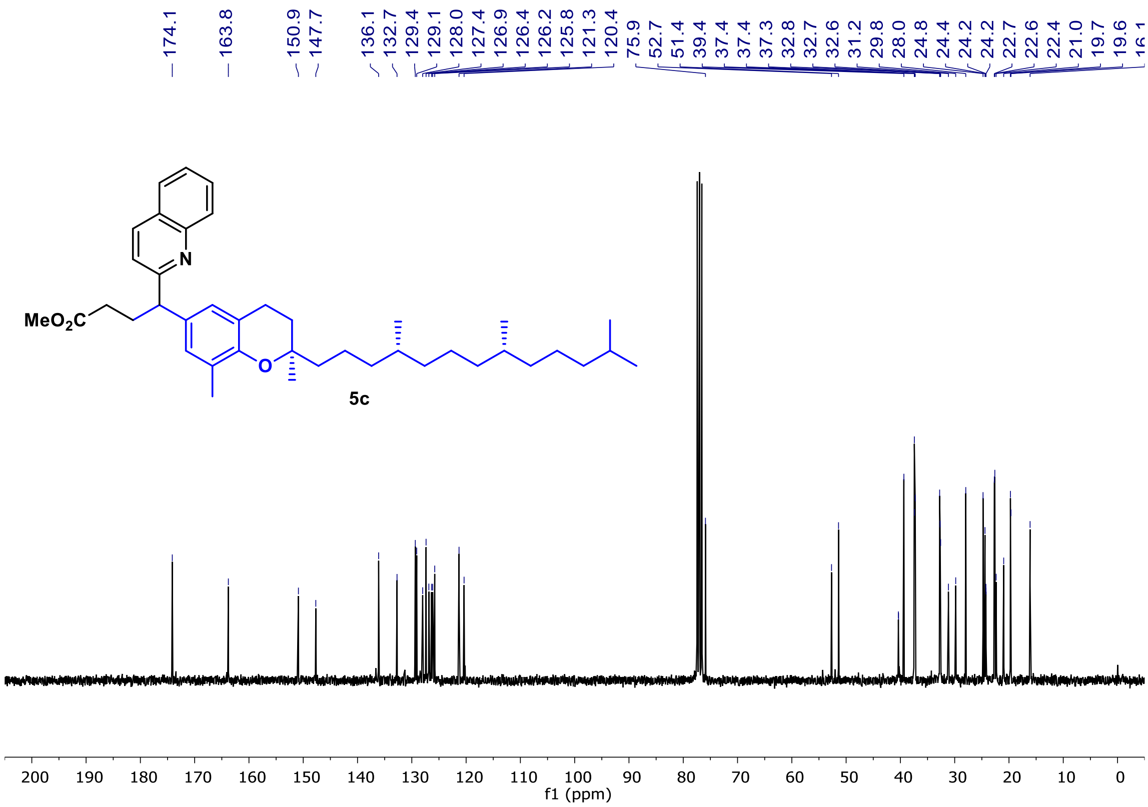

Supplementary Fig. 75. ^{13}C NMR Spectra of **5c**.

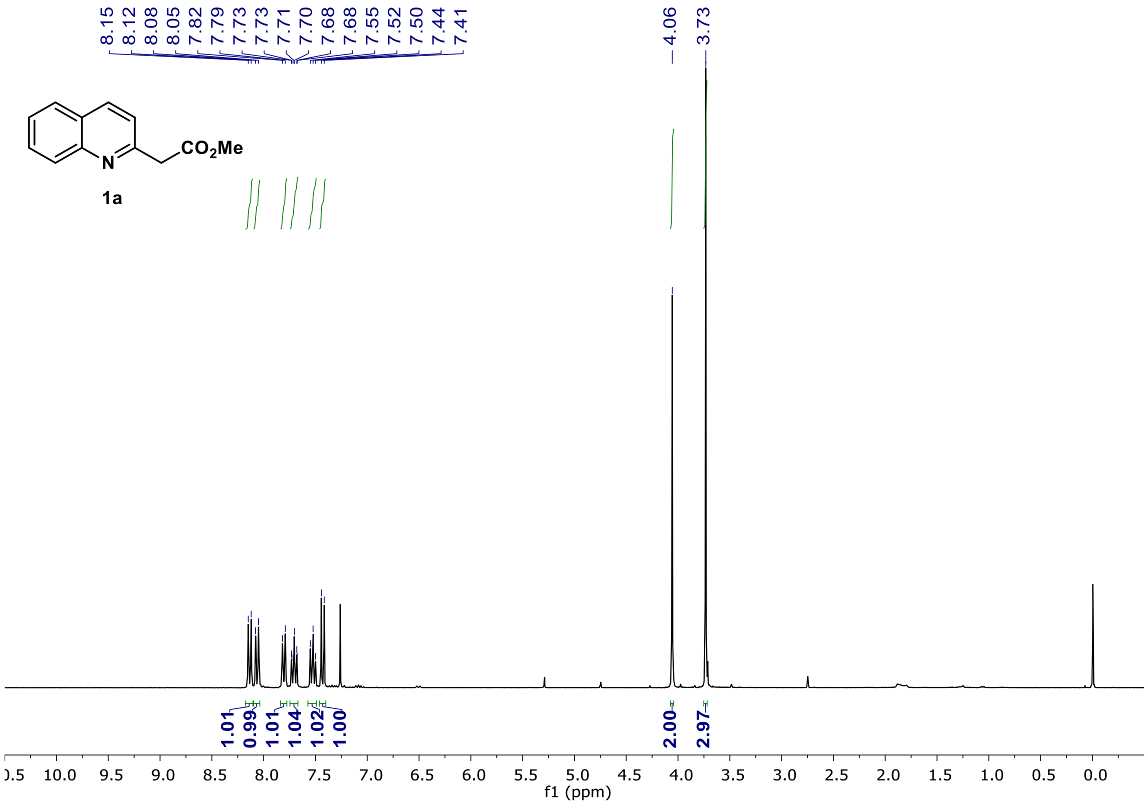

Supplementary Fig. 76. ^{1}H NMR Spectra of **1a**.

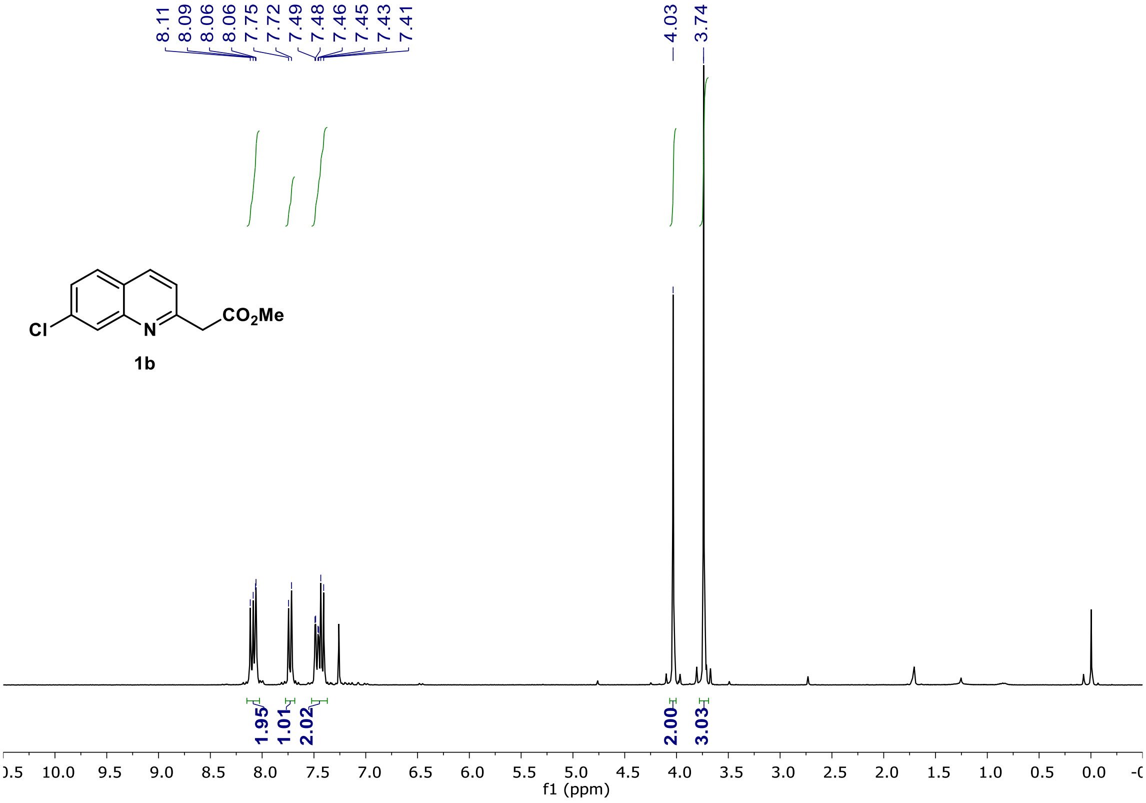

Supplementary Fig. 77. ^{1}H NMR Spectra of **1b**.

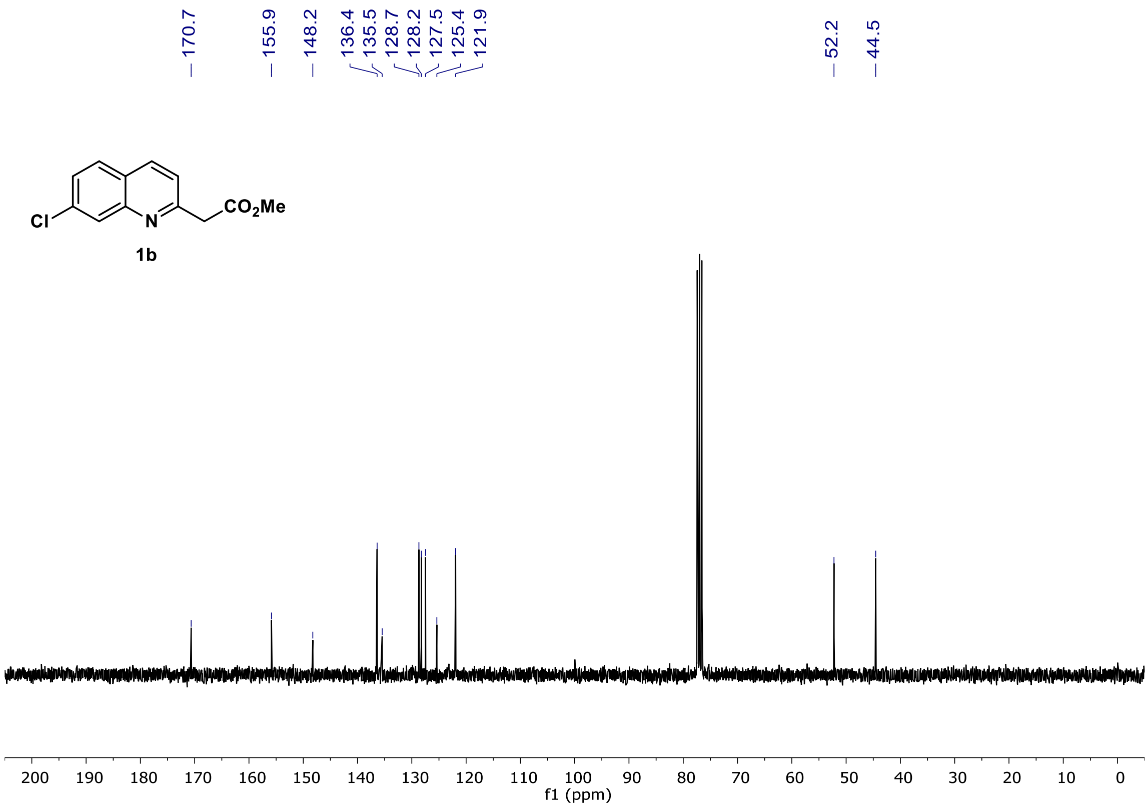

Supplementary Fig. 78. ^{13}C NMR Spectra of **1b**.

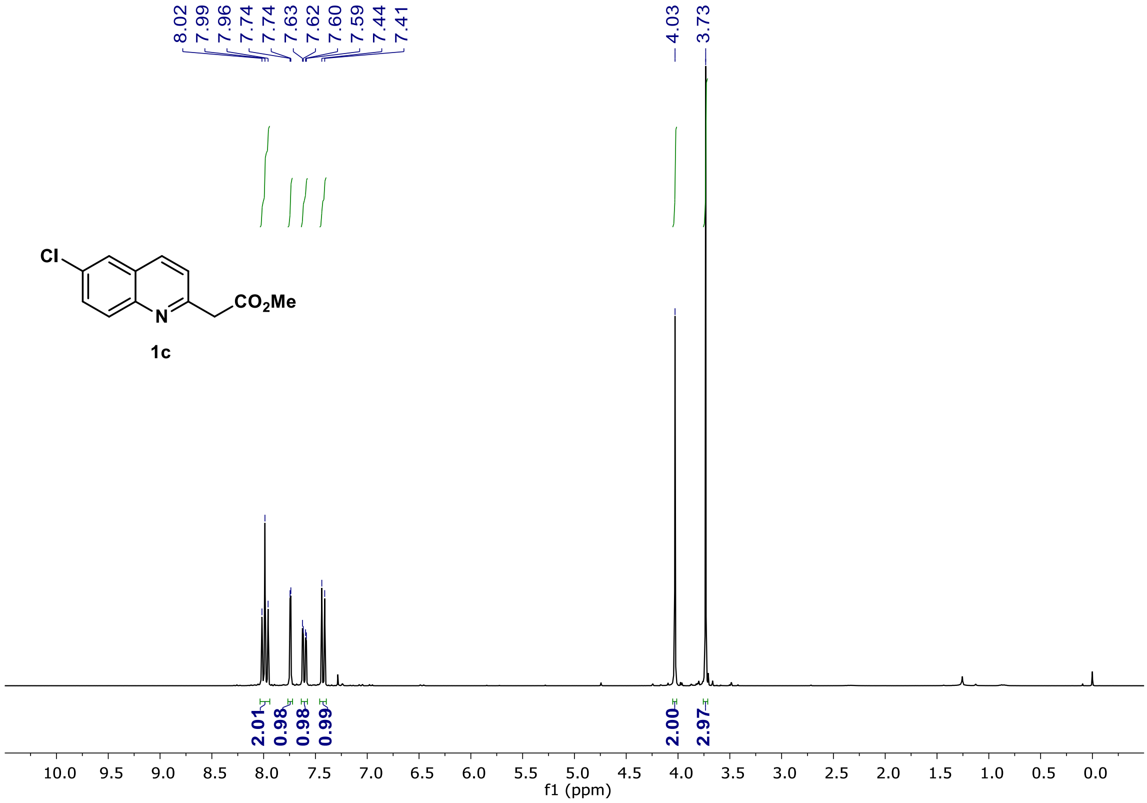

Supplementary Fig. 79. ^{1}H NMR Spectra of **1c**.

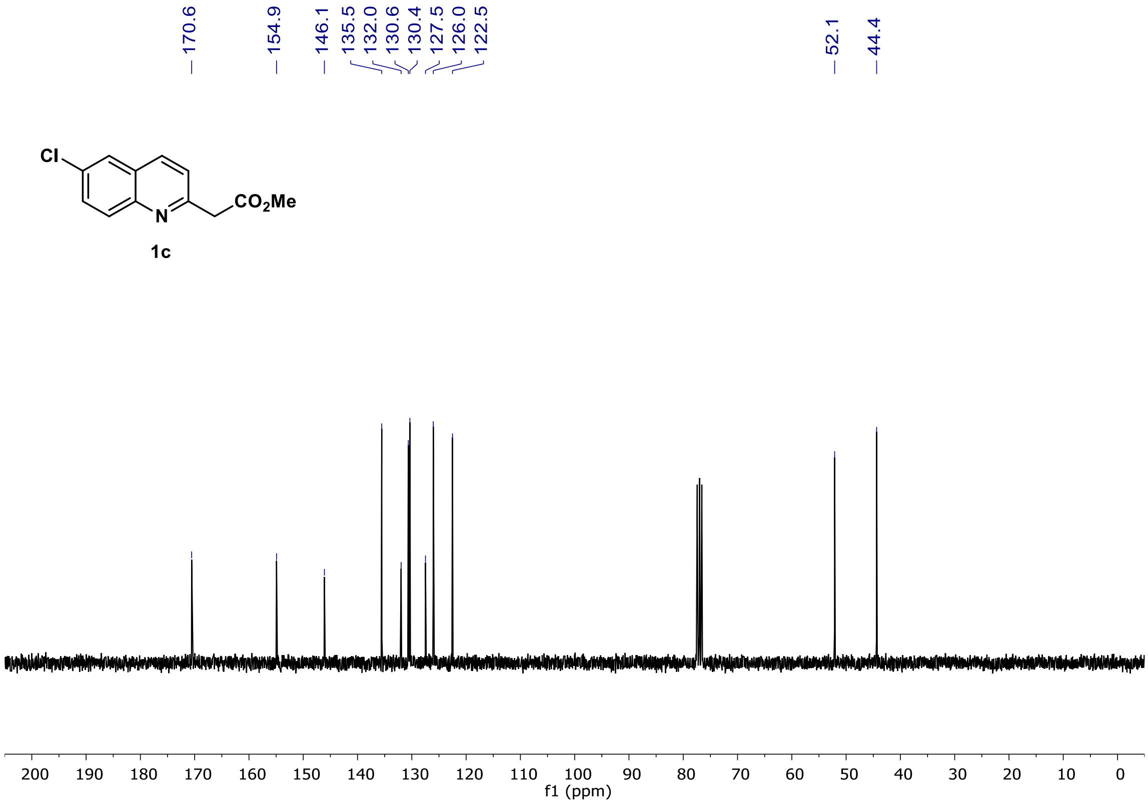

Supplementary Fig. 80. ^{13}C NMR Spectra of **1c**.

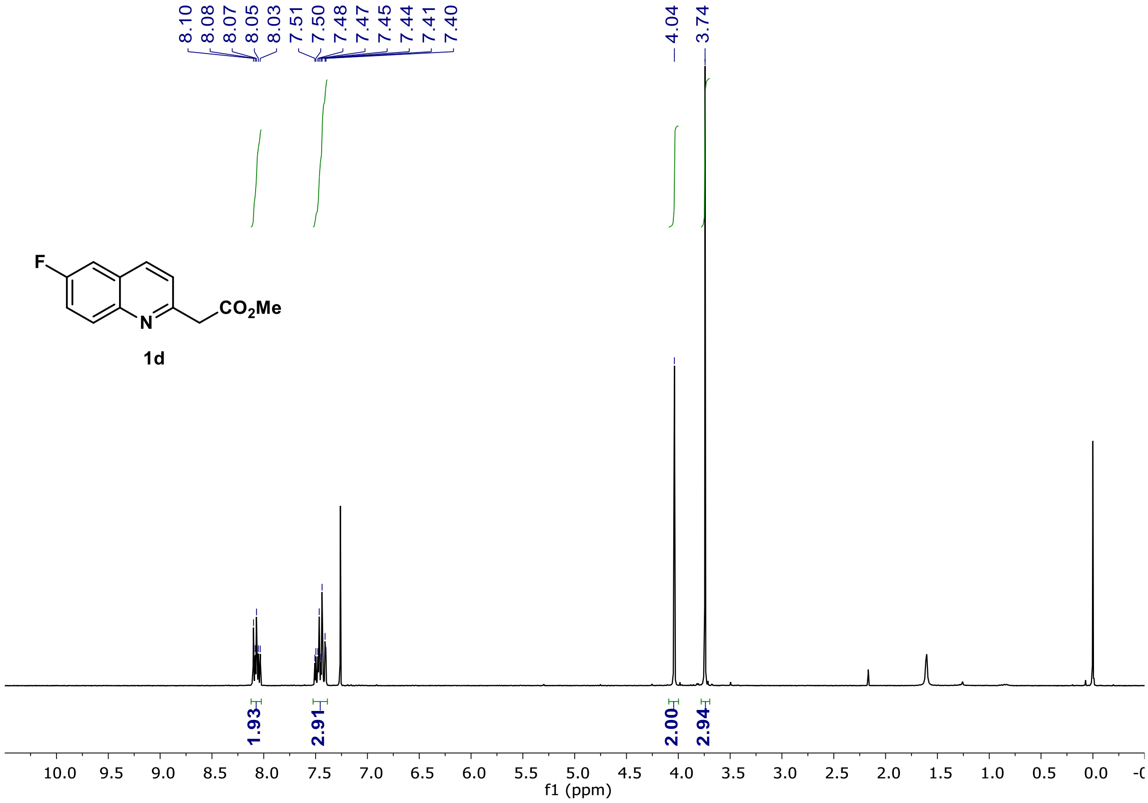

Supplementary Fig. 81. ^{1}H NMR Spectra of **1d**.

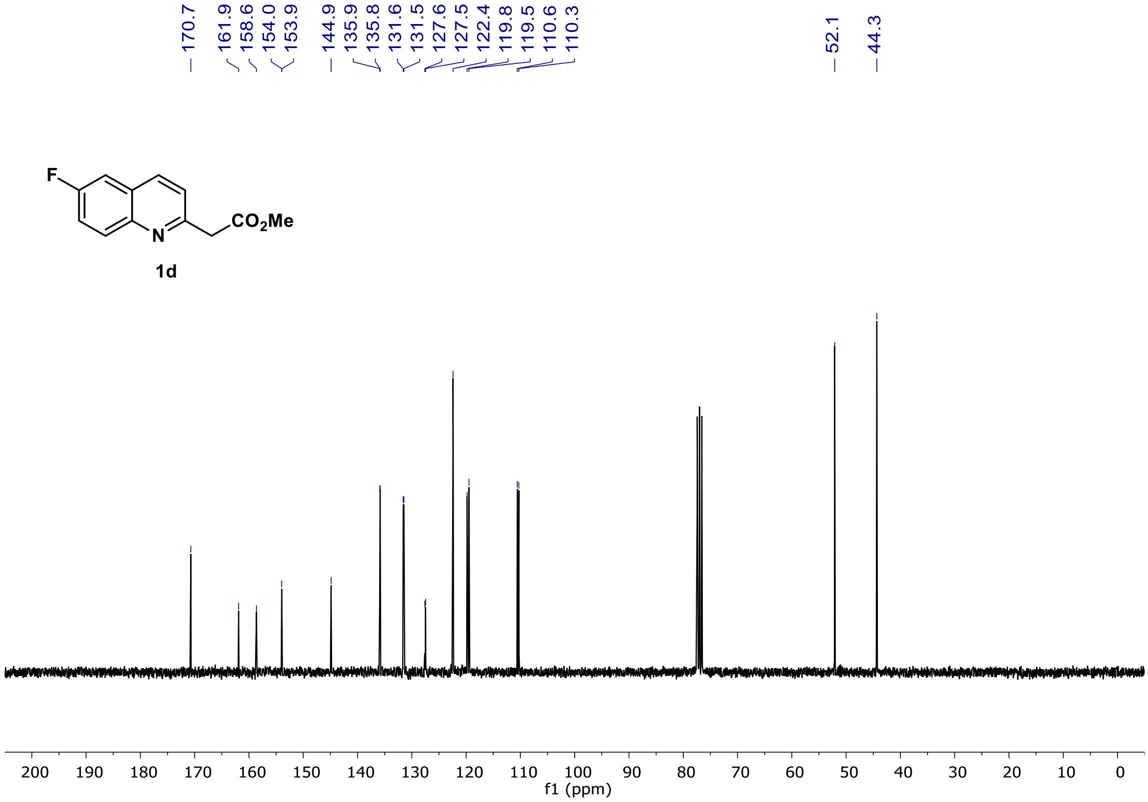

Supplementary Fig. 82. ^{13}C NMR Spectra of **1d**.

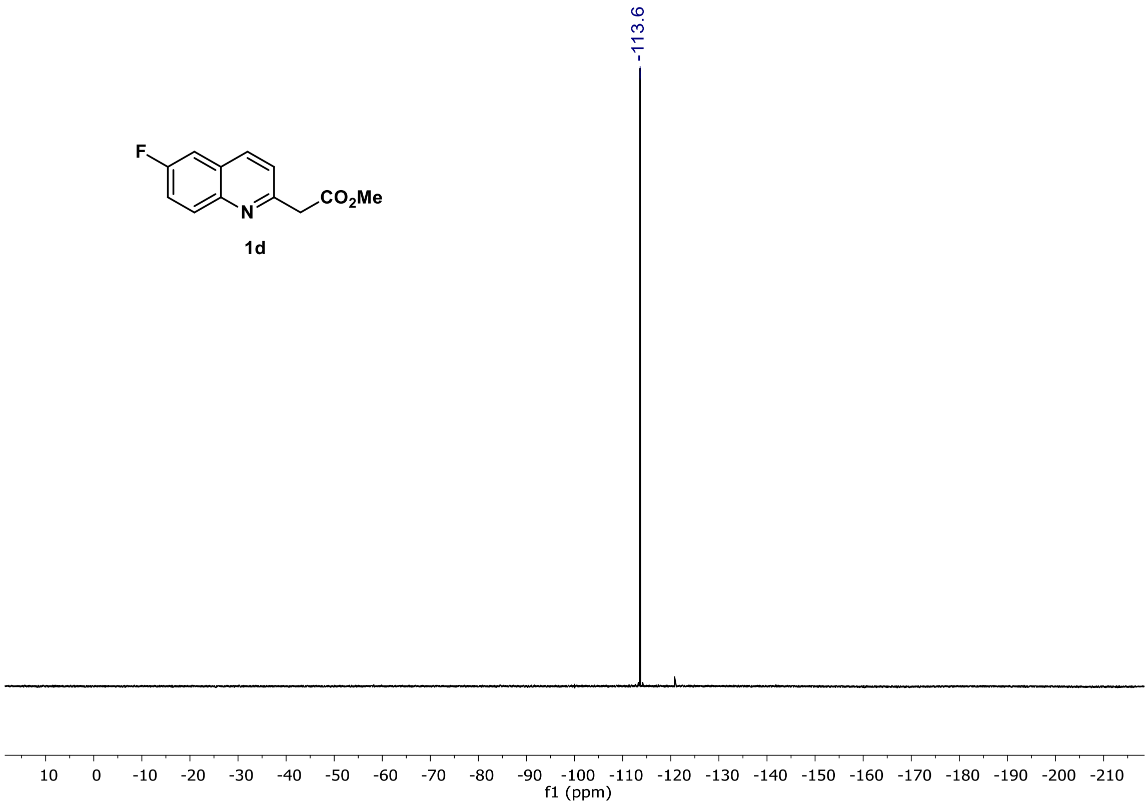

Supplementary Fig. 83. ^{19}F NMR Spectra of **1d**.

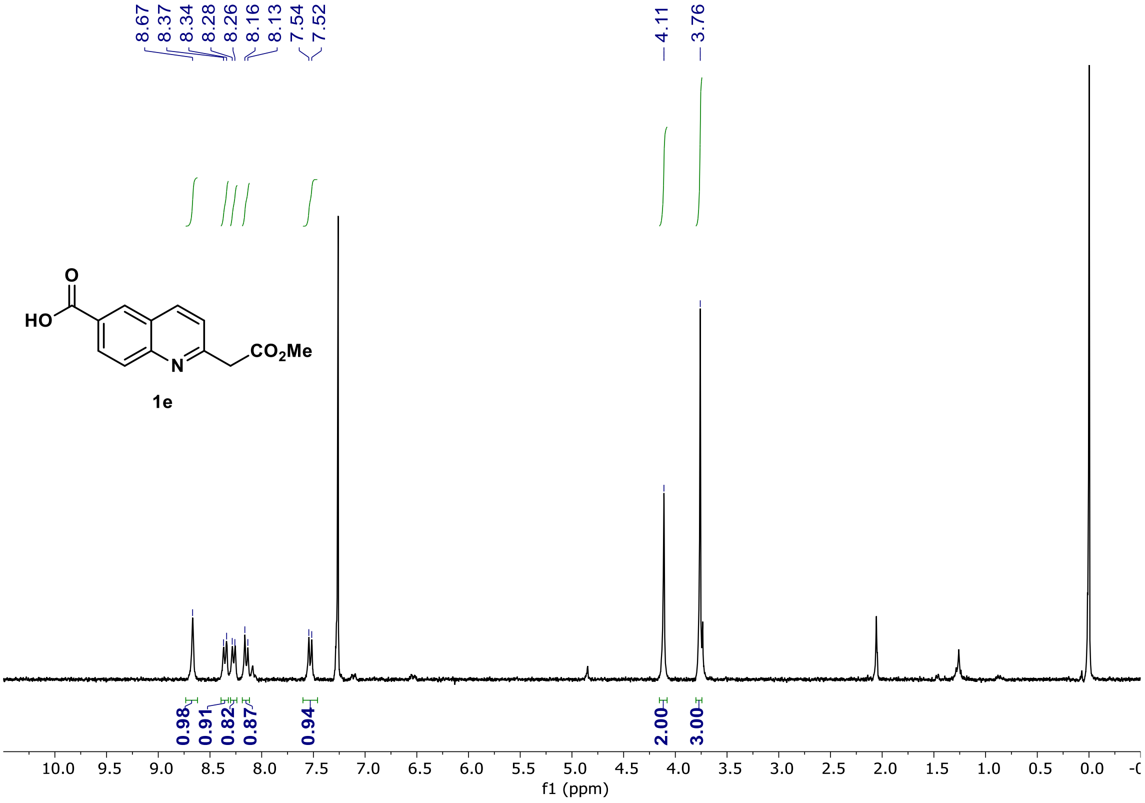

Supplementary Fig. 84. ^{1}H NMR Spectra of **1e**.

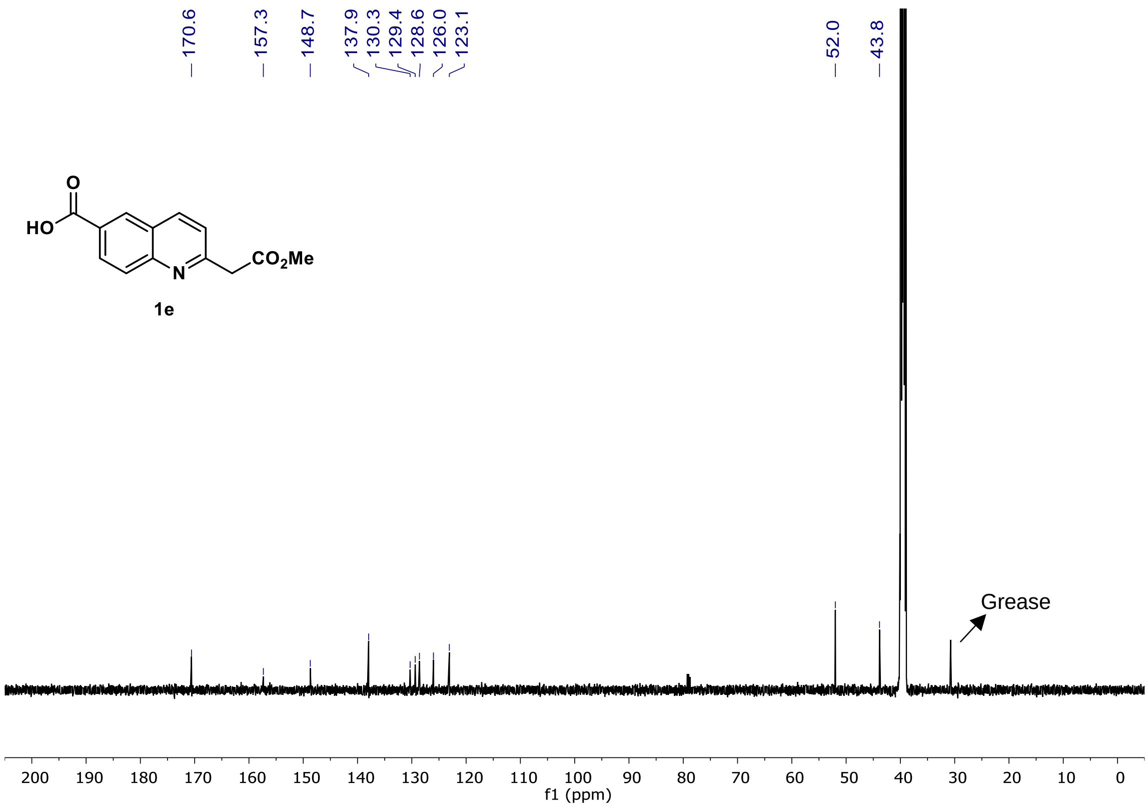

Supplementary Fig. 85. ^{13}C NMR Spectra of **1e**.

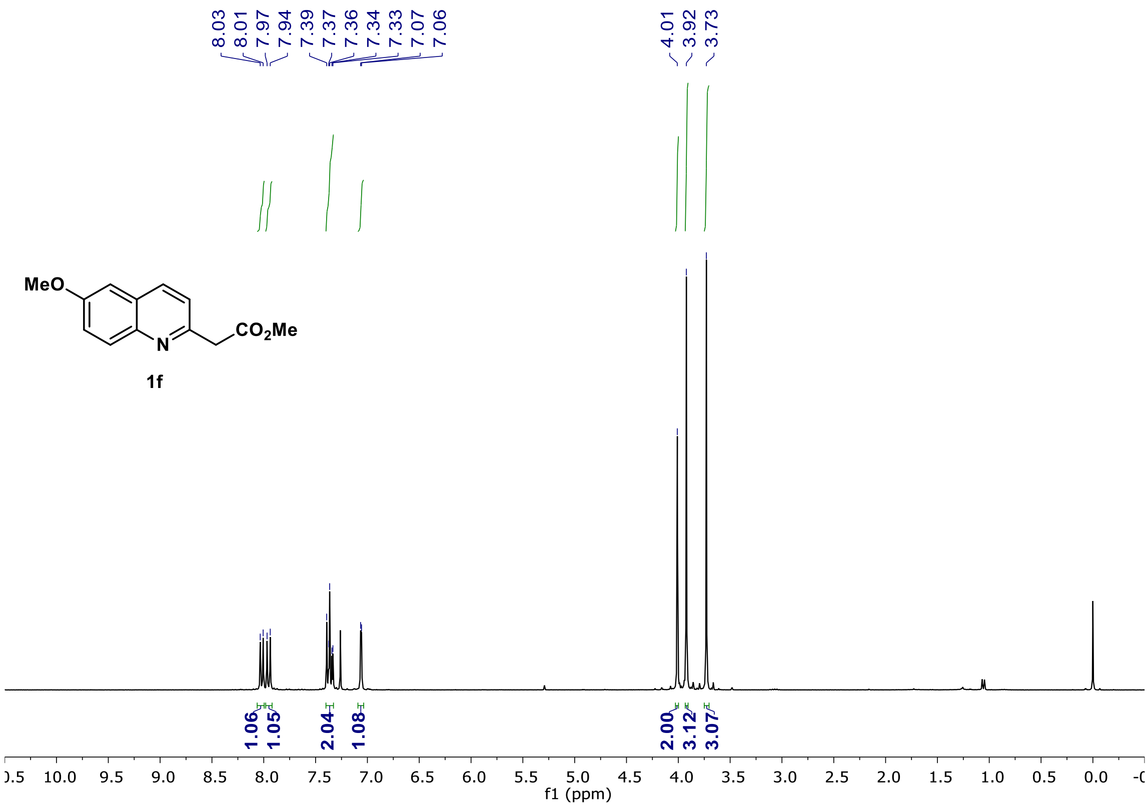

Supplementary Fig. 86. ^{1}H NMR Spectra of **1f**.

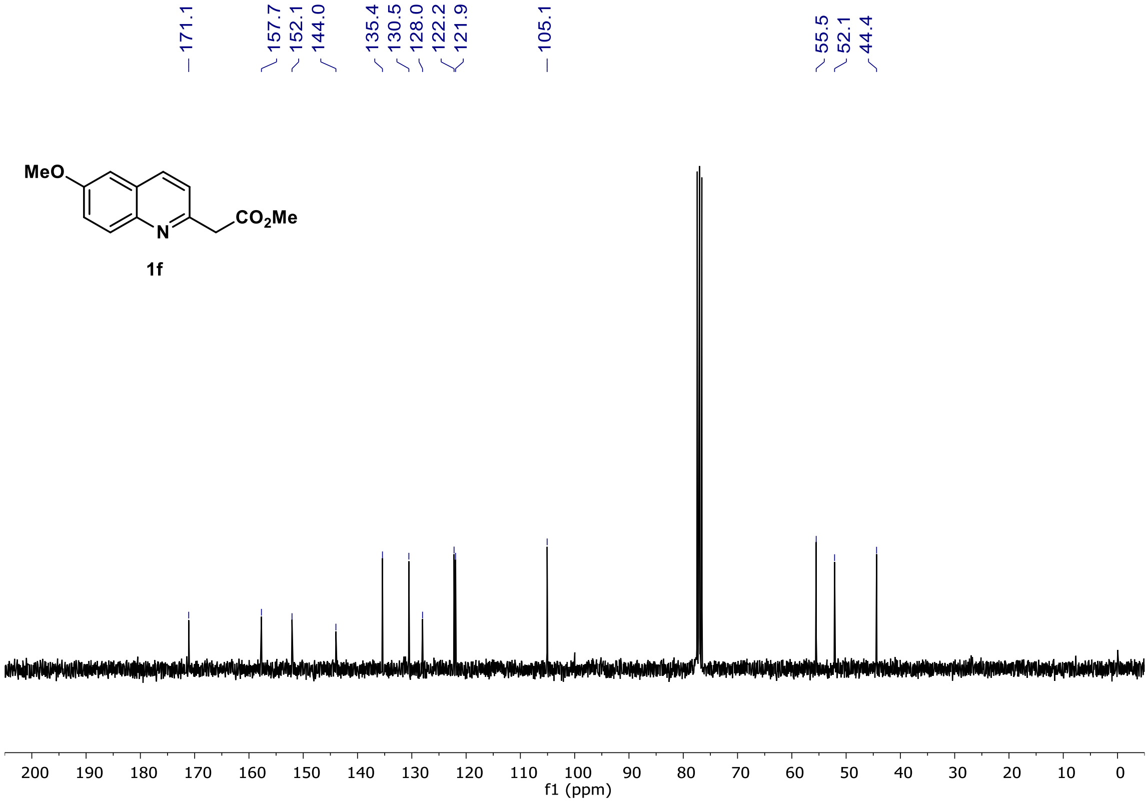

Supplementary Fig. 87. ^{13}C NMR Spectra of **1f**.

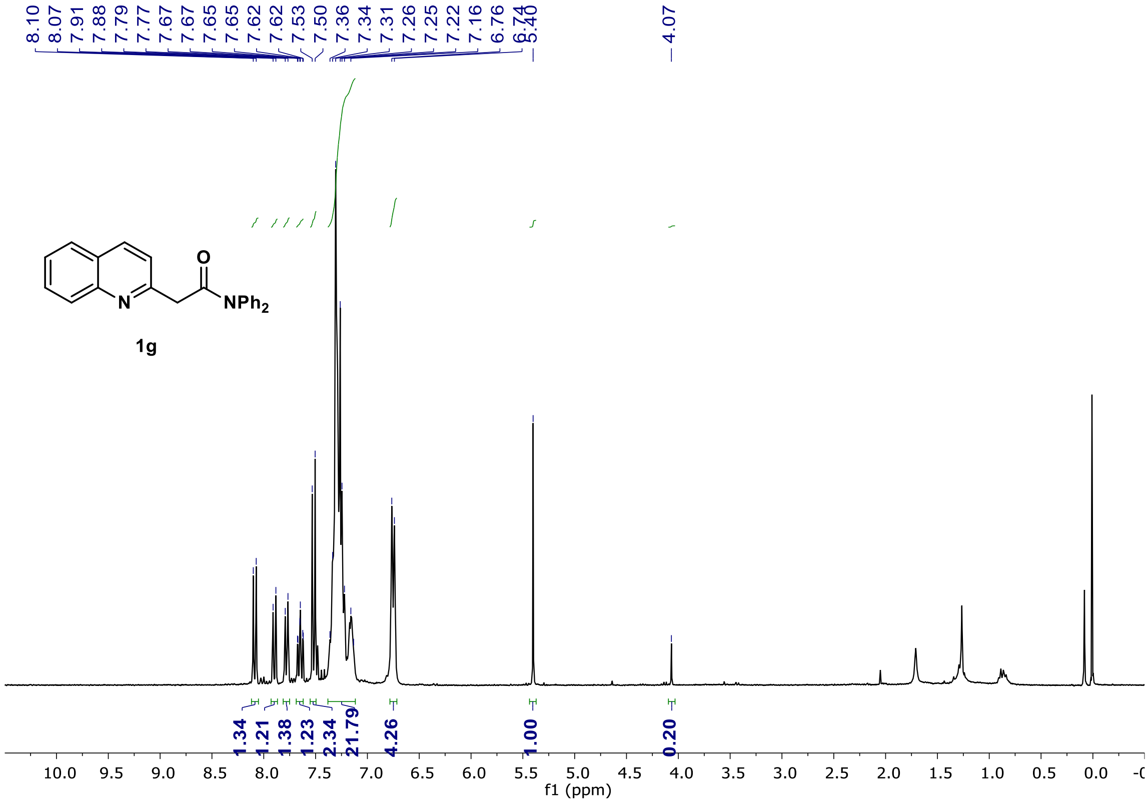

Supplementary Fig. 88. ^{1}H NMR Spectra of **1g**.

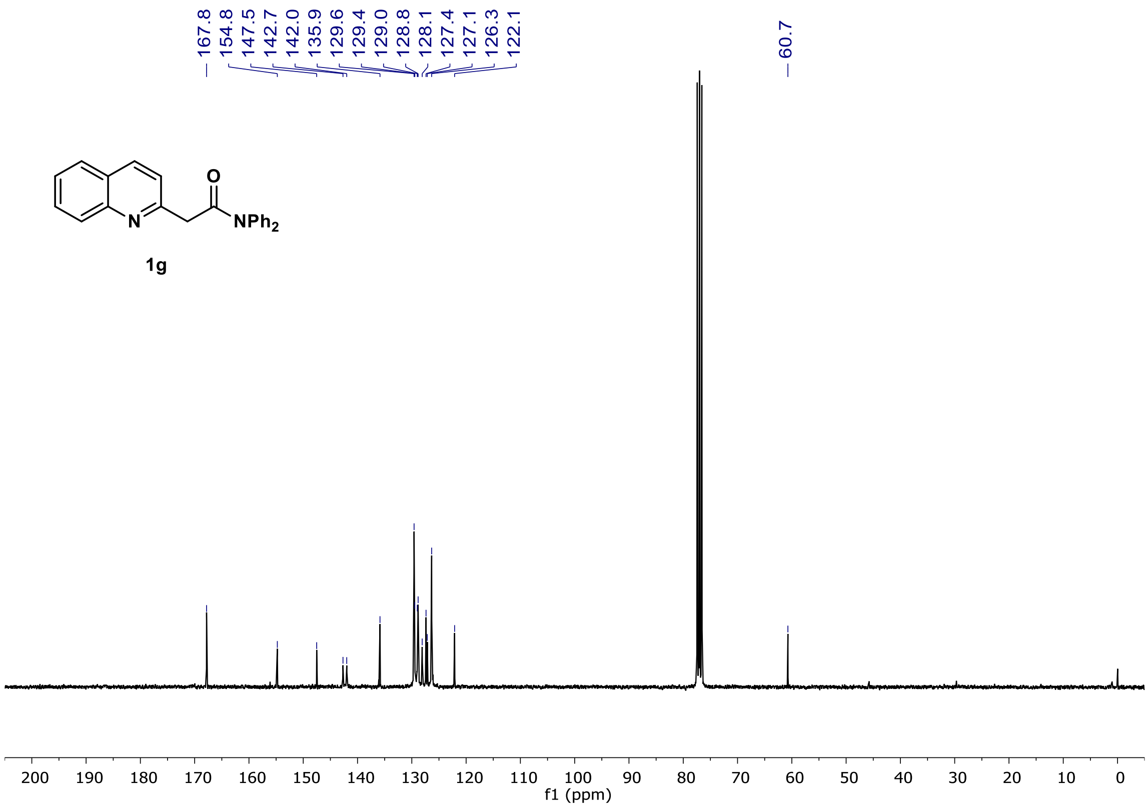

Supplementary Fig. 89. ^{13}C NMR Spectra of **1g**.

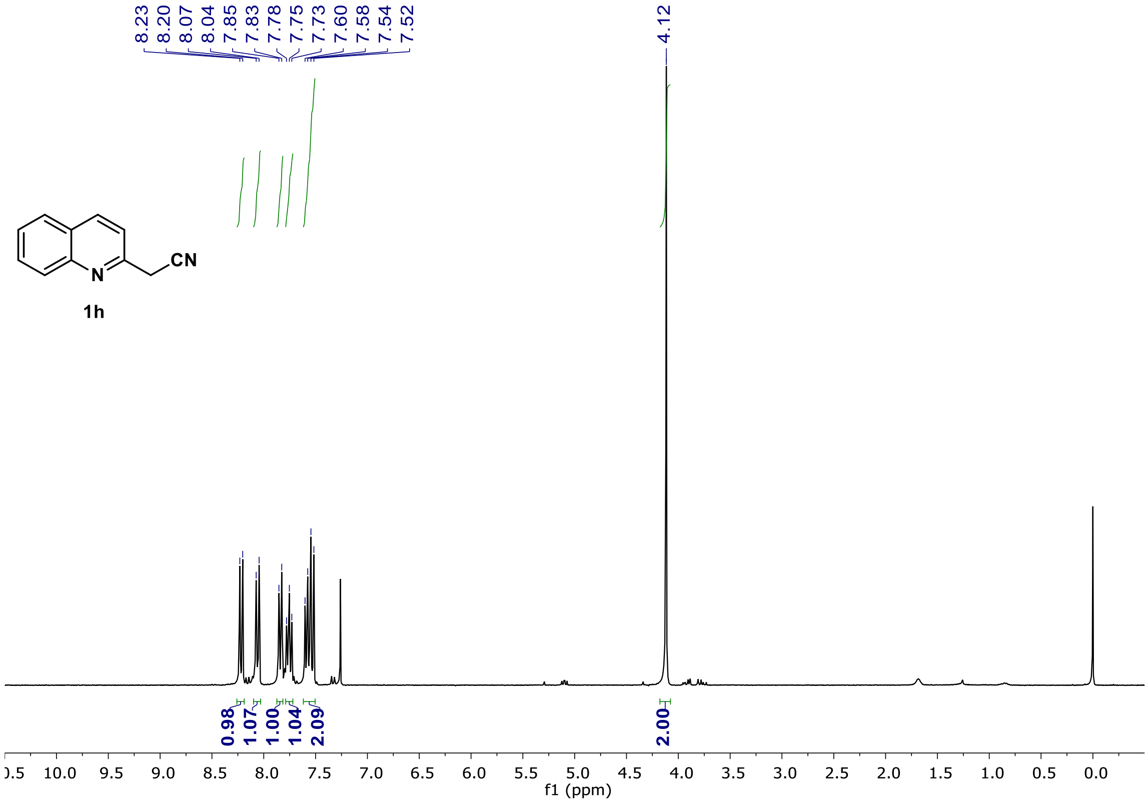

Supplementary Fig. 90. ^{1}H NMR Spectra of **1h**.

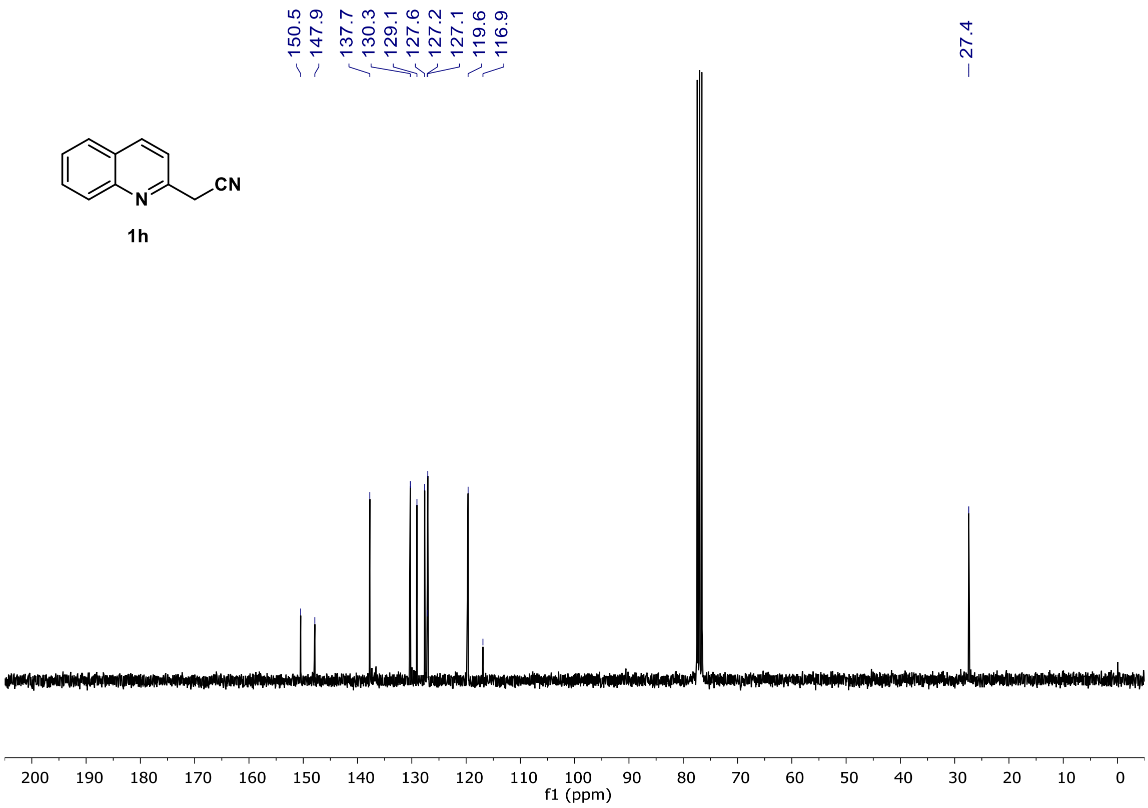

Supplementary Fig. 91. ^{13}C NMR Spectra of **1h**.

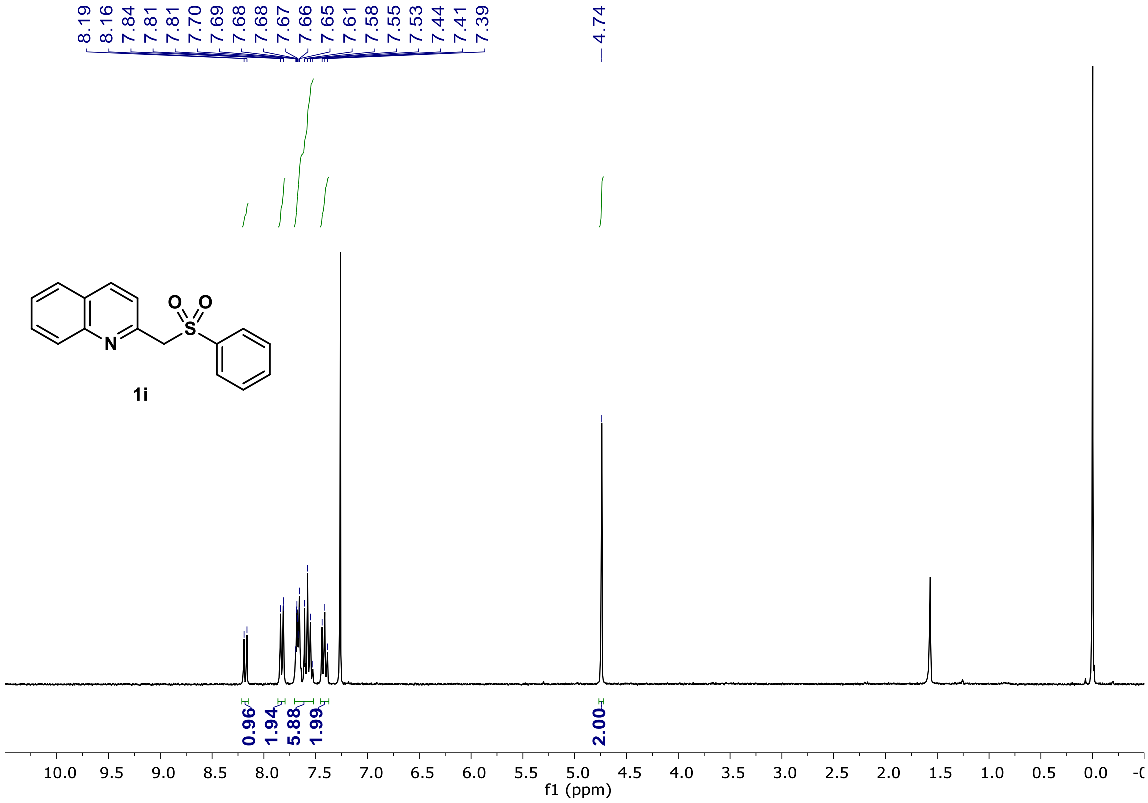

Supplementary Fig. 92. ^{1}H NMR Spectra of **1i**.

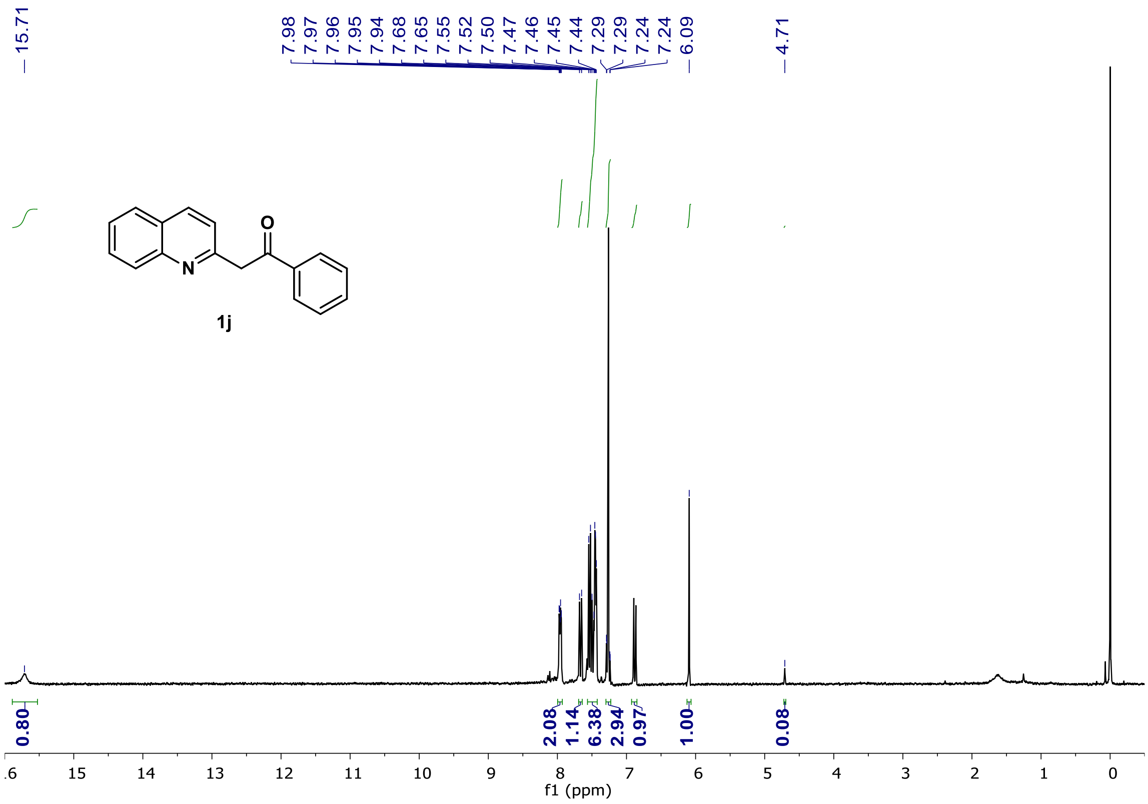

Supplementary Fig. 93. ^{1}H NMR Spectra of **1j**.

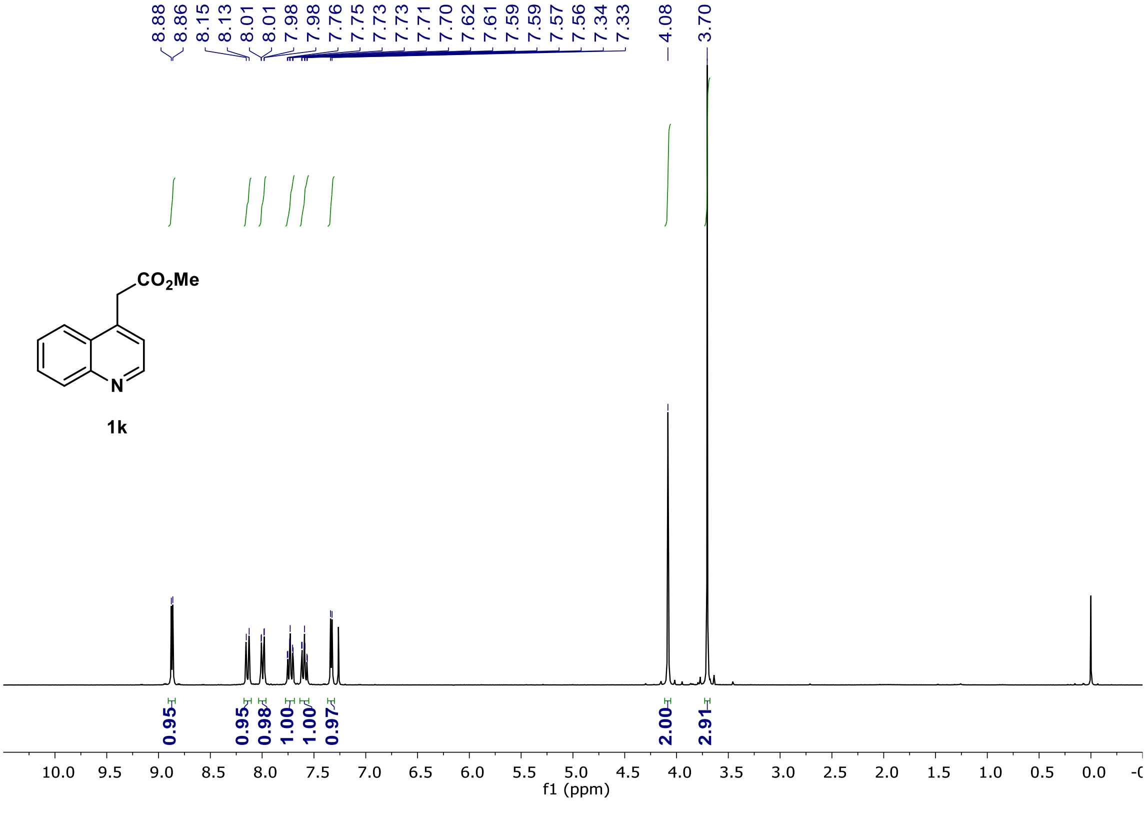

Supplementary Fig. 94. ^{1}H NMR Spectra of **1k**.

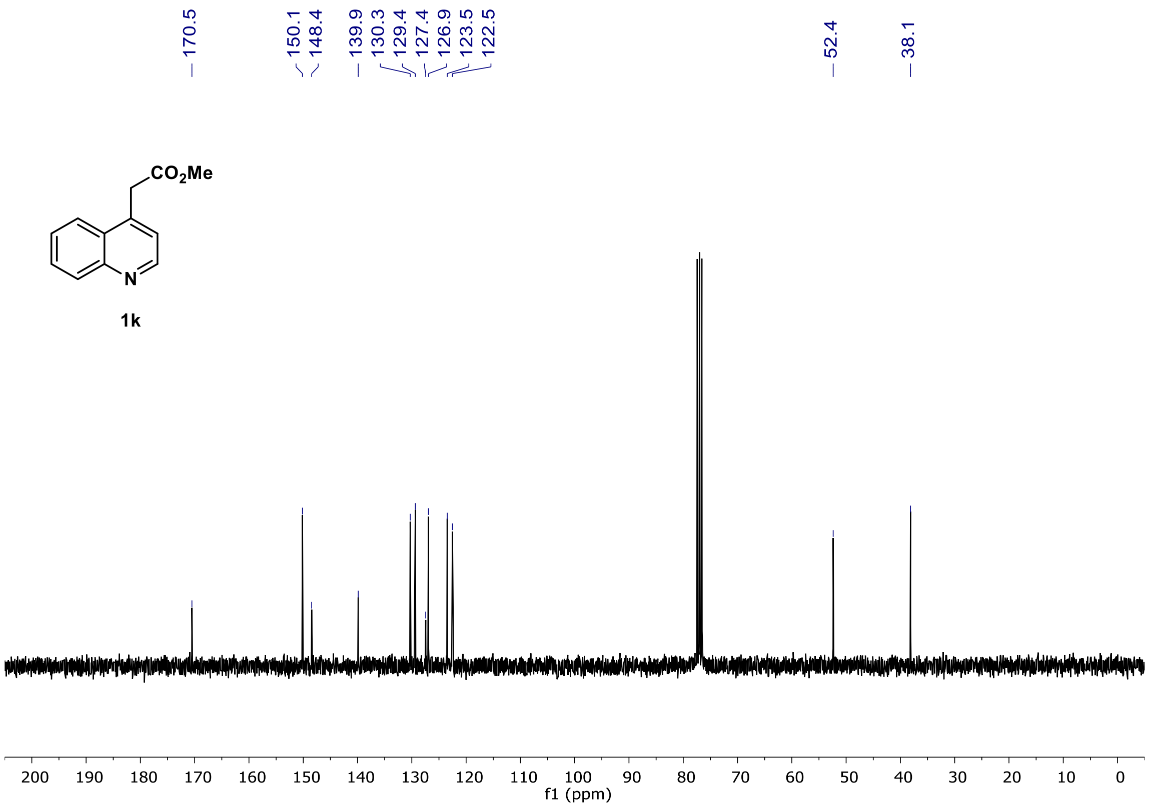

Supplementary Fig. 95. ^{13}C NMR Spectra of **1k**.

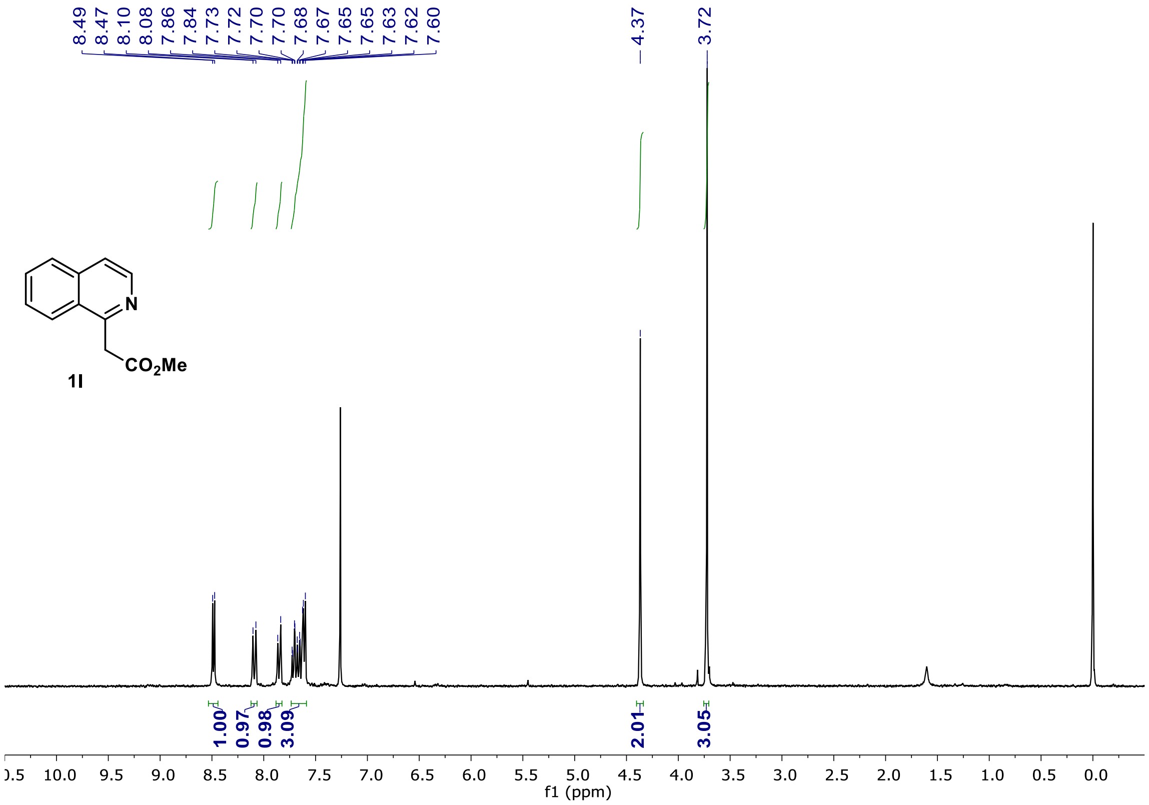

Supplementary Fig. 96. ^{1}H NMR Spectra of **1l**.

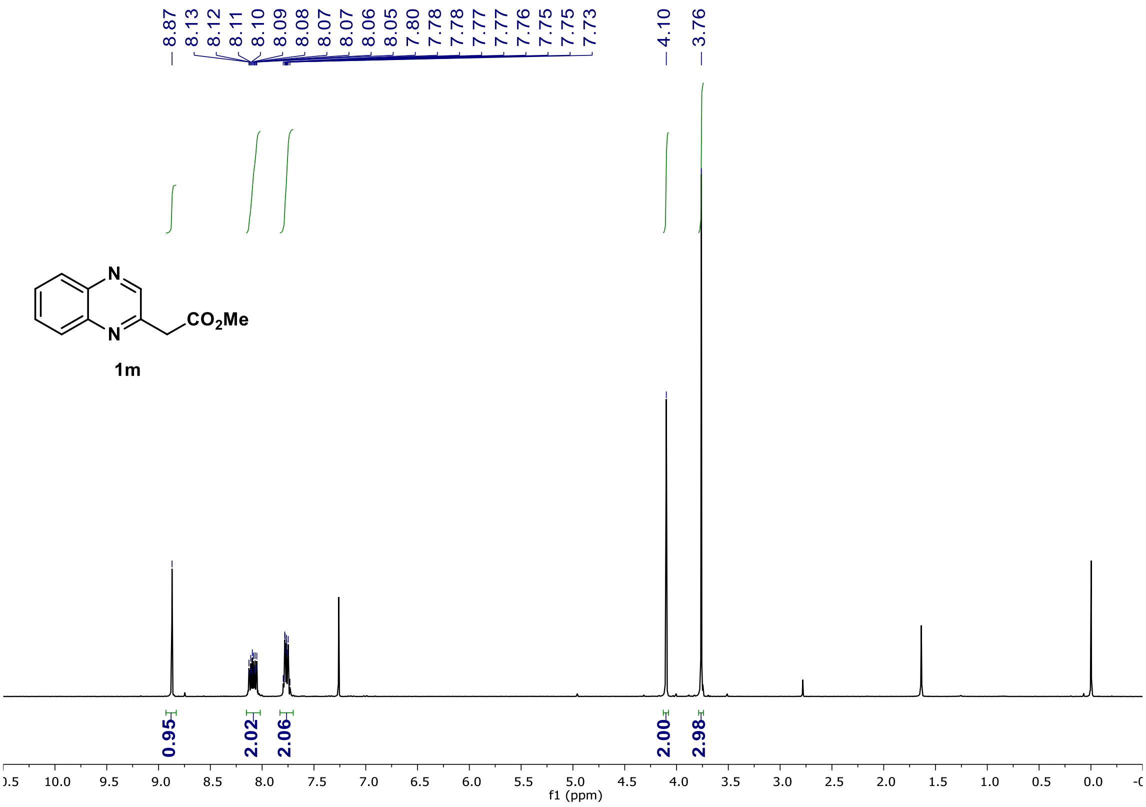

Supplementary Fig. 97. ^{1}H NMR Spectra of **1m**.

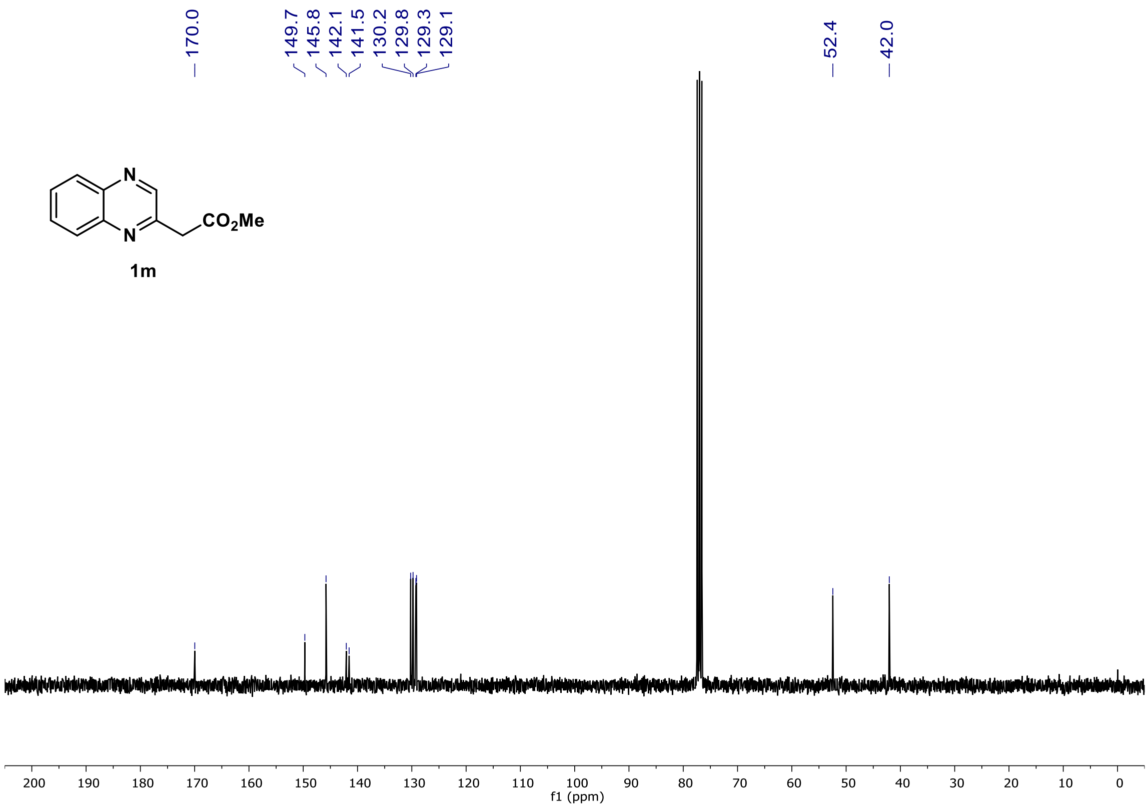

Supplementary Fig. 98. ^{13}C NMR Spectra of **1m**.

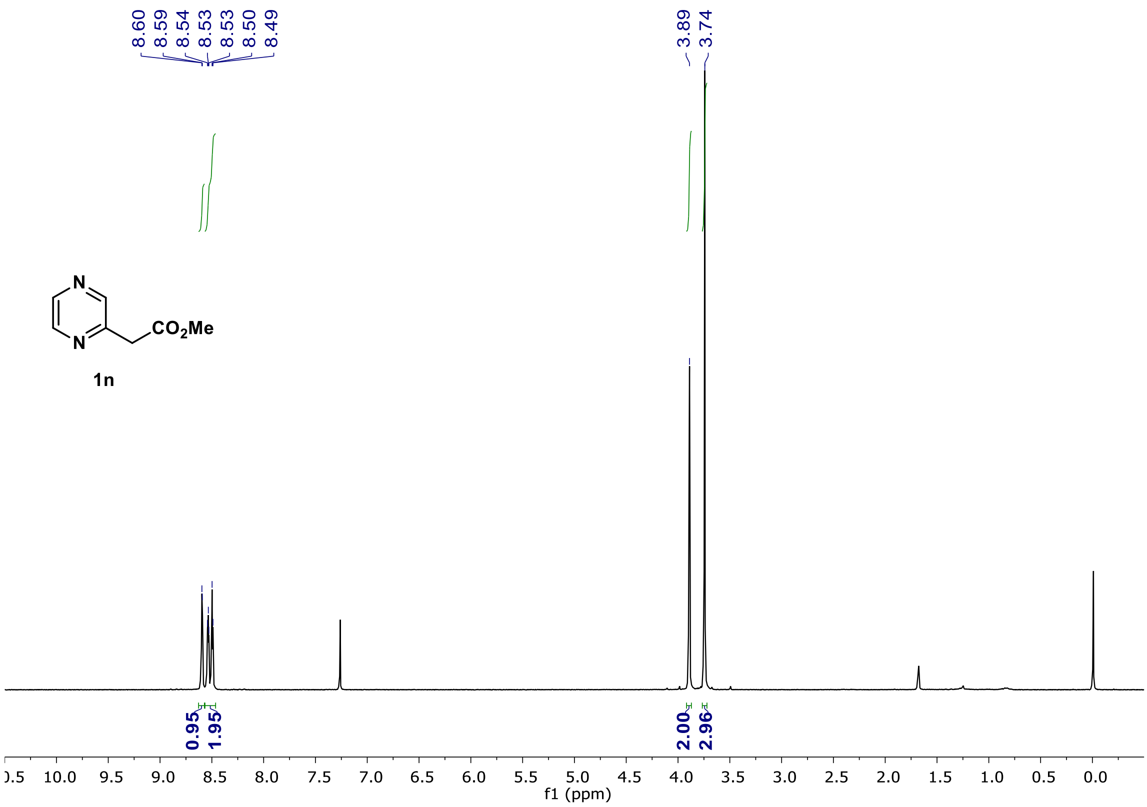

Supplementary Fig. 99. ^{1}H NMR Spectra of **1n**.

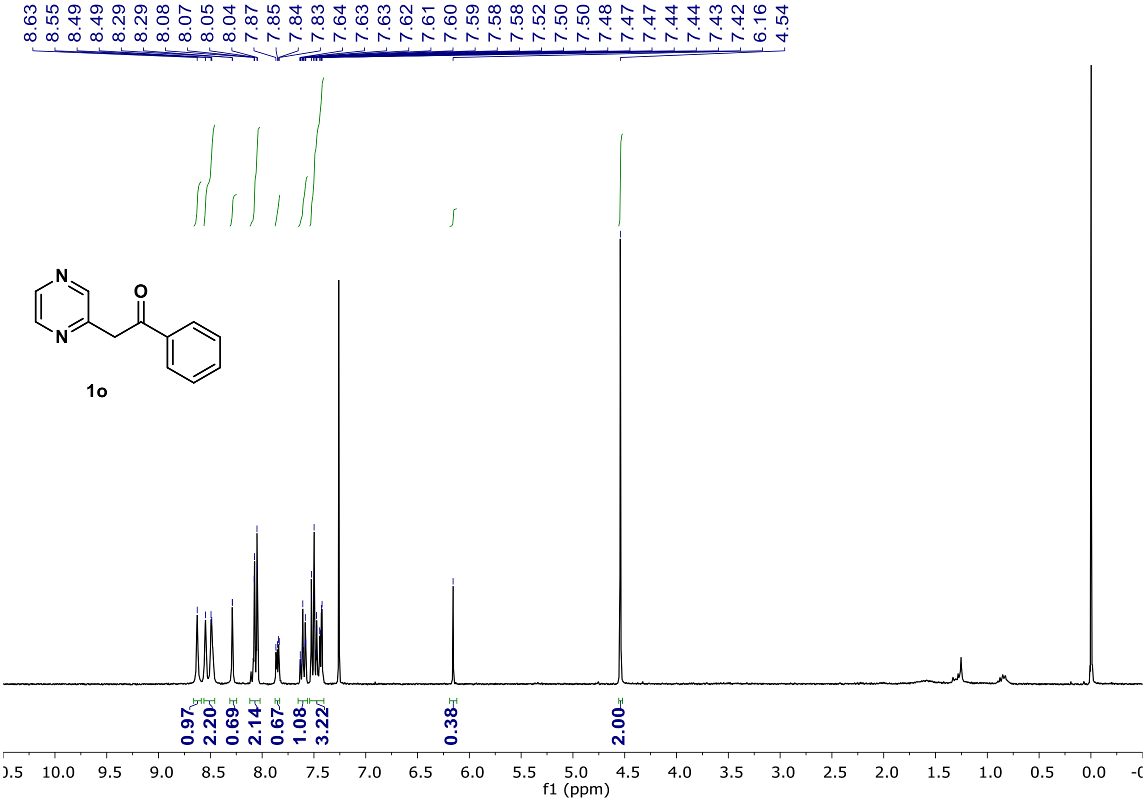

Supplementary Fig. 100. ^{1}H NMR Spectra of **1o**.

Supplementary References

1. Following Karplus equation: $^{3}J_{gauche}$= 2 − 5 Hz and $^{3}J_{anti}$= 8 – 15 Hz. In addition, the coupling constant is in accordance with previous reports: R. Martínez-Haya, L. Marzo, B. König, *Chem. Commun.* **54**, 11602−11605 (2018).

2. Waser, J.; Gaspar, B.; Nambu, H.; Carreira, E. M. Hydrazines and Azides via the Metal-Catalyzed Hydrohydrazination and Hydroazidation of Olefins. *J. Am. Chem. Soc.* **128**, 11693−11712 (2006).

3. Bergamasco, R.; Porter, Q. N.; Yap, C. Vinylindenes and some heteroanalogues in the Diels-Alder reaction. IV. Reactions of ethenetetracarbonitrile with some 3-vinylindoles. *Aust. J. Chem.* **30**, 1531–1544 (1977).

4. Jiang, X.; Boehm, P.; Hartwig, J. F. Stereodivergent Allylation of Azaaryl Acetamides and Acetates by Synergistic Iridium and Copper Catalysis. *J. Am. Chem. Soc.* **140**, 1239–1242 (2018).

5. Xiao, F.; Chen, S.; Chen, Y.; Huang, H.; Deng, G. J. Efficient 2-sulfolmethyl quinoline formation from 2-methylquinolines and sodium sulfinates under transition-metal free conditions. *Chem. Commun.* **51,** 652–654 (2015).

6. Zhang, H.-J.; Yang, Z.-P.; Gu, Q.; You, S.-L. Tandem Pd-Catalyzed Intermolecular Allylic Alkylation/Allylic Dearomatization Reaction of Benzoylmethyl pyridines, Pyrazines, and Quinolines. *Org. Lett.* **21**, 3314–3318 (2019).

7. Rousseaux, S.; Davi, M.; Sofack-Kreutzer, J.; Pierre, C.; Kefalidis, C. E.; Clot, E.; Fagnou, K.; Baudoin, O. Intramolecular Palladium-Catalyzed Alkane C−H Arylation from Aryl Chlorides. *J. Am. Chem. Soc.* **132**, 10706–10716 (2010).

8. Huang, C. Y.; Doyle, A. G. Nickel-Catalyzed Negishi Alkylations of Styrenyl Aziridines. *J. Am. Chem. Soc*. **134**, 9541–9544 (2012).

9. Mato, M.; Herlé, B.; Echavarren, A. M. Cyclopropanation by Gold- or Zinc-Catalyzed Retro-Buchner Reaction at Room Temperature. *Org. Lett*. **20**, 4341–4345 (2018).

10. Cong, F.; Wei, Y.; Tang, P. Combining photoredox and silver catalysis for azidotrifluoromethoxylation of styrenes. *Chem. Commun*. **54**, 4473–4476 (2018).

11. Lee, H.; Lee, Y.; Cho, S. H. Palladium-Catalyzed Chemoselective Negishi Cross-Coupling of Bis[(pinacolato)boryl]methylzinc Halides with Aryl (Pseudo)Halides. *Org. Lett.* **21**, 5912–5916 (2019).

12. Song, S.; Huang, X.; Liang, Y. F.; Tang, C.; Lia, X.; Jiao, N. From simple organobromides or olefins to highly value-added bromohydrins: a versatile performance of dimethyl sulfoxide. *Green. Chem.* **17**, 2727–2731 (2015).

13. A. C. Weedon, in CRC *Handbook of Organic Photochemistry and Photobiology*, ed. W. M. Horspool and P.-S. Song, CRC Press, Boca Raton, 1995, pp. 670–684.

14. Zhao, Y.; Truhlar, D. G. The M06 suite of density functionals for main group thermochemistry, thermochemical kinetics, noncovalent interactions, excited states, and transition elements: two new functionals and systematic testing of four M06-class functionals and 12 other functionals. *Theor. Chem. Acc.* **120**, 215–241 (2008).

15. Hehre, W. J.; Ditchfield, R.; Pople, J. A. Self-Consistent Molecular Orbital Methods. XII. Further Extensions of Gaussian-Type Basis Sets for Use in Molecular Orbital Studies of Organic Molecules. *J. Chem. Phys.* **56**, 2257–2261 (1972).

16. Marenich, A. V.; Cramer, C. J.; Truhlar, D. G. Universal Solvation Model Based on Solute Electron Density and on a Continuum Model of the Solvent Defined by the Bulk Dielectric Constant and Atomic Surface Tensions. *J. Phys. Chem. B.* **113**, 6378–6396 (2009).

17. Frisch, M. J.; Trucks, G. W.; Schlegel, H. B.; Scuseria, G. E.; Robb, M. A.; Cheeseman, J. R.; Scalmani, G.; Barone, V.; Mennucci, B.; Petersson, G. A. et al., Gaussian 09 Revision E.01, Wallingford CT: Gaussian, Inc., 2009.
